# Metagenomic assembly of new (sub)polar *Cyanobacteria* and their associated microbiome from non-axenic cultures

**DOI:** 10.1099/mgen.0.000212

**Published:** 2018-08-23

**Authors:** Luc Cornet, Amandine R. Bertrand, Marc Hanikenne, Emmanuelle J. Javaux, Annick Wilmotte, Denis Baurain

**Affiliations:** ^1^​ InBioS – PhytoSYSTEMS, Eukaryotic Phylogenomics, University of Liège, Liège, Belgium; ^2^​ UR Geology – Palaeobiogeology-Palaeobotany-Palaeopalynology, University of Liège, Liège, Belgium; ^3^​ InBioS – PhytoSYSTEMS, Functional Genomics and Plant Molecular Imaging, University of Liège, Liège, Belgium; ^4^​ InBioS – CIP, Centre for Protein Engineering, University of Liège, Liège, Belgium

**Keywords:** *Cyanobacteria*, Arctic, microbiome, metagenomics, phylogenomic analysis, Antarctic

## Abstract

*Cyanobacteria* form one of the most diversified phyla of *Bacteria*. They are important ecologically as primary producers, for Earth evolution and biotechnological applications. Yet, *Cyanobacteria* are notably difficult to purify and grow axenically, and most strains in culture collections contain heterotrophic bacteria that were probably associated with *Cyanobacteria* in the environment. Obtaining cyanobacterial DNA without contaminant sequences is thus a challenging and time-consuming task. Here, we describe a metagenomic pipeline that enables the easy recovery of genomes from non-axenic cultures. We tested this pipeline on 17 cyanobacterial cultures from the BCCM/ULC public collection and generated novel genome sequences for 12 polar or subpolar strains and three temperate ones, including three early-branching organisms that will be useful for phylogenomics. In parallel, we assembled 31 co-cultivated bacteria (12 nearly complete) from the same cultures and showed that they mostly belong to *Bacteroidetes* and *Proteobacteria*, some of them being very closely related in spite of geographically distant sampling sites.

## Data Summary

1. Cornet L and Baurain D, National Center for Biotechnology Information (NCBI) BioProject, accession PRJNA436342 (2018).

2. Cornet L and Baurain D, National Center for Biotechnology Information (NCBI) Sequence Read Archive (SRA), accessions SAMN08623419 to SAMN08623435 (2018).

3. Cornet L and Baurain D, National Center for Biotechnology Information (NCBI) DDBJ/ENA/GenBank, accessions QBLS00000000 to QBLZ00000000 and QBMA00000000 to QBMS00000000 (2018).

4. Cornet L and Baurain D, National Center for Biotechnology Information (NCBI) BioSample, accessions SAMN08895976 to SAMN0889600 (2018).

**Impact Statement**

Complete genomes of cold-adapted *Cyanobacteria* are underrepresented in databases, due to the difficulty of growing them axenically. In this work, we report the genome sequencing of 12 (sub)polar and three temperate *Cyanobacteria*, along with 21 *Proteobacteria* and five *Bacteroidetes* recovered from their microbiome. Following the use of a state-of-the-art metagenomic pipeline, 12 of our new cyanobacterial genome assemblies are of high quality, which indicates that even non-axenic cultures can yield complete genomes suitable for phylogenomics and comparative genomics. Beyond this main theme, we also address two methodological issues in self-standing Supplemental Appendices. Firstly, we investigated the fate of small subunit rRNA (16S) genes during metagenomic binning and observe that multi-copy rRNA operons are lost because of their higher sequencing coverage and divergent tetranucleotide frequencies. Secondly, we devised a measure of genomic identity to compare metagenomic bins of different completeness, which allowed us to show that *Cyanobacteria*-associated bacteria can be closely related in spite of considerable geographical distance between collection points.

## Introduction

*Cyanobacteria*, also called blue-green algae, are an intensively studied group of prokaryotes. This focus is notably due to their ecological importance, as they colonize a very diverse range of ecosystems and are a major component of the phytoplankton [1, 2]. They are also of primary interest in terms of evolution and palaeobiogeology, cyanobacteria having been present on Earth since the Proterozoic [3–5]. Emergence of oxygenic photosynthesis in this phylum, which led to the Great Oxygenation Event (GOE) around 2.4 billion years ago, had a critical impact on early Earth and evolution by increasing the level of free oxygen and subsequently creating new ecological niches [6–8]. Moreover, *Cyanobacteria* played a role in another major biological event, the spread of photosynthesis to eukaryotic lineages through an initial endosymbiosis termed ‘primary’, followed by several higher-order endosymbioses [9]. Finally, *Cyanobacteria* produce a large number of bioactive compounds (e.g. alkaloids, non-ribosomal peptides, polyketides), which make them promising for both biotechnological and biomedical applications [10–12]. The generation of an axenic cyanobacterial culture is notoriously difficult [1], especially for polar strains [13], and hence the need for tedious purification protocols [14]. In consequence, all cyanobacterial culture collections include many non-axenic cultures (e.g. American Type Culture Collection, ATCC; Czech Collection of Algae and Cyanobacteria, CCALA; University of Toronto Culture Collection of Algae and Cyanobacteria, UTCC; Culture Collection of Algae at the University of Texas, UTEX), with the notable exception of the Pasteur Culture Collection of Cyanobacteria, PCC. The difficulty of reaching axenicity results from bacterial communities living in close relationship with *Cyanobacteria* in nature. This microbiome has been described both from environmental samples [15–19] and from non-axenic cultures [20–22]. Moreover, *Bacteria*/*Cyanobacteria* associations appear to be stable in culture, as no significant differences could be found between bacterial communities accompanying *Cyanobacteria* in fresh samples and collection cultures [21]. Complex trophic interactions between *Cyanobacteria* and other bacterial phyla feeding on their sheaths, such as *Proteobacteria* and *Bacteroidetes*, have been described [23], as well as specific interactions, such as adhesion to heterocysts [20]. The presence of these bacterial communities consequently limits the use of non-axenic cyanobacterial cultures for genomic applications, because fragments of their genomes can eventually become part of published cyanobacterial genomes. Hence, we have recently shown that a large proportion (52 %) of publicly available genomes of *Cyanobacteria* are contaminated by such foreign sequences [24]. In 5 % of the surveyed genomes, these non-cyanobacterial contaminants even reach up to 41.5 % of the genome sequences deposited in the databases.

Owing to their clear scientific interest, obtaining authentic genome sequences of *Cyanobacteria* is an important issue. During the last decade, the rise of metagenomics has allowed an ever-better separation of the different components of a mixture of organisms, based on various properties of the metagenomic contigs, e.g. sequencing coverage and oligonucleotide signatures [25]. In this work, we use a straightforward pipeline that enables the efficient isolation of cyanobacterial genomes from non-axenic cultures. Easy to set up, this pipeline is composed of state-of-the-art metagenomic tools, metaSPAdes [26], MetaBAT [27], CheckM [28], followed by DIAMOND blastx analyses [29] and SSPACE [30] scaffolding. This pipeline allowed us to assemble 15 novel cyanobacterial genomes (12 high-quality, two medium-quality and one low-quality) from 17 polar, subpolar and temperate cultures of the BCCM/ULC public culture collection hosted by the University of Liège (Belgium), of which three appear to belong to early-branching strains in the cyanobacterial tree of life. In the process, we also characterized 31 different co-cultivated bacteria out of the 17 cyanobacterial cultures. Those ‘contaminant’ organisms mostly belong to *Proteobacteria* and *Bacteroidetes*, and some of them are very closely related to each other. Finally, we investigated why small subunit (SSU) rRNA (16S) genes are often lost during metagenomic binning and developed a new metric to compare genome bins with different levels of completeness.

## Methods

### Cyanobacterial cultures and DNA extraction

The 17 cyanobacterial cultures were selected in order to sequence new genomes of interesting Arctic and Antarctic organisms, the biodiversity of which is still not well known. All the strains used in this study were indeed collected from (sub)polar regions, with the exception of three Belgian strains, ULC335, added to the sequencing batch to obtain the first genome of the genus *Snowella*, and ULC186 and ULC187, both related to the (sub)polar strains but of temperate origin. All the *Cyanobacteria* from the present study are from freshwater. The cultures (deposited in the BCCM/ULC collection during the period 2011–2014; Table 1) were incubated at 15 °C in BG11 or BG110 medium and exposed to a constant white fluorescent light source (about 40 μmol photons m^−2^ s^−1^) for 4 weeks. DNA was extracted using the GenElute Bacterial Genomic DNA kit (Sigma-Aldrich) following the recommendations of the manufacturer. After control of the integrity of the genomic DNA by electrophoresis and quantification of the dsDNA concentration using the Quan-iT Picogreen dsDNA Assay kit (Thermo Fisher Scientific), a minimum of 1 µg of dsDNA was sent to the sequencing platform.

**Table 1.** Details of the ULC strains All details were extracted from the BCCM/ULC website: http://bccm.belspo.be/about-us/bccm-ulc. RT, room temperature; NA, not applicable.

Assembly	Strain	Name	Type	Prior affiliation	Morphology	Sheath	Deposit date	Habitat	Culture medium	Temperature (°C)
QBLS00000000	ULC187	*Pseudanabaena* sp. FW039	Non-axenic	Clade F	Filamentous	No	2012	Belgium, lake Ri Jaune	BG11	RT
QBML00000000	ULC066	*Pseudanabaena frigida* O-155	Non-axenic	Clade F	Filamentous	No	2011	Canadian Arctic, Bylot Island	BG11	12
QBMK00000000	ULC068	*Pseudanabaena* sp. O-202	Non-axenic	Clade F	Filamentous	No	2011	Canadian Subarctic, Québec, Kuujjuarapik	BG11	12
QBMM00000000	ULC065	*Cyanobium* sp. O-154	Non-axenic	Clade C1	Unicellular	No	2011	Canadian Arctic, Bylot Island	BG11	12
QBMG00000000	ULC082	*Cyanobium* sp. Chester Cone	Non-axenic	Clade C1	Unicellular	No	2011	Antarctica, Livingston Island	BG11	12
QBMF00000000	ULC084	*Cyanobium* sp. Laguna Chica	Non-axenic	Clade C1	Unicellular	No	2011	Antarctica, Livingston Island	BG11	12
QBMH00000000	ULC077	*Leptolyngbya* sp. O-157	Non-axenic	Clade C3	Filamentous	No	2011	Canadian Arctic, Bylot Island	BG11	12
QBMQ00000000	ULC007	*Phormidesmis priestleyi* ANT.LH52.4	Axenic	Clade C3	Filamentous	No	2011	Antarctica, Larsemann Hills	BG11	18
NA	ULC165	*Leptolyngbya* sp. OTC1/1	Non-axenic	Clade C3	Filamentous	Yes	2012	Antarctica, Sor Rondane Mountains	BG11	12
QBMC00000000	ULC129	*Leptolyngbya foveolarum* TM2FOS129	Non-axenic	Clade C3	Filamentous	No	2011	Antarctica, Transantarctic Mountains	BG11	12
QBMP00000000	ULC027	*Phormidium priestleyi* ANT.PROGRESS2.5	Non-axenic	Clade C3	Filamentous	No	2011	Antarctica, Larsemann Hills	BG11	18
QBLT00000000	ULC186	*Leptolyngbya* sp. FW074	Non-axenic	Clade C3	Filamentous	No	2012	Belgium, Renipont lake	BG11	RT
QBMN00000000	ULC041	*Leptolyngbya antarctica* ANT.ACE.1	Non-axenic	Clade C3	Filamentous	No	2011	Antarctica, Vestfold Hills	BG11	12
QBMJ00000000	ULC073	*Leptolyngbya glacialis* TM1FOS73	Non-axenic	Clade C3	Filamentous	Yes	2011	Antarctica, Transantarctic Mountains	BG11	18
QBMS00000000	ULC335	*Snowella* sp. FW024	Non-axenic	Clade B2	Unicellular	Yes	2014	Belgium, lake Falemprise	BG11	RT
NA	ULC146	*Nostoc* sp. ANT.UTS.183	Non-axenic	Clade B1	Filamentous heterocystous	Yes	2012	Antarctica, Sor Rondane Mountains	BG110	18
NA	ULC179	*Nostoc* sp. OTCcontrol	Non-axenic	Clade B2	Filamentous heterocystous	Yes	2012	Antarctica, Sor Rondane Mountains	BG110	12

### Metagenome sequencing and assembly

The 17 cyanobacterial cultures were sequenced (PE 2×250 nt) on the Illumina MiSeq sequencing platform (GIGA Genomics, University of Liège). Nextera XT libraries had a fragment size estimated at 800–900 nt. Raw sequencing reads were trimmed using Trimmomatic v0.35 [31]. Sequencing adapters were removed with the option illuminaclip NexteraPE-PE.fa : 2 : 30 : 20. Trimming values were selected to maximize genome bin sizes (in terms of bp), after preliminary testing. Trailing/leading values were set at 20, the sliding window at 10 : 20, the crop value at 145 and the minimal length at 80. Trimmed paired-end reads were assembled with metaSPAdes v3.10.1 [26] using default settings. Trimmed paired-end reads were then re-mapped on the metaSPAdes assemblies with BamM v1.7.3 (http://ecogenomics.github.io/BamM/), yielding BAM files suitable for the metagenomic analyses. Genome bins were determined with MetaBAT v0.30.1 [27], trying each built-in parameter set in turn (i.e. verysensitive, sensitive, specific, veryspecific and superspecific). CheckM v1.0.7 [28] was then used with the option lineage_wf to select the best MetaBAT parameter set for each metaSPAdes assembly. In practice, we first tried to select the MetaBAT parameter set that was the most suitable for the largest genome bin of a given metagenome (in terms of total assembly length), considering CheckM output statistics in the following order: (1) contamination, (2) strain heterogeneity and (3) completeness. When multiple parameter sets were equally optimal for the largest bin, we turned to the next-largest bin(s) for parameter selection. The non-assignment of a given contig to multiple bins was checked using the unique option of CheckM, while binning accuracy was assessed using the merge and tree_qa options after generating a marker set for *Bacteria*. The automatic taxonomic classification of CheckM was then extracted to determine the nature of each bin, either cyanobacterial or foreign. The strain names of cyanobacterial bins were attributed based on phenotypic observations during cultivation. Bins classified as root (i.e. unclassified) by CheckM were discarded from phylogenomic analyses. Contaminants (with respect to the taxon determined by CheckM) in each genome bin were further characterized using DIAMOND blastx v0.8.22 [29] and the companion parser developed in our article regarding the contamination of public cyanobacterial genomes [24]. To this end, we split the genome bins into non-overlapping pseudo-reads of 250 nt (with a custom Perl script), so as to increase the sensitivity of the analyses. We then used DIAMOND blastx to blast these pseudo-reads against a curated database derived from the release 30 of Ensembl Bacteria that we developed for our genome contamination analyses [24]. In parallel, contigs within each genome bin were scaffolded with SSPACE v.3.0 [30] using default settings, except that contigs were first extended using paired-end reads (-x 1) and that the minimum of read pairs required to compute a scaffold was set to 3 (-k 3). The fragmentation of the scaffolded genome bins was then analysed with QUAST v2.3 [32] using default settings, whereas their sequencing coverage was determined with BBMap v37.24 (http://bbmap.sourceforge.net/). Finally, protein sequences were predicted for all genome bins with Prodigal v2.6.2 [33] using the ab_initio mode. In Appendix S1, we provide the stepwise tutorial describing the set up and use of the metagenomic pipeline.

### Phylogenetic analyses

The complete proteomes of 64 cyanobacterial strains chosen to represent the diversity of the whole phylum were downloaded from the NCBI portal [34]. Details and download links for the selected proteomes are available in Tables 2 and S1 (available in the online version of this article), respectively. Orthology inference was performed with USEARCH v8.1 (64 bits) [35] and OrthoFinder v1.1.2, using the standard inflation parameter of 1.5 [36]. Out of 37  261 orthologous groups (OGs), 675 were selected with classify-ali.pl (part of the Bio-MUST-Core software package; D. Baurain; https://metacpan.org/release/Bio-MUST-Core) by enforcing in each OG the presence of ≥62 different organisms, represented by an average of ≤1.1 gene copy per organism. The 675 OGs were completed with sequences directly mined from the 15 cyanobacterial bins using our software package ‘42’, which strictly controls for orthology during sequence addition [37, 38]. Enriched OGs were then aligned with MAFFT v7.273 [39] and conserved sites were selected with BMGE v1.12 [40] using moderately severe settings (entropy cut-off 0.5, gap cut-off 0.2). A supermatrix of 79 organisms×170 983 unambiguously aligned amino-acid positions (3.9 % missing character states) was assembled with SCaFoS v1.30k [41] using the minimal evolutionary distance criterion for deciding between the few in-paralogous proteins. Finally, a phylogenomic tree was inferred with PhyloBayes-MPI v1.5a under the CAT+Γ4 model [42] by running two independent chains until 1500 cycles were obtained. The tree was rooted on the branch leading to the two *Gloeobacter* species. Convergence of the parameters was assessed using criteria given in the PhyloBayes manual and a conservative burn-in of 620 cycles was used (meandiff=0.04).

**Table 2.** Details regarding reference proteomes All details were extracted from the NCBI metadata.

Assembly	Bioproject	Taxid	Name
GCA_000484535.1	PRJNA162637	1183438	*Gloeobacter kilaueensis* JS1
GCF_000011385.1	PRJNA58011	251221	*Gloeobacter violaceus* PCC 7421
GCF_000013205.1	PRJNA224116	321327	*Synechococcus* sp. JA-3-3Ab
GCF_000013225.1	PRJNA224116	321332	*Synechococcus* sp. JA-2-3B'a(2-13)
GCF_000332275.1	PRJNA224116	195250	*Synechococcus* sp. PCC 7336
GCF_000317065.1	PRJNA224116	82654	*Pseudanabaena* sp. PCC 7367
GCF_000332215.1	PRJNA224116	927668	*Pseudanabaena biceps* PCC 7429
GCF_000317085.1	PRJNA224116	1173263	*Synechococcus* sp. PCC 7502
GCF_000332175.1	PRJNA224116	118173	*Pseudanabaena* sp. PCC 6802
GCF_000018105.1	PRJNA224116	329726	*Acaryochloris marina* MBIC11017
GCA_000022045.1	PRJNA28337	395961	*Cyanothece* sp. PCC 7425
GCF_000505665.1	PRJNA224116	1394889	*Thermosynechococcus* sp. NK55a
GCF_000316685.1	PRJNA224116	195253	*Synechococcus* sp. PCC 6312
GCF_000775285.1	PRJNA224116	1497020	*Neosynechococcus sphagnicola* sy1
GCF_000309945.1	PRJNA224116	864702	*Oscillatoriales cyanobacterium* JSC-12
GCF_001895925.1	PRJNA224116	1920490	*Phormidesmis priestleyi* ULC007
GCF_001650195.1	PRJNA224116	1850361	*Phormidesmis priestleyi* BC1401
GCF_000353285.1	PRJNA224116	272134	*Leptolyngbya boryana* PCC 6306
GCF_000733415.1	PRJNA224116	1487953	*Leptolyngbya* sp. JSC-1
GCF_000332095.2	PRJNA224116	1173264	*Leptolyngbya* sp. PCC 6406
GCF_000763385.1	PRJNA224116	1229172	*Leptolyngbya* sp. KIOST-1
GCF_000309385.1	PRJNA224116	118166	*Nodosilinea nodulosa* PCC 7104
GCF_000155595.1	PRJNA224116	91464	*Synechococcus* sp. PCC 7335
GCF_000482245.1	PRJNA224116	1385935	*Leptolyngbya* sp. Heron Island J
GCF_000316115.1	PRJNA224116	102129	*Leptolyngbya* sp. PCC 7375
GCF_000464785.1	PRJNA224116	1255374	*Planktothrix rubescens* NIVA-CYA 407
GCF_000175415.3	PRJNA224116	634502	*Arthrospira platensis* str. Paraca
GCF_000478195.2	PRJNA224116	1348334	*Lyngbya aestuarii* BL J
GCF_000332155.1	PRJNA224116	402777	*Kamptonema formosum* PCC 6407
GCF_000317475.1	PRJNA224116	179408	*Oscillatoria nigro-viridis* PCC 7112
GCF_000317105.1	PRJNA224116	56110	*Oscillatoria acuminata* PCC 6304
GCF_000317515.1	PRJNA224116	1173027	*Microcoleus* sp. PCC 7113
GCF_000021825.1	PRJNA224116	65393	*Cyanothece* sp. PCC 7424
GCA_000307995.2	PRJEA88171	1160280	*Microcystis aeruginosa* PCC 9432
GCF_000021805.1	PRJNA224116	41431	*Cyanothece* sp. PCC 8801
GCF_000737945.1	PRJNA256120	1527444	*Candidatus Atelocyanobacterium thalassa* isolate SIO64986
GCF_000284135.1	PRJNA224116	1080228	*Synechocystis* sp. PCC 6803 substr. GT-I
GCF_000715475.1	PRJNA224116	490193	*Synechococcus* sp. NKBG042902
GCF_000317655.1	PRJNA39697	292563	*Cyanobacterium stanieri* PCC 7202
GCF_000332055.1	PRJNA224116	102125	*Xenococcus* sp. PCC 7305
GCF_000317575.1	PRJNA224116	111780	*Stanieria cyanosphaera* PCC 7437
GCF_000380225.1	PRJNA224116	1128427	filamentous cyanobacterium ESFC-1
GCF_000317615.1	PRJNA224116	13035	*Dactylococcopsis salina* PCC 8305
GCF_000317495.1	PRJNA224116	1173022	*Crinalium epipsammum* PCC 9333
GCF_000317555.1	PRJNA224116	1173026	*Gloeocapsa* sp. PCC 7428
GCF_000317125.1	PRJNA224116	251229	*Chroococcidiopsis thermalis* PCC 7203
GCF_000582685.1	PRJNA224116	1469607	[*Scytonema hofmann*i] UTEX 2349
GCF_000789435.1	PRJNA224116	1532906	*Aphanizomenon flos-aquae* 2012/KM1/D3
GCF_000196515.1	PRJNA224116	551115	'*Nostoc azollae*' 0708
GCF_000316645.1	PRJNA224116	28072	*Nostoc* sp. PCC 7524
GCF_000204075.1	PRJNA10642	240292	*Anabaena variabilis* ATCC 29413
GCA_000340565.3	PRJNA185469	313624	*Nodularia spumigena* CCY9414
GCF_000020025.1	PRJNA224116	63737	*Nostoc punctiforme* PCC 73102
GCF_000332295.1	PRJNA224116	643473	*Fortiea contorta* PCC 7126
GCF_000346485.2	PRJNA224116	128403	*Scytonema hofmannii* PCC 7110
GCF_000734895.2	PRJNA224116	1337936	*Calothrix* sp. 336/3
GCF_000332255.1	PRJNA224116	1173021	cyanobacterium PCC 7702
GCF_000317225.1	PRJNA224116	98439	*Fischerella thermalis* PCC 7521
GCF_000012525.1	PRJNA224116	1140	*Synechococcus elongatus* PCC 7942
GCF_000586015.1	PRJNA224116	1451353	*Candidatus Synechococcus* spongiarum SH4
GCF_000155635.1	PRJNA224116	180281	*Cyanobium* sp. PCC 7001
GCA_000015705.1	PRJNA13496	59922	*Prochlorococcus marinus* str. MIT 9303
GCF_000011485.1	PRJNA224116	74547	*Prochlorococcus marinus* str. MIT 9313
GCF_000153805.1	PRJNA224116	313625	*Synechococcus* sp. BL107

To study the nature of the organisms co-cultivated in the cyanobacterial cultures, we relied on the release 1.4.0 of the RiboDB database [43] as a taxonomic reference. To this end, the 53 files corresponding to ribosomal proteins occurring in *Bacteria* were downloaded and aligned with MAFFT. The script ali2phylip.pl (part of Bio-MUST-Core) was then used to discard alignment sites with >50 % missing character states. Concatenation of the 53 alignments with SCaFoS yielded a supermatrix of 3474 organisms×6612 unambiguously aligned amino-acid positions (5.4 % missing character states) that was used to infer a preliminary tree with RAxML v8.1.17 [44] under the LG4X model (data not shown). This large ribosomal protein tree allowed us to select representative organisms based on patristic distances in order to maximize diversity. At a minimum distance of 0.7 substitutions per site, 200 organisms were retained using treeplot (from the MUST software package; [45]). Visual inspection of the tree inferred from this smaller dataset led us to further discard four fast-evolving organisms, yielding a total of 196 representative organisms. Both the large (3474 organisms) and the small (196 organisms) datasets were used in subsequent analyses. Hence, the 53 alignments (both large and small versions) were enriched (using again ‘42’) with sequences from the foreign (i.e. non-cyanobacterial) bins assembled from our 17 cyanobacterial cultures (31 bins in total, excluding unclassified CheckM bins). To control the origins of the enriching sequences, taxonomic filters of ‘42’ were enabled, so as to require all new sequences to belong to the taxon determined by CheckM during its analysis of each whole bin. After this step, four incomplete genome bins (ULC066-bin3, ULC073-bin4, ULC082-bin4, ULC146-bin6) were discarded due to their low prevalence in the alignments (<10 %). Enriched alignments were then processed as above with either ali2phylip.pl (large dataset) or BMGE (small dataset). The two resulting supermatrices assembled with SCaFoS contained 3501 organisms ×6613 unambiguously aligned amino-acid positions (6.0 % missing character states) and 223 organisms×7060 unambiguously aligned amino-acid positions (7.8 % missing character states), respectively. Finally, two different trees were inferred using either RAxML (large dataset) or PhyloBayes (small dataset). The trees were rooted on the branch leading to *Archaea*.

All phylogenetic trees were formatted using the script format-tree.pl (part of Bio-MUST-Core), FigTree v1.4.2 (http://tree.bio.ed.ac.uk/software/figtree/) and further arranged in InkScape v0.92 [46].

### SSU rRNA (16S) analyses

SSU rRNA (16S) genes were predicted using RNAmmer v1.2 [47] in all genome bins for the selected MetaBAT parameter set. Beyond regular bins, we also investigated an additional bin (called nobin) for each metagenome, which contained all the scaffolds rejected by MetaBAT during the binning process. Predicted rRNA sequences were taxonomically classified by sina v1.2.11 [48], using release 128 of the silva database composed of 1 922 213 SSU rRNA reference sequences [49].

## Results

### Metagenome sequencing and assembly

We obtained a total of 55 different genome bins from the separate sequencing and metagenomic assembly of the 17 cyanobacterial cultures (Table 3). Among those, we identified 15 bins as cyanobacterial (ULC007-bin1, ULC027-bin1, ULC041-bin1, ULC065-bin1, ULC066-bin1, ULC068-bin1, ULC073-bin1, ULC077-bin1, ULC082-bin1, ULC084-bin3, ULC129-bin1, ULC165-bin4, ULC186-bin1, ULC187-bin1, ULC335-bin1), based on CheckM classification ﻿[28], except for ULC165-bin4, which was classified after DIAMOND blastx results. For the two *Nostocales* strains (ULC146 and ULC179), we failed to recover any cyanobacterial bin (but see below for the analysis of the other bins). For 12 metagenomes, the cyanobacterial bin corresponded to the largest predicted bin, in terms of both total length and sequencing coverage (Table 3; see also Appendix S2). For two cultures, however, cyanobacterial bins were the smallest predicted (ULC084-bin3 and ULC165-bin4). Genome completeness, evaluated with CheckM, was ≥90 % [median=97.74 %, interquartile range (IQR)=4.04 %] for all cyanobacterial bins but lower for ULC165-bin4 (24.14 %). As expected, completeness correlated positively with the sequencing coverage of the bins in the metagenomic assemblies, but this correlation was barely significant (Pearson's *r*=0.52, *P*=0.05). The contamination level was evaluated to be <1.63 % (median=0.47 %, IQR=0.83 %) with CheckM and <2.62 % (median=1.26 %, IQR=0.40 %) with our DIAMOND blastx parser [24]. As our libraries were only composed of paired-ends (and not of mate pairs), the number of scaffolds obtained after metaSPAdes assembly and SSPACE scaffolding was ≥60 for all cyanobacterial genome bins (median=238, IQR=292) (Tables 3 and S2).

**Table 3.** Assembly statistics, taxonomy, completeness, contamination and coverage of genome bins The taxonomic label (CM taxon), genome completeness (CM compl.) and contamination level (CM contam.) were computed with CheckM. Sequencing coverage (med) was computed with BBMap, while bin length was extracted from QUAST output. Length (%) represents the proportion of assembled data in a bin with respect to the total amount of data of the corresponding metagenome. In the Nature column, cyanobacterial bins are denoted by C, microbiome bins by M, unclassified bins by U and nobins by No. Genome bins used in phylogenetic inference are marked by an asterisk (*) and discarded bins by a dash (−). NA, not applicable.

Strain	MetaBAT setting	Bin	CM taxon	Nature	No. of scaffolds	Length (%)	Coverage (med)	CM compl.	CM contam.
ULC335	Veryspecific	1	Cyanobacteria*	C	238	20.84	10.90	98.91	0.51
		2	Flavobacteriaceae*	M	67	13.73	11.12	99.29	0.12
		3	Bacteroidetes*	M	576	12.83	4.46	65.45	0.49
		4	Alphaproteobacteria*	M	271	4.79	4.13	32.28	0
		0	Nobin	No	23 056	47.81	1.88	na	na
ULC007	Superspecific	1	Cyanobacteria*	C	84	91.14	26.62	98.11	0
		2	Unclassified	U	12	4.95	72.12	0	0
		0	Nobin	No	358	3.91	1.48	na	na
ULC027	Verysensitive	1	Cyanobacteria*	C	439	21.40	6.27	90.43	0.27
		2	Alphaproteobacteria*	M	190	16.16	7.71	95.02	1.16
		3	Sphingomonadales*	M	293	12.03	6.18	60.21	2.35
		4	Unclassified	U	164	4.16	5.09	4.17	0
		0	Nobin	No	24 364	46.24	1.89	na	na
ULC041	Verysensitive	1	Cyanobacteria*	C	287	84.76	31.38	96.2	1.63
		2	Unclassified	U	24	9.36	44.33	0	0
		0	Nobin	No	441	5.88	3.97	na	na
ULC065	Veryspecific	1	Cyanobacteria*	C	95	22.36	38.37	99.09	0.27
		2	Xanthomonadaceae*	M	332	19.33	6.19	83.73	1.23
		0	Nobin	No	20 555	58.31	1.73	na	na
ULC066	Superspecific	1	Cyanobacteria*	C	67	28.81	21.86	98.82	0.47
		2	Bacteroidetes*	M	401	13.94	4.93	76.91	1.23
		3	*Betaproteobacteria−*	M	152	2.86	3.48	15.86	0
		0	Nobin	No	24 558	54.38	1.69	na	na
ULC068	Superspecific	1	Cyanobacteria*	C	60	57.04	29.34	97.09	0.71
		2	Unclassified	U	3	2.56	22.60	0	0
		0	Nobin	No	10 385	40.41	1.42	na	na
ULC073	Verysensitive	1	Cyanobacteria*	C	476	22.70	10.74	92.03	1.42
		2	Betaproteobacteria*	M	65	16.26	7.99	97.92	0.67
		3	Sphingomonadales*	M	603	15.78	4.94	70.57	5.3
		4	*Bacteria−*	M	156	2.79	4.39	10.71	0
		5	Unclassified	U	26	1.40	15.02	0	0
		6	Unclassified	U	29	1.38	6.45	0	0
		0	Nobin	No	16 790	39.68	1.94	na	na
ULC077	Veryspecific	1	Cyanobacteria*	C	407	47.37	15.08	97.64	0.47
		0	Nobin	No	14 903	52.63	1.83	na	na
ULC082	Veryspecific	1	Cyanobacteria*	C	124	11.49	19.85	97.74	0.27
		2	Bacteria*	M	529	9.77	4.50	62.77	7.54
		3	Bacteria*	M	542	8.16	3.88	46.21	9.28
		4	*Bacteria*−	M	120	1.72	4.73	11.13	0
		5	Unclassified	U	74	1.67	4.57	0	0
		0	Nobin	No	30 077	67.18	2.15	na	na
ULC084	Superspecific	1	Betaproteobacteria*	M	232	23.15	5.67	93.61	1.73
		2	Alphaproteobacteria*	M	222	22.39	6.65	92.46	1.38
		3	Cyanobacteria*	C	116	21.88	20.78	98.55	0
		0	Nobin	No	10 835	32.58	1.59	na	na
ULC129	Verysensitive	1	Cyanobacteria*	C	299	38.35	18.46	98.64	0.77
		0	Nobin	No	21 968	61.65	1.62	na	na
ULC146	Superspecific	1	Burkholderiales*	M	177	16.18	10.96	96.57	0.93
		2	Flavobacteriaceae*	M	285	12.91	6.27	94.94	0.35
		3	Sphingomonadales*	M	74	11.54	14.23	88.9	1.39
		4	Betaproteobacteria*	M	98	10.85	7.64	97.46	1.09
		5	Alphaproteobacteria*	M	350	7.56	6.25	75.87	0.32
		6	*Bacteria*−	M	243	3.11	4.68	10.82	0
		7	Unclassified	U	21	1.86	12.53	8.33	0
		0	Nobin	No	28 569	35.99	1.72	na	na
ULC165	Verysensitive	1	Xanthomonadaceae*	M	53	15.37	24.76	99.54	0.8
		2	Alphaproteobacteria*	M	167	14.52	7.75	96.29	1.22
		3	Burkholderiales*	M	473	10.01	4.40	41.41	0.47
		4	Bacteria*	C	356	6.30	3.90	24.14	1.72
		0	Nobin	No	19 409	53.79	2.08	na	na
ULC179	Superspecific	1	Alphaproteobacteria*	M	247	18.89	16.30	98.54	60.19
		2	Rhizobiales*	M	261	16.95	8.86	94.78	0.94
		3	Alphaproteobacteria*	M	111	13.62	21.92	98.73	0.22
		4	Cytophagales*	M	718	13.40	4.60	67.06	0.3
		5	Alphaproteobacteria*	M	68	4.70	16.67	35.78	0
		6	Rhizobiales*	M	170	2.16	4.18	12.58	0
		7	Unclassified	U	16	1.69	41.33	0	0
		0	Nobin	No	13 101	28.59	1.94	na	na
ULC186	Verysensitive	1	Cyanobacteria*	C	412	67.38	21.10	93.18	1.64
		0	Nobin	No	6559	32.62	1.52	na	na
ULC187	Veryspecific	1	Cyanobacteria*	C	62	62.18	33.11	99.29	0.47
		0	Nobin	No	8482	37.82	1.43	na	na

Altogether, we identified 40 bins that were not of cyanobacterial origin out of our 17 cyanobacterial cultures. Among these foreign genome bins, we classified 21 as *Proteobacteria* and five as *Bacteroidetes*, and thus 26 bins contained organisms belonging to two bacterial phyla known to participate in the cyanobacterial microbiome [21, 50]. The remaining 14 bins could only be classified as *Bacteria* (five) or were left unclassified (nine) by CheckM. While unclassified bins were discarded from subsequent analyses, bins identified at the *Bacteria* level were retained. Genome completeness of these 31 bacterial bins was very heterogeneous (median=71.96 %, IQR=51.84 %). As for cyanobacterial bins, but more significantly, completeness correlated positively with sequencing coverage, lowly covered bins being the less complete (Pearson's *r*=0.46, *P*=0.007). Nevertheless, we managed to recover 13 nearly complete foreign bins (completeness ≥90 %). According to CheckM, the contamination level (foreign sequences not belonging to the taxonomic label of the bin under study) of the 26 classified non-cyanobacterial bins was always <9.28 % (median=0.8 %, IQR=1.13 %), except for ULC179-bin1 (60.19 %). The contamination level of the bins classified as *Bacteria* was not recorded, because such a high taxonomic rank made its evaluation meaningless. As for cyanobacterial bins, the number of scaffolds of the 31 bacterial bins remained quite high (>53, median=232, IQR=205). In spite of three cases of possible complementarity (in terms of recovered marker genes) suggested by CheckM (ULC027-bin3/ULC027-bin4, ULC146-bin3/ULC146-bin7 and ULC082-bin3/ULC082-bin4), the two first involving unclassified bins, the corresponding bins were not merged because CheckM phylogenetic placement was never congruent. Details about genome bins are available in Table S2. We released scaffolded assemblies and protein predictions for all the bins having a completeness ≥90 %, whether classified as cyanobacterial (14) or probable microbiome organisms (13).

### Cyanobacterial phylogenomics

**Fig. 1.**
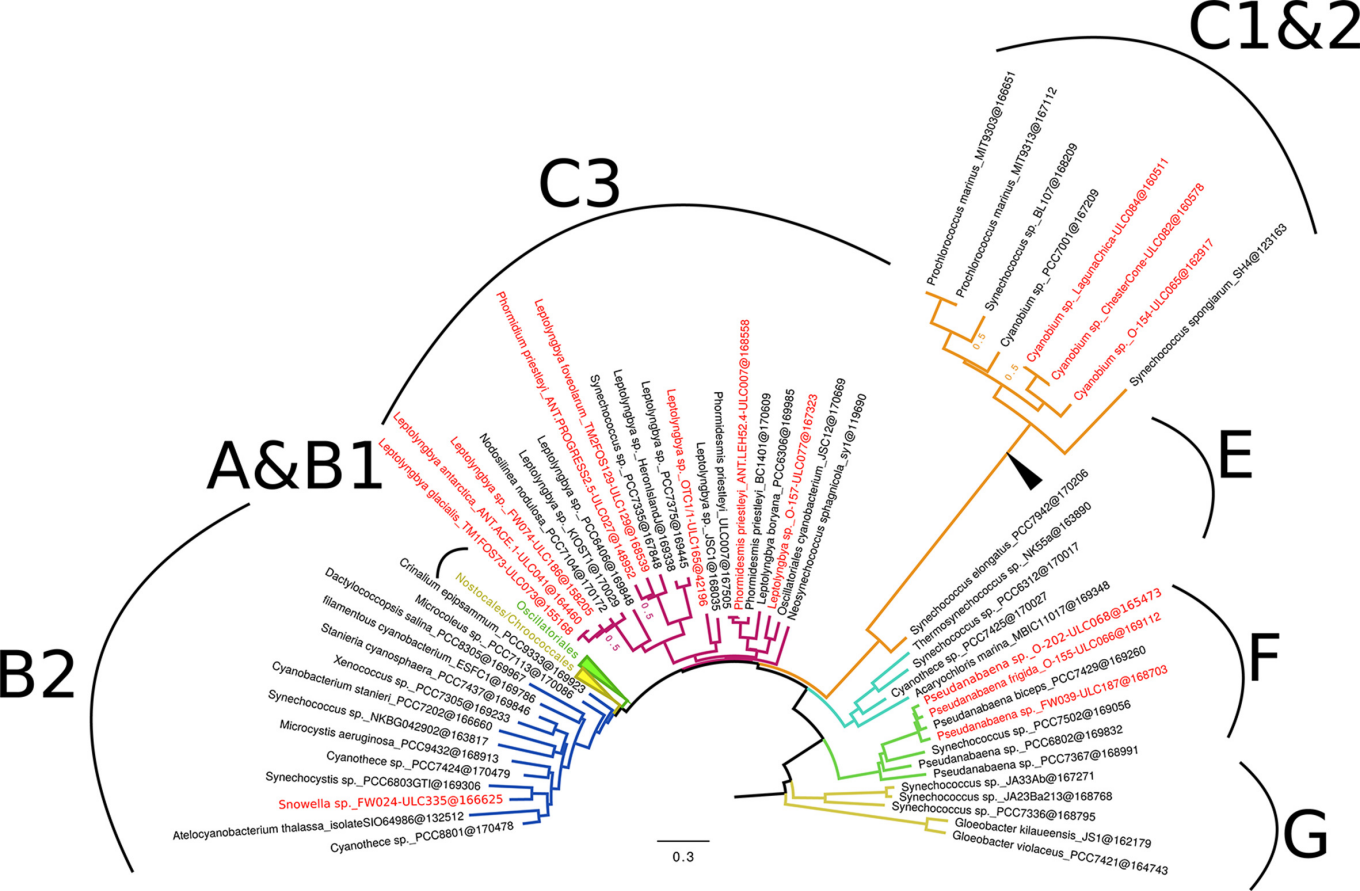
Phylogenomic tree of 64 broadly sampled *Cyanobacteria* showing the phylogenetic position of the 15 cyanobacterial genome bins. The Bayesian tree was inferred under the CAT+Γ_4_ model from a supermatrix made of 675 genes (79 organisms×170 983 amino-acid positions). Cyanobacterial clades (see Table 1) were named according to Shih *et al*. [52]. Trailing numbers in tip labels give the number of amino-acid positions effectively present in the corresponding concatenated sequence, whereas numbers at nodes are posterior probabilities (PP) computed from two independent chains (only PP values ≤1.0 are shown). Genome bins are shown in red. The location of the alternative root proposed by Tria *et al.* [70] is indicated by an arrowhead.

A phylogenomic analysis based on 675 genes and 64 reference *Cyanobacteria* showed that three cyanobacterial bins (i.e. excluding ULC335) were situated in the basal part of the cyanobacterial tree, here defined as clades G, F and E [51] (Fig. 1). Clade C, mainly composed of *Leptolyngbya* species and picoplanktonic *Cyanobacteria*, contains 11 cyanobacterial bins (Fig. 1). Statistical support [Bayesian posterior probability (PP)] was maximal except for three nodes. In the following, we refer to the cyanobacterial clades using the nomenclature defined by Shih *et al*. [52], since theirs was the first to fully sample the cyanobacterial morphological diversity (i.e. Sections I–V from [1]). Three ULC strains (*Pseudanabaena* sp. ULC187, *Pseudanabaena frigida* ULC066 and *Leptolyngbya* sp. ULC068) are located at a very basal (i.e. ‘early-branching’) position in clade F, and form a cluster with the reference strain *Pseudanabaena biceps* PCC 7429. Three other strains, identified as *Cyanobium* sp. (ULC065, ULC082 and ULC084), emerge together from the picocyanobacteria clade C1. Although their C1 membership is indisputable, the exact branching point within clade C1 is not resolved (PP=0.51). The six *Leptolyngbya* strains (*Leptolyngbya* sp. ULC077/ULC165/ULC186, *L. antarctica* ULC041, *L. glacialis* ULC073 and *L. foveolarum* ULC129) and the two *Phormidesmis*/*Phormidium priestleyi* strains (ULC007 and ULC027) are located in clade C3, mainly composed of reference *Leptolyngbya* strains. While two strains (*Leptolyngbya* sp. ULC077 and ULC165) each form an additional single branch within clade C3, five other strains emerge as two new sub-groups: *Leptolyngbya foveolarum* ULC129 and *Phormidium priestleyi* ULC027 on the one hand (yet weakly supported: PP=0.51), and *Leptolyngbya* sp. ULC186, *Leptolyngbya antarctica* ULC041 and *Leptolyngbya glacialis* ULC073 on the other. As expected, our new assembly of *Phormidesmis priestleyi* ULC007 is extremely close to the first release of the same genome (*Phormidesmis priestleyi* ULC007 GCF_001895925.1), which we used as positive control for our pipeline [53]. Finally, *Snowella* sp. ULC335 is part of clade B2, composed of various cyanobacterial genera from the orders *Pleurocapsales* and *Chroococales* [54]. This strain branches with *Synechocystis* sp. PCC 6803, which is among the most comprehensively studied *Cyanobacteria*, again with maximal support.

### Microbiome phylogenomics

**Fig. 2.**
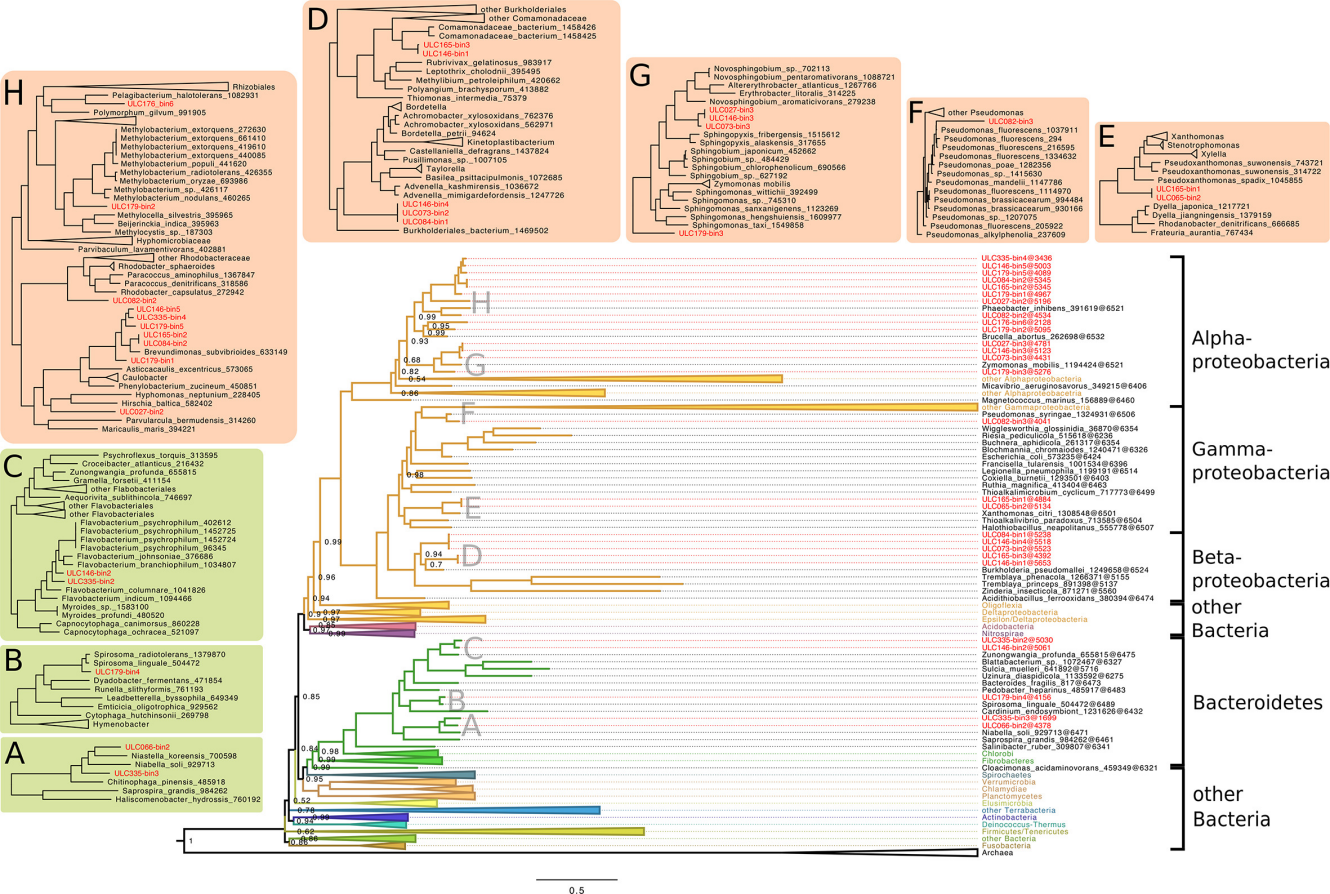
Phylogenomic tree of 196 broadly sampled *Bacteria* and *Archaea* showing the phylogenetic position of 27 microbiome genome bins. The Bayesian tree was inferred under the CAT+Γ_4_ model from a supermatrix made of 53 ribosomal genes (223 organisms×7060 amino-acid positions). PP values ≤1.0 are shown at the corresponding nodes. Surrounding subtrees are excerpts from a large maximum-likelihood tree inferred under the LG4X model from the full supermatrix (3501 organisms ×6613 amino-acid positions; Fig. S1). The 27 microbiome bins are indicated in red. *Bacteroidetes* bins are shown on a green background, whereas *Protebacteria* bins are shown on an orange background.

To identify the organisms in the putative microbiome bins recovered from the 17 cultures, we built two phylogenomic trees with different taxon samplings of reference prokaryotes from a concatenation of 53 ribosomal proteins (see Materials and Methods). Fig. 2 shows the small tree (193 *Bacteria* and 30 *Archaea*), surrounded by zooms in specific regions of the large tree (3374 *Bacteria* and 127 *Archaea*; Fig. S1). Only 27 out of 31 non-cyanobacterial bins could be included in the tree, four bins (marked by a dash in Table 3) being too incomplete to be positioned robustly (see Materials and Methods). The resolution of the small tree was quite good, with 78 % of the nodes having PP≥0.90 and no node having a PP<0.50. This analysis showed that all 27 analysed microbiome bins fall either in *Bacteroidetes* (five bins) or in *Proteobacteria* (14 bins in *Alphaproteobacteria*, five bins in *Betaproteobacteria* and three bins in *Gammaproteobacteria*) (Fig. 2), the tree allowing us to precisely determine the CheckM ‘bacterial’ affiliation of ULC082-bin3 to *Gammaproteobacteria*. In all cases, microbiome bins were sisters to one or more of the representative organisms with PP≥0.99, except for ULC179-bin3 (PP=0.63). Insets A–C of Fig. 2 demonstrate that the five *Bacteroidetes* bins correspond to different organisms, despite the fact that they appear closely clustered in the small tree. However, the picture is different for the bins falling in *Proteobacteria* (insets E–H). Whereas they are globally scattered across the phylum, there exist five cases (involving 11 bins) for which two or three bins from different cyanobacterial cultures appear extremely close in the large tree: ULC073-bin2/ULC084-bin1/ULC146-bin4 (D), ULC146-bin1/ULC165-bin3 (D), ULC065-bin2/ULC165-bin1 (E), ULC027-bin3/ULC146-bin3 (G) and ULC084-bin2/ULC165-bin2 (H). Taking this into account, the 27 microbiome bins only create 21 terminal branches in the large tree, five of them (representing six strains) clustering with a reference strain of *Brevundimonas subvibrioide*s (H).

### SSU rRNA (16S) analyses

In an attempt to refine the taxonomic analysis of all our genome bins, we predicted their SSU rRNA (16S) with RNAmmer [47]. Hence, we managed to predict 38 sequences (Table 4). Unfortunately, the vast majority (33) of the rRNA genes were predicted from unbinned metagenomic contigs (nobins; see Materials and Methods). When the taxon corresponding to the rRNA was straightforward to match with the taxon of one of the bins from the same cyanobacterial culture (based on congruent CheckM and sina classifications), we manually affiliated the rRNA gene to that bin. This was possible for 20 predicted rRNA genes, but 13 sequences could not be reliably affiliated to any genome bin (empty cells in Table 4). According to sina [48], only 10 of the predicted SSU rRNA genes were of cyanobacterial origin, whereas eight sequences were left unclassified. The 20 remaining sequences were of either *Proteobacteria* or *Bacteroidetes* origin, thereby confirming the results of our phylogenomic analysis of microbiome bins based on rRNA proteins. Two best hits were encountered more than once by sina: *Blastomonas* sp. AAP25 (from a Czech freshwater lake) in ULC073-bin6 and ULC146-bin3, and ‘Uncultured bacterium’ clone B3NR69D12 (from a drinking water biofilm) in ULC073-bin2 and ULC084-bin1.

**Table 4.** SSU rRNA (16S) gene prediction, taxonomy and coverage The last-common ancestor (LCA) classification and top hits were retrieved from sina analyses. The bins with SSU rRNA (16S) genes directly predicted from the genome bins (without manual assignment) are indicated by an asterisk (*). Coverage values were computed with BBMap. NA, not applicable.

Strain	SSUref_128 taxon	SSUref_128 top hit	Bin affiliation		Coverage
ULC335	*Snowella*	*Snowella litoralis* 1LT47S05	bin0	bin1	37.00
ULC335	*Brevundimonas*	Uncultured *Brevundimonas* sp.	bin0		22.23
ULC335	*Flavobacterium*	Uncultured bacterium clone N4_091	bin0	bin2	58.44
ULC335	Unclassified	na	bin0		10.25
ULC335	*Hydrogenophaga*	*Hydrogenophaga palleronii*	bin0		9.64
ULC335	*Rhodobacteraceae*	Uncultured bacterium clone ZWB3-3	bin0		7.79
ULC007	*Leptolyngbya*	*Phormidesmis priestleyi* ANT.LG2.4 16S	bin0	bin1	85.23
ULC027	Unclassified	na	bin2	bin2*	54.08
ULC041	*Leptolyngbya*	*Leptolyngbya antarctica* ANT.LACV6.1	bin0	bin1	97.23
ULC065	*Arenimonas*	Uncultured bacterium clone a33	bin0	bin2	40.66
ULC065	*Synechococcus*	*Cyanobium* sp. JJ17-5	bin0	bin1	165.13
ULC066	*Limnobacter*	Uncultured bacterium clone S25	bin0	bin3	14.15
ULC066	Unclassified	na	bin0		21.51
ULC066	FamilyI	*Pseudanabaena biceps* PCC 7429	bin0	bin1	50.06
ULC068	FamilyI	*Pseudanabaena* sp. Sai012	bin0	bin1	68.53
ULC073	*Sphingomonadaceae*	*Blastomonas* sp. AAP25	bin6	bin6*	31.87
ULC073	*Leptolyngbya*	*Leptolyngbya antarctica* ANT.LACV6.1	bin0	bin1	33.60
ULC073	*Limnobacter*	Uncultured bacterium clone B3NR69D12	bin0	bin2	19.58
ULC077	Unclassified	na	bin0	bin1	52.80
ULC082	*Hydrogenophaga*	Uncultured *Comamonadaceae* bacterium	bin0		18.85
ULC082	*Brevundimonas*	Uncultured alphaproteobacterium clone KWK6S.50	bin0		25.08
ULC082	Unclassified	na	bin0		32.77
ULC082	*Pseudomonas*	*Pseudomonas* sp. WCS374	bin0		32.71
ULC082	*Synechococcus*	*Synechococcus* sp. MW97C4	bin0	bin1	93.93
ULC084	*Brevundimonas*	Uncultured alphaproteobacterium	bin0	bin2	31.39
ULC084	*Synechococcus*	Uncultured bacterium clone MS81	bin0	bin3	87.30
ULC084	*Limnobacter*	Uncultured bacterium clone B3NR69D12	bin0	bin1	16.99
ULC129	*Phormidium*	Uncultured bacterium clone GBII-52	bin0	bin1	52.71
ULC146	*Sphingomonadaceae*	*Blastomonas* sp. AAP25	bin3	bin3*	81.39
ULC146	*Flavobacterium*	*Flavobacterium* sp. Leaf359	bin0	bin2	25.91
ULC146	*Hydrogenophaga*	*Hydrogenophaga* sp. Root209	bin1	bin1*	61.13
ULC165	Unclassified	na	bin0		85.20
ULC165	Unclassified	na	bin0		98.62
ULC179	*Devosia*	*Devosia psychrophila* strain Cr7-05	bin0		97.88
ULC179	Unclassified	na	bin0		16.43
ULC179	*Polymorphobacter*	Uncultured *Sphingomonadaceae* bacterium	bin3	bin3*	91.23
ULC186	FamilyI	*Leptolyngbya* sp. 0BB32S02	bin0	bin1	116.04
ULC187	FamilyI	*Pseudanabaena* sp. Sai010	bin0	bin1	81.01

## Discussion

According to the standards developed by the Genomic Standards Consortium for the minimum information about metagenomes of bacteria and archaea [25], the vast majority (14) of the cyanobacterial bins are of medium-quality, as their genome completeness is ≥90 % and their contamination level <5 % (both with CheckM and with DIAMOND blastx). Yet, they are still composed of a large number of scaffolds (≥60), due to the use of short insert DNA libraries for sequencing (Tables 3 and S2). In contrast, the only low-quality cyanobacterial assembly obtained here (ULC165-bin4) shows a completeness of 24.14 %, in agreement with the lowest coverage obtained over all four ULC165 bins (3.90 %). The situation is worse with the two *Nostocales* cultures (ULC146 and ULC179), for which we could not isolate any cyanobacterial bin. This lack of cyanobacterial contigs can be explained by the fact that these three strains (ULC146, ULC165 and ULC179) produce a thick polysaccharidic sheath that hinders DNA extraction [1]. Such a thick sheath is thought to protect the organisms from the harsh conditions of their hostile environment (Sør Rondane Mountains in Antarctica in all three cases). The use of a DNA extraction protocol more adapted to these organisms with a thick sheath (e.g. [55]) might have given different results and should be considered for future applications. Regardless, the recovery of only one cyanobacterium per sample provides molecular evidence for the integrity of the cultures in the BCCM/ULC collection.

When MetaBAT partitioned the metagenomic contigs, it produced nine small bins that were left unclassified by CheckM. In two cases, unclassified bins were identified as complementary (of CheckM marker genes) to another bin from the same metagenome (ULC027-bin3/ULC027-bin4; ULC146-bin3/ULC146-bin7; see above). Despite similar values in GC content and sequencing coverage, we did not merge these bins, thereby following the recommendations in the CheckM manual, because we had no indication about the phylogenetic affiliation of the unclassified bins. Because they only represented a very small fraction of the metagenomes, we discarded these bins from our phylogenetic analyses. Puzzlingly, such a bin was also recovered from strain ULC007, for which no foreign bin was expected due to its axenicity. While the sequencing coverage of the unclassified bin (ULC007-bin2) was more than twice that of the main bin (ULC007-bin1), tetranucleotide frequencies (TNFs) were undistinguishable between the two bins (Figs S1 and S3). This suggests that the corresponding contigs originate from the same organism but that the small bin contains contigs encoded in multiple copies in the genome. We attempted to characterize some unclassified bins from a functional point a view using Prodigal [33] and Blast2GO [56]. Unfortunately, the results were largely inconclusive and we could not ascertain whether these bins (containing some transferases, e.g. acyltransferases, transferring one-carbon groups, transferring nitrogenous groups) correspond to aberrant chromosomal regions (e.g. laterally transferred segments, repetitive elements) or to plasmids (data not shown).

Even if our assemblies are globally of medium quality, they often lack SSU rRNA (16S) genes. Hence, out of 38 predicted rRNA genes, as few as five were predicted from genome bins (all of which are foreign bins), leaving 50 bins without any rRNA gene. Apparently, rRNA genes are rejected by MetaBAT, because we could only predict them from unbinned contigs (nobins) in all remaining cases (33). Importantly, this outcome was independent of the parameter set used for MetaBAT (data not shown). We nonetheless elected to favour this software because its binning performance in terms of completeness is better than that of other recent tools, such as CONCOCT [57], GroopM [58], MaxBin [59] and Canopy [60] (see figure 3 of Kang *et al.* [27]). Whenever sina [48] successfully classified a predicted SSU rRNA (16S) gene, we did our best to manually affiliate it to the corresponding genome bin (Table 4). Consequently, 10 of our 15 cyanobacterial bins turned into high-quality genomes [25]. In this respect, it is worth mentioning that, among the 651 cyanobacterial genome assemblies available on the NCBI as of December 2017, only 458 have an SSU rRNA (16S) gene, based on RNAmmer [47] predictions (data not shown). According to our analyses, the frequent loss of rRNA genes is caused by the presence of multiple copies of the rRNA operon in many bacterial genomes [61], resulting in short rRNA-bearing contigs due to incomplete assembly of repeated regions. Because these contigs are dominated by the rRNA operon, they feature both a higher sequencing coverage and divergent TNFs, two properties that interfere with the binning process carried out by MetaBAT and other metagenomic software (Appendix S2). Yet, an improved sequencing depth might have positively impacted the results of our study. Even if sequencing coverage (ranging between 6.27 and 38.37) was sufficient to ensure reliable binning of the cyanobacterial contigs, deeper coverage would have resulted in more complete bins, whether cyanobacterial or corresponding to the microbiome bacteria. More data could also have improved assembly contiguity (in terms of scaffold size), which in turn might have helped with the binning of rRNA genes. This is particularly important because SSU rRNA (16S) is still the standard for microbial taxonomy [49]. Another way to improve the assembly quality is to use third-generation sequencing (TGS), such as Pacific Bioscience (PacBio) or Oxford Nanopore Technology (ONT). These approaches use long reads of 10 kb (instead of 250 nt with Illumina), which has been shown to increase the contiguity of assemblies, especially in bacteria [62, 63]. Regarding the exploitation of non-axenic cultures, it has been recently shown that plasmid binning from PacBio data could avoid the production of small unclassified bins by considering features others than TNF and coverage alone [64].

Our phylogenomic tree of *Cyanobacteria* is based on the largest supermatrix (in terms of conserved positions) to date (64 non-contaminated and complete reference strains; >170 000 unambiguously aligned amino-acid positions). It is congruent with other recent cyanobacterial phylogenies [52, 65]. We chose to root the tree on the *Gloeobacter* species (clade G), following the practice of many recent cyanobacterial phylogenies (e.g. [8, 40, 52, 65–68]). Nevertheless, it is worth mentioning that the basal position of *Gloeobacter* has been criticized [69] and that an alternative rooting has been recently proposed [70]. Interestingly, three of the cyanobacterial bins corresponding to polar or subpolar strains are clearly located in the basal part of the tree. The BCCM/ULC collection has a focus on (sub)polar cyanobacterial strains that may present interesting features to survive freeze/thaw cycles, seasonally contrasted light intensities, high UV radiation, desiccation and other stresses. Cyanobacterial diversity from such environments is presently underrepresented in comparison to that of marine *Cyanobacteria*. This is notably due to the difficulty of cultivating these organisms from ‘cold regions’, such as polar or alpine *Cyanobacteria* [13]. Hence, increasing the sampling of (cyano)bacteria from these environments may lead to a better understanding of their functional adaptation to environmental pressures, which is especially important in the context of climate change [13]. Moreover, the three ‘early-branching’ *Pseudanabaena* strains (ULC066, ULC068 and ULC187 in clade F) should prove useful to improve the resolution of the phylogeny of *Cyanobacteria* in further studies by increasing their taxon sampling. Two of these strains were isolated from Canadian samples and ULC066 even originates from the Arctic (Table 1).

When the sequencing coverage was sufficient, we also assembled the foreign (i.e. non-cyanobacterial) bins. According to Bowers *et al*. [25], 13 of these bins are of medium quality (completeness ≥90 %) and 18 bins are of low quality (completeness <90 %) (Table 3). All are either of *Proteobacteria* or *Bacteroidetes* origin, as assessed by both CheckM and phylogenomic inference. All the *Cyanobacteria* of the present study are freshwater organisms. Consequently, the cyanobacterial microbiome from other environments might be completely different. From our phylogenomic analysis, it appears that the 27 analysed bins represent 21 different terminal branches in the tree (Fig. 2). As 11 were indistinguishable (or very closely related) in spite of the use of 53 ribosomal proteins, we investigated whether they represented genuinely different samplings of highly similar associated organisms or were the result of cross-contamination during *Cyanobacteria* isolation/cultivation or DNA processing (Appendix S3). Altogether, genome-wide similarity measurements suggest that cross-contamination may not be involved, even if sampling sites were occasionally very distant (i.e. Arctic and Antarctic samples). Inset H of Fig. 2 shows a group of six foreign bins clustered around a reference strain of *Brevundimonas subvibrioides*. As this alphaproteobacterium frequently appears as a last common ancestor taxon in sina classifications of SSU rRNA (16S) sequences (Table 4), this indicates that *Brevundimonas* (or related taxa) is regularly present in ULC cultures and probably naturally associated with *Cyanobacteria*. More generally, the classification of all identifiable foreign bins as either *Proteobacteria* or *Bacteroidetes* suggests that the associated organisms come from the original environment and accompanied the *Cyanobacteria* through the isolation steps. Indeed, these two phyla are known to co-evolve with *Cyanobacteria* through complex trophic relations [21, 50]. We probably identified only these two phyla in our foreign bins because they are the most abundant [21], whereas other associated bacterial phyla (*Actinobacteria*, *Gemmatimonadetes*, *Planctomycetes*, *Verrucomicrobia*) have been described in the cyanobacterial microbiome [15–17, 21]. This result is completely in line with our recent analysis of the level of contamination in publicly available cyanobacterial genomes, in which foreign sequences were also mainly classified as *Proteobacteria* and *Bacteroidetes* [24]. In other words, the difficulty with purifying non-axenic cyanobacterial cultures, possibly combined with the accidental transfer of associated bacteria during the isolation process (or any subsequent step), is probably the main cause for genome contamination. This certainly highlights the importance of careful bioinformatic protocols for genome data processing. In this respect, we compared our new assembly of ULC007 to the previous release of the same strain, based on a HiSeq run in addition to the MiSeq run used here [53]. Interestingly, all CheckM values (completeness, contamination, strain heterogeneity) for ULC007-bin1 were slightly better than those obtained for our previously published assembly (completeness 98.11 vs 95.99, contamination 0 vs 1.18, strain heterogeneity 0 vs 100). As the latter had used more primary data and benefited from a thorough curation by hand, this indicates that the fully automated metagenomic pipeline of the present study is also applicable for axenic strains.

### Conclusion

In this work, we showed that a quite straightforward metagenomic protocol allows us to take advantage of non-axenic cyanobacterial cultures. Our pipeline yields medium-quality genomes with a high level of completeness (high sensitivity) for a very low level of contaminant sequences (high specificity), which could be very useful for phylogenomic analyses. In contrast, it has the disadvantage of regularly discarding multi-copy SSU rRNA (16S) genes during the binning of metagenomic contigs. We have shown that this loss is due to their higher sequencing coverage and divergent TNFs, which are especially detrimental for short contigs. The metagenomic pipeline reported here has nevertheless the advantage of facilitating the assembly of cyanobacterial genomes, as long as enough genomic DNA can be extracted from the strains. Our results further indicate that the microbiome of different cultures can sometimes contain associated bacteria that are very closely related, even when sampling sites are very distant. Finally, we have released 14 novel cyanobacterial assemblies, including 11 (sub)polar strains, and 13 assemblies of organisms belonging to their microbiome.

## Supplementary Data

Supplementary File 1
