# Supplementary material for: Metagenomic assembly of new (sub)polar Cyanobacteria and their associated microbiome from non-axenic cultures
Source: Microb Genom. 2018 Aug 23;4(9):e000212. doi: 10.1099/mgen.0.000212 (PMC6202449; doi:10.1099/mgen.0.000212)
Supplement: Supplementary File 1 [file mgen-4-212-s001.pdf]

Supplemental Material for

Metagenomic assembly of new (sub)arctic Cyanobacteria and their associated microbiome from non-axenic cultures

Luc Cornet[1,2], Amandine R. Bertrand[1,3], Marc Hanikenne[3], Emmanuelle J. Javaux[2], Annick Wilmotte[4], Denis Baurain[1]*

1 InBioS – PhytoSYSTEMS, Eukaryotic Phylogenomics, University of Liège, Liège, Belgium

2 UR Geology – Palaeobiogeology-Palaeobotany-Palaeopalynology, University of Liège, Liège, Belgium

3 InBioS – PhytoSYSTEMS, Functional Genomics and Plant Molecular Imaging, University of Liège, Liège, Belgium

4 InBioS – CIP, Centre for Protein Engineering, University of Liège, Liège, Belgium

* corresponding author

Figure S1: Phylogenomic tree of 3501 Bacteria and Archaea showing the phylogenetic position of 27 microbiome genome bins. The tree was inferred by maximum-likelihood under the LG4X model from a supermatrix of 6613 amino-acid positions.

[The figure is so large that it is at the end of this file as a gigantic single page.]

Table S1: Details and download links about reference proteomes. All details were extracted from NCBI metadata.

Table S2: Assembly statistics, taxonomy, completeness, contamination and coverage of genome bins. The taxonomic label (CheckM taxon), the genome completeness (CM compl.), the contamination level (CM contam. and CM str. het.) were computed with CheckM, whereas DBX columns (DBX Cyano., DBX contam., DBX unknown, DBX unclass.) were computed by our DIAMOND blastx parser. Sequencing coverage [Coverage (med) and Coverage (IQR)] was computed with BBMap, while other statistics were extracted from QUAST output. Genome bins used in phylogenetic inference are marked by an (*) and discarded bins by an (-).

Assembly	Bioproject	Taxid	Name	Date of publication
GCA_000484535.1	PRJNA162637	1183438	Gloeobacter kilaueensis JS1	2013/10/28
GCF_000011385.1	PRJNA58011	251221	Gloeobacter violaceus PCC 7421	2003/10/03
GCF_000013205.1	PRJNA224116	321327	Synechococcus sp. JA-3-3Ab	2006/02/06
GCF_000013225.1	PRJNA224116	321332	Synechococcus sp. JA-2-3B'a(2-13)	2006/02/06
GCF_000332275.1	PRJNA224116	195250	Synechococcus sp. PCC 7336	2013/01/22
GCF_000317065.1	PRJNA224116	82654	Pseudanabaena sp. PCC 7367	2012/12/05
GCF_000332215.1	PRJNA224116	927668	Pseudanabaena biceps PCC 7429	2013/01/18
GCF_000317085.1	PRJNA224116	1173263	Synechococcus sp. PCC 7502	2012/12/05
GCF_000332175.1	PRJNA224116	118173	Pseudanabaena sp. PCC 6802	2013/01/22
GCF_000018105.1	PRJNA224116	329726	Acaryochloris marina MBIC11017	2007/10/16
GCA_000022045.1	PRJNA28337	395961	Cyanothece sp. PCC 7425	2009/01/09
GCF_000505665.1	PRJNA224116	1394889	Thermosynechococcus sp. NK55a	2013/12/11
GCF_000316685.1	PRJNA224116	195253	Synechococcus sp. PCC 6312	2012/12/04
GCF_000775285.1	PRJNA224116	1497020	Neosynechococcus sphagnicola sy1	2014/09/29
GCF_000309945.1	PRJNA224116	864702	Oscillatoriales cyanobacterium JSC-12	2012/11/05
GCF_001895925.1	PRJNA224116	1920490	Phormidesmis priestleyi ULC007	2016/12/09
GCF_001650195.1	PRJNA224116	1850361	Phormidesmis priestleyi BC1401	2016/05/23
GCF_000353285.1	PRJNA224116	272134	Leptolyngbya boryana PCC 6306	2013/04/06
GCF_000733415.1	PRJNA224116	1487953	Leptolyngbya sp. JSC-1	2014/07/25
GCF_000332095.2	PRJNA224116	1173264	Leptolyngbya sp. PCC 6406	2014/01/15
GCF_000763385.1	PRJNA224116	1229172	Leptolyngbya sp. KIOST-1	2014/10/03
GCF_000309385.1	PRJNA224116	118166	Nodosilinea nodulosa PCC 7104	2012/11/05
GCF_000155595.1	PRJNA224116	91464	Synechococcus sp. PCC 7335	2008/08/15
GCF_000482245.1	PRJNA224116	1385935	Leptolyngbya sp. Heron Island J	2013/10/25
GCF_000316115.1	PRJNA224116	102129	Leptolyngbya sp. PCC 7375	2012/12/03
GCF_000464785.1	PRJNA224116	1255374	Planktothrix rubescens NIVA-CYA 407	2013/09/06
GCF_000175415.3	PRJNA224116	634502	Arthrospira platensis str. Paraca	2014/06/06
GCF_000478195.2	PRJNA224116	1348334	Lyngbya aestuarii BL J	2013/10/21
GCF_000332155.1	PRJNA224116	402777	Kamptonema formosum PCC 6407	2013/01/22
GCF_000317475.1	PRJNA224116	179408	Oscillatoria nigro-viridis PCC 7112	2012/12/06
GCF_000317105.1	PRJNA224116	56110	Oscillatoria acuminata PCC 6304	2012/12/05
GCF_000317515.1	PRJNA224116	1173027	Microcoleus sp. PCC 7113	2012/12/06
GCF_000021825.1	PRJNA224116	65393	Cyanothece sp. PCC 7424	2008/12/17
GCA_000307995.2	PRJEA88171	1160280	Microcystis aeruginosa PCC 9432	2012/08/06
GCF_000021805.1	PRJNA224116	41431	Cyanothece sp. PCC 8801	2008/12/17
GCF_000737945.1	PRJNA256120	1527444	Candidatus Atelocyanobacterium thalassa isolate SIO64986	2014/08/05
GCF_000284135.1	PRJNA224116	1080228	Synechocystis sp. PCC 6803 substr. GT-I	2011/12/01
GCF_000715475.1	PRJNA224116	490193	Synechococcus sp. NKBG042902	2014/06/27
GCF_000317655.1	PRJNA39697	292563	Cyanobacterium stanieri PCC 7202	2012/12/07
GCF_000332055.1	PRJNA224116	102125	Xenococcus sp. PCC 7305	2013/01/17
GCF_000317575.1	PRJNA224116	111780	Stanieria cyanosphaera PCC 7437	2012/12/06
GCF_000380225.1	PRJNA224116	1128427	filamentous cyanobacterium ESFC-1	2013/04/23
GCF_000317615.1	PRJNA224116	13035	Dactylococcopsis salina PCC 8305	2012/12/07
GCF_000317495.1	PRJNA224116	1173022	Crinalium epipsammum PCC 9333	2012/12/06
GCF_000317555.1	PRJNA224116	1173026	Gloeocapsa sp. PCC 7428	2012/12/06
GCF_000317125.1	PRJNA224116	251229	Chroococcidiopsis thermalis PCC 7203	2012/12/05
GCF_000582685.1	PRJNA224116	1469607	[Scytonema hofmanni] UTEX 2349	2014/02/27
GCF_000789435.1	PRJNA224116	1532906	Aphanizomenon flos-aquae 2012/KM1/D3	2014/12/04
GCF_000196515.1	PRJNA224116	551115	'Nostoc azollae' 0708	2010/06/14
GCF_000316645.1	PRJNA224116	28072	Nostoc sp. PCC 7524	2012/12/04
GCF_000204075.1	PRJNA10642	240292	Anabaena variabilis ATCC 29413	2005/09/15
GCA_000340565.3	PRJNA185469	313624	Nodularia spumigena CCY9414	2014/04/03
GCF_000020025.1	PRJNA224116	63737	Nostoc punctiforme PCC 73102	2008/04/24
GCF_000332295.1	PRJNA224116	643473	Fortiea contorta PCC 7126	2013/01/22
GCF_000346485.2	PRJNA224116	128403	Scytonema hofmannii PCC 7110	2016/03/16
GCF_000734895.2	PRJNA224116	1337936	Calothrix sp. 336/3	2015/05/06
GCF_000332255.1	PRJNA224116	1173021	cyanobacterium PCC 7702	2013/01/22
GCF_000317225.1	PRJNA224116	98439	Fischerella thermalis PCC 7521	2012/12/05
GCF_000012525.1	PRJNA224116	1140	Synechococcus elongatus PCC 7942	2005/11/08
GCF_000586015.1	PRJNA224116	1451353	Candidatus Synechococcus spongiarum SH4	2014/03/07
GCF_000155635.1	PRJNA224116	180281	Cyanobium sp. PCC 7001	2008/08/29
GCA_000015705.1	PRJNA13496	59922	Prochlorococcus marinus str. MIT 9303	2007/01/22
GCF_000011485.1	PRJNA224116	74547	Prochlorococcus marinus str. MIT 9313	2003/08/18
GCF_000153805.1	PRJNA224116	313625	Synechococcus sp. BL107	2006/10/23

Assembly	ftp link
GCA_000484535.1	ftp://ftp.ncbi.nlm.nih.gov/genomes/all/GCA/000/484/535/GCA_000484535.1_ASM48453v1
GCF_000011385.1	ftp://ftp.ncbi.nlm.nih.gov/genomes/all/GCF/000/011/385/GCF_000011385.1_ASM1138v1
GCF_000013205.1	ftp://ftp.ncbi.nlm.nih.gov/genomes/all/GCF/000/013/205/GCF_000013205.1_ASM1320v1
GCF_000013225.1	ftp://ftp.ncbi.nlm.nih.gov/genomes/all/GCF/000/013/225/GCF_000013225.1_ASM1322v1
GCF_000332275.1	ftp://ftp.ncbi.nlm.nih.gov/genomes/all/GCF/000/332/275/GCF_000332275.1_ASM33227v1
GCF_000317065.1	ftp://ftp.ncbi.nlm.nih.gov/genomes/all/GCF/000/317/065/GCF_000317065.1_ASM31706v1
GCF_000332215.1	ftp://ftp.ncbi.nlm.nih.gov/genomes/all/GCF/000/332/215/GCF_000332215.1_ASM33221v1
GCF_000317085.1	ftp://ftp.ncbi.nlm.nih.gov/genomes/all/GCF/000/317/085/GCF_000317085.1_ASM31708v1
GCF_000332175.1	ftp://ftp.ncbi.nlm.nih.gov/genomes/all/GCF/000/332/175/GCF_000332175.1_ASM33217v1
GCF_000018105.1	ftp://ftp.ncbi.nlm.nih.gov/genomes/all/GCF/000/018/105/GCF_000018105.1_ASM1810v1
GCA_000022045.1	ftp://ftp.ncbi.nlm.nih.gov/genomes/all/GCA/000/022/045/GCA_000022045.1_ASM2204v1
GCF_000505665.1	ftp://ftp.ncbi.nlm.nih.gov/genomes/all/GCF/000/505/665/GCF_000505665.1_ASM50566v1
GCF_000316685.1	ftp://ftp.ncbi.nlm.nih.gov/genomes/all/GCF/000/316/685/GCF_000316685.1_ASM31668v1
GCF_000775285.1	ftp://ftp.ncbi.nlm.nih.gov/genomes/all/GCF/000/775/285/GCF_000775285.1_ASM77528v1
GCF_000309945.1	ftp://ftp.ncbi.nlm.nih.gov/genomes/all/GCF/000/309/945/GCF_000309945.1_ASM30994v1
GCF_001895925.1	ftp://ftp.ncbi.nlm.nih.gov/genomes/all/GCF/001/895/925/GCF_001895925.1_ASM189592v1
GCF_001650195.1	ftp://ftp.ncbi.nlm.nih.gov/genomes/all/GCF/001/650/195/GCF_001650195.1_ASM165019v1
GCF_000353285.1	ftp://ftp.ncbi.nlm.nih.gov/genomes/all/GCF/000/353/285/GCF_000353285.1_ASM35328v1
GCF_000733415.1	ftp://ftp.ncbi.nlm.nih.gov/genomes/all/GCF/000/733/415/GCF_000733415.1_ASM73341v1
GCF_000332095.2	ftp://ftp.ncbi.nlm.nih.gov/genomes/all/GCF/000/332/095/GCF_000332095.2_ASM33209v2
GCF_000763385.1	ftp://ftp.ncbi.nlm.nih.gov/genomes/all/GCF/000/763/385/GCF_000763385.1_ASM76338v1
GCF_000309385.1	ftp://ftp.ncbi.nlm.nih.gov/genomes/all/GCF/000/309/385/GCF_000309385.1_ASM30938v1
GCF_000155595.1	ftp://ftp.ncbi.nlm.nih.gov/genomes/all/GCF/000/155/595/GCF_000155595.1_ASM15559v1
GCF_000482245.1	ftp://ftp.ncbi.nlm.nih.gov/genomes/all/GCF/000/482/245/GCF_000482245.1_LepHIscaffolds
GCF_000316115.1	ftp://ftp.ncbi.nlm.nih.gov/genomes/all/GCF/000/316/115/GCF_000316115.1_ASM31611v1
GCF_000464785.1	ftp://ftp.ncbi.nlm.nih.gov/genomes/all/GCF/000/464/785/GCF_000464785.1_NC407_1
GCF_000175415.3	ftp://ftp.ncbi.nlm.nih.gov/genomes/all/GCF/000/175/415/GCF_000175415.3_ASM17541v3
GCF_000478195.2	ftp://ftp.ncbi.nlm.nih.gov/genomes/all/GCF/000/478/195/GCF_000478195.2_ASM47819v2
GCF_000332155.1	ftp://ftp.ncbi.nlm.nih.gov/genomes/all/GCF/000/332/155/GCF_000332155.1_ASM33215v1
GCF_000317475.1	ftp://ftp.ncbi.nlm.nih.gov/genomes/all/GCF/000/317/475/GCF_000317475.1_ASM31747v1
GCF_000317105.1	ftp://ftp.ncbi.nlm.nih.gov/genomes/all/GCF/000/317/105/GCF_000317105.1_ASM31710v1
GCF_000317515.1	ftp://ftp.ncbi.nlm.nih.gov/genomes/all/GCF/000/317/515/GCF_000317515.1_ASM31751v1
GCF_000021825.1	ftp://ftp.ncbi.nlm.nih.gov/genomes/all/GCF/000/021/825/GCF_000021825.1_ASM2182v1
GCA_000307995.2	ftp://ftp.ncbi.nlm.nih.gov/genomes/all/GCA/000/307/995/GCA_000307995.2_ASM30799v2
GCF_000021805.1	ftp://ftp.ncbi.nlm.nih.gov/genomes/all/GCF/000/021/805/GCF_000021805.1_ASM2180v1
GCF_000737945.1	ftp://ftp.ncbi.nlm.nih.gov/genomes/all/GCF/000/737/945/GCF_000737945.1_ASM73794v1
GCF_000284135.1	ftp://ftp.ncbi.nlm.nih.gov/genomes/all/GCF/000/284/135/GCF_000284135.1_ASM28413v1
GCF_000715475.1	ftp://ftp.ncbi.nlm.nih.gov/genomes/all/GCF/000/715/475/GCF_000715475.1_ASM71547v1
GCF_000317655.1	ftp://ftp.ncbi.nlm.nih.gov/genomes/all/GCF/000/317/655/GCF_000317655.1_ASM31765v1
GCF_000332055.1	ftp://ftp.ncbi.nlm.nih.gov/genomes/all/GCF/000/332/055/GCF_000332055.1_ASM33205v1
GCF_000317575.1	ftp://ftp.ncbi.nlm.nih.gov/genomes/all/GCF/000/317/575/GCF_000317575.1_ASM31757v1
GCF_000380225.1	ftp://ftp.ncbi.nlm.nih.gov/genomes/all/GCF/000/380/225/GCF_000380225.1_ASM38022v1
GCF_000317615.1	ftp://ftp.ncbi.nlm.nih.gov/genomes/all/GCF/000/317/615/GCF_000317615.1_ASM31761v1
GCF_000317495.1	ftp://ftp.ncbi.nlm.nih.gov/genomes/all/GCF/000/317/495/GCF_000317495.1_ASM31749v1
GCF_000317555.1	ftp://ftp.ncbi.nlm.nih.gov/genomes/all/GCF/000/317/555/GCF_000317555.1_ASM31755v1
GCF_000317125.1	ftp://ftp.ncbi.nlm.nih.gov/genomes/all/GCF/000/317/125/GCF_000317125.1_ASM31712v1
GCF_000582685.1	ftp://ftp.ncbi.nlm.nih.gov/genomes/all/GCF/000/582/685/GCF_000582685.1_ASM58268v1
GCF_000789435.1	ftp://ftp.ncbi.nlm.nih.gov/genomes/all/GCF/000/789/435/GCF_000789435.1_ASM78943v1
GCF_000196515.1	ftp://ftp.ncbi.nlm.nih.gov/genomes/all/GCF/000/196/515/GCF_000196515.1_ASM19651v1
GCF_000316645.1	ftp://ftp.ncbi.nlm.nih.gov/genomes/all/GCF/000/316/645/GCF_000316645.1_ASM31664v1
GCF_000204075.1	ftp://ftp.ncbi.nlm.nih.gov/genomes/all/GCF/000/204/075/GCF_000204075.1_ASM20407v1
GCA_000340565.3	ftp://ftp.ncbi.nlm.nih.gov/genomes/all/GCA/000/340/565/GCA_000340565.3_ASM34056v3
GCF_000020025.1	ftp://ftp.ncbi.nlm.nih.gov/genomes/all/GCF/000/020/025/GCF_000020025.1_ASM2002v1
GCF_000332295.1	ftp://ftp.ncbi.nlm.nih.gov/genomes/all/GCF/000/332/295/GCF_000332295.1_ASM33229v1
GCF_000346485.2	ftp://ftp.ncbi.nlm.nih.gov/genomes/all/GCF/000/346/485/GCF_000346485.2_ASM34648v2
GCF_000734895.2	ftp://ftp.ncbi.nlm.nih.gov/genomes/all/GCF/000/734/895/GCF_000734895.2_ASM73489v2
GCF_000332255.1	ftp://ftp.ncbi.nlm.nih.gov/genomes/all/GCF/000/332/255/GCF_000332255.1_ASM33225v1
GCF_000317225.1	ftp://ftp.ncbi.nlm.nih.gov/genomes/all/GCF/000/317/225/GCF_000317225.1_FisPCC7521_1.0
GCF_000012525.1	ftp://ftp.ncbi.nlm.nih.gov/genomes/all/GCF/000/012/525/GCF_000012525.1_ASM1252v1
GCF_000586015.1	ftp://ftp.ncbi.nlm.nih.gov/genomes/all/GCF/000/586/015/GCF_000586015.1_SynSpo.0
GCF_000155635.1	ftp://ftp.ncbi.nlm.nih.gov/genomes/all/GCF/000/155/635/GCF_000155635.1_ASM15563v1
GCA_000015705.1	ftp://ftp.ncbi.nlm.nih.gov/genomes/all/GCA/000/015/705/GCA_000015705.1_ASM1570v1
GCF_000011485.1	ftp://ftp.ncbi.nlm.nih.gov/genomes/all/GCF/000/011/485/GCF_000011485.1_ASM1148v1
GCF_000153805.1	ftp://ftp.ncbi.nlm.nih.gov/genomes/all/GCF/000/153/805/GCF_000153805.1_ASM15380v1

Strain	MetaBAT setting	Bin	CheckM taxon	#Scaffolds	#Contigs	#C. (>1000 nt)	Length (nt)	Length (%)	L. (>1000 nt)	GC (%)	L50	N75 (nt)
ULC335	veryspecific	1	Cyanobacteria*	238	306	294	4602507	20.84	4595893	40.74	40.75	24097
		2	Flavobacteriaceae*	67	114	109	3031816	13.73	3027871	32.72	32.74	54021
		3	Bacteroidetes*	576	1335	938	2834244	12.83	2592321	45.95	45.96	3591
		4	Alphaproteobacteria*	271	645	387	1056769	4.79	905578	67.05	67.06	3052
		0	nobin	23056	24863	1517	10558473	47.81	2338854	61.30	52.35	2306
ULC007	superspecific	1	Cyanobacteria*	84	106	106	5223811	91.14	5223491	48.62	48.63	72864
		2	Unclassified	12	17	16	283907	4.95	282902	48.48	48.54	22102
		0	nobin	358	358	49	224030	3.91	157489	48.69	48.51	6599
ULC027	verysensitive	1	Cyanobacteria*	439	1018	870	4766714	21.40	4673662	50.35	50.4	7298
		2	Alphaproteobacteria*	190	553	471	3599900	16.16	3549598	63.22	63.25	11716
		3	Sphingomonadales*	293	782	599	2678920	12.03	2571356	62.95	62.97	5694
		4	Unclassified	164	414	280	927349	4.16	850288	65.33	65.69	4292
		0	nobin	24364	26287	1032	10298357	46.24	1737206	60.46	52.73	2732
ULC041	verysensitive	1	Cyanobacteria*	287	319	313	4697657	84.76	4694951	58.23	58.23	21370
		2	Unclassified	24	24	24	518635	9.36	518635	51.50	51.5	33120
		0	nobin	441	444	68	325698	5.88	256390	54.55	53.23	8350
ULC065	veryspecific	1	Cyanobacteria*	95	119	117	3082321	22.36	3080895	66.03	66.03	38827
		2	Xanthomonadaceae*	332	767	601	2664261	19.33	2565831	67.81	67.84	5855
		0	nobin	20555	21891	649	8036576	58.31	1004543	63.22	62.66	2548
ULC066	superspecific	1	Cyanobacteria*	67	86	86	5380362	28.81	5379882	41.84	41.84	99258
		2	Bacteroidetes*	401	904	670	2603362	13.94	2457932	39.33	39.53	4764
		3	Betaproteobacteria-	152	367	219	534032	2.86	442359	52.02	52.15	2784
		0	nobin	24558	26258	1225	10154658	54.38	1892140	58.57	48.27	2394
ULC068	superspecific	1	Cyanobacteria*	60	84	83	4551587	57.04	4550868	42.22	42.23	80830
		2	Unclassified	3	4	4	203969	2.56	203969	42.75	42.75	61745
		0	nobin	10385	10655	40	3224460	40.41	165266	62.14	43.04	103751
ULC073	verysensitive	1	Cyanobacteria*	476	619	572	4603354	22.70	4578777	57.76	57.74	10574
		2	Betaproteobacteria*	65	237	224	3297508	16.26	3287197	52.41	52.44	25326
		3	Sphingomonadales*	603	1581	1009	3199566	15.78	2861160	63.66	63.78	3639
		4	Bacteria-	156	372	200	566613	2.79	462681	63.90	64.34	2882
		5	Unclassified	26	27	27	283727	1.40	283717	49.79	49.79	13765
		6	Unclassified	29	70	58	279658	1.38	273663	57.84	57.96	6091
		0	nobin	16790	18486	1325	8046800	39.68	2063350	62.84	58.35	2376
ULC077	veryspecific	1	Cyanobacteria*	407	451	446	5440088	47.37	5436761	48.17	48.17	17549
		0	nobin	14903	16237	658	6044297	52.63	1134577	56.66	50.09	3489
ULC082	veryspecific	1	Cyanobacteria*	124	148	145	2790347	11.49	2788897	65.29	65.31	37235
		2	Bacteria*	529	1318	842	2371940	9.77	2087863	63.55	63.6	3235
		3	Bacteria*	542	1266	756	1981145	8.16	1666752	59.58	59.71	2939
		4	Bacteria-	120	260	158	417999	1.72	357553	66.70	66.9	2961
		5	Unclassified	74	172	125	405790	1.67	377521	53.86	53.85	3737
		0	nobin	30077	33680	3169	16310948	67.18	4763602	63.05	59.77	2314
ULC084	superspecific	1	Betaproteobacteria*	232	718	585	2976717	23.15	2892002	52.52	52.63	6715
		2	Alphaproteobacteria*	222	573	482	2878549	22.39	2826872	64.33	64.34	8706
		3	Cyanobacteria*	116	139	129	2813103	21.88	2809623	65.45	65.46	33348
		0	nobin	10835	11315	276	4188263	32.58	775675	59.91	53.48	6615
ULC129	verysensitive	1	Cyanobacteria*	299	317	316	4724332	38.35	4723951	51.02	51.02	20681
		0	nobin	21968	22744	305	7596258	61.65	588929	53.45	49.42	4773
ULC146	superspecific	1	Burkholderiales*	177	347	319	4889081	16.18	4871892	64.90	64.91	24186
		2	Flavobacteriaceae*	285	679	592	3900875	12.91	3842453	37.74	37.87	8727
		3	Sphingomonadales*	74	133	125	3487715	11.54	3483390	63.94	63.95	43449
		4	Betaproteobacteria*	98	289	258	3278028	10.85	3257798	52.43	52.46	19528
		5	Alphaproteobacteria*	350	771	582	2282825	7.56	2172115	66.42	66.53	5042
		6	Bacteria-	243	550	336	940367	3.11	810232	61.75	61.77	3017
		7	Unclassified	21	30	29	561075	1.86	560413	65.64	65.64	29757
		0	nobin	28569	29987	976	10872327	35.99	1638657	57.78	56.11	2467
ULC165	verysensitive	1	Xanthomonadaceae*	53	72	66	3099833	15.37	3097278	68.28	68.28	83934
		2	Alphaproteobacteria*	167	409	367	2927747	14.52	2903874	64.53	64.56	11367
		3	Burkholderiales*	473	1056	681	2018813	10.01	1786411	63.70	63.8	3287
		4	Bacteria*	356	674	471	1270968	6.30	1141258	47.34	47.65	3006
		0	nobin	19409	21418	2321	10846889	53.79	3733515	57.99	53.8	2416
ULC179	superspecific	1	Alphaproteobacteria*	247	359	344	5293532	18.89	5284967	66.86	66.87	23221
		2	Rhizobiales*	261	671	577	4749544	16.95	4692884	65.61	65.66	12479
		3	Alphaproteobacteria*	111	162	154	3817570	13.62	3813929	67.71	67.72	39185
		4	Cytophagales*	718	1658	1180	3753883	13.40	3454758	51.71	51.91	3772
		5	Alphaproteobacteria*	68	95	89	1318133	4.70	1314626	64.79	64.79	24810
		6	Rhizobiales*	170	391	231	604053	2.16	504692	61.84	62.25	2997
		7	Unclassified	16	22	22	473840	1.69	473830	61.96	61.96	30325
		0	nobin	13101	14683	1888	8010556	28.59	3418733	57.66	54.68	3032
ULC186	verysensitive	1	Cyanobacteria*	412	441	433	5062396	67.38	5057101	57.41	57.41	16589
		0	nobin	6559	6646	190	2450340	32.62	628243	50.93	53.77	9233
ULC187	veryspecific	1	Cyanobacteria*	62	71	71	4461408	62.18	4461288	43.15	43.15	115626
		0	nobin	8482	8566	40	2713800	37.82	234659	59.70	43.51	40647

Strain	MetaBAT setting	Bin	CheckM taxon	Coverage (med)	Coverage (IQR)	CM compl.	CM contam.	CM str. het.	DBX Cyano.	DBX contam.
ULC335	veryspecific	1	Cyanobacteria*	10.90	1.02	98.91	0.51	0	57.00	1.26
		2	Flavobacteriaceae*	11.12	1.37	99.29	0.12	0	0.06	69.60
		3	Bacteroidetes*	4.46	0.68	65.45	0.49	0	0.23	44.02
		4	Alphaproteobacteria*	4.13	0.75	32.28	0	0	0.14	58.32
		0	nobin	1.88	1.10	NA	NA	NA	0.63	46.97
ULC007	superspecific	1	Cyanobacteria*	26.62	1.26	98.11	0	0	58.12	0.71
		2	Unclassified	72.12	4.38	0	0	0	42.82	3.06
		0	nobin	1.48	24.50	NA	NA	NA	31.18	2.51
ULC027	verysensitive	1	Cyanobacteria*	6.27	1.24	90.43	0.27	0	52.26	1.02
		2	Alphaproteobacteria*	7.71	1.37	95.02	1.16	0	0.36	36.58
		3	Sphingomonadales*	6.18	1.20	60.21	2.35	7.14	0.13	58.84
		4	Unclassified	5.09	0.99	4.17	0	0	0.00	58.47
		0	nobin	1.89	1.11	NA	NA	NA	2.61	47.76
ULC041	verysensitive	1	Cyanobacteria*	31.38	7.36	96.2	1.63	22.22	48.68	1.30
		2	Unclassified	44.33	13.47	0	0	0	38.64	2.73
		0	nobin	3.97	31.72	NA	NA	NA	26.86	1.86
ULC065	veryspecific	1	Cyanobacteria*	38.37	10.53	99.09	0.27	0	54.48	1.34
		2	Xanthomonadaceae*	6.19	1.32	83.73	1.23	0	0.06	68.62
		0	nobin	1.73	0.88	NA	NA	NA	0.27	49.47
ULC066	superspecific	1	Cyanobacteria*	21.86	2.26	98.82	0.47	50	64.79	0.97
		2	Bacteroidetes*	4.93	0.75	76.91	1.23	0	0.32	53.00
		3	Betaproteobacteria-	3.48	0.54	15.86	0	0	0.00	74.50
		0	nobin	1.69	0.90	NA	NA	NA	0.33	42.49
ULC068	superspecific	1	Cyanobacteria*	29.34	0.80	97.09	0.71	0	64.09	1.37
		2	Unclassified	22.60	1.32	0	0	0	54.52	2.69
		0	nobin	1.42	0.62	NA	NA	NA	1.67	42.78
ULC073	verysensitive	1	Cyanobacteria*	10.74	1.99	92.03	1.42	12.5	48.29	1.45
		2	Betaproteobacteria*	7.99	0.54	97.92	0.67	0	0.02	73.70
		3	Sphingomonadales*	4.94	1.21	70.57	5.3	5.41	0.05	60.55
		4	Bacteria-	4.39	1.59	10.71	0	0	0.00	69.36
		5	Unclassified	15.02	2.12	0	0	0	39.22	4.96
		6	Unclassified	6.45	1.52	0	0	0	0.62	46.64
		0	nobin	1.94	1.37	NA	NA	NA	1.80	51.59
ULC077	veryspecific	1	Cyanobacteria*	15.08	2.30	97.64	0.47	0	57.21	1.00
		0	nobin	1.83	1.16	NA	NA	NA	3.62	44.61
ULC082	veryspecific	1	Cyanobacteria*	19.85	5.24	97.74	0.27	50	57.40	1.84
		2	Bacteria*	4.50	1.00	62.77	7.54	4.76	0.04	58.67
		3	Bacteria*	3.88	0.70	46.21	9.28	0	0.00	80.26
		4	Bacteria-	4.73	1.44	11.13	0	0	0.12	63.46
		5	Unclassified	4.57	0.64	0	0	0	0.00	70.78
		0	nobin	2.15	1.32	NA	NA	NA	0.28	55.82
ULC084	superspecific	1	Betaproteobacteria*	5.67	0.69	93.61	1.73	0	0.06	74.26
		2	Alphaproteobacteria*	6.65	1.23	92.46	1.38	0	0.03	67.26
		3	Cyanobacteria*	20.78	5.58	98.55	0	0	57.26	2.63
		0	nobin	1.59	0.74	NA	NA	NA	1.33	43.66
ULC129	verysensitive	1	Cyanobacteria*	18.46	2.64	98.64	0.77	25	53.27	1.03
		0	nobin	1.62	0.74	NA	NA	NA	1.83	36.96
ULC146	superspecific	1	Burkholderiales*	10.96	1.97	96.57	0.93	0	0.05	68.54
		2	Flavobacteriaceae*	6.27	0.88	94.94	0.35	0	0.04	65.50
		3	Sphingomonadales*	14.23	1.93	88.9	1.39	14.29	0.10	60.31
		4	Betaproteobacteria*	7.64	0.72	97.46	1.09	0	0.03	73.74
		5	Alphaproteobacteria*	6.25	1.48	75.87	0.32	0	0.04	63.80
		6	Bacteria-	4.68	1.62	10.82	0	0	0.13	63.90
		7	Unclassified	12.53	1.26	8.33	0	0	0.04	60.31
		0	nobin	1.72	0.93	NA	NA	NA	8.43	42.17
ULC165	verysensitive	1	Xanthomonadaceae*	24.76	4.65	99.54	0.8	0	0.09	70.37
		2	Alphaproteobacteria*	7.75	1.58	96.29	1.22	16.67	0.04	68.60
		3	Burkholderiales*	4.40	1.05	41.41	0.47	0	0.04	66.06
		4	Bacteria*	3.90	0.77	24.14	1.72	0	50.56	1.23
		0	nobin	2.08	1.58	NA	NA	NA	15.26	29.20
ULC179	superspecific	1	Alphaproteobacteria*	16.30	5.72	98.54	60.19	22.41	0.05	74.90
		2	Rhizobiales*	8.86	2.08	94.78	0.94	0	0.00	65.89
		3	Alphaproteobacteria*	21.92	5.67	98.73	0.22	0	0.10	48.99
		4	Cytophagales*	4.60	0.77	67.06	0.3	0	0.11	68.27
		5	Alphaproteobacteria*	16.67	6.03	35.78	0	0	0.00	63.92
		6	Rhizobiales*	4.18	1.28	12.58	0	0	0.00	67.76
		7	Unclassified	41.33	11.73	0	0	0	1.26	38.69
		0	nobin	1.94	1.77	NA	NA	NA	5.12	48.73
ULC186	verysensitive	1	Cyanobacteria*	21.10	4.81	93.18	1.64	9.09	47.80	1.49
		0	nobin	1.52	0.76	NA	NA	NA	8.95	37.47
ULC187	veryspecific	1	Cyanobacteria*	33.11	1.39	99.29	0.47	0	62.11	0.77
		0	nobin	1.43	0.60	NA	NA	NA	2.98	41.52

Supplemental Appendix S1 / Stepwise tutorial for installing and running the metagenomic pipeline

Cornet et al. (2018) Metagenomic assembly of non-axenic Cyanobacteria

v0.0.1 / July 6th, 2018

Contents

1 Installation **1**

1.1 Trimmomatic 1

1.2 SPAdes 2

1.3 BamM 2

1.4 MetaBAT 2

1.5 CheckM 2

1.6 DIAMOND blastx 2

1.7 DIAMOND blastx companion parser 2

1.8 SSPACE 3

2 Metagenomic pipeline **3**

2.1 Trimming of the Illumina reads 3

2.2 Metagenome assembly 3

2.3 Binning process 3

2.3.1 Copy SPAdes assembly 3

2.3.2 Make coverage files 3

2.3.3 Run MetaBat at different thresholds 3

2.4 Evaluation of binning accuracy 4

2.4.1 Run CheckM to select MetaBat run 4

2.4.2 Run DIAMOND blastx and companion parser 4

2.5 Scaffolding 5

1 Installation

The first step of the pipeline is to install the different tools used in the study.

1.1 Trimmomatic

```
$ wget http://www.usadellab.org/cms/uploads/supplementary/Trimmomatic/Trimmomatic-0.38.zip .
$ unzip Trimmomatic-0.38.zip
```

1.2 SPAdes

Follow the instructions for SPAdes installation:
http://cab.spbu.ru/files/release3.12.0/manual.html

```
$ wget http://cab.spbu.ru/files/release3.12.0/SPAdes-3.12.0-Linux.tar.gz
$ tar -xzf SPAdes-3.12.0-Linux.tar.gz
$ cd SPAdes-3.12.0-Linux/bin/
```

1.3 BamM

Follow the instructions for BamM installation:
http://ecogenomics.github.io/BamM/

```
$ sudo apt-get -y install git automake build-essential zlib1g-dev python2.7-numpy libtool
$ git clone https://github.com/Ecogenomics/BamM.git
$ cd BamM/
$ sudo python setup.py install
```

1.4 MetaBAT

Follow the instructions for MetaBat installation:
https://bitbucket.org/berkeleylab/metabat/overview

```
$ wget https://bitbucket.org/berkeleylab/metabat/get/master.tar.gz .
$ tar xzvf master.tar.gz
$ sudo apt-get update
$ sudo apt-get install -y build-essential libboost-all-dev scons git curl libncurses5-dev zlib1g-dev
$ cd berkeleylab-metabat-*
$ scons PREFIX=$HOME/metabat install
```

1.5 CheckM

Follow the instructions for CheckM installation:
https://github.com/Ecogenomics/CheckM/wiki/Installation#how-to-install-checkm

```
$ sudo pip install numpy
$ sudo pip install checkm-genome
```

1.6 DIAMOND blastx

Follow the instructions for DIAMOND blastx installation:
https://github.com/bbuchfink/diamond

```
$ wget http://github.com/bbuchfink/diamond/releases/download/v0.9.22/diamond-linux64.tar.gz
$ tar xzf diamond-linux64.tar.gz
```

1.7 DIAMOND blastx companion parser

```
$ cpanm Bio::MUST::Core
# create a local version of NCBI taxonomy
```

```
$ setup-taxdir.pl --taxdir=may-2018
# download parsing script (and unzip) from:
# https://doi.org/10.6084/m9.figshare.5006039
```

1.8 SSPACE

Follow the instructions for SSPACE2 installation:
https://github.com/nsoranzo/sspace_basic

Note: if wanted SSPACE3 can be obtained from BaseClear.

2 Metagenomic pipeline

2.1 Trimming of the Illumina reads

```
$ java -jar /PATH-TO/trimmomatic-0.32.jar PE \
$   -threads NUMBER-OF-THREADS \
$   ulc027-R1_prinseq.fastq ulc027-R2_prinseq.fastq \
$   ulc027-R1.paired.fastq ulc027-R1.unpaired.fastq \
$   ulc027-R2.paired.fastq ulc027-R2.unpaired.fastq \
$   ILLUMINACLIP:/PATH-TO/NexteraPE-PE.fa:2:30:20 \
$   LEADING:20 TRAILING:20 SLIDINGWINDOW:10:20 CROP:145 \
$   MINLEN:80
```

2.2 Metagenome assembly

```
$ /PATH-TO/spades.py --pe1-1 `ls ulc027-R1.paired.fastq` \
$   --pe1-2 `ls ulc027-R2.paired.fastq` \
$   --meta -t NUMBER-OF-THREADS -m MEMORY RAM -o ulc027-meta
```

2.3 Binning process

2.3.1 Copy SPAdes assembly

```
$ cp ulc027-meta/scaffolds.fasta .
$ mv scaffolds.fasta ulc027.fasta
```

2.3.2 Make coverage files

```
$ bamm make -d ulc027.fasta -i ulc027-R1.paired.fastq ulc027-R2.paired.fastq -t NUMBER-OF-THREADS
```

2.3.3 Run MetaBat at different thresholds

```
$ runMetaBat.sh --superspecific -t NUMBER-OF-THREADS ulc027.fasta \
    ulc027.ulc027-R1.pairedstq.bam ulc027.ulc027-R2.pairedstq.bam
$ runMetaBat.sh --specific -t NUMBER-OF-THREADS ulc027.fasta \
    ulc027.ulc027-R1.pairedstq.bam ulc027.ulc027-R2.pairedstq.bam
$ runMetaBat.sh --sensitive -t NUMBER-OF-THREADS ulc027.fasta \
```

```
    ulc027.ulc027-R1.pairedstq.bam ulc027.ulc027-R2.pairedstq.bam
$ runMetaBat.sh --verysensitive -t NUMBER-OF-THREADS ulc027.fasta \
    ulc027.ulc027-R1.pairedstq.bam ulc027.ulc027-R2.pairedstq.bam
$ runMetaBat.sh --veryspecific -t  NUMBER-OF-THREADS ulc027.fasta \
    ulc027.ulc027-R1.pairedstq.bam ulc027.ulc027-R2.pairedstq.bam
```

2.4 Evaluation of binning accuracy

2.4.1 Run CheckM to select MetaBat run

```
$ mkdir bins
$ mv *.fa bins

# fastest way
$ checkm unique ./bins -x fa

# careful way
$ checkm merge bacteria.ms ./bins ./output -x fa -t NUMBER-OF-THREADS
$ checkm tree ./bins ./out -x fa -t NUMBER-OF-THREADS
$ checkm tree_qa ./out
$ checkm lineage_set ./out marker.file
$ checkm analyze marker.file ./bins ./out -x fa -t NUMBER-OF-THREADS
$ checkm qa marker.file ./out -t NUMBER-OF-THREADS
```

2.4.2 Run DIAMOND blastx and companion parser

2.4.2.1 Download curated protein database derived from Ensembl Bacteria 30

```
# download script from: https://doi.org/10.6084/m9.figshare.5006039
$ unzip download-scripts.zip
$ cd download-scripts
$ tail -n 27763 curated-ensembl-30.sh > 2down.sh
$ bash 2down.sh
$ gunzip *.gz
```

2.4.2.2 Make DIAMOND database

```
$ cat *.fa > all-file.fa
$ diamond makedb --in all-file.fa -d DBNAME -p NUMBER-OF-THREADS
```

2.4.2.3 Run DIAMOND blastx on MetaBat bins

```
$ fasta2ali.pl *bins*.fa
$ chmod a+x split-ali.pl
$ for f in *bins*.ali; do split-ali.pl $f $f.sreads 250 250; done
$ ali2fasta.pl *.sreads
$ find *sreads.fasta > fasta.list
$ perl -i -nle 's/\.fasta//g; print' fasta.list
$ for f in `cat fasta.list`; do mkdir $f; done
$ for f in `cat fasta.list`; do diamond blastx -d /PATH-TO-DBNAME \
```

```
    -q $f.fasta -o $f.dblastx -k 100 -e 1e-3 -f tab -p NUMBER-OF-THREADS -t $f; done
```

2.4.2.4 Parse DIAMOND blastx output

```
$ chmod a+x blastx-parser.pl
$ ./blastx-taxonomic-parsing.pl *.dblastx --min-hit=1 --contam-list=NO \
    --blast-extension=-sreads.dblastx 2> logt
```

2.5 Scaffolding

Having selected the MetaBAT run and identified the bins of interest, scaffolding can be carried out.

```
$ echo PE bowtie ulc027-R1.paired.fastq ulc027-R2.paired.fastq 550 0.9 FR > libraries.txt
# 550 = insertsize / 0.9 = errorinsertsize
$ SSPACE.pl -l libraries.txt -s ulc027.fasta.*verysensitive_-t*.fa \
    -x 1 -m 32 -k 3 -n 15 -p 1 -b sspace
```

Supplemental Appendix S2

During the genome binning step with MetaBAT, we lost most of our SSU rRNA (16S) sequences. As far as we know, the causes for this common issue [1] have not yet been discussed in the metagenomic literature. In order to understand why those rRNA genes are discarded instead of being placed in their respective genome bin, we investigated the possible differences in sequencing coverage and tetranucleotide frequencies (TNFs) between the contigs bearing rRNA genes and the other contigs composing the MetaBAT genome bins.

Methods

For each contig, the sequencing coverage (totalAvgDepth) was extracted from the output files of MetaBAT [2]. Coverage values were then grouped by genome bin and plotted as box-and-whiskers plots, themselves grouped by metagenome. The contigs bearing unbinned SSU rRNA (16S) sequences were all pooled in a single special pseudo-bin (r) for each metagenome.

TNFs were obtained for each contig with the program compseq [from the EMBOSS software package; [3] using default settings. The resulting observed frequencies were used to perform 17 principal component analyses (PCAs; using the R function prcomp of the stats package; https://stat.ethz.ch/R-manual/R-devel/library/stats/html/prcomp.html), each one combining all the contigs of all genome bins from a single metagenome, including rRNA-bearing contigs, whether correctly binned by MetaBAT, manually affiliated by us or left unaffiliated (see main text). The two first components were then plotted for each metagenome.

For each contig bearing a rRNA sequence, the length was extracted from the output files of MetaBAT. rRNA-bearing contig lengths were then grouped in two categories (binned or unbinned) and plotted as box-and-whiskers plots. The same partition in two categories was used to compare modified Z-scores [4] measuring the deviation of the sequencing coverage of rRNA-bearing contigs with respect to the median coverage of the corresponding bins. Because of this requirement, rRNA genes left unaffiliated had to be excluded from the latter analysis.

Results and Discussion

Our working hypothesis was that the presence of multi-copy rRNA operons in Bacteria [5] would result in a higher sequencing coverage, which might interfere with MetaBAT binning. As shown in **Figure S2**, most contigs with rRNA genes that could not be automatically binned (in red) display markedly higher coverage values than “regular” genome bin contigs. In contrast, when the coverage of rRNA-bearing contigs is less extreme, they can be included in a genome bin (black arrowheads in

ULC027-bin2, ULC073-bin6, ULC146-bin1/bin3, and ULC179-bin3). When it is not the case, the problem stems from the divergent TNFs of rRNA genes. Indeed, the PCAs in **Figure S3** show that unbinned rRNA-bearing contigs (colored arrowheads) either cluster outside all bin clouds (ULC335-nobin) or cluster in a bin cloud for which the coverage is incompatible (ULC082-nobin, ULC179-nobin), whereas the five rRNA-bearing contigs that were automatically binned all lie in their respective bin cloud (black arrowheads). Inversely, there exist rRNA-bearing contigs that could not be binned because of their incompatible coverage only (e.g., ULC073-bin2, ULC084-bin1/bin2, ULC129-bin1), while a number could not be binned due to the incompatibility of both their coverage and TNFs (e.g., ULC007, ULC041, ULC068, ULC082-bin1, ULC146-bin2, ULC165, ULC187). Given that MetaBAT uses a distance combining the sequencing coverage and the TNFs to partition the contigs into distinct genome bins, it is not surprising that it discards the majority of rRNA-bearing contigs in light of these results.

To better understand the factors governing the binning fate of rRNA-bearing contigs, we compared their lengths. As shown in **Figure S4a**, binned contigs (in black) are considerably longer than unbinned contigs (in red). One possible cause for this difference might be that unbinned rRNA-bearing contigs exist in multiple copies in their respective genome. Such a situation would prevent the assembler software to extend the contigs much beyond the rRNA operon itself, because of the different genomic contexts surrounding the operon. Interestingly, unbinned rRNA-bearing contigs that we were able to manually affiliate a posteriori show a coverage significantly higher than the median coverage of their respective genome bin (**Figure S4b**), which suggests that they indeed do exist in multiple copies.

Conclusion

Our analyses suggest that the loss of rRNA sequences during the binning process carried out by MetaBAT is both driven by the higher sequencing coverage of these genes with respect to the coverage of other genome contigs and by their TNFs that are also markedly different. While these two factors matter, they are not completely independent. In particular, the divergent TNFs of rRNA genes are overwhelmed by the “regular” TNFs of the surrounding sequences when the rRNA operon is included in a long contig, which allows single-copy rRNA operons to be automatically binned. In contrast, multi-copy rRNA operons, which lie on shorter contigs, do not benefit from this dilution phenomenon. Consequently, their divergent TNFs add up to their higher sequencing coverage, resulting in the loss of rRNA genes. Altogether, automatic binning of rRNA genes appears to remain very challenging and would probably be improved by the use of longer sequencing reads, so as to assemble the multi-copy rRNA operons on longer contigs.

References

1. **Cornet L, Meunier L, Vlierberghe MV, Leonard RR, Durieu B, *et al.*** Consensus assessment of the contamination level of publicly available cyanobacterial genomes. *bioRxiv* 2018;301788. In review for PLOS ONE

2. **Kang DD, Froula J, Egan R, Wang Z**. MetaBAT, an efficient tool for accurately reconstructing single genomes from complex microbial communities. *PeerJ* 2015;3:e1165.

3. **Rice P, Longden I, Bleasby A**. EMBOSS: the European Molecular Biology Open Software Suite. *Trends Genet TIG* 2000;16:276–277.

4. **Iglewicz B, Hoaglin D**. The ASQC Basic References in Quality Control: Statistical Techniques. In: *How to Detect and Handle Outliers*. Milwaukee: ASQC Quality Press; 1993.

5. **Klappenbach JA, Dunbar JM, Schmidt TM**. rRNA Operon Copy Number Reflects Ecological Strategies of Bacteria. *Appl Environ Microbiol* 2000;66:1328–1333.

Figures

Figure S2: Comparison of the sequencing coverage of unbinned contigs bearing SSU rRNA (16S) genes to the coverage of other genome contigs grouped by genome bin and by metagenome. Numbers on the X axis refer to the bin numbers in the main text (r = rRNA genes) and always follow the same color code: from the first to the last, blue, green, purple, orange, yellow, brown and pink. Stars denote cyanobacterial bins, while arrowheads indicate the coverage (and bins) of the rRNA-bearing contigs that were automatically binned by MetaBAT.

Figure S3: Principal Component Analyses of tetranucleotide frequencies in all contigs for each metagenome. Unbinned contigs bearing rRNA genes are colored in red. Each genome bin is colored, from the first to the last, in blue, green, purple, orange, yellow, brown and pink (as in **Figure S1**). Cyanobacterial bins were circled by hand using dashed lines. Arrowheads point to rRNA-bearing contigs, either automatically binned by MetaBAT (in black) or manually affiliated (colored after their MetaBAT bin) or left unaffiliated (in red).

Figure S4: Comparison between binned and unbinned contigs bearing rRNA genes for a) their length and b) their deviation from the median sequencing coverage of their respective bin. Numbers on top of box-and-whiskers are the number of contigs contributing to each box. Unbinned contigs used in **b)** are restricted to those that we were able to manually affiliate.

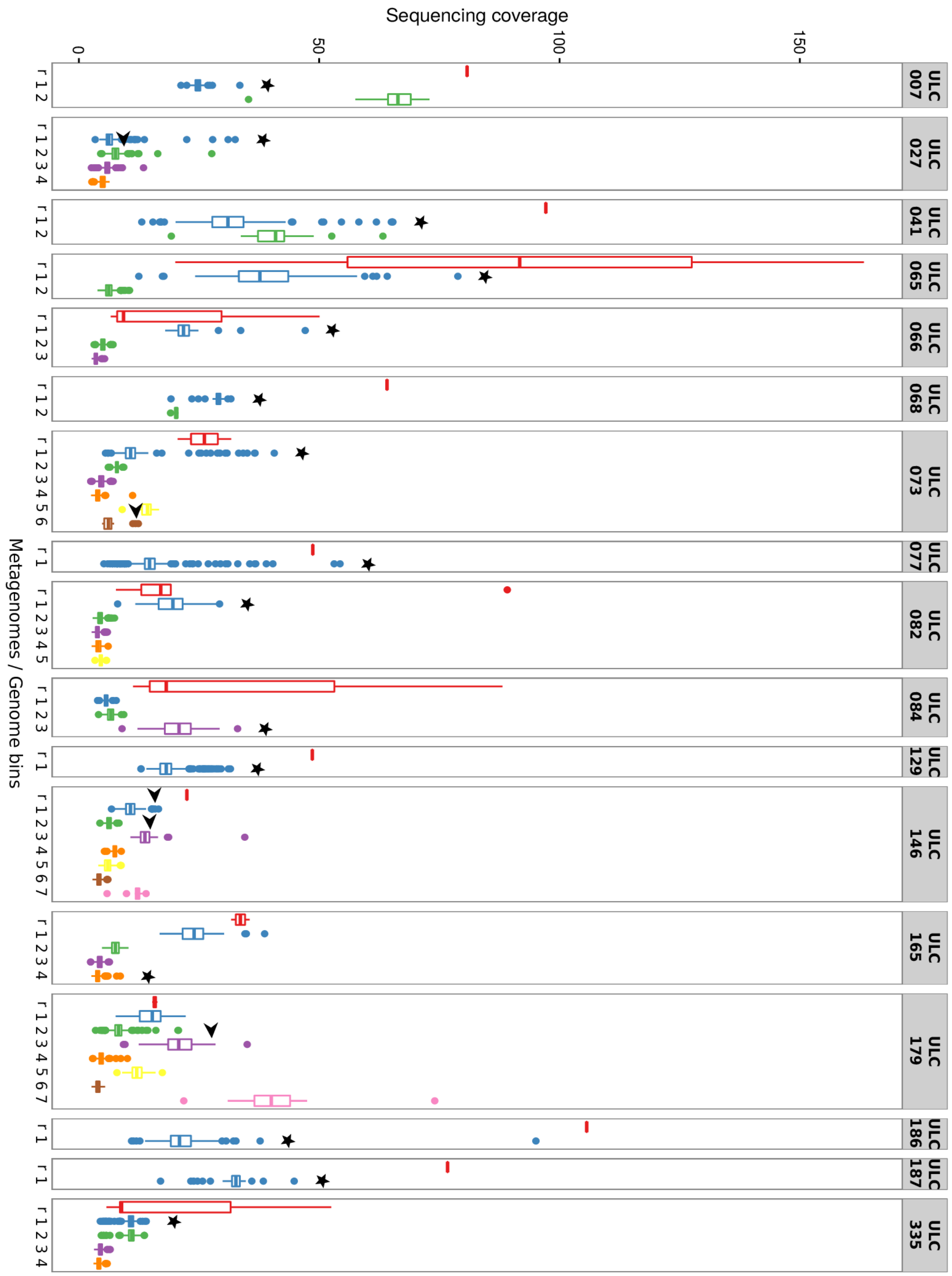
Sequencing coverage
0
50
100
150
Metagenomes / Genome bins
ULC 007
ULC 027
ULC 041
ULC 065
ULC 066
ULC 068
ULC 073
ULC 077
ULC 082
ULC 084
ULC 129
ULC 146
ULC 165
ULC 179
ULC 186
ULC 187
ULC 335

Figure S2

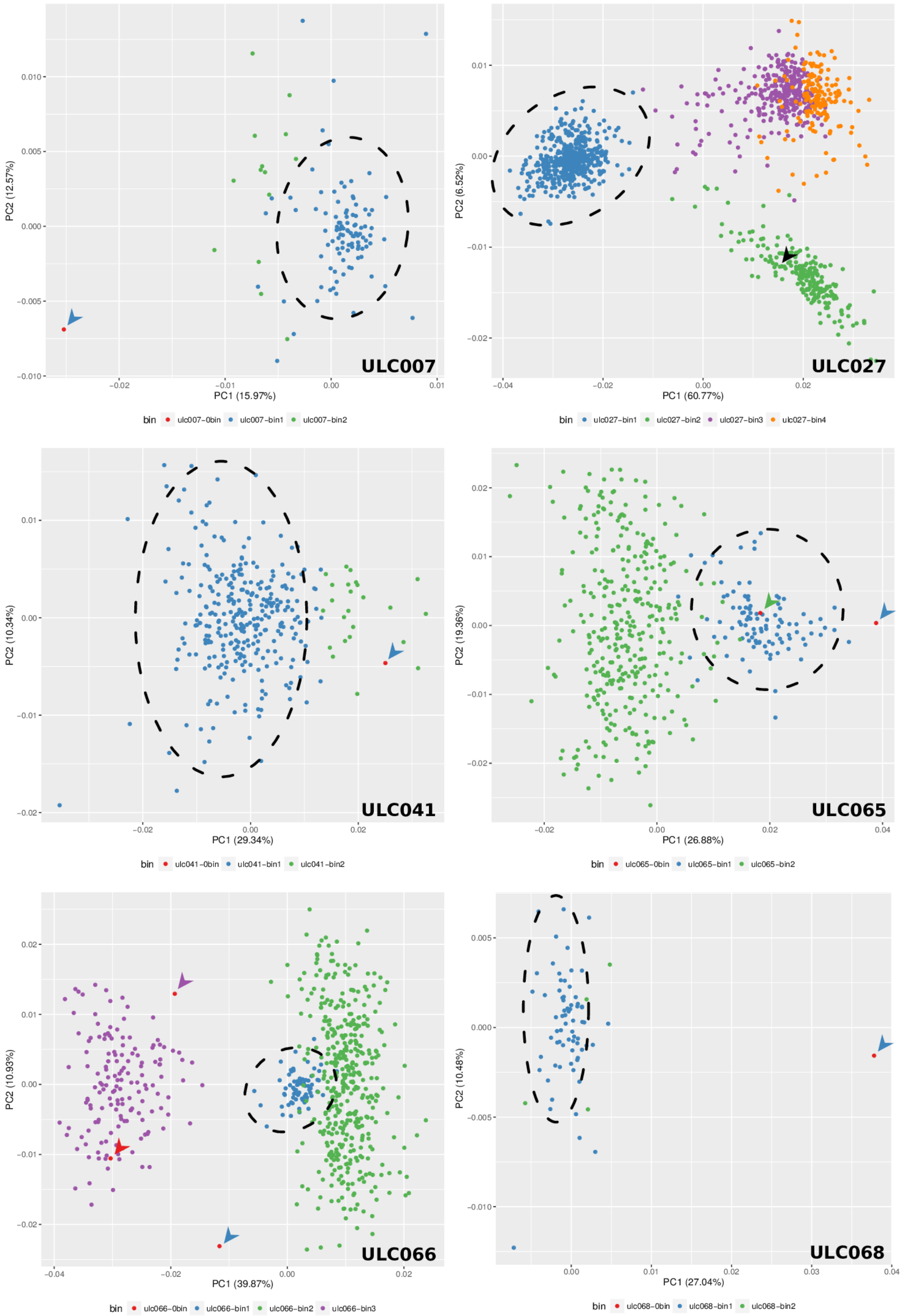
ULC007
PC1 (15.97%)
PC2 (12.57%)
bin ulc007-0bin ulc007-bin1 ulc007-bin2
ULC027
PC1 (60.77%)
PC2 (6.52%)
bin ulc027-bin1 ulc027-bin2 ulc027-bin3 ulc027-bin4
ULC041
PC1 (29.34%)
PC2 (10.34%)
bin ulc041-0bin ulc041-bin1 ulc041-bin2
ULC065
PC1 (26.88%)
PC2 (19.36%)
bin ulc065-0bin ulc065-bin1 ulc065-bin2
ULC066
PC1 (39.87%)
PC2 (10.93%)
bin ulc066-0bin ulc066-bin1 ulc066-bin2 ulc066-bin3
ULC068
PC1 (27.04%)
PC2 (10.48%)
bin ulc068-0bin ulc068-bin1 ulc068-bin2

Figure S3

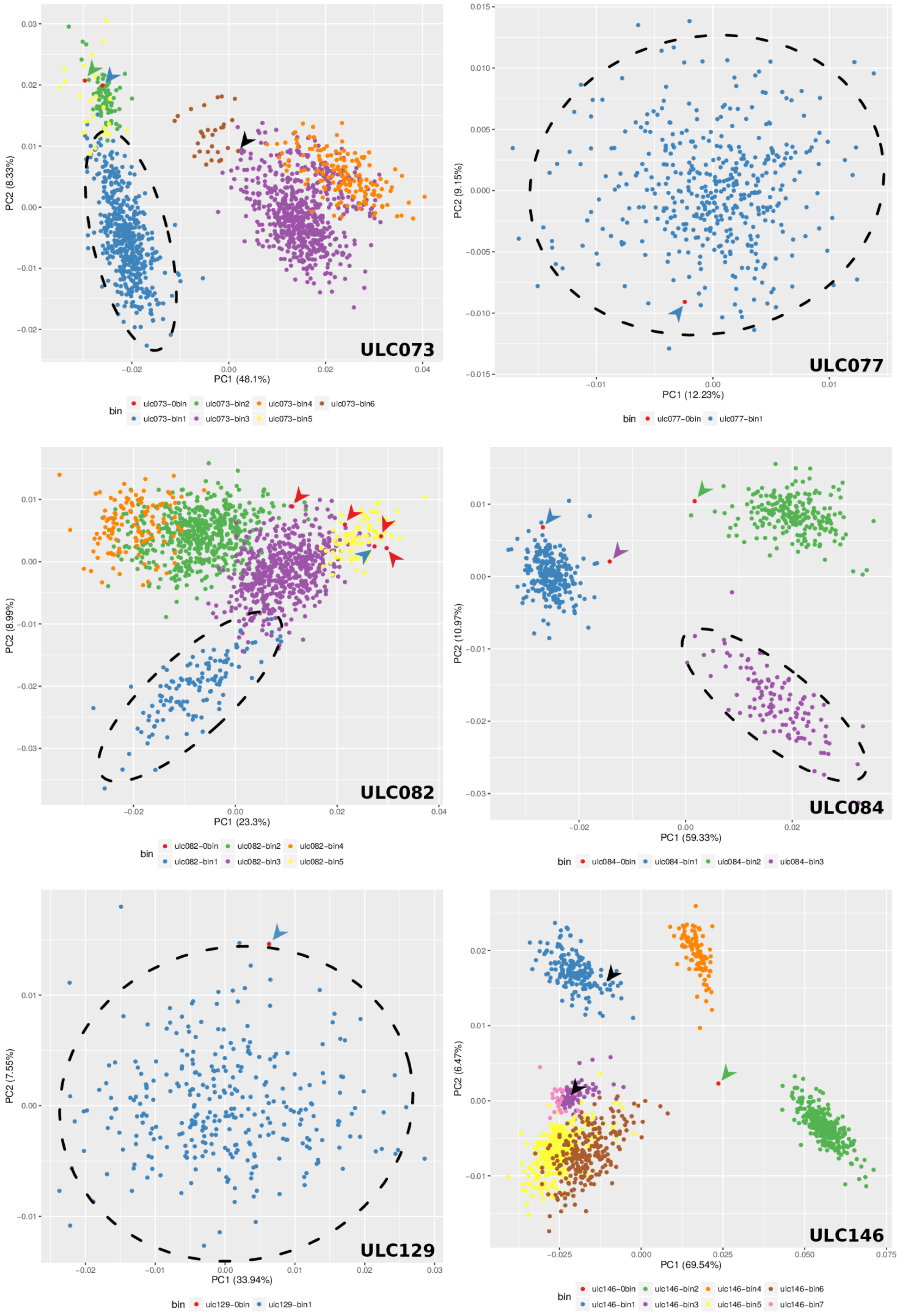

Figure S3 (continued)

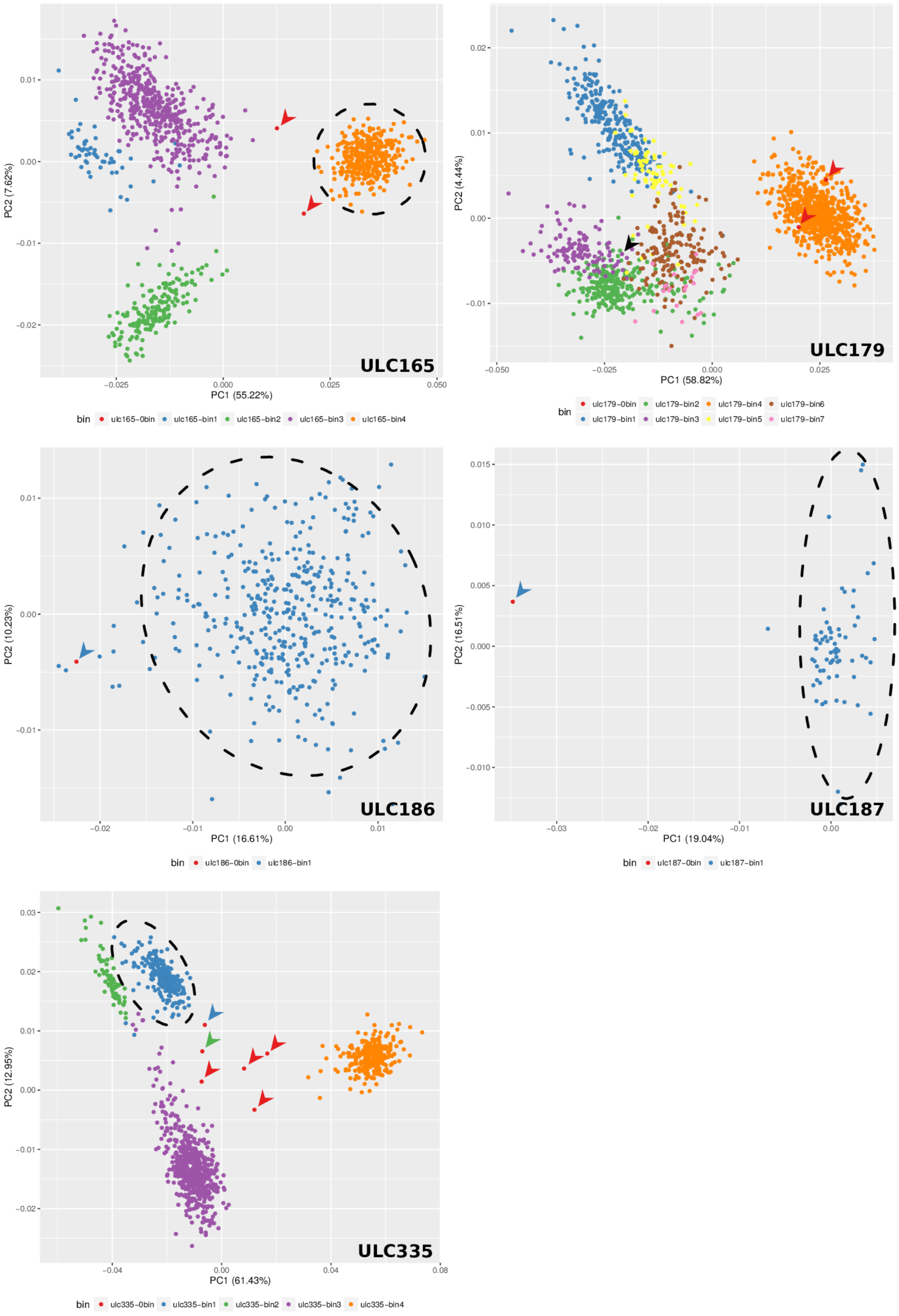

Figure S3 (continued)

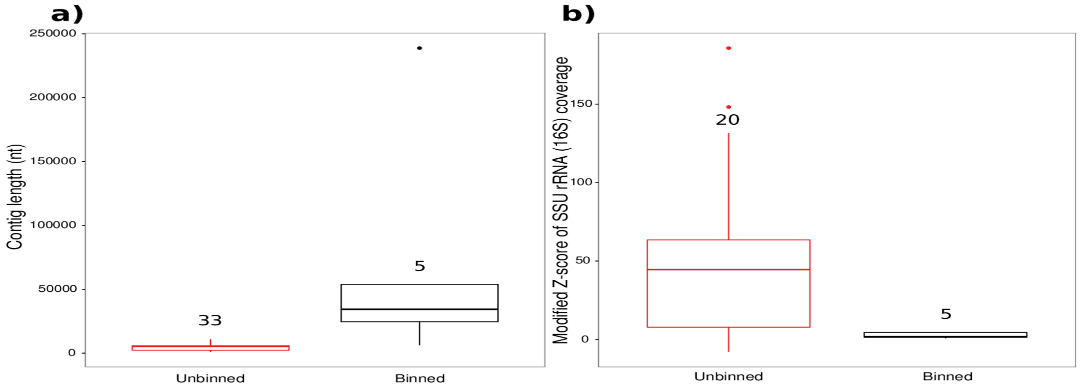
a)
250000
200000
150000
100000
50000
0
Contig length (nt)
33
5
Unbinned
Binned
b)
Modified Z-score of SSU rRNA (16S) coverage
150
100
50
0
20
5
Unbinned
Binned

Figure S4

Supplemental Appendix S3

Our phylogenomic analysis of the cyanobacterial microbiome has shown that five sets of foreign bins (11 bins in total) appear indistinguishable in the tree (**Figure 2**). This can be explained by two causes: 1) association of the same or very closely related microbiome taxa to the Cyanobacteria, resulting in redundancy across multiple cyanobacterial cultures or 2) cross-contamination of cultures during strain propagation or of genomic DNA during nucleic acid extraction. In this supplemental note, we investigated the issue through all-vs-all BLASTN analyses on whole genome bins, using first the standard Average Nucleotide Identity (ANI) and then a newly developed metric, dubbed the Identical Genome Fraction (IGF), designed to better take into account the differential coverage of the bins during their comparison.

Methods

With the aim of not missing any possible case of redundancy or cross-contamination, we included an additional bin (called nobin) to each metagenome, containing the scaffolds rejected by MetaBAT during the binning process. ANI was computed with the Python script average_nucleotide_identity.py from the pyani software package [1] on genome bins split into overlapping fragments of 1020 nt, whereas IGF (introduced below) was computed with a custom Perl script (available upon request) on genome bins split into non-overlapping fragments (= pseudo-reads) of 250 nt. In both cases, BLASTN [2] was used to blast each split genome bin against all unsplit genome bins, using options inspired by [3]: -dust no -xdrop_ungap 150 -xdrop_gap 150 -xdrop_gap_final 150 -evalue 1e-15 -max_target_seqs 1.

To compute a neighbor-joining tree with the neighbor program of the PHYLIP package [4, 5], we first built a reduced version of the IGF matrix by selecting only the genome bins involved in any pair with an IGF value ≥75%. This IGF tree was then made comparable with the small phylogenomic tree of the co-cultivated organisms by pruning the latter tree with the prune-tree.pl script (part of the Bio-MUST-Core software package; D. Baurain; https://metacpan.org/release/Bio-MUST-Core). Details about sampling sites were retrieved from the BCCM/ULC web site (http://bccm.belspo.be/catalogues).

Results and Discussion

We identified 20 bins (including the 11 bins discussed above) sharing a very high ANI (≥98%) with another bin (**Table S3**), thus well over the species threshold (94%) defined in Goris et al. (2007) [3]. However, ANI was not suitable for this study because it is computed only from homologous fragments (BLASTN identity ≥30%) [3], which can collectively cover only a (very) small part of the genomes (**Table S4**).

Hence, among the 20 high ANI matches, only 7 were computed on ≥80% of the genome bins (**Table S4**) (median = 52%, IQR = 68%). Moreover, ANI does not take into account any potential difference in completeness between the metagenomic bins under comparison. This is an important concern because it complicates the interpretation of ANI values. In contrast, the similarity distance of Mauve [6] would be a more suitable metric, because it is defined as the number of aligned nucleotides (estimated with nucmer; [7]) between the two genomes divided by their average size. Yet, when comparing two genome bins with a large difference in completeness, such use of the average size results in a mechanically reduced value of the similarity distance. As no tool was adapted to our purpose, i.e., to estimate bin similarity based on all fragments of two genome bins with possibly different completeness values, we developed the Identical Genome Fraction (IGF), a metric inspired from both the ANI and Mauve similarity distance. IGF is defined as the ratio between the number of identical fragments between two bins (preferring the BLASTN direction yielding the highest estimate) and the number of fragments in the smallest of the two bins: IGF = $\max(A \cap B, B \cap A) / \min(A, B)$. By default, two fragments are considered as identical when perfectly aligning (100% identity) over their full length (100% coverage).

We identified 13 bins (included in the 20 ANI matches) displaying a conspicuously high IGF (≥75%) with another bin (**Table S5**). The maximum IGF observed between any two bins (ULC146-bin4/ULC073-bin2) was 92.4%. We tested different identity thresholds and even with a threshold set at 25% (instead of 100%), no perfect match could be detected between our genome bins (at the exception of self-matches). This suggests that foreign bin redundancy is not the result of cross-contamination between cultures. As expected, the 11 bins identified in the microbiome phylogenomic tree are all comprised in these 13 high-IGF bins. ULC073-bin3 (included in the tree) and ULC066-bin3 (not in the tree, see main text) are the two additional bins with IGF ≥75%. **Figure S5** shows that, contrary to **Figure 2**, the 13 genome bins yield distinct terminal branches in the neighbor-joining tree based on the IGF matrix. This increased resolution is not surprising, considering that IGF is computed from full genome bins and not only from ribosomal proteins. Nevertheless, the bin clusters present in **Figure S5** all correspond to bin clusters observed in **Figure 2**, indicating that the two approaches are congruent for our purpose. Interestingly, all bin clusters comprise strains from different sampling sites, at the exception of the cluster composed of ULC146-bin1 and ULC165-bin3, which were both sampled in Antarctic Sør Rondane Mountains. Finally, no bin pair involving artificial bins of metagenomic contigs rejected by MetaBAT (nobins) was identified.

Conclusion

Our analysis failed to bring evidence for cross-contamination between BCCM/ULC cyanobacterial cultures. The 11 foreign genome bins that appear indistinguishable in

the microbiome phylogenomic tree thus seem to correspond to very similar, but distinct, taxa present in multiple cultures despite distant sampling sites. This result suggests that these 11 genome bins belong to associated organisms in close interaction with Cyanobacteria, independently of their geographical location, even when Cyanobacteria were sampled from the opposite Earth poles. This is reminiscent of the Baas-Becking statement ‘Everything is everywhere, but the environment selects’ [8], where the ecological selection would mostly be defined by the presence of Cyanobacteria. However, the observation that the IGF metric can differentiate closely related bins indicates that evolutionary processes are at play, processes that could be due to long-term co-evolution.

Ideally, IGF should be repetitively estimated from multiple samples of the same culture to define a lower bound for identical organisms. Indeed, independent samples of the same organism would not necessarily yield an IGF of 100% due to slight variations during sequencing and assembly. Since we only had one sample of each culture in our experimental design, this could not be tested. For the same reason, we were not able to use MetaGen, a reference-free learning program not based on coverage [9]. This is somewhat unfortunate as this very recent metagenomic tool reportedly performs better than MetaBAT when given multiple samples (≥ 10) of the same mixtures [9].

References

1. **Pritchard L, H. Glover R, Humphris S, G. Elphinstone J, K. Toth I**. Genomics and taxonomy in diagnostics for food security: soft-rotting enterobacterial plant pathogens. *Anal Methods* 2016;8:12–24.

2. **Boratyn GM, Schäffer AA, Agarwala R, Altschul SF, Lipman DJ, *et al.*** Domain enhanced lookup time accelerated BLAST. *Biol Direct* 2012;7:12.

3. **Goris J, Konstantinidis KT, Klappenbach JA, Coenye T, Vandamme P, *et al.*** DNA–DNA hybridization values and their relationship to whole-genome sequence similarities. *Int J Syst Evol Microbiol* 2007;57:81–91.

4. **Felsenstein J**. Mathematical Evolutionary Theory. *Science*. http://link.galegroup.com/apps/doc/A8273695/AONE?sid=googlescholar (1989, accessed 11 March 2018).

5. **Felsenstein J**. Accuracy of Coalescent Likelihood Estimates: Do We Need More Sites, More Sequences, or More Loci? *Mol Biol Evol* 2006;23:691–700.

6. **Darling ACE, Mau B, Blattner FR, Perna NT**. Mauve: Multiple Alignment of Conserved Genomic Sequence With Rearrangements. *Genome Res* 2004;14:1394–1403.

7. **Kurtz S, Phillippy A, Delcher AL, Smoot M, Shumway M, *et al.*** Versatile and open software for comparing large genomes. *Genome Biol* 2004;5:R12.

8. **De Wit R, Bouvier T**. ‘Everything is everywhere, but, the environment selects’; what did Baas Becking and Beijerinck really say? *Environ Microbiol* 2006;8:755–758.

9. **Xing X, Liu JS, Zhong W**. MetaGen: reference-free learning with multiple metagenomic samples. *Genome Biol* 2017;18:187.

Figures and Tables

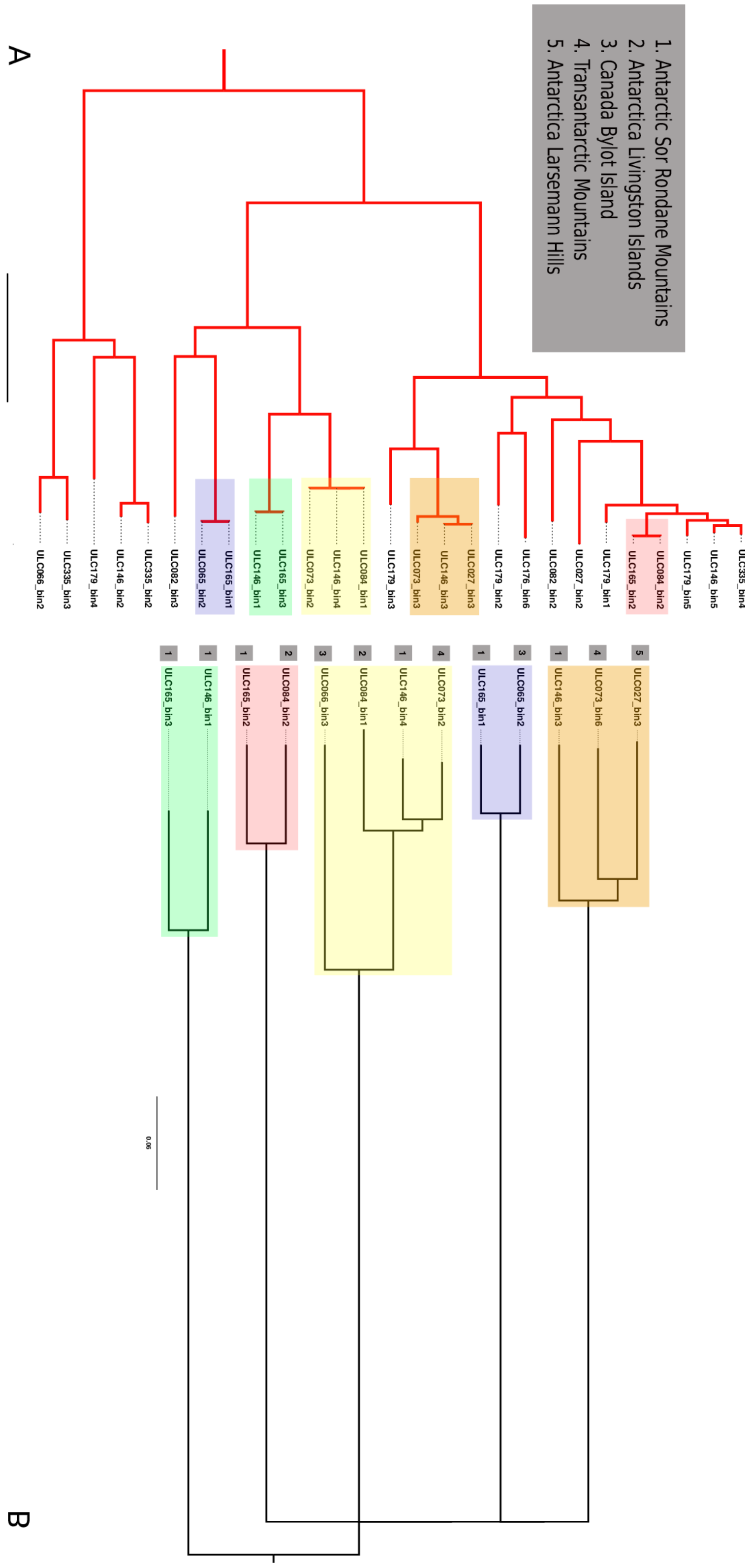

Figure S5

Figure S5: Mirror comparison between a reduced version of the microbiome phylogenomic tree and an IGF neighbor-joining tree. The phylogenomic tree was inferred under the CAT+Γ_4 model from a supermatrix made of 53 ribosomal genes. The 27 genome bins (**A**) were pruned out of this tree. The second tree (**B**) was inferred using neighbor-joining, based on a reduced version of the IGF matrix. Sampling information was collected from the BCCM/ULC website.

Table S3: Average Nucleotide Identity identity matrix as computed by pyani.

Table S4: Average Nucleotide Identity coverage matrix as computed by pyani.

Table S5: Identical Genome Fraction matrix (IGF).

	ULC335-bin1	ULC335-bin2	ULC335-bin3	ULC335-bin4	ULC335-nobin	ULC007-bin1	ULC007-bin2	ULC007-nobin
**ULC335-bin1**		0.942	0.704	0.723	0.956	0.743	0.741	0.739
**ULC335-bin2**	0.769		0.729	0.000	0.815	0.716	0.000	0.000
**ULC335-bin3**	0.704	0.730		0.714	0.787	0.723	0.000	0.000
**ULC335-bin4**	0.723	0.000	0.714		0.775	0.726	0.000	0.000
**ULC335-nobin**	0.912	0.890	0.818	0.774		0.736	0.696	0.812
**ULC007-bin1**	0.743	0.716	0.720	0.727	0.739		0.798	0.904
**ULC007-bin2**	0.753	0.000	0.000	0.000	0.000	0.786		0.843
**ULC007-nobin**	0.745	0.000	0.000	0.000	0.854	0.930	0.858	
**ULC027-bin1**	0.738	0.725	0.714	0.745	0.735	0.748	0.782	0.737
**ULC027-bin2**	0.707	0.748	0.717	0.771	0.760	0.727	0.000	0.817
**ULC027-bin3**	0.712	0.717	0.723	0.767	0.786	0.734	0.000	
**ULC027-bin4**	0.000	0.000	0.710	0.762	0.773	0.738	0.000	0.000
**ULC027-nobin**	0.735	0.718	0.720	0.779	0.784	0.738	0.704	0.851
**ULC041-bin1**	0.737	0.701	0.732	0.739	0.747	0.749	0.806	0.865
**ULC041-bin2**	0.725	0.000	0.780	0.727	0.742	0.747	0.760	
**ULC041-nobin**	0.743	0.000	0.000	0.000	0.839	0.762	0.000	0.837
**ULC065-bin1**	0.726	0.000	0.729	0.750	0.741	0.737	0.810	0.951
**ULC065-bin2**	0.713	0.701	0.726	0.767	0.764	0.721	0.694	0.000
**ULC065-nobin**	0.734	0.741	0.724	0.790	0.805	0.753	0.809	0.845
**ULC066-bin1**	0.742	0.730	0.711	0.720	0.730	0.742	0.777	0.934
**ULC066-bin2**	0.727	0.727	0.742	0.706	0.751	0.735	0.000	
**ULC066-bin3**	0.716	0.000	0.721	0.754	0.745	0.722	0.000	0.000
**ULC066-nobin**	0.725	0.724	0.747	0.786	0.788	0.730	0.687	0.862
**ULC068-bin1**	0.741	0.715	0.722	0.707	0.734	0.742	0.760	0.831
**ULC068-bin2**	0.769	0.000	0.717	0.000	0.779	0.739	0.000	0.758
**ULC068-nobin**	0.746	0.790	0.732	0.799	0.790	0.734	0.000	0.819
**ULC073-bin1**	0.736	0.725	0.727	0.746	0.745	0.751	0.695	0.871
**ULC073-bin2**	0.726	0.719	0.726	0.732	0.753	0.712	0.000	0.997
**ULC073-bin3**	0.722	0.708	0.719	0.769	0.782	0.724	0.000	
**ULC073-bin4**	0.000	0.701	0.000	0.772	0.772	0.729	0.735	0.000
**ULC073-bin5**	0.746	0.000	0.000	0.000	0.730	0.755	0.761	0.740
**ULC073-bin6**	0.000	0.000	0.000	0.774	0.803	0.000	0.000	0.801
**ULC073-nobin**	0.736	0.716	0.723	0.773	0.796	0.749	0.719	0.856
**ULC077-bin1**	0.742	0.720	0.714	0.724	0.726	0.759	0.761	0.918
**ULC077-nobin**	0.737	0.725	0.727	0.792	0.794	0.773	0.797	0.911
**ULC082-bin1**	0.726	0.000	0.718	0.755	0.740	0.742	0.817	0.782
**ULC082-bin2**	0.711	0.739	0.719	0.860	0.824	0.729	0.000	0.000
**ULC082-bin3**	0.719	0.713	0.738	0.761	0.782	0.722	0.000	0.000
**ULC082-bin4**	0.000	0.718	0.000	0.904	0.884	0.717	0.000	0.000
**ULC082-bin5**	0.000	0.000	0.000	0.695	0.769	0.000	0.000	0.000
**ULC082-nobin**	0.719	0.711	0.736	0.858	0.837	0.725	0.698	0.822
**ULC084-bin1**	0.719	0.706	0.719	0.743	0.753	0.728	0.690	0.000
**ULC084-bin2**	0.724	0.702	0.711	0.810	0.798	0.725	0.000	0.709
**ULC084-bin3**	0.728	0.743	0.713	0.752	0.745	0.741	0.758	0.776
**ULC084-nobin**	0.720	0.728	0.721	0.779	0.799	0.741	0.786	0.842
**ULC129-bin1**	0.736	0.724	0.714	0.722	0.737	0.746	0.753	0.756
**ULC129-nobin**	0.742	0.741	0.744	0.772	0.801	0.750	0.727	0.821
**ULC146-bin1**	0.701	0.693	0.729	0.750	0.842	0.721	0.000	0.782
**ULC146-bin2**	0.721	0.773	0.726	0.738	0.729	0.725	0.000	0.000
**ULC146-bin3**	0.717	0.000	0.716	0.775	0.784	0.717	0.000	0.818
**ULC146-bin4**	0.716	0.717	0.730	0.742	0.751	0.722	0.000	0.000
**ULC146-bin5**	0.702	0.720	0.721	0.853	0.847	0.723	0.000	0.802
**ULC146-bin6**	0.000	0.000	0.000	0.774	0.770	0.737	0.000	0.000
**ULC146-bin7**	0.000	0.000	0.747	0.747	0.776	0.726	0.000	0.000
**ULC146-nobin**	0.748	0.762	0.725	0.805	0.802	0.749	0.774	0.831
**ULC165-bin1**	0.704	0.725	0.718	0.765	0.760	0.715	0.695	0.000
**ULC165-bin2**	0.722	0.703	0.706	0.811	0.799	0.725	0.000	0.692
**ULC165-bin3**	0.000	0.000	0.749	0.751	0.842	0.734	0.000	0.000
**ULC165-bin4**	0.749	0.709	0.733	0.690	0.747	0.758	0.786	0.800
**ULC165-nobin**	0.744	0.764	0.726	0.778	0.821	0.758	0.772	0.801
**ULC179-bin1**	0.712	0.717	0.721	0.827	0.818	0.726	0.000	0.000
**ULC179-bin2**	0.706	0.000	0.719	0.783	0.780	0.720	0.000	0.737
**ULC179-bin3**	0.717	0.700	0.713	0.767	0.763	0.731	0.000	0.815
**ULC179-bin4**	0.727	0.726	0.740	0.749	0.735	0.728	0.694	0.000
**ULC179-bin5**	0.693	0.000	0.717	0.832	0.820	0.727	0.000	0.000
**ULC179-bin6**	0.000	0.000	0.725	0.762	0.762	0.718	0.000	0.000
**ULC179-bin7**	0.000	0.000	0.000	0.758	0.756	0.000	0.000	0.000
**ULC179-nobin**	0.749	0.724	0.744	0.799	0.783	0.747	0.780	0.822
**ULC186-bin1**	0.738	0.723	0.727	0.744	0.746	0.751	0.794	0.776
**ULC186-nobin**	0.761	0.760	0.740	0.777	0.908	0.748	0.788	0.867
**ULC187-bin1**	0.747	0.729	0.717	0.718	0.744	0.741	0.701	0.778
**ULC187-nobin**	0.740	0.720	0.715	0.805	0.797	0.740	0.716	0.857

	ULC027-bin1	ULC027-bin2	ULC027-bin3	ULC027-bin4	ULC027-nobin	ULC041-bin1	ULC041-bin2	ULC041-nobin
**ULC335-bin1**	0.737	0.707	0.712	0.000	0.734	0.737	0.725	0.746
**ULC335-bin2**	0.725	0.748	0.000	0.000	0.719	0.701	0.000	0.000
**ULC335-bin3**	0.717	0.717	0.723	0.710	0.722	0.731	0.780	0.000
**ULC335-bin4**	0.750	0.772	0.770	0.759	0.782	0.743	0.727	0.000
**ULC335-nobin**	0.737	0.763	0.784	0.777	0.783	0.750	0.736	0.809
**ULC007-bin1**	0.748	0.727	0.732	0.738	0.734	0.749	0.746	0.754
**ULC007-bin2**	0.778	0.000	0.000	0.000	0.709	0.789	0.760	0.000
**ULC007-nobin**	0.793	0.854	0.928	0.000	0.854	0.871	0.989	0.837
**ULC027-bin1**		0.734	0.730	0.706	0.859	0.761	0.760	0.749
**ULC027-bin2**	0.734		0.758	0.746	0.772	0.736	0.727	0.769
**ULC027-bin3**	0.733	0.758		0.771	0.791	0.735	0.716	0.711
**ULC027-bin4**	0.709	0.750	0.764		0.784	0.728	0.000	0.000
**ULC027-nobin**	0.892	0.770	0.792	0.782		0.777	0.829	0.866
**ULC041-bin1**	0.761	0.736	0.737	0.728	0.775		0.883	0.929
**ULC041-bin2**	0.760	0.727	0.716	0.000	0.840	0.858		0.878
**ULC041-nobin**	0.747	0.769	0.711	0.000	0.882	0.959	0.948	
**ULC065-bin1**	0.737	0.739	0.742	0.755	0.739	0.759	0.739	0.806
**ULC065-bin2**	0.719	0.753	0.750	0.745	0.752	0.743	0.724	0.741
**ULC065-nobin**	0.738	0.766	0.959	0.951	0.924	0.745	0.763	0.836
**ULC066-bin1**	0.738	0.726	0.723	0.000	0.739	0.741	0.741	0.876
**ULC066-bin2**	0.708	0.725	0.712	0.000	0.719	0.712	0.000	0.000
**ULC066-bin3**	0.722	0.729	0.716	0.000	0.989	0.731	0.000	0.000
**ULC066-nobin**	0.735	0.977	0.949	0.941	0.920	0.742	0.733	0.851
**ULC068-bin1**	0.739	0.722	0.729	0.717	0.757	0.740	0.767	0.769
**ULC068-bin2**	0.709	0.000	0.000	0.000	0.736	0.736	0.000	0.000
**ULC068-nobin**	0.733	0.899	0.776	0.769	0.923	0.751	0.755	0.818
**ULC073-bin1**	0.758	0.729	0.732	0.727	0.764	0.986	0.967	0.961
**ULC073-bin2**	0.727	0.735	0.743	0.733	0.988	0.729	0.000	
**ULC073-bin3**	0.725	0.754	0.987	0.985	0.893	0.732	0.718	0.724
**ULC073-bin4**	0.742	0.753	0.797	0.867	0.956	0.742	0.000	
**ULC073-bin5**	0.775	0.724	0.711	0.000	0.884	0.978	0.977	0.959
**ULC073-bin6**	0.700	0.818	0.995	0.859	0.926	0.749	0.000	0.000
**ULC073-nobin**	0.748	0.763	0.887	0.918	0.957	0.954	0.950	0.969
**ULC077-bin1**	0.747	0.724	0.721	0.727	0.745	0.750	0.749	0.774
**ULC077-nobin**	0.746	0.774	0.905	0.901	0.946	0.757	0.760	0.839
**ULC082-bin1**	0.739	0.739	0.741	0.728	0.751	0.757	0.742	0.761
**ULC082-bin2**	0.729	0.758	0.761	0.755	0.767	0.747	0.764	0.000
**ULC082-bin3**	0.735	0.747	0.752	0.737	0.755	0.740	0.000	0.723
**ULC082-bin4**	0.726	0.769	0.772	0.757	0.775	0.759	0.709	0.000
**ULC082-bin5**	0.000	0.710	0.730	0.000	0.762	0.704	0.000	0.000
**ULC082-nobin**	0.726	0.762	0.763	0.750	0.797	0.742	0.753	0.811
**ULC084-bin1**	0.731	0.733	0.729	0.721	0.986	0.725	0.000	
**ULC084-bin2**	0.732	0.759	0.761	0.751	0.761	0.746	0.732	0.000
**ULC084-bin3**	0.742	0.740	0.740	0.724	0.749	0.757	0.729	0.780
**ULC084-nobin**	0.755	0.775	0.987	0.985	0.880	0.738	0.722	0.853
**ULC129-bin1**	0.788	0.728	0.729	0.733	0.795	0.754	0.750	0.743
**ULC129-nobin**	0.821	0.985	0.907	0.909	0.942	0.783	0.790	0.848
**ULC146-bin1**	0.739	0.746	0.751	0.740	0.880	0.735	0.722	0.798
**ULC146-bin2**	0.719	0.763	0.717	0.000	0.719	0.701	0.000	0.000
**ULC146-bin3**	0.728	0.759	0.996	0.992	0.878	0.734	0.723	0.982
**ULC146-bin4**	0.728	0.730	0.733	0.723	0.987	0.739	0.702	0.000
**ULC146-bin5**	0.727	0.768	0.769	0.733	0.774	0.731	0.722	0.775
**ULC146-bin6**	0.730	0.756	0.828	0.777	0.981	0.739	0.000	0.000
**ULC146-bin7**	0.000	0.749	0.993	0.995	0.892	0.000	0.685	0.719
**ULC146-nobin**	0.743	0.766	0.806	0.805	0.954	0.745	0.723	0.824
**ULC165-bin1**	0.724	0.749	0.754	0.747	0.750	0.747	0.756	0.737
**ULC165-bin2**	0.733	0.757	0.760	0.749	0.762	0.738	0.732	0.000
**ULC165-bin3**	0.730	0.754	0.754	0.745	0.877	0.743	0.760	0.835
**ULC165-bin4**	0.744	0.725	0.717	0.000	0.746	0.746	0.751	0.000
**ULC165-nobin**	0.751	0.765	0.856	0.837	0.884	0.749	0.772	0.821
**ULC179-bin1**	0.724	0.761	0.766	0.745	0.775	0.746	0.707	0.720
**ULC179-bin2**	0.731	0.764	0.766	0.758	0.816	0.740	0.730	0.722
**ULC179-bin3**	0.730	0.759	0.776	0.771	0.771	0.731	0.746	0.799
**ULC179-bin4**	0.721	0.730	0.743	0.734	0.732	0.736	0.727	0.000
**ULC179-bin5**	0.726	0.762	0.763	0.751	0.780	0.732	0.000	0.000
**ULC179-bin6**	0.740	0.755	0.770	0.746	0.768	0.727	0.000	0.710
**ULC179-bin7**	0.000	0.761	0.765	0.000	0.755	0.000	0.000	0.000
**ULC179-nobin**	0.745	0.769	0.768	0.747	0.785	0.743	0.738	0.824
**ULC186-bin1**	0.759	0.741	0.726	0.726	0.747	0.842	0.832	0.854
**ULC186-nobin**	0.750	0.768	0.760	0.744	0.786	0.846	0.858	0.888
**ULC187-bin1**	0.744	0.727	0.723	0.000	0.735	0.737	0.739	0.767
**ULC187-nobin**	0.762	0.778	0.975	0.973	0.928	0.751	0.728	0.844

	ULC065-bin1	ULC065-bin2	ULC065-nobin	ULC066-bin1	ULC066-bin2	ULC066-bin3	ULC066-nobin	ULC068-bin1
**ULC335-bin1**	0.726	0.713	0.730	0.743	0.727	0.716	0.726	0.741
**ULC335-bin2**	0.000	0.000	0.743	0.727	0.727	0.000	0.724	0.711
**ULC335-bin3**	0.730	0.733	0.722	0.711	0.743	0.721	0.748	0.723
**ULC335-bin4**	0.751	0.770	0.791	0.723	0.706	0.754	0.789	0.707
**ULC335-nobin**	0.745	0.766	0.805	0.726	0.753	0.747	0.786	0.736
**ULC007-bin1**	0.737	0.719	0.753	0.742	0.727	0.719	0.732	0.742
**ULC007-bin2**	0.832	0.694	0.704	0.777	0.000	0.000	0.000	0.762
**ULC007-nobin**	0.963	0.000	0.893	0.942		0.000	0.918	0.859
**ULC027-bin1**	0.737	0.718	0.731	0.738	0.709	0.722	0.730	0.739
**ULC027-bin2**	0.740	0.753	0.762	0.728	0.725	0.715	0.989	0.727
**ULC027-bin3**	0.742	0.750	0.969	0.726	0.712	0.716	0.960	0.731
**ULC027-bin4**	0.755	0.752	0.968	0.000	0.000	0.000	0.956	0.717
**ULC027-nobin**	0.739	0.756	0.921	0.740	0.722	0.982	0.923	0.754
**ULC041-bin1**	0.758	0.744	0.740	0.741	0.712	0.729	0.744	0.740
**ULC041-bin2**	0.743	0.724	0.776	0.741	0.000	0.000	0.733	0.773
**ULC041-nobin**	0.809	0.741	0.909	0.871	0.000	0.000	0.902	0.772
**ULC065-bin1**		0.743	0.762	0.730	0.713	0.710	0.751	0.731
**ULC065-bin2**	0.742		0.762	0.699	0.740	0.748	0.756	0.726
**ULC065-nobin**	0.801	0.767		0.806	0.730	0.745	0.935	0.760
**ULC066-bin1**	0.729	0.699	0.816		0.710	0.728	0.788	0.824
**ULC066-bin2**	0.713	0.740	0.736	0.710		0.000	0.824	0.712
**ULC066-bin3**	0.715	0.748	0.744	0.728	0.000		0.793	0.727
**ULC066-nobin**	0.751	0.758	0.936	0.825	0.853	0.789		0.800
**ULC068-bin1**	0.732	0.724	0.757	0.825	0.712	0.721	0.800	
**ULC068-bin2**	0.000	0.000	0.000	0.828	0.000	0.000	0.000	0.841
**ULC068-nobin**	0.754	0.977	0.934	0.835	0.734	0.748	0.913	0.915
**ULC073-bin1**	0.761	0.738	0.747	0.737	0.726	0.728	0.740	0.737
**ULC073-bin2**	0.734	0.742	0.749	0.733	0.726	0.993	0.995	0.721
**ULC073-bin3**	0.740	0.754	0.961	0.720	0.724	0.734	0.945	0.726
**ULC073-bin4**	0.747	0.759	0.785	0.000	0.740	0.776	0.783	0.720
**ULC073-bin5**	0.769	0.000	0.739	0.740	0.000		0.927	0.773
**ULC073-bin6**	0.726	0.000	0.987	0.686	0.703	0.000	0.978	0.000
**ULC073-nobin**	0.751	0.756	0.921	0.767	0.745	0.868	0.894	0.736
**ULC077-bin1**	0.736	0.727	0.744	0.747	0.716	0.708	0.748	0.744
**ULC077-nobin**	0.750	0.987	0.927	0.795	0.713	0.983	0.973	0.751
**ULC082-bin1**	0.821	0.741	0.755	0.736	0.722	0.725	0.742	0.737
**ULC082-bin2**	0.737	0.750	0.820	0.734	0.722	0.771	0.795	0.721
**ULC082-bin3**	0.740	0.763	0.766	0.721	0.724	0.723	0.756	0.723
**ULC082-bin4**	0.758	0.759	0.786	0.686	0.000	0.702	0.786	0.696
**ULC082-bin5**	0.000	0.873	0.742	0.000	0.000	0.000	0.732	0.000
**ULC082-nobin**	0.766	0.761	0.808	0.726	0.730	0.752	0.783	0.724
**ULC084-bin1**	0.731	0.739	0.743	0.731	0.716	0.992	0.993	0.740
**ULC084-bin2**	0.749	0.755	0.912	0.726	0.722	0.706	0.769	0.719
**ULC084-bin3**	0.823	0.744	0.782	0.729	0.719	0.729	0.750	0.735
**ULC084-nobin**	0.783	0.758	0.918	0.726	0.725	0.957	0.944	0.726
**ULC129-bin1**	0.743	0.732	0.731	0.737	0.725	0.709	0.730	0.738
**ULC129-nobin**	0.747	0.759	0.909	0.737	0.743	0.981	0.979	0.737
**ULC146-bin1**	0.743	0.760	0.952	0.723	0.709	0.748	0.810	0.725
**ULC146-bin2**	0.710	0.707	0.000	0.735	0.731	0.000	0.731	0.710
**ULC146-bin3**	0.739	0.751	0.966	0.717	0.727	0.757	0.958	0.727
**ULC146-bin4**	0.727	0.736	0.744	0.715	0.721	0.993	0.995	0.724
**ULC146-bin5**	0.742	0.754	0.782	0.710	0.713	0.715	0.776	0.715
**ULC146-bin6**	0.744	0.748	0.780	0.717	0.710	0.713	0.775	0.724
**ULC146-bin7**	0.737	0.740	0.972	0.000	0.000	0.000	0.960	0.708
**ULC146-nobin**	0.746	0.759	0.888	0.743	0.736	0.891	0.873	0.744
**ULC165-bin1**	0.743	0.998	0.941	0.707	0.742	0.722	0.753	0.717
**ULC165-bin2**	0.746	0.755	0.909	0.730	0.708	0.729	0.772	0.710
**ULC165-bin3**	0.744	0.762	0.954	0.743	0.750	0.742	0.797	0.734
**ULC165-bin4**	0.735	0.733	0.768	0.749	0.727	0.698	0.731	0.741
**ULC165-nobin**	0.746	0.804	0.900	0.746	0.722	0.753	0.854	0.748
**ULC179-bin1**	0.741	0.755	0.780	0.710	0.704	0.732	0.774	0.708
**ULC179-bin2**	0.741	0.755	0.768	0.720	0.724	0.746	0.783	0.728
**ULC179-bin3**	0.734	0.755	0.768	0.725	0.729	0.724	0.769	0.722
**ULC179-bin4**	0.718	0.734	0.732	0.720	0.724	0.736	0.734	0.726
**ULC179-bin5**	0.741	0.754	0.777	0.697	0.000	0.747	0.776	0.717
**ULC179-bin6**	0.733	0.766	0.772	0.714	0.000	0.723	0.766	0.735
**ULC179-bin7**	0.000	0.000	0.750	0.000	0.000	0.000	0.754	0.000
**ULC179-nobin**	0.750	0.756	0.780	0.752	0.737	0.716	0.784	0.747
**ULC186-bin1**	0.756	0.736	0.743	0.739	0.713	0.732	0.737	0.739
**ULC186-nobin**	0.752	0.764	0.808	0.743	0.739	0.758	0.797	0.732
**ULC187-bin1**	0.732	0.709	0.729	0.799	0.734	0.731	0.739	0.797
**ULC187-nobin**	0.745	0.764	0.946	0.818	0.739	0.828	0.912	0.807

	ULC068-bin2	ULC068-nobin	ULC073-bin1	ULC073-bin2	ULC073-bin3	ULC073-bin4	ULC073-bin5	ULC073-bin6
**ULC335-bin1**	0.769	0.739	0.736	0.725	0.722	0.689	0.746	0.000
**ULC335-bin2**	0.000	0.782	0.750	0.716	0.708	0.701	0.000	0.000
**ULC335-bin3**	0.717	0.727	0.728	0.726	0.719	0.000	0.000	0.000
**ULC335-bin4**	0.000	0.795	0.753	0.733	0.773	0.770	0.000	0.774
**ULC335-nobin**	0.778	0.784	0.748	0.757	0.779	0.771	0.730	0.799
**ULC007-bin1**	0.739	0.739	0.752	0.714	0.724	0.729	0.751	0.000
**ULC007-bin2**	0.000	0.000	0.706	0.000	0.000	0.735	0.761	0.000
**ULC007-nobin**	0.756	0.833	0.886	0.998	0.928	0.000	0.741	0.801
**ULC027-bin1**	0.709	0.734	0.758	0.724	0.720	0.742	0.770	0.700
**ULC027-bin2**	0.000	0.895	0.729	0.736	0.756	0.753	0.724	0.818
**ULC027-bin3**	0.000	0.776	0.733	0.742	0.992	0.795	0.711	0.985
**ULC027-bin4**	0.000	0.774	0.730	0.730	0.988	0.870	0.000	0.858
**ULC027-nobin**	0.738	0.909	0.763	0.985	0.882	0.938	0.891	0.896
**ULC041-bin1**	0.736	0.746	0.985	0.726	0.734	0.738	0.968	0.749
**ULC041-bin2**	0.000	0.743	0.975	0.000	0.718	0.000	0.976	0.000
**ULC041-nobin**	0.000	0.844	0.955		0.724		0.951	0.000
**ULC065-bin1**	0.000	0.752	0.761	0.734	0.741	0.746	0.769	0.726
**ULC065-bin2**	0.000	0.977	0.741	0.742	0.752	0.754	0.000	0.000
**ULC065-nobin**	0.000	0.921	0.745	0.751	0.955	0.778	0.751	0.981
**ULC066-bin1**	0.827	0.817	0.737	0.727	0.718	0.000	0.740	0.686
**ULC066-bin2**	0.000	0.736	0.726	0.726	0.724	0.740	0.000	0.703
**ULC066-bin3**	0.000	0.743	0.728	0.994	0.733	0.776		0.000
**ULC066-nobin**	0.000	0.897	0.740	0.992	0.936	0.778	0.930	0.971
**ULC068-bin1**	0.844	0.836	0.737	0.719	0.726	0.720	0.769	0.000
**ULC068-bin2**		0.750	0.000	0.708	0.000	0.000	0.000	0.000
**ULC068-nobin**	0.835		0.742	0.753	0.772	0.763	0.724	0.832
**ULC073-bin1**	0.000	0.741		0.728	0.734	0.748	0.895	0.769
**ULC073-bin2**	0.708	0.752	0.728		0.726	0.749	0.717	0.000
**ULC073-bin3**	0.000	0.768	0.733	0.727		0.800	0.709	0.807
**ULC073-bin4**	0.000	0.765	0.746	0.749	0.801		0.000	0.000
**ULC073-bin5**	0.000	0.736	0.869	0.726	0.709	0.000		0.000
**ULC073-bin6**	0.000	0.834	0.769	0.000	0.816	0.000	0.000	
**ULC073-nobin**	0.738	0.908	0.861	0.780	0.796	0.828	0.952	0.818
**ULC077-bin1**	0.745	0.768	0.749	0.731	0.728	0.728	0.772	0.000
**ULC077-nobin**	0.727	0.961	0.752	0.989	0.900	0.803	0.973	0.960
**ULC082-bin1**	0.000	0.744	0.756	0.726	0.739	0.732	0.762	0.000
**ULC082-bin2**	0.746	0.777	0.744	0.742	0.759	0.752	0.000	0.782
**ULC082-bin3**	0.000	0.755	0.741	0.750	0.749	0.747	0.000	0.767
**ULC082-bin4**	0.000	0.785	0.730	0.736	0.766	0.745	0.000	0.781
**ULC082-bin5**	0.000	0.733	0.000	0.730	0.710	0.000	0.000	0.000
**ULC082-nobin**	0.774	0.784	0.743	0.753	0.760	0.764	0.770	0.801
**ULC084-bin1**	0.719	0.749	0.733	0.998	0.725	0.718	0.731	0.726
**ULC084-bin2**	0.000	0.885	0.736	0.727	0.756	0.752	0.000	0.720
**ULC084-bin3**	0.720	0.742	0.757	0.737	0.737	0.768	0.754	0.000
**ULC084-nobin**	0.788	0.798	0.740	0.973	0.984	0.790	0.977	0.985
**ULC129-bin1**	0.768	0.754	0.753	0.725	0.736	0.729	0.752	0.708
**ULC129-nobin**	0.744	0.911	0.797	0.991	0.900	0.868	0.921	0.962
**ULC146-bin1**	0.000	0.791	0.740	0.751	0.751	0.749	0.736	0.761
**ULC146-bin2**	0.000	0.775	0.683	0.729	0.714	0.000	0.000	0.692
**ULC146-bin3**	0.772	0.773	0.738	0.733	0.992	0.792	0.000	0.984
**ULC146-bin4**	0.708	0.758	0.727	0.999	0.726	0.749	0.911	0.000
**ULC146-bin5**	0.000	0.774	0.740	0.733	0.760	0.753	0.000	0.793
**ULC146-bin6**	0.724	0.758	0.739	0.759	0.820	0.841	0.000	0.772
**ULC146-bin7**	0.000	0.761	0.703	0.735	0.993	0.770	0.730	0.830
**ULC146-nobin**	0.744	0.896	0.744	0.870	0.843	0.919	0.944	0.928
**ULC165-bin1**	0.000	0.982	0.744	0.741	0.751	0.738	0.000	0.732
**ULC165-bin2**	0.000	0.889	0.734	0.734	0.760	0.753	0.691	0.760
**ULC165-bin3**	0.000	0.800	0.730	0.755	0.753	0.742	0.727	0.750
**ULC165-bin4**	0.708	0.758	0.755	0.734	0.000	0.000	0.774	0.000
**ULC165-nobin**	0.754	0.830	0.749	0.750	0.859	0.878	0.753	0.940
**ULC179-bin1**	0.000	0.774	0.744	0.737	0.761	0.758	0.734	0.754
**ULC179-bin2**	0.000	0.767	0.741	0.732	0.763	0.759	0.721	0.747
**ULC179-bin3**	0.755	0.767	0.738	0.731	0.777	0.775	0.000	0.794
**ULC179-bin4**	0.000	0.726	0.733	0.735	0.737	0.763	0.725	0.000
**ULC179-bin5**	0.000	0.773	0.733	0.749	0.760	0.763	0.000	0.791
**ULC179-bin6**	0.000	0.762	0.734	0.737	0.765	0.760	0.000	0.754
**ULC179-bin7**	0.000	0.783	0.000	0.725	0.743	0.789	0.000	0.777
**ULC179-nobin**	0.761	0.783	0.747	0.748	0.762	0.773	0.737	0.819
**ULC186-bin1**	0.718	0.742	0.843	0.728	0.729	0.746	0.848	0.749
**ULC186-nobin**	0.811	0.807	0.847	0.770	0.773	0.736	0.854	0.836
**ULC187-bin1**	0.877	0.784	0.737	0.727	0.728	0.705	0.741	0.000
**ULC187-nobin**	0.827	0.902	0.747	0.823	0.973	0.926	0.827	0.986

	ULC073-nobin	ULC077-bin1	ULC077-nobin	ULC082-bin1	ULC082-bin2	ULC082-bin3	ULC082-bin4	ULC082-bin5
**ULC335-bin1**	0.735	0.742	0.740	0.727	0.710	0.719	0.000	0.000
**ULC335-bin2**	0.684	0.720	0.725	0.000	0.739	0.713	0.718	0.000
**ULC335-bin3**	0.723	0.712	0.722	0.718	0.721	0.739	0.695	0.000
**ULC335-bin4**	0.771	0.724	0.791	0.755	0.866	0.761	0.905	0.695
**ULC335-nobin**	0.794	0.726	0.790	0.743	0.819	0.781	0.872	0.763
**ULC007-bin1**	0.752	0.759	0.762	0.741	0.723	0.725	0.717	0.000
**ULC007-bin2**	0.719	0.784	0.824	0.842	0.000	0.000	0.000	0.000
**ULC007-nobin**	0.887	0.856	0.874	0.807	0.933	0.000	0.000	0.000
**ULC027-bin1**	0.745	0.747	0.745	0.738	0.729	0.734	0.726	0.000
**ULC027-bin2**	0.757	0.723	0.767	0.737	0.755	0.750	0.766	0.710
**ULC027-bin3**	0.908	0.720	0.916	0.739	0.760	0.749	0.769	0.730
**ULC027-bin4**	0.942	0.727	0.897	0.731	0.756	0.738	0.747	0.000
**ULC027-nobin**	0.955	0.747	0.940	0.752	0.767	0.758	0.772	0.720
**ULC041-bin1**	0.951	0.750	0.749	0.756	0.740	0.737	0.757	0.704
**ULC041-bin2**	0.956	0.749	0.763	0.742	0.764	0.000	0.709	0.000
**ULC041-nobin**	0.973	0.778	0.862	0.770	0.000	0.723	0.000	0.000
**ULC065-bin1**	0.751	0.736	0.746	0.821	0.738	0.740	0.753	0.000
**ULC065-bin2**	0.753	0.727	0.991	0.744	0.747	0.760	0.753	0.698
**ULC065-nobin**	0.922	0.743	0.919	0.756	0.818	0.770	0.779	0.727
**ULC066-bin1**	0.768	0.747	0.799	0.733	0.725	0.722	0.689	0.000
**ULC066-bin2**	0.736	0.713	0.696	0.722	0.709	0.727	0.000	0.000
**ULC066-bin3**	0.867	0.708	0.990	0.736	0.767	0.725	0.702	0.000
**ULC066-nobin**	0.893	0.741	0.966	0.744	0.793	0.754	0.782	0.732
**ULC068-bin1**	0.730	0.744	0.754	0.732	0.721	0.725	0.696	0.000
**ULC068-bin2**	0.738	0.745	0.727	0.000	0.746	0.000	0.000	0.000
**ULC068-nobin**	0.914	0.738	0.965	0.747	0.779	0.759	0.787	0.733
**ULC073-bin1**	0.832	0.749	0.745	0.756	0.742	0.739	0.724	0.000
**ULC073-bin2**	0.762	0.733	0.993	0.726	0.740	0.749	0.736	0.734
**ULC073-bin3**	0.791	0.738	0.897	0.739	0.756	0.744	0.763	0.710
**ULC073-bin4**	0.827	0.725	0.805	0.742	0.756	0.747	0.745	0.000
**ULC073-bin5**	0.902	0.772	0.972	0.770	0.000	0.000	0.000	0.000
**ULC073-bin6**	0.810	0.000	0.966	0.000	0.760	0.767	0.781	0.000
**ULC073-nobin**		0.740	0.883	0.747	0.762	0.763	0.770	0.813
**ULC077-bin1**	0.739		0.928	0.738	0.733	0.734	0.720	0.000
**ULC077-nobin**	0.890	0.963		0.750	0.778	0.762	0.812	0.710
**ULC082-bin1**	0.743	0.738	0.750		0.750	0.734	0.733	
**ULC082-bin2**	0.760	0.734	0.775	0.746		0.744	0.761	0.000
**ULC082-bin3**	0.764	0.731	0.765	0.733	0.741		0.740	0.729
**ULC082-bin4**	0.764	0.729	0.817	0.735	0.760	0.740		0.000
**ULC082-bin5**	0.802	0.000	0.710	0.000	0.000	0.734	0.000	
**ULC082-nobin**	0.792	0.739	0.788	0.757	0.783	0.781	0.803	0.796
**ULC084-bin1**	0.871	0.734	0.993	0.724	0.734	0.745	0.725	0.742
**ULC084-bin2**	0.756	0.727	0.958	0.743	0.800	0.740	0.835	0.696
**ULC084-bin3**	0.756	0.738	0.746	0.986	0.756	0.736	0.754	0.000
**ULC084-nobin**	0.915	0.745	0.946	0.923	0.832	0.766	0.772	0.730
**ULC129-bin1**	0.752	0.744	0.745	0.743	0.726	0.734	0.693	0.000
**ULC129-nobin**	0.924	0.737	0.969	0.752	0.778	0.769	0.760	0.719
**ULC146-bin1**	0.950	0.726	0.782	0.745	0.743	0.785	0.745	0.738
**ULC146-bin2**	0.000	0.719	0.717	0.000	0.747	0.723	0.716	0.000
**ULC146-bin3**	0.926	0.713	0.904	0.737	0.753	0.752	0.759	0.726
**ULC146-bin4**	0.899	0.718	0.993	0.721	0.739	0.741	0.739	0.731
**ULC146-bin5**	0.763	0.723	0.792	0.737	0.827	0.744	0.841	0.730
**ULC146-bin6**	0.964	0.721	0.793	0.751	0.749	0.754	0.755	0.750
**ULC146-bin7**	0.933	0.723	0.923	0.735	0.747	0.746	0.760	0.000
**ULC146-nobin**	0.945	0.751	0.877	0.749	0.771	0.751	0.792	0.785
**ULC165-bin1**	0.750	0.726	0.993	0.738	0.740	0.759	0.756	0.730
**ULC165-bin2**	0.757	0.729	0.958	0.745	0.810	0.743	0.821	0.000
**ULC165-bin3**	0.951	0.727	0.783	0.740	0.747	0.809	0.741	0.733
**ULC165-bin4**	0.745	0.757	0.736	0.737	0.000	0.720	0.685	0.000
**ULC165-nobin**	0.900	0.758	0.822	0.747	0.764	0.764	0.767	0.728
**ULC179-bin1**	0.759	0.724	0.788	0.742	0.799	0.748	0.822	0.720
**ULC179-bin2**	0.768	0.729	0.767	0.741	0.757	0.741	0.766	0.731
**ULC179-bin3**	0.773	0.720	0.763	0.736	0.756	0.737	0.761	0.000
**ULC179-bin4**	0.734	0.732	0.742	0.728	0.726	0.744	0.736	0.000
**ULC179-bin5**	0.756	0.718	0.785	0.736	0.811	0.742	0.829	0.000
**ULC179-bin6**	0.765	0.722	0.759	0.722	0.758	0.739	0.745	0.000
**ULC179-bin7**	0.754	0.731	0.785	0.752	0.757	0.000	0.820	0.000
**ULC179-nobin**	0.778	0.744	0.787	0.746	0.763	0.758	0.778	0.724
**ULC186-bin1**	0.821	0.751	0.756	0.759	0.741	0.730	0.742	0.742
**ULC186-nobin**	0.826	0.746	0.813	0.769	0.761	0.800	0.767	0.000
**ULC187-bin1**	0.725	0.742	0.743	0.734	0.734	0.725	0.695	0.000
**ULC187-nobin**	0.938	0.741	0.898	0.751	0.786	0.754	0.805	0.000

	ULC082-nobin	ULC084-bin1	ULC084-bin2	ULC084-bin3	ULC084-nobin	ULC129-bin1	ULC129-nobin	ULC146-bin1
**ULC335-bin1**	0.716	0.717	0.724	0.728	0.717	0.736	0.744	0.701
**ULC335-bin2**	0.711	0.706	0.702	0.743	0.725	0.727	0.736	0.693
**ULC335-bin3**	0.734	0.718	0.711	0.713	0.721	0.714	0.735	0.729
**ULC335-bin4**	0.880	0.741	0.811	0.755	0.775	0.722	0.766	0.754
**ULC335-nobin**	0.841	0.756	0.795	0.745	0.795	0.733	0.795	0.841
**ULC007-bin1**	0.722	0.727	0.726	0.742	0.744	0.745	0.748	0.723
**ULC007-bin2**	0.698	0.690	0.000	0.761	0.000	0.762	0.727	0.000
**ULC007-nobin**	0.904	0.000	0.709	0.803	0.903	0.755	0.869	0.782
**ULC027-bin1**	0.725	0.730	0.730	0.742	0.750	0.788	0.817	0.739
**ULC027-bin2**	0.759	0.732	0.759	0.742	0.770	0.730	0.988	0.747
**ULC027-bin3**	0.762	0.729	0.761	0.740	0.988	0.730	0.911	0.752
**ULC027-bin4**	0.754	0.725	0.752	0.732	0.989	0.733	0.910	0.742
**ULC027-nobin**	0.802	0.984	0.765	0.752	0.869	0.798	0.935	0.876
**ULC041-bin1**	0.741	0.728	0.746	0.757	0.736	0.754	0.787	0.735
**ULC041-bin2**	0.762	0.000	0.742	0.729	0.711	0.753	0.790	0.716
**ULC041-nobin**	0.852		0.000	0.783	0.893	0.752	0.898	0.798
**ULC065-bin1**	0.774	0.731	0.750	0.823	0.786	0.742	0.740	0.744
**ULC065-bin2**	0.761	0.740	0.755	0.746	0.746	0.735	0.749	0.762
**ULC065-nobin**	0.812	0.745	0.903	0.772	0.913	0.734	0.903	0.953
**ULC066-bin1**	0.722	0.733	0.727	0.729	0.723	0.738	0.731	0.723
**ULC066-bin2**	0.722	0.715	0.722	0.719	0.724	0.725	0.740	0.709
**ULC066-bin3**	0.750	0.993	0.747	0.729	0.968	0.714	0.987	0.750
**ULC066-nobin**	0.785	0.990	0.773	0.748	0.936	0.731	0.972	0.809
**ULC068-bin1**	0.728	0.740	0.719	0.735	0.724	0.738	0.733	0.727
**ULC068-bin2**	0.774	0.719	0.000	0.720	0.788	0.768	0.744	0.000
**ULC068-nobin**	0.789	0.757	0.882	0.745	0.795	0.744	0.916	0.800
**ULC073-bin1**	0.742	0.733	0.736	0.757	0.738	0.752	0.797	0.738
**ULC073-bin2**	0.752	0.997	0.726	0.741	0.972	0.729	0.993	0.751
**ULC073-bin3**	0.759	0.726	0.756	0.739	0.981	0.737	0.904	0.753
**ULC073-bin4**	0.763	0.718	0.751	0.766	0.794	0.729	0.876	0.750
**ULC073-bin5**	0.777	0.731	0.000	0.760	0.976	0.754	0.901	0.736
**ULC073-bin6**	0.789	0.726	0.714	0.000	0.987	0.708	0.965	0.761
**ULC073-nobin**	0.797	0.858	0.760	0.754	0.897	0.749	0.918	0.953
**ULC077-bin1**	0.739	0.733	0.729	0.738	0.732	0.744	0.733	0.727
**ULC077-nobin**	0.794	0.988	0.955	0.751	0.950	0.746	0.967	0.788
**ULC082-bin1**	0.764	0.725	0.748	0.986	0.929	0.743	0.746	0.744
**ULC082-bin2**	0.783	0.739	0.801	0.755	0.826	0.727	0.774	0.746
**ULC082-bin3**	0.782	0.747	0.740	0.737	0.762	0.736	0.764	0.789
**ULC082-bin4**	0.805	0.725	0.837	0.758	0.773	0.693	0.767	0.746
**ULC082-bin5**	0.788	0.742	0.696	0.000	0.730	0.873	0.773	0.744
**ULC082-nobin**		0.752	0.807	0.841	0.812	0.730	0.792	0.840
**ULC084-bin1**	0.751		0.732	0.734	0.794	0.726	0.992	0.752
**ULC084-bin2**	0.808	0.731		0.745	0.769	0.738	0.760	0.745
**ULC084-bin3**	0.867	0.735	0.745		0.787	0.740	0.757	0.747
**ULC084-nobin**	0.824	0.787	0.772	0.791		0.741	0.917	0.788
**ULC129-bin1**	0.725	0.725	0.738	0.741	0.738		0.863	0.736
**ULC129-nobin**	0.799	0.989	0.765	0.755	0.922	0.899		0.943
**ULC146-bin1**	0.844	0.751	0.744	0.746	0.781	0.737	0.941	
**ULC146-bin2**	0.719	0.723	0.709	0.713	0.719	0.712	0.743	0.716
**ULC146-bin3**	0.763	0.734	0.756	0.744	0.988	0.733	0.914	0.753
**ULC146-bin4**	0.750	0.997	0.744	0.732	0.970	0.730	0.992	0.752
**ULC146-bin5**	0.836	0.731	0.807	0.745	0.774	0.722	0.760	0.749
**ULC146-bin6**	0.752	0.747	0.750	0.748	0.784	0.710	0.793	0.748
**ULC146-bin7**	0.753	0.723	0.766	0.740	0.988	0.000	0.923	0.765
**ULC146-nobin**	0.837	0.848	0.781	0.747	0.850	0.749	0.891	0.769
**ULC165-bin1**	0.759	0.740	0.753	0.740	0.748	0.723	0.749	0.760
**ULC165-bin2**	0.810	0.731	0.998	0.740	0.892	0.731	0.756	0.751
**ULC165-bin3**	0.846	0.750	0.746	0.735	0.789	0.735	0.935	0.996
**ULC165-bin4**	0.785	0.723	0.728	0.733	0.754	0.751	0.764	0.718
**ULC165-nobin**	0.838	0.750	0.804	0.745	0.870	0.749	0.880	0.985
**ULC179-bin1**	0.817	0.732	0.805	0.751	0.772	0.731	0.761	0.748
**ULC179-bin2**	0.822	0.730	0.764	0.740	0.771	0.728	0.767	0.750
**ULC179-bin3**	0.759	0.729	0.760	0.742	0.776	0.731	0.769	0.751
**ULC179-bin4**	0.739	0.729	0.731	0.728	0.739	0.732	0.740	0.739
**ULC179-bin5**	0.820	0.734	0.807	0.744	0.761	0.717	0.759	0.753
**ULC179-bin6**	0.762	0.729	0.752	0.725	0.778	0.727	0.764	0.747
**ULC179-bin7**	0.777	0.691	0.780	0.000	0.787	0.728	0.728	0.750
**ULC179-nobin**	0.789	0.745	0.777	0.748	0.796	0.738	0.780	0.771
**ULC186-bin1**	0.742	0.738	0.734	0.756	0.759	0.755	0.755	0.737
**ULC186-nobin**	0.810	0.772	0.757	0.765	0.839	0.752	0.827	0.816
**ULC187-bin1**	0.765	0.727	0.724	0.731	0.766	0.735	0.741	0.730
**ULC187-nobin**	0.805	0.820	0.954	0.751	0.958	0.749	0.899	0.765

	ULC146-bin2	ULC146-bin3	ULC146-bin4	ULC146-bin5	ULC146-bin6	ULC146-bin7	ULC146-nobin	ULC165-bin1
**ULC335-bin1**	0.734	0.717	0.716	0.704	0.000	0.000	0.744	0.704
**ULC335-bin2**	0.773	0.000	0.717	0.709	0.000	0.000	0.762	0.725
**ULC335-bin3**	0.727	0.716	0.730	0.722	0.000	0.747	0.724	0.724
**ULC335-bin4**	0.738	0.775	0.742	0.854	0.772	0.746	0.817	0.768
**ULC335-nobin**	0.733	0.783	0.754	0.838	0.771	0.779	0.802	0.763
**ULC007-bin1**	0.725	0.717	0.719	0.721	0.737	0.726	0.745	0.715
**ULC007-bin2**	0.000	0.000	0.000	0.000	0.000	0.000	0.758	0.695
**ULC007-nobin**	0.000	0.822		0.722	0.000	0.000	0.833	0.000
**ULC027-bin1**	0.719	0.727	0.723	0.726	0.730	0.000	0.736	0.723
**ULC027-bin2**	0.763	0.757	0.730	0.766	0.753	0.758	0.761	0.749
**ULC027-bin3**	0.717	0.997	0.733	0.767	0.828	0.991	0.810	0.754
**ULC027-bin4**	0.000	0.995	0.723	0.734	0.778	0.995	0.806	0.751
**ULC027-nobin**	0.728	0.861	0.985	0.774	0.959	0.875	0.951	0.756
**ULC041-bin1**	0.701	0.736	0.738	0.732	0.738	0.000	0.744	0.745
**ULC041-bin2**	0.000	0.723	0.702	0.740	0.000	0.685	0.721	0.756
**ULC041-nobin**	0.000		0.000	0.722	0.000	0.719	0.823	0.753
**ULC065-bin1**	0.710	0.741	0.724	0.741	0.742	0.737	0.742	0.745
**ULC065-bin2**	0.707	0.752	0.736	0.752	0.745	0.740	0.755	0.998
**ULC065-nobin**	0.729	0.958	0.750	0.785	0.778	0.960	0.888	0.926
**ULC066-bin1**	0.735	0.717	0.715	0.701	0.717	0.000	0.739	0.707
**ULC066-bin2**	0.732	0.727	0.722	0.713	0.710	0.000	0.729	0.742
**ULC066-bin3**	0.000	0.757	0.994	0.748	0.713	0.000	0.914	0.725
**ULC066-nobin**	0.732	0.947	0.991	0.776	0.777	0.948	0.872	0.754
**ULC068-bin1**	0.710	0.727	0.723	0.713	0.724	0.708	0.740	0.717
**ULC068-bin2**	0.000	0.772	0.708	0.000	0.724	0.000	0.744	0.000
**ULC068-nobin**	0.779	0.774	0.756	0.780	0.764	0.767	0.908	0.981
**ULC073-bin1**	0.683	0.737	0.726	0.736	0.743	0.700	0.742	0.743
**ULC073-bin2**	0.729	0.733	0.998	0.729	0.759	0.732	0.887	0.740
**ULC073-bin3**	0.714	0.990	0.728	0.756	0.818	0.991	0.845	0.752
**ULC073-bin4**	0.000	0.796	0.749	0.754	0.841	0.784	0.925	0.745
**ULC073-bin5**	0.000	0.000	0.732	0.000	0.000	0.730	0.966	0.000
**ULC073-bin6**	0.000	0.990	0.000	0.765	0.781	0.849	0.943	0.732
**ULC073-nobin**	0.828	0.907	0.886	0.763	0.951	0.911	0.942	0.754
**ULC077-bin1**	0.719	0.713	0.721	0.718	0.721	0.723	0.746	0.727
**ULC077-nobin**	0.717	0.905	0.991	0.790	0.800	0.907	0.888	0.988
**ULC082-bin1**	0.000	0.741	0.720	0.733	0.751	0.732	0.752	0.741
**ULC082-bin2**	0.747	0.758	0.739	0.826	0.751	0.749	0.768	0.745
**ULC082-bin3**	0.724	0.754	0.743	0.745	0.753	0.746	0.757	0.761
**ULC082-bin4**	0.716	0.765	0.744	0.845	0.755	0.760	0.799	0.761
**ULC082-bin5**	0.000	0.726	0.728	0.731	0.744	0.000	0.845	0.766
**ULC082-nobin**	0.716	0.763	0.752	0.826	0.753	0.756	0.832	0.759
**ULC084-bin1**	0.723	0.734	0.997	0.730	0.747	0.723	0.867	0.741
**ULC084-bin2**	0.709	0.757	0.744	0.806	0.747	0.763	0.782	0.753
**ULC084-bin3**	0.713	0.747	0.733	0.745	0.748	0.738	0.747	0.743
**ULC084-nobin**	0.716	0.988	0.969	0.776	0.790	0.984	0.862	0.756
**ULC129-bin1**	0.712	0.733	0.731	0.721	0.710	0.694	0.743	0.724
**ULC129-nobin**	0.748	0.909	0.989	0.766	0.792	0.917	0.901	0.756
**ULC146-bin1**	0.716	0.750	0.752	0.745	0.744	0.765	0.773	0.759
**ULC146-bin2**		0.715	0.707	0.712	0.000	0.000	0.802	0.705
**ULC146-bin3**	0.715		0.737	0.759	0.788	0.797	0.786	0.750
**ULC146-bin4**	0.707	0.738		0.734	0.736	0.715	0.790	0.742
**ULC146-bin5**	0.712	0.762	0.737		0.757	0.760	0.774	0.759
**ULC146-bin6**	0.000	0.791	0.736	0.758		0.765	0.810	0.743
**ULC146-bin7**	0.000	0.825	0.715	0.763	0.765		0.780	0.753
**ULC146-nobin**	0.835	0.788	0.795	0.777	0.815	0.779		0.756
**ULC165-bin1**	0.705	0.751	0.743	0.757	0.738	0.749	0.754	
**ULC165-bin2**	0.734	0.756	0.731	0.804	0.750	0.759	0.785	0.748
**ULC165-bin3**	0.699	0.748	0.752	0.750	0.751	0.763	0.795	0.760
**ULC165-bin4**	0.714	0.728	0.740	0.000	0.732	0.000	0.797	0.735
**ULC165-nobin**	0.747	0.849	0.751	0.773	0.886	0.839	0.873	0.774
**ULC179-bin1**	0.719	0.758	0.731	0.820	0.757	0.756	0.795	0.754
**ULC179-bin2**	0.706	0.764	0.734	0.774	0.760	0.754	0.907	0.749
**ULC179-bin3**	0.716	0.774	0.733	0.760	0.769	0.764	0.773	0.754
**ULC179-bin4**	0.724	0.732	0.727	0.730	0.719	0.752	0.741	0.730
**ULC179-bin5**	0.732	0.754	0.742	0.826	0.750	0.751	0.802	0.747
**ULC179-bin6**	0.000	0.760	0.739	0.762	0.746	0.744	0.843	0.763
**ULC179-bin7**	0.000	0.752	0.000	0.759	0.740	0.768	0.824	0.721
**ULC179-nobin**	0.728	0.770	0.747	0.783	0.769	0.751	0.860	0.758
**ULC186-bin1**	0.717	0.732	0.732	0.742	0.740	0.717	0.746	0.738
**ULC186-nobin**	0.751	0.777	0.765	0.765	0.805	0.760	0.824	0.768
**ULC187-bin1**	0.721	0.724	0.727	0.704	0.730	0.704	0.741	0.729
**ULC187-nobin**	0.759	0.975	0.819	0.800	0.836	0.980	0.900	0.762

	ULC165-bin2	ULC165-bin3	ULC165-bin4	ULC165-nobin	ULC179-bin1	ULC179-bin2	ULC179-bin3	ULC179-bin4
**ULC335-bin1**	0.722	0.000	0.747	0.742	0.715	0.706	0.714	0.725
**ULC335-bin2**	0.703	0.000	0.709	0.766	0.717	0.000	0.700	0.726
**ULC335-bin3**	0.710	0.749	0.717	0.726	0.721	0.719	0.713	0.740
**ULC335-bin4**	0.813	0.754	0.690	0.781	0.832	0.786	0.770	0.749
**ULC335-nobin**	0.796	0.840	0.740	0.822	0.816	0.778	0.766	0.741
**ULC007-bin1**	0.725	0.728	0.755	0.755	0.724	0.721	0.728	0.723
**ULC007-bin2**	0.000	0.000	0.776	0.777	0.000	0.000	0.000	0.000
**ULC007-nobin**	0.692	0.000	0.808	0.793	0.000	0.737	0.815	0.000
**ULC027-bin1**	0.733	0.723	0.743	0.749	0.719	0.730	0.729	0.720
**ULC027-bin2**	0.757	0.755	0.701	0.762	0.761	0.765	0.760	0.730
**ULC027-bin3**	0.760	0.753	0.717	0.865	0.766	0.765	0.776	0.739
**ULC027-bin4**	0.753	0.745	0.000	0.836	0.747	0.765	0.773	0.734
**ULC027-nobin**	0.766	0.865	0.761	0.876	0.777	0.815	0.775	0.738
**ULC041-bin1**	0.738	0.741	0.746	0.747	0.746	0.739	0.729	0.736
**ULC041-bin2**	0.740	0.760	0.751	0.778	0.737	0.730	0.746	0.727
**ULC041-nobin**	0.000	0.835	0.000	0.818	0.720	0.721	0.799	0.000
**ULC065-bin1**	0.746	0.742	0.731	0.744	0.743	0.742	0.734	0.719
**ULC065-bin2**	0.755	0.762	0.733	0.810	0.756	0.755	0.756	0.735
**ULC065-nobin**	0.897	0.944	0.765	0.907	0.780	0.774	0.774	0.739
**ULC066-bin1**	0.730	0.728	0.745	0.744	0.711	0.720	0.723	0.718
**ULC066-bin2**	0.708	0.750	0.727	0.713	0.704	0.724	0.742	0.726
**ULC066-bin3**	0.744	0.744	0.698	0.747	0.744	0.753	0.730	0.736
**ULC066-nobin**	0.773	0.797	0.724	0.849	0.778	0.788	0.775	0.739
**ULC068-bin1**	0.710	0.734	0.738	0.746	0.711	0.725	0.720	0.726
**ULC068-bin2**	0.000	0.000	0.708	0.763	0.000	0.000	0.755	0.000
**ULC068-nobin**	0.881	0.803	0.742	0.830	0.782	0.776	0.772	0.733
**ULC073-bin1**	0.735	0.727	0.753	0.749	0.742	0.741	0.737	0.733
**ULC073-bin2**	0.734	0.755	0.740	0.751	0.736	0.731	0.733	0.737
**ULC073-bin3**	0.759	0.751	0.000	0.865	0.761	0.765	0.779	0.739
**ULC073-bin4**	0.754	0.742	0.000	0.889	0.760	0.765	0.774	0.763
**ULC073-bin5**	0.000	0.727	0.770	0.750	0.734	0.721	0.000	0.730
**ULC073-bin6**	0.760	0.750	0.000	0.948	0.756	0.747	0.791	0.000
**ULC073-nobin**	0.761	0.953	0.757	0.902	0.762	0.770	0.775	0.737
**ULC077-bin1**	0.728	0.725	0.753	0.754	0.726	0.732	0.720	0.732
**ULC077-nobin**	0.956	0.785	0.745	0.820	0.792	0.770	0.769	0.740
**ULC082-bin1**	0.745	0.738	0.736	0.747	0.740	0.743	0.734	0.728
**ULC082-bin2**	0.809	0.749	0.000	0.762	0.802	0.762	0.758	0.724
**ULC082-bin3**	0.745	0.809	0.706	0.766	0.749	0.744	0.740	0.745
**ULC082-bin4**	0.823	0.741	0.685	0.768	0.827	0.772	0.766	0.752
**ULC082-bin5**	0.000	0.735	0.000	0.768	0.725	0.731	0.000	0.873
**ULC082-nobin**	0.808	0.837	0.782	0.833	0.816	0.814	0.761	0.742
**ULC084-bin1**	0.731	0.750	0.723	0.750	0.729	0.732	0.728	0.729
**ULC084-bin2**	0.998	0.743	0.696	0.810	0.808	0.764	0.760	0.733
**ULC084-bin3**	0.739	0.731	0.733	0.744	0.750	0.742	0.741	0.729
**ULC084-nobin**	0.888	0.794	0.754	0.878	0.777	0.778	0.782	0.745
**ULC129-bin1**	0.730	0.732	0.748	0.746	0.737	0.724	0.729	0.732
**ULC129-nobin**	0.764	0.935	0.762	0.891	0.768	0.776	0.775	0.745
**ULC146-bin1**	0.750	0.992	0.715	0.989	0.747	0.750	0.750	0.737
**ULC146-bin2**	0.734	0.699	0.714	0.747	0.717	0.706	0.716	0.723
**ULC146-bin3**	0.756	0.746	0.728	0.854	0.758	0.764	0.775	0.729
**ULC146-bin4**	0.731	0.751	0.730	0.751	0.732	0.733	0.733	0.727
**ULC146-bin5**	0.805	0.750	0.000	0.777	0.825	0.776	0.763	0.730
**ULC146-bin6**	0.750	0.743	0.000	0.891	0.760	0.762	0.773	0.719
**ULC146-bin7**	0.761	0.763	0.000	0.853	0.757	0.760	0.767	0.752
**ULC146-nobin**	0.783	0.781	0.790	0.869	0.793	0.900	0.778	0.749
**ULC165-bin1**	0.748	0.757	0.735	0.761	0.753	0.750	0.754	0.729
**ULC165-bin2**		0.746	0.735	0.767	0.807	0.764	0.758	0.736
**ULC165-bin3**	0.748		0.714	0.778	0.748	0.749	0.755	0.728
**ULC165-bin4**	0.743	0.714		0.801	0.764	0.738	0.738	0.726
**ULC165-nobin**	0.767	0.776	0.828		0.769	0.813	0.781	0.733
**ULC179-bin1**	0.805	0.747	0.764	0.767		0.766	0.759	0.738
**ULC179-bin2**	0.765	0.747	0.719	0.814	0.768		0.761	0.729
**ULC179-bin3**	0.757	0.751	0.738	0.777	0.760	0.762		0.736
**ULC179-bin4**	0.736	0.728	0.726	0.736	0.740	0.729	0.736	
**ULC179-bin5**	0.798	0.746	0.000	0.766	0.815	0.773	0.762	0.742
**ULC179-bin6**	0.752	0.752	0.000	0.763	0.768	0.769	0.758	0.745
**ULC179-bin7**	0.762	0.774	0.000	0.771	0.768	0.745	0.845	0.750
**ULC179-nobin**	0.781	0.763	0.750	0.790	0.777	0.785	0.787	0.808
**ULC186-bin1**	0.737	0.740	0.751	0.750	0.739	0.735	0.736	0.740
**ULC186-nobin**	0.764	0.811	0.752	0.822	0.759	0.771	0.765	0.747
**ULC187-bin1**	0.723	0.731	0.743	0.739	0.723	0.718	0.716	0.726
**ULC187-nobin**	0.953	0.762	0.755	0.864	0.796	0.784	0.786	0.738

	ULC179-bin5	ULC179-bin6	ULC179-bin7	ULC179-nobin	ULC186-bin1	ULC186-nobin	ULC187-bin1	ULC187-nobin
**ULC335-bin1**	0.693	0.000	0.000	0.746	0.738	0.861	0.748	0.734
**ULC335-bin2**	0.000	0.000	0.000	0.722	0.723	0.749	0.730	0.718
**ULC335-bin3**	0.717	0.725	0.000	0.733	0.726	0.725	0.717	0.712
**ULC335-bin4**	0.835	0.765	0.746	0.789	0.750	0.769	0.718	0.804
**ULC335-nobin**	0.811	0.759	0.760	0.778	0.746	0.885	0.743	0.790
**ULC007-bin1**	0.727	0.718	0.000	0.743	0.750	0.749	0.741	0.759
**ULC007-bin2**	0.000	0.000	0.000	0.774	0.774	0.786	0.701	0.000
**ULC007-nobin**	0.000	0.000	0.000	0.835	0.791	0.906	0.788	0.892
**ULC027-bin1**	0.726	0.740	0.703	0.743	0.758	0.748	0.744	0.761
**ULC027-bin2**	0.762	0.756	0.761	0.758	0.742	0.759	0.725	0.771
**ULC027-bin3**	0.763	0.769	0.766	0.763	0.727	0.754	0.723	0.977
**ULC027-bin4**	0.753	0.759	0.000	0.754	0.731	0.737	0.000	0.970
**ULC027-nobin**	0.779	0.772	0.755	0.781	0.747	0.785	0.739	0.912
**ULC041-bin1**	0.731	0.725	0.000	0.742	0.842	0.838	0.737	0.743
**ULC041-bin2**	0.000	0.000	0.000	0.746	0.839	0.851	0.739	0.727
**ULC041-nobin**	0.000	0.710	0.000	0.839	0.862	0.881	0.771	0.879
**ULC065-bin1**	0.738	0.735	0.000	0.746	0.756	0.750	0.732	0.742
**ULC065-bin2**	0.752	0.763	0.000	0.751	0.735	0.762	0.706	0.758
**ULC065-nobin**	0.775	0.768	0.759	0.778	0.744	0.804	0.730	0.932
**ULC066-bin1**	0.697	0.714	0.000	0.742	0.738	0.743	0.798	0.798
**ULC066-bin2**	0.000	0.000	0.000	0.733	0.713	0.734	0.734	0.747
**ULC066-bin3**	0.756	0.723	0.000	0.715	0.732	0.740	0.727	0.825
**ULC066-nobin**	0.776	0.773	0.752	0.780	0.739	0.793	0.746	0.899
**ULC068-bin1**	0.717	0.731	0.000	0.741	0.737	0.732	0.797	0.804
**ULC068-bin2**	0.000	0.000	0.000	0.749	0.718	0.811	0.886	0.833
**ULC068-nobin**	0.778	0.767	0.796	0.783	0.745	0.808	0.786	0.900
**ULC073-bin1**	0.732	0.734	0.000	0.744	0.843	0.841	0.737	0.742
**ULC073-bin2**	0.749	0.737	0.725	0.742	0.731	0.766	0.728	0.819
**ULC073-bin3**	0.760	0.763	0.741	0.758	0.729	0.755	0.730	0.970
**ULC073-bin4**	0.761	0.760	0.789	0.774	0.746	0.713	0.705	0.925
**ULC073-bin5**	0.000	0.000	0.000	0.731	0.852	0.829	0.741	0.000
**ULC073-bin6**	0.791	0.754	0.777	0.795	0.749	0.836	0.000	0.991
**ULC073-nobin**	0.759	0.764	0.753	0.775	0.824	0.815	0.727	0.920
**ULC077-bin1**	0.716	0.716	0.731	0.739	0.751	0.748	0.742	0.737
**ULC077-nobin**	0.786	0.766	0.774	0.784	0.755	0.802	0.742	0.894
**ULC082-bin1**	0.736	0.722	0.752	0.748	0.758	0.785	0.734	0.757
**ULC082-bin2**	0.811	0.756	0.757	0.758	0.740	0.762	0.737	0.777
**ULC082-bin3**	0.745	0.742	0.000	0.750	0.731	0.789	0.723	0.751
**ULC082-bin4**	0.834	0.745	0.820	0.778	0.734	0.727	0.695	0.801
**ULC082-bin5**	0.000	0.000	0.000	0.730	0.742	0.000	0.000	0.000
**ULC082-nobin**	0.815	0.759	0.779	0.782	0.744	0.800	0.762	0.798
**ULC084-bin1**	0.734	0.729	0.691	0.739	0.739	0.768	0.729	0.816
**ULC084-bin2**	0.806	0.749	0.778	0.772	0.734	0.753	0.724	0.956
**ULC084-bin3**	0.744	0.725	0.000	0.745	0.756	0.763	0.731	0.745
**ULC084-nobin**	0.767	0.785	0.774	0.794	0.756	0.831	0.763	0.955
**ULC129-bin1**	0.715	0.727	0.728	0.736	0.755	0.748	0.735	0.746
**ULC129-nobin**	0.770	0.767	0.760	0.779	0.757	0.808	0.744	0.892
**ULC146-bin1**	0.748	0.744	0.750	0.765	0.736	0.806	0.729	0.756
**ULC146-bin2**	0.732	0.000	0.000	0.728	0.717	0.738	0.722	0.764
**ULC146-bin3**	0.754	0.760	0.742	0.764	0.730	0.779	0.726	0.973
**ULC146-bin4**	0.740	0.739	0.000	0.745	0.731	0.758	0.725	0.817
**ULC146-bin5**	0.827	0.763	0.759	0.781	0.747	0.768	0.706	0.795
**ULC146-bin6**	0.751	0.746	0.728	0.766	0.736	0.774	0.742	0.834
**ULC146-bin7**	0.747	0.744	0.768	0.747	0.726	0.759	0.711	0.974
**ULC146-nobin**	0.803	0.830	0.812	0.853	0.746	0.812	0.748	0.891
**ULC165-bin1**	0.746	0.763	0.721	0.754	0.738	0.751	0.729	0.754
**ULC165-bin2**	0.797	0.753	0.762	0.779	0.737	0.762	0.722	0.955
**ULC165-bin3**	0.744	0.751	0.758	0.764	0.740	0.803	0.731	0.757
**ULC165-bin4**	0.000	0.000	0.000	0.745	0.754	0.743	0.745	0.723
**ULC165-nobin**	0.770	0.773	0.771	0.784	0.751	0.805	0.742	0.852
**ULC179-bin1**	0.814	0.765	0.756	0.771	0.738	0.757	0.720	0.791
**ULC179-bin2**	0.774	0.765	0.735	0.779	0.732	0.764	0.717	0.775
**ULC179-bin3**	0.760	0.760	0.846	0.773	0.736	0.756	0.716	0.779
**ULC179-bin4**	0.742	0.729	0.750	0.782	0.741	0.746	0.726	0.740
**ULC179-bin5**		0.766	0.765	0.778	0.723	0.753	0.711	0.789
**ULC179-bin6**	0.768		0.768	0.785	0.741	0.765	0.705	0.773
**ULC179-bin7**	0.767	0.768		0.815	0.000	0.000	0.757	0.730
**ULC179-nobin**	0.778	0.786	0.833		0.741	0.798	0.744	0.790
**ULC186-bin1**	0.725	0.736	0.000	0.742		0.879	0.741	0.797
**ULC186-nobin**	0.756	0.770	0.000	0.792	0.923		0.766	0.841
**ULC187-bin1**	0.708	0.705	0.757	0.738	0.741	0.761		0.870
**ULC187-nobin**	0.793	0.787	0.734	0.790	0.785	0.830	0.889	

	ULC335-bin1	ULC335-bin2	ULC335-bin3	ULC335-bin4	ULC335-nobin	ULC007-bin1	ULC007-bin2	ULC007-nobin
**ULC335-bin1**		0.002	0.000	0.000	0.008	0.040	0.000	0.001
**ULC335-bin2**	0.001		0.004		0.004	0.000		
**ULC335-bin3**	0.000	0.004		0.000	0.001	0.001		
**ULC335-bin4**	0.000		0.001		0.027	0.003		
**ULC335-nobin**	0.002	0.002	0.000	0.005		0.001	0.000	0.001
**ULC007-bin1**	0.035	0.000	0.001	0.001	0.002		0.001	0.003
**ULC007-bin2**	0.004					0.015		0.008
**ULC007-nobin**	0.012				0.018	0.071	0.016	
**ULC027-bin1**	0.023	0.000	0.000	0.000	0.003	0.039	0.001	0.000
**ULC027-bin2**	0.000	0.000	0.001	0.015	0.031	0.001		0.000
**ULC027-bin3**	0.000	0.000	0.000	0.010	0.046	0.001		0.000
**ULC027-bin4**			0.000	0.007	0.042	0.001		
**ULC027-nobin**	0.001	0.000	0.000	0.007	0.032	0.002	0.000	0.001
**ULC041-bin1**	0.023	0.000	0.001	0.001	0.005	0.044	0.000	0.001
**ULC041-bin2**	0.006		0.000	0.000	0.002	0.019	0.004	0.000
**ULC041-nobin**	0.004				0.010	0.013		0.024
**ULC065-bin1**	0.007		0.001	0.002	0.010	0.017	0.000	0.000
**ULC065-bin2**	0.000	0.000	0.001	0.006	0.028	0.001	0.000	
**ULC065-nobin**	0.000	0.000	0.000	0.010	0.059	0.001	0.000	0.001
**ULC066-bin1**	0.026	0.000	0.001	0.000	0.001	0.030	0.001	0.001
**ULC066-bin2**	0.001	0.011	0.033	0.000	0.014	0.000		0.000
**ULC066-bin3**	0.001		0.001	0.002	0.030	0.001		
**ULC066-nobin**	0.001	0.001	0.002	0.008	0.034	0.001	0.000	0.001
**ULC068-bin1**	0.028	0.000	0.001	0.000	0.001	0.036	0.001	0.001
**ULC068-bin2**	0.007		0.001		0.006	0.005		0.006
**ULC068-nobin**	0.001	0.008	0.001	0.012	0.042	0.002		0.001
**ULC073-bin1**	0.023	0.000	0.001	0.001	0.006	0.041	0.000	0.001
**ULC073-bin2**	0.001	0.001	0.001	0.001	0.025	0.001		0.000
**ULC073-bin3**	0.000	0.000	0.000	0.011	0.049	0.001		0.000
**ULC073-bin4**		0.000		0.010	0.035	0.001	0.000	
**ULC073-bin5**	0.011				0.002	0.023	0.006	0.001
**ULC073-bin6**				0.001	0.014			0.003
**ULC073-nobin**	0.001	0.000	0.000	0.008	0.042	0.002	0.000	0.001
**ULC077-bin1**	0.035	0.000	0.000	0.000	0.002	0.078	0.002	0.002
**ULC077-nobin**	0.002	0.000	0.001	0.008	0.034	0.003	0.002	0.005
**ULC082-bin1**	0.010		0.001	0.003	0.009	0.019	0.001	0.001
**ULC082-bin2**	0.001	0.000	0.001	0.028	0.086	0.001		
**ULC082-bin3**	0.001	0.000	0.001	0.004	0.029	0.002		
**ULC082-bin4**		0.001		0.117	0.206	0.002		
**ULC082-bin5**				0.001	0.002			
**ULC082-nobin**	0.000	0.000	0.000	0.024	0.087	0.001	0.000	0.001
**ULC084-bin1**	0.002	0.000	0.001	0.002	0.026	0.001	0.000	
**ULC084-bin2**	0.000	0.000	0.001	0.060	0.108	0.001		0.000
**ULC084-bin3**	0.009	0.000	0.000	0.002	0.008	0.015	0.001	0.001
**ULC084-nobin**	0.000	0.000	0.001	0.007	0.052	0.001	0.000	0.001
**ULC129-bin1**	0.023	0.000	0.000	0.000	0.003	0.035	0.001	0.001
**ULC129-nobin**	0.001	0.002	0.002	0.005	0.029	0.002	0.000	0.001
**ULC146-bin1**	0.000	0.000	0.001	0.005	0.140	0.001		0.000
**ULC146-bin2**	0.000	0.097	0.004	0.000	0.003	0.000		
**ULC146-bin3**	0.000		0.000	0.011	0.050	0.001		0.000
**ULC146-bin4**	0.001	0.000	0.001	0.001	0.024	0.001		
**ULC146-bin5**	0.000	0.000	0.001	0.097	0.156	0.001		0.000
**ULC146-bin6**				0.008	0.033	0.000		
**ULC146-bin7**			0.000	0.011	0.057	0.000		
**ULC146-nobin**	0.006	0.002	0.001	0.009	0.034	0.009	0.000	0.001
**ULC165-bin1**	0.000	0.000	0.001	0.007	0.030	0.001	0.000	
**ULC165-bin2**	0.000	0.000	0.000	0.059	0.108	0.001		0.000
**ULC165-bin3**			0.001	0.005	0.143	0.001		
**ULC165-bin4**	0.034	0.000	0.000	0.000	0.001	0.071	0.002	0.001
**ULC165-nobin**	0.010	0.000	0.000	0.005	0.047	0.021	0.001	0.001
**ULC179-bin1**	0.000	0.000	0.000	0.058	0.114	0.001		
**ULC179-bin2**	0.000		0.000	0.009	0.033	0.001		0.000
**ULC179-bin3**	0.000	0.000	0.000	0.011	0.039	0.001		0.000
**ULC179-bin4**	0.000	0.004	0.004	0.001	0.004	0.001	0.000	
**ULC179-bin5**	0.000		0.000	0.050	0.113	0.001		
**ULC179-bin6**			0.001	0.013	0.032	0.001		
**ULC179-bin7**				0.002	0.009			
**ULC179-nobin**	0.003	0.001	0.001	0.005	0.020	0.005	0.000	0.001
**ULC186-bin1**	0.022	0.000	0.000	0.001	0.005	0.042	0.001	0.001
**ULC186-nobin**	0.003	0.006	0.002	0.002	0.048	0.007	0.001	0.002
**ULC187-bin1**	0.031	0.001	0.001	0.000	0.001	0.031	0.000	0.001
**ULC187-nobin**	0.000	0.000	0.001	0.015	0.047	0.001	0.000	0.002

	ULC027-bin1	ULC027-bin2	ULC027-bin3	ULC027-bin4	ULC027-nobin	ULC041-bin1	ULC041-bin2	ULC041-nobin
**ULC335-bin1**	0.024	0.000	0.000		0.002	0.023	0.001	0.000
**ULC335-bin2**	0.000	0.000			0.001	0.000		
**ULC335-bin3**	0.001	0.001	0.000	0.000	0.001	0.001	0.000	
**ULC335-bin4**	0.001	0.050	0.026	0.006	0.041	0.003	0.000	
**ULC335-nobin**	0.001	0.016	0.018	0.006	0.032	0.003	0.000	0.001
**ULC007-bin1**	0.036	0.001	0.001	0.000	0.003	0.039	0.002	0.001
**ULC007-bin2**	0.009				0.003	0.005	0.008	
**ULC007-nobin**	0.004	0.004	0.001		0.028	0.021	0.001	0.034
**ULC027-bin1**		0.001	0.001	0.000	0.009	0.054	0.003	0.001
**ULC027-bin2**	0.001		0.016	0.004	0.027	0.002	0.000	0.000
**ULC027-bin3**	0.001	0.021		0.002	0.076	0.003	0.000	0.000
**ULC027-bin4**	0.000	0.017	0.004		0.062	0.002		
**ULC027-nobin**	0.006	0.014	0.029	0.008		0.004	0.001	0.001
**ULC041-bin1**	0.055	0.002	0.002	0.000	0.009		0.003	0.003
**ULC041-bin2**	0.026	0.002	0.000		0.016	0.017		0.007
**ULC041-nobin**	0.017	0.005	0.002		0.012	0.074	0.037	
**ULC065-bin1**	0.017	0.003	0.003	0.001	0.007	0.032	0.001	0.000
**ULC065-bin2**	0.001	0.012	0.011	0.003	0.019	0.005	0.000	0.000
**ULC065-nobin**	0.001	0.017	0.068	0.017	0.100	0.004	0.001	0.001
**ULC066-bin1**	0.018	0.001	0.000		0.002	0.014	0.001	0.001
**ULC066-bin2**	0.001	0.000	0.000		0.001	0.000		
**ULC066-bin3**	0.002	0.002	0.005		0.116	0.004		
**ULC066-nobin**	0.001	0.115	0.045	0.011	0.075	0.003	0.000	0.001
**ULC068-bin1**	0.020	0.001	0.000	0.000	0.004	0.018	0.004	0.001
**ULC068-bin2**	0.005				0.005	0.001		
**ULC068-nobin**	0.002	0.040	0.016	0.004	0.085	0.004	0.000	0.001
**ULC073-bin1**	0.054	0.002	0.002	0.000	0.008	0.451	0.022	0.012
**ULC073-bin2**	0.001	0.003	0.004	0.000	0.110	0.002		0.000
**ULC073-bin3**	0.001	0.023	0.303	0.083	0.137	0.004	0.000	0.000
**ULC073-bin4**	0.001	0.020	0.044	0.025	0.254	0.003		0.000
**ULC073-bin5**	0.026	0.005	0.001		0.025	0.108	0.201	0.048
**ULC073-bin6**	0.001	0.005	0.287	0.006	0.062	0.001		
**ULC073-nobin**	0.002	0.016	0.062	0.027	0.220	0.021	0.002	0.006
**ULC077-bin1**	0.032	0.001	0.000	0.000	0.003	0.036	0.002	0.001
**ULC077-nobin**	0.003	0.010	0.020	0.004	0.070	0.004	0.000	0.001
**ULC082-bin1**	0.018	0.004	0.003	0.001	0.007	0.037	0.001	0.001
**ULC082-bin2**	0.002	0.020	0.018	0.004	0.035	0.003	0.000	
**ULC082-bin3**	0.002	0.004	0.006	0.001	0.013	0.004		0.000
**ULC082-bin4**	0.001	0.040	0.028	0.006	0.041	0.005	0.001	
**ULC082-bin5**		0.001	0.001		0.002	0.001		
**ULC082-nobin**	0.001	0.015	0.010	0.003	0.026	0.003	0.000	0.000
**ULC084-bin1**	0.002	0.004	0.003	0.001	0.108	0.003		0.000
**ULC084-bin2**	0.002	0.036	0.026	0.007	0.036	0.003	0.001	
**ULC084-bin3**	0.017	0.004	0.004	0.001	0.008	0.035	0.001	0.001
**ULC084-nobin**	0.001	0.016	0.137	0.039	0.074	0.003	0.000	0.001
**ULC129-bin1**	0.128	0.001	0.001	0.000	0.014	0.051	0.002	0.001
**ULC129-nobin**	0.006	0.099	0.020	0.004	0.068	0.004	0.000	0.001
**ULC146-bin1**	0.001	0.008	0.006	0.002	0.032	0.003	0.000	0.000
**ULC146-bin2**	0.000	0.000	0.000		0.000	0.000		
**ULC146-bin3**	0.001	0.022	0.353	0.093	0.118	0.004	0.000	0.000
**ULC146-bin4**	0.001	0.003	0.004	0.000	0.110	0.002	0.000	
**ULC146-bin5**	0.002	0.043	0.023	0.004	0.040	0.004	0.000	0.000
**ULC146-bin6**	0.001	0.017	0.054	0.014	0.330	0.001		
**ULC146-bin7**		0.010	0.187	0.219	0.130		0.001	0.000
**ULC146-nobin**	0.004	0.014	0.024	0.008	0.143	0.006	0.000	0.001
**ULC165-bin1**	0.002	0.011	0.008	0.003	0.017	0.004	0.001	0.000
**ULC165-bin2**	0.001	0.035	0.018	0.006	0.033	0.003	0.001	
**ULC165-bin3**	0.001	0.006	0.009	0.003	0.036	0.004	0.000	0.000
**ULC165-bin4**	0.034	0.000	0.000		0.003	0.043	0.002	
**ULC165-nobin**	0.012	0.010	0.025	0.007	0.045	0.015	0.001	0.001
**ULC179-bin1**	0.001	0.036	0.020	0.006	0.036	0.004	0.000	0.000
**ULC179-bin2**	0.001	0.021	0.012	0.004	0.054	0.004	0.000	0.000
**ULC179-bin3**	0.001	0.026	0.045	0.018	0.060	0.003	0.000	0.000
**ULC179-bin4**	0.001	0.001	0.001	0.000	0.002	0.001	0.000	
**ULC179-bin5**	0.001	0.039	0.021	0.005	0.040	0.003		
**ULC179-bin6**	0.001	0.019	0.016	0.006	0.040	0.004		0.000
**ULC179-bin7**		0.001	0.003		0.014			
**ULC179-nobin**	0.003	0.010	0.008	0.001	0.022	0.003	0.000	0.001
**ULC186-bin1**	0.048	0.002	0.001	0.000	0.006	0.234	0.007	0.006
**ULC186-nobin**	0.009	0.005	0.004	0.001	0.018	0.040	0.003	0.005
**ULC187-bin1**	0.022	0.000	0.000		0.002	0.019	0.001	0.000
**ULC187-nobin**	0.002	0.021	0.129	0.032	0.138	0.003	0.000	0.001

	ULC065-bin1	ULC065-bin2	ULC065-nobin	ULC066-bin1	ULC066-bin2	ULC066-bin3	ULC066-nobin	ULC068-bin1
**ULC335-bin1**	0.005	0.000	0.001	0.030	0.000	0.000	0.001	0.028
**ULC335-bin2**			0.000	0.001	0.009		0.002	0.001
**ULC335-bin3**	0.000	0.001	0.001	0.001	0.031	0.000	0.007	0.001
**ULC335-bin4**	0.006	0.015	0.053	0.001	0.001	0.001	0.048	0.001
**ULC335-nobin**	0.004	0.011	0.046	0.001	0.004	0.002	0.033	0.001
**ULC007-bin1**	0.010	0.000	0.001	0.031	0.000	0.000	0.001	0.031
**ULC007-bin2**	0.002	0.001	0.001	0.015				0.022
**ULC007-nobin**	0.007		0.016	0.034	0.003		0.019	0.034
**ULC027-bin1**	0.011	0.001	0.002	0.020	0.000	0.000	0.002	0.019
**ULC027-bin2**	0.002	0.009	0.024	0.001	0.000	0.000	0.256	0.000
**ULC027-bin3**	0.003	0.011	0.157	0.001	0.000	0.001	0.127	0.001
**ULC027-bin4**	0.003	0.010	0.106				0.092	0.001
**ULC027-nobin**	0.003	0.008	0.079	0.001	0.000	0.008	0.077	0.002
**ULC041-bin1**	0.021	0.003	0.005	0.016	0.000	0.001	0.004	0.018
**ULC041-bin2**	0.005	0.001	0.006	0.009			0.003	0.025
**ULC041-nobin**	0.003	0.001	0.008	0.013			0.010	0.009
**ULC065-bin1**		0.006	0.007	0.009	0.000	0.000	0.006	0.010
**ULC065-bin2**	0.006		0.019	0.000	0.000	0.003	0.022	0.001
**ULC065-nobin**	0.004	0.009		0.001	0.000	0.001	0.087	0.001
**ULC066-bin1**	0.005	0.000	0.001		0.000	0.000	0.002	0.215
**ULC066-bin2**	0.000	0.000	0.000	0.001			0.002	0.001
**ULC066-bin3**	0.001	0.013	0.009	0.001			0.011	0.001
**ULC066-nobin**	0.003	0.009	0.070	0.001	0.001	0.001		0.001
**ULC068-bin1**	0.007	0.001	0.001	0.251	0.001	0.000	0.003	
**ULC068-bin2**				0.044				0.045
**ULC068-nobin**	0.004	0.110	0.105	0.009	0.000	0.001	0.073	0.005
**ULC073-bin1**	0.022	0.003	0.004	0.017	0.000	0.000	0.004	0.017
**ULC073-bin2**	0.002	0.009	0.009	0.001	0.001	0.073	0.303	0.001
**ULC073-bin3**	0.004	0.010	0.123	0.000	0.000	0.001	0.102	0.000
**ULC073-bin4**	0.003	0.011	0.036		0.000	0.001	0.034	0.000
**ULC073-bin5**	0.007		0.001	0.017		0.006	0.015	0.022
**ULC073-bin6**	0.001		0.198	0.001	0.001		0.181	
**ULC073-nobin**	0.003	0.009	0.079	0.001	0.000	0.001	0.057	0.001
**ULC077-bin1**	0.009	0.001	0.001	0.033	0.000	0.000	0.002	0.030
**ULC077-nobin**	0.004	0.129	0.046	0.002	0.000	0.029	0.147	0.002
**ULC082-bin1**	0.232	0.005	0.007	0.011	0.000	0.000	0.007	0.007
**ULC082-bin2**	0.004	0.010	0.132	0.000	0.000	0.001	0.051	0.001
**ULC082-bin3**	0.003	0.016	0.013	0.001	0.001	0.002	0.016	0.001
**ULC082-bin4**	0.006	0.021	0.048	0.000		0.001	0.051	0.000
**ULC082-bin5**		0.000	0.001				0.003	
**ULC082-nobin**	0.004	0.013	0.042	0.000	0.000	0.002	0.026	0.001
**ULC084-bin1**	0.002	0.011	0.009	0.001	0.001	0.085	0.329	0.001
**ULC084-bin2**	0.003	0.013	0.071	0.001	0.000	0.000	0.037	0.001
**ULC084-bin3**	0.225	0.006	0.009	0.008	0.000	0.000	0.007	0.009
**ULC084-nobin**	0.008	0.007	0.105	0.001	0.000	0.007	0.102	0.001
**ULC129-bin1**	0.012	0.001	0.001	0.019	0.000	0.000	0.002	0.017
**ULC129-nobin**	0.002	0.007	0.042	0.001	0.001	0.017	0.142	0.001
**ULC146-bin1**	0.005	0.018	0.071	0.000	0.000	0.005	0.028	0.000
**ULC146-bin2**	0.000	0.000		0.001	0.007		0.001	0.001
**ULC146-bin3**	0.003	0.012	0.144	0.000	0.000	0.001	0.120	0.000
**ULC146-bin4**	0.002	0.009	0.009	0.001	0.001	0.083	0.321	0.001
**ULC146-bin5**	0.004	0.016	0.047	0.000	0.000	0.000	0.045	0.001
**ULC146-bin6**	0.003	0.007	0.031	0.000	0.000	0.000	0.034	0.001
**ULC146-bin7**	0.002	0.007	0.125				0.103	0.000
**ULC146-nobin**	0.003	0.006	0.052	0.004	0.001	0.001	0.043	0.004
**ULC165-bin1**	0.006	0.415	0.076	0.000	0.000	0.001	0.020	0.001
**ULC165-bin2**	0.004	0.013	0.072	0.000	0.000	0.001	0.038	0.000
**ULC165-bin3**	0.004	0.019	0.086	0.000	0.000	0.004	0.033	0.000
**ULC165-bin4**	0.008	0.000	0.001	0.032	0.000	0.000	0.002	0.034
**ULC165-nobin**	0.005	0.010	0.040	0.009	0.000	0.001	0.031	0.009
**ULC179-bin1**	0.004	0.013	0.040	0.000	0.000	0.001	0.039	0.001
**ULC179-bin2**	0.003	0.009	0.036	0.001	0.000	0.000	0.038	0.001
**ULC179-bin3**	0.004	0.015	0.039	0.001	0.000	0.000	0.038	0.001
**ULC179-bin4**	0.001	0.002	0.002	0.000	0.004	0.000	0.002	0.001
**ULC179-bin5**	0.004	0.015	0.040	0.001		0.001	0.037	0.000
**ULC179-bin6**	0.003	0.009	0.043	0.000		0.000	0.033	0.001
**ULC179-bin7**			0.009				0.009	
**ULC179-nobin**	0.003	0.005	0.021	0.002	0.001	0.000	0.018	0.003
**ULC186-bin1**	0.022	0.003	0.004	0.019	0.000	0.000	0.004	0.018
**ULC186-nobin**	0.004	0.007	0.020	0.003	0.004	0.001	0.015	0.003
**ULC187-bin1**	0.008	0.000	0.001	0.201	0.000	0.000	0.002	0.187
**ULC187-nobin**	0.003	0.011	0.116	0.005	0.000	0.005	0.101	0.003

	ULC068-bin2	ULC068-nobin	ULC073-bin1	ULC073-bin2	ULC073-bin3	ULC073-bin4	ULC073-bin5	ULC073-bin6
**ULC335-bin1**	0.000	0.001	0.023	0.001	0.000	0.000	0.001	
**ULC335-bin2**		0.007	0.000	0.001	0.000	0.000		
**ULC335-bin3**	0.000	0.001	0.001	0.001	0.000			
**ULC335-bin4**		0.027	0.004	0.004	0.031	0.006		0.000
**ULC335-nobin**	0.000	0.015	0.004	0.011	0.021	0.003	0.000	0.001
**ULC007-bin1**	0.000	0.002	0.037	0.001	0.001	0.000	0.001	
**ULC007-bin2**			0.001			0.001	0.006	
**ULC007-nobin**	0.007	0.011	0.013	0.002	0.001		0.001	0.004
**ULC027-bin1**	0.000	0.001	0.053	0.001	0.001	0.000	0.002	0.000
**ULC027-bin2**		0.028	0.003	0.003	0.019	0.003	0.000	0.000
**ULC027-bin3**		0.014	0.003	0.004	0.352	0.009	0.000	0.031
**ULC027-bin4**		0.009	0.002	0.002	0.279	0.015		0.002
**ULC027-nobin**	0.000	0.029	0.004	0.044	0.057	0.019	0.001	0.002
**ULC041-bin1**	0.000	0.002	0.440	0.001	0.003	0.000	0.007	0.000
**ULC041-bin2**		0.002	0.182		0.000		0.111	
**ULC041-nobin**		0.006	0.179	0.001	0.001	0.001	0.049	
**ULC065-bin1**		0.004	0.033	0.002	0.004	0.001	0.001	0.000
**ULC065-bin2**		0.109	0.004	0.011	0.011	0.002		
**ULC065-nobin**		0.045	0.003	0.005	0.063	0.004	0.000	0.009
**ULC066-bin1**	0.002	0.006	0.015	0.001	0.000		0.001	0.000
**ULC066-bin2**		0.000	0.000	0.001	0.000	0.000		0.000
**ULC066-bin3**		0.005	0.004	0.461	0.006	0.001	0.003	
**ULC066-nobin**		0.026	0.003	0.113	0.043	0.003	0.000	0.006
**ULC068-bin1**	0.002	0.003	0.017	0.001	0.000	0.000	0.001	
**ULC068-bin2**		0.002		0.001				
**ULC068-nobin**	0.000		0.004	0.007	0.017	0.003	0.000	0.001
**ULC073-bin1**		0.003		0.002	0.002	0.000	0.002	0.000
**ULC073-bin2**	0.000	0.005	0.003		0.003	0.000	0.000	
**ULC073-bin3**		0.013	0.002	0.004		0.011	0.000	0.002
**ULC073-bin4**		0.014	0.002	0.002	0.063			
**ULC073-bin5**		0.003	0.015	0.001	0.001			
**ULC073-bin6**		0.012	0.001		0.021			
**ULC073-nobin**	0.000	0.026	0.004	0.005	0.040	0.007	0.001	0.001
**ULC077-bin1**	0.000	0.001	0.037	0.001	0.000	0.000	0.001	
**ULC077-nobin**	0.000	0.048	0.004	0.182	0.018	0.002	0.001	0.003
**ULC082-bin1**		0.004	0.035	0.002	0.004	0.001	0.001	
**ULC082-bin2**	0.000	0.021	0.004	0.004	0.017	0.003		0.000
**ULC082-bin3**		0.008	0.003	0.012	0.006	0.001		0.000
**ULC082-bin4**		0.026	0.003	0.008	0.032	0.008		0.001
**ULC082-bin5**		0.002		0.003	0.001			
**ULC082-nobin**	0.000	0.015	0.003	0.008	0.011	0.002	0.000	0.001
**ULC084-bin1**	0.000	0.005	0.003	0.502	0.004	0.000	0.000	0.000
**ULC084-bin2**		0.032	0.003	0.003	0.024	0.003		0.000
**ULC084-bin3**	0.000	0.005	0.039	0.002	0.003	0.000	0.002	
**ULC084-nobin**	0.000	0.017	0.003	0.049	0.133	0.005	0.002	0.018
**ULC129-bin1**	0.000	0.001	0.048	0.001	0.001	0.000	0.001	0.000
**ULC129-nobin**	0.000	0.020	0.004	0.097	0.020	0.003	0.001	0.003
**ULC146-bin1**		0.015	0.004	0.023	0.008	0.001	0.000	0.000
**ULC146-bin2**		0.006	0.000	0.000	0.000			0.000
**ULC146-bin3**	0.000	0.016	0.003	0.004	0.344	0.009		0.021
**ULC146-bin4**	0.000	0.005	0.002	0.456	0.003	0.000	0.000	
**ULC146-bin5**		0.023	0.004	0.003	0.024	0.003		0.001
**ULC146-bin6**	0.001	0.010	0.002	0.000	0.077	0.020		0.001
**ULC146-bin7**		0.008	0.002	0.003	0.308	0.009	0.000	0.000
**ULC146-nobin**	0.000	0.023	0.005	0.005	0.034	0.014	0.001	0.003
**ULC165-bin1**		0.105	0.004	0.009	0.011	0.002		0.000
**ULC165-bin2**		0.030	0.004	0.004	0.023	0.003	0.000	0.001
**ULC165-bin3**		0.016	0.003	0.027	0.012	0.001	0.000	0.000
**ULC165-bin4**	0.001	0.000	0.037	0.002			0.002	
**ULC165-nobin**	0.000	0.013	0.014	0.005	0.029	0.005	0.001	0.002
**ULC179-bin1**		0.020	0.003	0.004	0.025	0.005	0.000	0.001
**ULC179-bin2**		0.019	0.003	0.003	0.016	0.003	0.000	0.000
**ULC179-bin3**	0.000	0.017	0.003	0.003	0.051	0.008		0.000
**ULC179-bin4**		0.002	0.001	0.001	0.001	0.000	0.000	
**ULC179-bin5**		0.021	0.003	0.003	0.026	0.004		0.001
**ULC179-bin6**		0.022	0.003	0.004	0.022	0.005		0.000
**ULC179-bin7**		0.002		0.000	0.006	0.004		0.002
**ULC179-nobin**	0.000	0.010	0.003	0.002	0.008	0.001	0.000	0.001
**ULC186-bin1**	0.000	0.002	0.214	0.001	0.002	0.000	0.004	0.000
**ULC186-nobin**	0.000	0.009	0.038	0.007	0.003	0.000	0.002	0.001
**ULC187-bin1**	0.006	0.005	0.019	0.001	0.000	0.000	0.000	
**ULC187-nobin**	0.015	0.036	0.003	0.031	0.126	0.012	0.000	0.019

	ULC073-nobin	ULC077-bin1	ULC077-nobin	ULC082-bin1	ULC082-bin2	ULC082-bin3	ULC082-bin4	ULC082-bin5
**ULC335-bin1**	0.001	0.042	0.002	0.006	0.000	0.000		
**ULC335-bin2**	0.000	0.001	0.000		0.000	0.000	0.000	
**ULC335-bin3**	0.000	0.000	0.001	0.001	0.001	0.001	0.000	
**ULC335-bin4**	0.042	0.001	0.036	0.006	0.060	0.007	0.046	0.000
**ULC335-nobin**	0.035	0.001	0.021	0.003	0.025	0.008	0.010	0.000
**ULC007-bin1**	0.003	0.081	0.002	0.010	0.000	0.001	0.000	
**ULC007-bin2**	0.001	0.028	0.027	0.002				
**ULC007-nobin**	0.027	0.018	0.041	0.006	0.000			
**ULC027-bin1**	0.004	0.037	0.003	0.011	0.001	0.001	0.000	
**ULC027-bin2**	0.026	0.001	0.012	0.003	0.013	0.002	0.005	0.000
**ULC027-bin3**	0.141	0.000	0.036	0.002	0.014	0.004	0.004	0.000
**ULC027-bin4**	0.182	0.001	0.024	0.001	0.010	0.002	0.003	
**ULC027-nobin**	0.175	0.002	0.044	0.003	0.011	0.004	0.003	0.000
**ULC041-bin1**	0.033	0.042	0.005	0.022	0.002	0.002	0.001	0.000
**ULC041-bin2**	0.018	0.017	0.004	0.005	0.002		0.001	
**ULC041-nobin**	0.136	0.013	0.017	0.006		0.001		
**ULC065-bin1**	0.006	0.016	0.006	0.211	0.003	0.002	0.001	
**ULC065-bin2**	0.018	0.001	0.243	0.005	0.009	0.012	0.004	0.000
**ULC065-nobin**	0.081	0.001	0.035	0.003	0.049	0.005	0.004	0.000
**ULC066-bin1**	0.001	0.033	0.002	0.005	0.000	0.000	0.000	
**ULC066-bin2**	0.000	0.000	0.000	0.000	0.000	0.001		
**ULC066-bin3**	0.011	0.000	0.268	0.001	0.003	0.007	0.001	
**ULC066-nobin**	0.047	0.001	0.086	0.003	0.017	0.005	0.003	0.000
**ULC068-bin1**	0.001	0.036	0.002	0.004	0.000	0.001	0.000	
**ULC068-bin2**	0.002	0.003	0.002		0.002			
**ULC068-nobin**	0.065	0.002	0.093	0.004	0.019	0.007	0.005	0.000
**ULC073-bin1**	0.006	0.044	0.005	0.021	0.002	0.002	0.000	
**ULC073-bin2**	0.008	0.002	0.275	0.002	0.003	0.007	0.001	0.000
**ULC073-bin3**	0.078	0.000	0.028	0.003	0.013	0.003	0.004	0.000
**ULC073-bin4**	0.078	0.002	0.019	0.003	0.011	0.003	0.006	
**ULC073-bin5**	0.005	0.027	0.023	0.009				
**ULC073-bin6**	0.024		0.043		0.002	0.001	0.001	
**ULC073-nobin**		0.002	0.025	0.003	0.011	0.004	0.003	0.000
**ULC077-bin1**	0.002		0.011	0.008	0.001	0.001	0.000	
**ULC077-nobin**	0.032	0.018		0.003	0.008	0.007	0.004	0.000
**ULC082-bin1**	0.006	0.016	0.007		0.003	0.003	0.001	0.000
**ULC082-bin2**	0.028	0.001	0.015	0.003		0.003	0.004	
**ULC082-bin3**	0.011	0.002	0.017	0.004	0.004		0.001	0.001
**ULC082-bin4**	0.043	0.002	0.041	0.007	0.022	0.005		
**ULC082-bin5**	0.001		0.003			0.004		
**ULC082-nobin**	0.025	0.001	0.021	0.003	0.011	0.005	0.006	0.001
**ULC084-bin1**	0.017	0.001	0.279	0.002	0.003	0.008	0.001	0.000
**ULC084-bin2**	0.030	0.001	0.065	0.004	0.039	0.005	0.032	0.000
**ULC084-bin3**	0.006	0.014	0.006	0.383	0.003	0.003	0.001	
**ULC084-nobin**	0.087	0.001	0.052	0.014	0.033	0.004	0.004	0.000
**ULC129-bin1**	0.004	0.036	0.002	0.010	0.001	0.001	0.000	
**ULC129-nobin**	0.041	0.001	0.066	0.002	0.010	0.004	0.002	0.000
**ULC146-bin1**	0.062	0.001	0.028	0.005	0.006	0.013	0.002	0.000
**ULC146-bin2**		0.000	0.000		0.000	0.000	0.000	
**ULC146-bin3**	0.168	0.001	0.033	0.003	0.013	0.003	0.005	0.000
**ULC146-bin4**	0.020	0.001	0.278	0.001	0.002	0.007	0.001	0.001
**ULC146-bin5**	0.035	0.000	0.034	0.005	0.053	0.005	0.038	0.000
**ULC146-bin6**	0.305	0.001	0.014	0.001	0.010	0.004	0.006	0.000
**ULC146-bin7**	0.215	0.001	0.026	0.004	0.007	0.001	0.005	
**ULC146-nobin**	0.126	0.007	0.020	0.003	0.010	0.003	0.003	0.000
**ULC165-bin1**	0.017	0.001	0.235	0.005	0.006	0.013	0.003	0.000
**ULC165-bin2**	0.030	0.001	0.065	0.004	0.040	0.004	0.024	
**ULC165-bin3**	0.068	0.000	0.031	0.005	0.007	0.022	0.001	0.001
**ULC165-bin4**	0.003	0.068	0.003	0.008		0.001	0.000	
**ULC165-nobin**	0.051	0.020	0.016	0.005	0.007	0.004	0.002	0.000
**ULC179-bin1**	0.033	0.001	0.027	0.004	0.029	0.005	0.029	0.000
**ULC179-bin2**	0.028	0.000	0.012	0.003	0.014	0.004	0.004	0.000
**ULC179-bin3**	0.060	0.001	0.014	0.004	0.015	0.005	0.004	
**ULC179-bin4**	0.002	0.001	0.001	0.001	0.001	0.001	0.000	
**ULC179-bin5**	0.030	0.001	0.028	0.004	0.040	0.003	0.031	
**ULC179-bin6**	0.031	0.001	0.012	0.003	0.024	0.008	0.002	
**ULC179-bin7**	0.014	0.001	0.004	0.002	0.003		0.000	
**ULC179-nobin**	0.015	0.004	0.009	0.002	0.007	0.002	0.002	0.000
**ULC186-bin1**	0.017	0.042	0.005	0.022	0.002	0.002	0.000	0.000
**ULC186-nobin**	0.024	0.007	0.015	0.004	0.004	0.005	0.001	
**ULC187-bin1**	0.002	0.033	0.002	0.007	0.001	0.001	0.000	
**ULC187-nobin**	0.122	0.001	0.051	0.003	0.017	0.003	0.007	

	ULC082-nobin	ULC084-bin1	ULC084-bin2	ULC084-bin3	ULC084-nobin	ULC129-bin1	ULC129-nobin	ULC146-bin1
**ULC335-bin1**	0.001	0.001	0.000	0.006	0.000	0.023	0.001	0.000
**ULC335-bin2**	0.000	0.000	0.000	0.000	0.000	0.000	0.004	0.000
**ULC335-bin3**	0.002	0.002	0.001	0.000	0.001	0.001	0.004	0.001
**ULC335-bin4**	0.237	0.007	0.165	0.006	0.023	0.001	0.027	0.019
**ULC335-nobin**	0.114	0.010	0.037	0.003	0.023	0.002	0.023	0.085
**ULC007-bin1**	0.003	0.001	0.001	0.008	0.001	0.032	0.002	0.001
**ULC007-bin2**	0.001	0.001		0.005		0.009	0.002	
**ULC007-nobin**	0.011		0.002	0.006	0.013	0.015	0.017	0.009
**ULC027-bin1**	0.003	0.001	0.001	0.010	0.001	0.127	0.009	0.002
**ULC027-bin2**	0.043	0.003	0.029	0.002	0.013	0.001	0.165	0.010
**ULC027-bin3**	0.034	0.003	0.027	0.004	0.175	0.001	0.044	0.011
**ULC027-bin4**	0.028	0.002	0.021	0.002	0.140	0.001	0.028	0.011
**ULC027-nobin**	0.039	0.039	0.015	0.003	0.034	0.007	0.054	0.020
**ULC041-bin1**	0.007	0.002	0.002	0.021	0.002	0.052	0.006	0.004
**ULC041-bin2**	0.006		0.003	0.005	0.001	0.020	0.003	0.000
**ULC041-nobin**	0.007	0.001		0.006	0.010	0.009	0.009	0.007
**ULC065-bin1**	0.016	0.002	0.003	0.205	0.009	0.019	0.003	0.008
**ULC065-bin2**	0.048	0.012	0.014	0.007	0.009	0.002	0.013	0.030
**ULC065-nobin**	0.081	0.005	0.035	0.004	0.058	0.001	0.042	0.055
**ULC066-bin1**	0.001	0.001	0.000	0.004	0.000	0.016	0.001	0.000
**ULC066-bin2**	0.001	0.001	0.000	0.000	0.000	0.000	0.003	0.000
**ULC066-bin3**	0.035	0.478	0.002	0.001	0.052	0.002	0.189	0.046
**ULC066-nobin**	0.041	0.110	0.016	0.003	0.046	0.001	0.103	0.019
**ULC068-bin1**	0.002	0.000	0.001	0.006	0.001	0.017	0.001	0.000
**ULC068-bin2**	0.003	0.002		0.002	0.001	0.003	0.002	
**ULC068-nobin**	0.059	0.007	0.037	0.005	0.022	0.002	0.046	0.029
**ULC073-bin1**	0.007	0.002	0.002	0.024	0.002	0.049	0.006	0.004
**ULC073-bin2**	0.029	0.452	0.003	0.002	0.058	0.001	0.179	0.033
**ULC073-bin3**	0.036	0.004	0.022	0.003	0.145	0.001	0.039	0.012
**ULC073-bin4**	0.026	0.001	0.016	0.002	0.028	0.001	0.028	0.010
**ULC073-bin5**	0.008	0.001		0.016	0.032	0.011	0.018	0.002
**ULC073-bin6**	0.016	0.001	0.002		0.221	0.003	0.068	0.005
**ULC073-nobin**	0.041	0.007	0.015	0.003	0.048	0.003	0.039	0.046
**ULC077-bin1**	0.002	0.001	0.001	0.007	0.001	0.031	0.002	0.001
**ULC077-nobin**	0.048	0.166	0.039	0.003	0.039	0.002	0.081	0.028
**ULC082-bin1**	0.011	0.002	0.004	0.387	0.020	0.017	0.003	0.008
**ULC082-bin2**	0.052	0.004	0.047	0.004	0.049	0.002	0.025	0.013
**ULC082-bin3**	0.031	0.013	0.007	0.004	0.008	0.003	0.010	0.031
**ULC082-bin4**	0.161	0.005	0.225	0.006	0.030	0.000	0.030	0.021
**ULC082-bin5**	0.020	0.003	0.001		0.001	0.000	0.001	0.003
**ULC082-nobin**		0.009	0.057	0.005	0.020	0.001	0.019	0.074
**ULC084-bin1**	0.035		0.003	0.002	0.008	0.001	0.181	0.033
**ULC084-bin2**	0.215	0.003		0.006	0.016	0.001	0.019	0.012
**ULC084-bin3**	0.023	0.002	0.007		0.006	0.019	0.004	0.007
**ULC084-nobin**	0.067	0.007	0.014	0.004		0.001	0.050	0.021
**ULC129-bin1**	0.003	0.001	0.001	0.012	0.001		0.007	0.001
**ULC129-nobin**	0.035	0.088	0.010	0.002	0.030	0.006		0.043
**ULC146-bin1**	0.174	0.020	0.007	0.004	0.015	0.001	0.054	
**ULC146-bin2**	0.000	0.000	0.000	0.000	0.000	0.000	0.004	0.000
**ULC146-bin3**	0.039	0.003	0.025	0.004	0.172	0.001	0.038	0.012
**ULC146-bin4**	0.030	0.439	0.003	0.002	0.048	0.001	0.180	0.029
**ULC146-bin5**	0.248	0.005	0.185	0.005	0.024	0.001	0.024	0.013
**ULC146-bin6**	0.024	0.001	0.011	0.002	0.024	0.001	0.020	0.008
**ULC146-bin7**	0.026	0.002	0.022	0.003	0.148		0.031	0.008
**ULC146-nobin**	0.047	0.004	0.020	0.003	0.028	0.004	0.030	0.009
**ULC165-bin1**	0.040	0.010	0.013	0.007	0.009	0.002	0.012	0.029
**ULC165-bin2**	0.210	0.004	0.483	0.004	0.038	0.001	0.020	0.013
**ULC165-bin3**	0.169	0.025	0.008	0.004	0.016	0.002	0.058	0.477
**ULC165-bin4**	0.001	0.001	0.000	0.006	0.001	0.034	0.004	0.001
**ULC165-nobin**	0.058	0.005	0.013	0.005	0.020	0.011	0.022	0.123
**ULC179-bin1**	0.185	0.003	0.149	0.003	0.022	0.001	0.021	0.014
**ULC179-bin2**	0.064	0.003	0.018	0.003	0.016	0.001	0.016	0.015
**ULC179-bin3**	0.040	0.003	0.027	0.005	0.026	0.002	0.017	0.014
**ULC179-bin4**	0.002	0.001	0.002	0.001	0.001	0.001	0.005	0.002
**ULC179-bin5**	0.178	0.004	0.157	0.003	0.022	0.001	0.019	0.010
**ULC179-bin6**	0.044	0.003	0.020	0.003	0.017	0.002	0.024	0.013
**ULC179-bin7**	0.010	0.000	0.003		0.006	0.000	0.002	0.003
**ULC179-nobin**	0.028	0.002	0.011	0.002	0.011	0.002	0.011	0.010
**ULC186-bin1**	0.006	0.001	0.002	0.020	0.002	0.047	0.003	0.004
**ULC186-nobin**	0.037	0.006	0.005	0.005	0.011	0.007	0.018	0.035
**ULC187-bin1**	0.002	0.001	0.000	0.007	0.001	0.019	0.001	0.001
**ULC187-nobin**	0.060	0.029	0.075	0.004	0.085	0.001	0.053	0.015

	**ULC146-bin2**	**ULC146-bin3**	**ULC146-bin4**	**ULC146-bin5**	**ULC146-bin6**	**ULC146-bin7**	**ULC146-nobin**	**ULC165-bin1**
**ULC335-bin1**	0.000	0.000	0.001	0.000			0.011	0.000
**ULC335-bin2**	0.124		0.000	0.000			0.006	0.000
**ULC335-bin3**	0.005	0.000	0.002	0.001		0.000	0.002	0.001
**ULC335-bin4**	0.000	0.036	0.004	0.212	0.007	0.006	0.064	0.021
**ULC335-nobin**	0.001	0.025	0.010	0.042	0.004	0.004	0.037	0.013
**ULC007-bin1**	0.000	0.001	0.001	0.000	0.000	0.000	0.015	0.001
**ULC007-bin2**							0.010	0.001
**ULC007-nobin**		0.008	0.000	0.001			0.021	
**ULC027-bin1**	0.000	0.001	0.001	0.001	0.000		0.008	0.001
**ULC027-bin2**	0.000	0.021	0.003	0.027	0.004	0.002	0.027	0.010
**ULC027-bin3**	0.000	0.460	0.005	0.020	0.019	0.040	0.073	0.009
**ULC027-bin4**		0.354	0.001	0.012	0.014	0.135	0.075	0.010
**ULC027-nobin**	0.000	0.054	0.044	0.014	0.039	0.010	0.160	0.008
**ULC041-bin1**	0.000	0.003	0.002	0.002	0.000		0.010	0.003
**ULC041-bin2**		0.001	0.001	0.001		0.001	0.003	0.005
**ULC041-nobin**		0.001		0.001		0.001	0.012	0.002
**ULC065-bin1**	0.000	0.004	0.002	0.003	0.001	0.000	0.008	0.007
**ULC065-bin2**	0.000	0.015	0.011	0.014	0.002	0.002	0.017	0.488
**ULC065-nobin**	0.000	0.081	0.005	0.020	0.005	0.012	0.070	0.036
**ULC066-bin1**	0.000	0.000	0.001	0.000	0.000		0.007	0.000
**ULC066-bin2**	0.010	0.001	0.001	0.000	0.000		0.002	0.000
**ULC066-bin3**		0.003	0.521	0.001	0.000		0.015	0.007
**ULC066-nobin**	0.001	0.055	0.118	0.016	0.004	0.008	0.048	0.010
**ULC068-bin1**	0.001	0.000	0.001	0.000	0.000	0.000	0.009	0.000
**ULC068-bin2**		0.001	0.001		0.003		0.002	
**ULC068-nobin**	0.008	0.022	0.006	0.023	0.004	0.002	0.067	0.126
**ULC073-bin1**	0.000	0.002	0.002	0.002	0.000	0.000	0.009	0.003
**ULC073-bin2**	0.000	0.004	0.453	0.002	0.000	0.001	0.013	0.009
**ULC073-bin3**	0.000	0.383	0.003	0.020	0.024	0.055	0.093	0.011
**ULC073-bin4**		0.059	0.002	0.013	0.033	0.010	0.211	0.011
**ULC073-bin5**			0.001			0.001	0.030	
**ULC073-bin6**		0.244		0.005	0.001	0.001	0.107	0.003
**ULC073-nobin**	0.000	0.093	0.009	0.015	0.047	0.019	0.178	0.010
**ULC077-bin1**	0.000	0.001	0.001	0.000	0.000	0.000	0.012	0.001
**ULC077-nobin**	0.000	0.023	0.184	0.017	0.003	0.003	0.034	0.146
**ULC082-bin1**		0.004	0.002	0.004	0.000	0.001	0.009	0.006
**ULC082-bin2**	0.000	0.020	0.003	0.053	0.004	0.002	0.035	0.008
**ULC082-bin3**	0.001	0.006	0.011	0.006	0.002	0.000	0.010	0.020
**ULC082-bin4**	0.001	0.037	0.006	0.211	0.013	0.006	0.061	0.023
**ULC082-bin5**		0.000	0.005	0.002	0.001		0.001	0.002
**ULC082-nobin**	0.000	0.014	0.009	0.054	0.002	0.002	0.035	0.013
**ULC084-bin1**	0.000	0.004	0.487	0.004	0.000	0.000	0.010	0.010
**ULC084-bin2**	0.000	0.030	0.003	0.146	0.004	0.004	0.055	0.014
**ULC084-bin3**	0.000	0.005	0.002	0.004	0.001	0.001	0.009	0.008
**ULC084-nobin**	0.000	0.174	0.040	0.017	0.006	0.025	0.067	0.008
**ULC129-bin1**	0.000	0.001	0.001	0.001	0.000	0.000	0.008	0.001
**ULC129-nobin**	0.002	0.023	0.097	0.010	0.003	0.003	0.040	0.007
**ULC146-bin1**	0.000	0.009	0.020	0.007	0.002	0.001	0.013	0.019
**ULC146-bin2**		0.000	0.000	0.000			0.004	0.000
**ULC146-bin3**	0.000		0.003	0.020	0.017	0.001	0.066	0.011
**ULC146-bin4**	0.001	0.003		0.004	0.001	0.000	0.007	0.009
**ULC146-bin5**	0.000	0.030	0.006		0.007	0.003	0.039	0.020
**ULC146-bin6**		0.063	0.002	0.017		0.008	0.044	0.007
**ULC146-bin7**		0.003	0.002	0.010	0.013		0.066	0.013
**ULC146-nobin**	0.002	0.029	0.003	0.012	0.005	0.004		0.006
**ULC165-bin1**	0.000	0.012	0.009	0.015	0.002	0.003	0.014	
**ULC165-bin2**	0.000	0.030	0.003	0.147	0.005	0.003	0.057	0.012
**ULC165-bin3**	0.000	0.010	0.023	0.007	0.002	0.001	0.016	0.018
**ULC165-bin4**	0.000	0.000	0.001		0.000		0.015	0.000
**ULC165-nobin**	0.000	0.032	0.005	0.011	0.008	0.005	0.046	0.009
**ULC179-bin1**	0.000	0.026	0.004	0.148	0.005	0.004	0.062	0.015
**ULC179-bin2**	0.000	0.020	0.004	0.020	0.003	0.002	0.128	0.009
**ULC179-bin3**	0.000	0.060	0.003	0.025	0.012	0.014	0.053	0.015
**ULC179-bin4**	0.004	0.002	0.001	0.001	0.000	0.000	0.006	0.002
**ULC179-bin5**	0.000	0.029	0.003	0.152	0.006	0.002	0.056	0.014
**ULC179-bin6**		0.020	0.003	0.020	0.004	0.002	0.055	0.015
**ULC179-bin7**		0.009		0.008	0.000	0.000	0.009	0.000
**ULC179-nobin**	0.001	0.010	0.002	0.009	0.002	0.001	0.037	0.005
**ULC186-bin1**	0.000	0.002	0.002	0.002	0.000	0.000	0.009	0.003
**ULC186-nobin**	0.004	0.007	0.008	0.003	0.001	0.001	0.018	0.006
**ULC187-bin1**	0.001	0.001	0.001	0.000	0.000	0.000	0.008	0.000
**ULC187-nobin**	0.001	0.162	0.029	0.032	0.012	0.021	0.098	0.010

	ULC165-bin2	ULC165-bin3	ULC165-bin4	ULC165-nobin	ULC179-bin1	ULC179-bin2	ULC179-bin3	ULC179-bin4
**ULC335-bin1**	0.000		0.010	0.022	0.000	0.000	0.000	0.000
**ULC335-bin2**	0.000		0.000	0.000	0.000		0.000	0.004
**ULC335-bin3**	0.000	0.000	0.000	0.001	0.001	0.000	0.001	0.005
**ULC335-bin4**	0.166	0.009	0.000	0.032	0.232	0.041	0.038	0.002
**ULC335-nobin**	0.038	0.036	0.000	0.055	0.057	0.021	0.021	0.002
**ULC007-bin1**	0.000	0.000	0.018	0.039	0.001	0.001	0.001	0.001
**ULC007-bin2**			0.007	0.017				
**ULC007-nobin**	0.001		0.007	0.043		0.002	0.006	
**ULC027-bin1**	0.001	0.001	0.009	0.024	0.001	0.001	0.001	0.001
**ULC027-bin2**	0.029	0.004	0.000	0.021	0.043	0.027	0.028	0.001
**ULC027-bin3**	0.020	0.007	0.000	0.072	0.033	0.021	0.064	0.001
**ULC027-bin4**	0.019	0.006		0.059	0.028	0.018	0.076	0.000
**ULC027-nobin**	0.015	0.010	0.000	0.051	0.023	0.036	0.032	0.001
**ULC041-bin1**	0.002	0.002	0.012	0.030	0.004	0.004	0.003	0.001
**ULC041-bin2**	0.004	0.000	0.006	0.014	0.000	0.000	0.001	0.000
**ULC041-nobin**		0.002		0.016	0.001	0.002	0.004	
**ULC065-bin1**	0.004	0.003	0.003	0.012	0.006	0.005	0.005	0.001
**ULC065-bin2**	0.015	0.014	0.000	0.029	0.022	0.015	0.021	0.003
**ULC065-nobin**	0.037	0.027	0.000	0.057	0.031	0.030	0.026	0.001
**ULC066-bin1**	0.000	0.000	0.008	0.017	0.000	0.001	0.001	0.000
**ULC066-bin2**	0.000	0.000	0.000	0.001	0.000	0.000	0.000	0.005
**ULC066-bin3**	0.004	0.015	0.000	0.022	0.007	0.003	0.002	0.001
**ULC066-nobin**	0.017	0.009	0.000	0.034	0.025	0.025	0.023	0.001
**ULC068-bin1**	0.000	0.000	0.010	0.019	0.001	0.001	0.001	0.001
**ULC068-bin2**			0.003	0.007			0.001	
**ULC068-nobin**	0.037	0.013	0.000	0.040	0.035	0.034	0.027	0.002
**ULC073-bin1**	0.003	0.001	0.010	0.028	0.003	0.003	0.003	0.001
**ULC073-bin2**	0.004	0.016	0.001	0.015	0.005	0.004	0.003	0.001
**ULC073-bin3**	0.022	0.008		0.075	0.037	0.025	0.064	0.001
**ULC073-bin4**	0.019	0.004		0.077	0.036	0.022	0.055	0.000
**ULC073-bin5**		0.001	0.010	0.018	0.001	0.001		0.001
**ULC073-bin6**	0.006	0.003		0.066	0.015	0.006	0.007	
**ULC073-nobin**	0.015	0.021	0.000	0.069	0.024	0.022	0.039	0.001
**ULC077-bin1**	0.001	0.000	0.016	0.036	0.001	0.000	0.000	0.000
**ULC077-nobin**	0.040	0.014	0.001	0.030	0.025	0.012	0.012	0.001
**ULC082-bin1**	0.004	0.004	0.004	0.014	0.007	0.006	0.005	0.001
**ULC082-bin2**	0.051	0.007		0.024	0.059	0.029	0.025	0.001
**ULC082-bin3**	0.006	0.024	0.000	0.015	0.011	0.009	0.009	0.001
**ULC082-bin4**	0.169	0.004	0.000	0.040	0.280	0.042	0.038	0.001
**ULC082-bin5**		0.008		0.002	0.002	0.001		0.000
**ULC082-nobin**	0.057	0.030	0.000	0.048	0.075	0.028	0.016	0.001
**ULC084-bin1**	0.004	0.017	0.001	0.015	0.005	0.005	0.004	0.001
**ULC084-bin2**	0.491	0.005	0.000	0.035	0.218	0.030	0.034	0.002
**ULC084-bin3**	0.004	0.003	0.003	0.013	0.006	0.005	0.006	0.001
**ULC084-nobin**	0.031	0.010	0.000	0.050	0.028	0.023	0.031	0.001
**ULC129-bin1**	0.001	0.001	0.009	0.023	0.001	0.001	0.002	0.001
**ULC129-nobin**	0.011	0.019	0.001	0.033	0.016	0.013	0.012	0.003
**ULC146-bin1**	0.008	0.196	0.000	0.243	0.013	0.014	0.012	0.001
**ULC146-bin2**	0.000	0.000	0.000	0.000	0.000	0.000	0.000	0.004
**ULC146-bin3**	0.025	0.006	0.000	0.073	0.032	0.027	0.066	0.002
**ULC146-bin4**	0.002	0.014	0.000	0.015	0.005	0.005	0.004	0.001
**ULC146-bin5**	0.189	0.006		0.039	0.272	0.041	0.041	0.002
**ULC146-bin6**	0.017	0.003		0.067	0.024	0.016	0.048	0.000
**ULC146-bin7**	0.015	0.005		0.064	0.026	0.014	0.090	0.001
**ULC146-nobin**	0.021	0.004	0.002	0.046	0.032	0.076	0.027	0.003
**ULC165-bin1**	0.012	0.012	0.000	0.021	0.021	0.013	0.018	0.002
**ULC165-bin2**		0.006	0.000	0.027	0.223	0.035	0.034	0.002
**ULC165-bin3**	0.009		0.000	0.017	0.014	0.011	0.010	0.002
**ULC165-bin4**	0.001	0.000		0.013	0.000	0.001	0.000	0.000
**ULC165-nobin**	0.010	0.004	0.002		0.017	0.021	0.029	0.001
**ULC179-bin1**	0.156	0.006	0.000	0.031		0.037	0.032	0.002
**ULC179-bin2**	0.022	0.005	0.000	0.036	0.033		0.024	0.001
**ULC179-bin3**	0.026	0.006	0.000	0.059	0.038	0.029		0.002
**ULC179-bin4**	0.002	0.001	0.000	0.002	0.002	0.002	0.002	
**ULC179-bin5**	0.156	0.004		0.027	0.196	0.026	0.029	0.001
**ULC179-bin6**	0.021	0.009		0.025	0.035	0.048	0.020	0.002
**ULC179-bin7**	0.007	0.001		0.012	0.008	0.002	0.021	0.000
**ULC179-nobin**	0.012	0.004	0.001	0.019	0.015	0.023	0.012	0.003
**ULC186-bin1**	0.002	0.002	0.011	0.028	0.003	0.003	0.003	0.001
**ULC186-nobin**	0.004	0.017	0.002	0.029	0.007	0.009	0.008	0.002
**ULC187-bin1**	0.001	0.000	0.009	0.017	0.000	0.000	0.001	0.000
**ULC187-nobin**	0.076	0.006	0.000	0.051	0.048	0.029	0.038	0.002

	ULC179-bin5	ULC179-bin6	ULC179-bin7	ULC179-nobin	ULC186-bin1	ULC186-nobin	ULC187-bin1	ULC187-nobin
**ULC335-bin1**	0.000			0.005	0.023	0.003	0.030	0.000
**ULC335-bin2**				0.002	0.000	0.004	0.001	0.000
**ULC335-bin3**	0.000	0.000		0.003	0.001	0.002	0.001	0.001
**ULC335-bin4**	0.064	0.007	0.001	0.027	0.004	0.004	0.001	0.029
**ULC335-nobin**	0.018	0.003	0.000	0.017	0.003	0.013	0.001	0.014
**ULC007-bin1**	0.000	0.000		0.006	0.040	0.003	0.027	0.001
**ULC007-bin2**				0.004	0.013	0.005	0.001	
**ULC007-nobin**				0.019	0.016	0.013	0.022	0.011
**ULC027-bin1**	0.000	0.000	0.000	0.004	0.050	0.005	0.021	0.001
**ULC027-bin2**	0.014	0.003	0.000	0.018	0.002	0.003	0.001	0.012
**ULC027-bin3**	0.010	0.003	0.000	0.019	0.002	0.003	0.001	0.104
**ULC027-bin4**	0.007	0.004		0.009	0.002	0.003		0.079
**ULC027-nobin**	0.008	0.004	0.001	0.019	0.004	0.005	0.001	0.039
**ULC041-bin1**	0.001	0.001		0.005	0.249	0.020	0.018	0.001
**ULC041-bin2**				0.001	0.063	0.014	0.012	0.001
**ULC041-nobin**		0.001		0.009	0.097	0.028	0.007	0.007
**ULC065-bin1**	0.002	0.001		0.006	0.034	0.003	0.011	0.002
**ULC065-bin2**	0.008	0.002		0.011	0.005	0.005	0.001	0.009
**ULC065-nobin**	0.010	0.005	0.001	0.024	0.003	0.007	0.001	0.042
**ULC066-bin1**	0.000	0.000		0.003	0.017	0.002	0.170	0.001
**ULC066-bin2**				0.003	0.000	0.003	0.001	0.000
**ULC066-bin3**	0.003	0.000		0.003	0.004	0.005	0.002	0.020
**ULC066-nobin**	0.008	0.003	0.000	0.017	0.003	0.005	0.001	0.028
**ULC068-bin1**	0.000	0.000		0.004	0.019	0.002	0.185	0.001
**ULC068-bin2**				0.001	0.001	0.001	0.131	0.149
**ULC068-nobin**	0.011	0.005	0.000	0.024	0.003	0.008	0.009	0.032
**ULC073-bin1**	0.001	0.000		0.005	0.236	0.020	0.018	0.001
**ULC073-bin2**	0.001	0.001	0.000	0.004	0.002	0.004	0.001	0.021
**ULC073-bin3**	0.012	0.004	0.001	0.016	0.003	0.002	0.000	0.087
**ULC073-bin4**	0.010	0.005	0.003	0.017	0.002	0.001	0.000	0.043
**ULC073-bin5**				0.002	0.075	0.014	0.008	
**ULC073-bin6**	0.003	0.001	0.003	0.014	0.002	0.005		0.142
**ULC073-nobin**	0.007	0.004	0.001	0.017	0.012	0.007	0.001	0.044
**ULC077-bin1**	0.000	0.000	0.000	0.005	0.039	0.003	0.028	0.001
**ULC077-nobin**	0.008	0.002	0.000	0.011	0.004	0.007	0.001	0.023
**ULC082-bin1**	0.002	0.001	0.000	0.006	0.038	0.004	0.010	0.002
**ULC082-bin2**	0.023	0.007	0.001	0.019	0.005	0.003	0.001	0.017
**ULC082-bin3**	0.002	0.003		0.008	0.004	0.005	0.001	0.004
**ULC082-bin4**	0.096	0.004	0.001	0.022	0.004	0.002	0.001	0.032
**ULC082-bin5**				0.001	0.001			
**ULC082-nobin**	0.023	0.003	0.000	0.017	0.003	0.007	0.001	0.013
**ULC084-bin1**	0.002	0.001	0.000	0.003	0.002	0.004	0.001	0.023
**ULC084-bin2**	0.072	0.004	0.000	0.024	0.003	0.003	0.001	0.057
**ULC084-bin3**	0.002	0.001		0.005	0.035	0.004	0.011	0.003
**ULC084-nobin**	0.008	0.003	0.001	0.020	0.003	0.007	0.001	0.053
**ULC129-bin1**	0.000	0.000	0.000	0.003	0.050	0.004	0.018	0.001
**ULC129-nobin**	0.004	0.003	0.000	0.012	0.003	0.006	0.001	0.020
**ULC146-bin1**	0.003	0.002	0.000	0.012	0.004	0.015	0.001	0.007
**ULC146-bin2**	0.000			0.001	0.000	0.002	0.001	0.000
**ULC146-bin3**	0.011	0.004	0.002	0.017	0.003	0.004	0.001	0.102
**ULC146-bin4**	0.001	0.001		0.003	0.003	0.005	0.001	0.020
**ULC146-bin5**	0.088	0.005	0.002	0.025	0.003	0.003	0.000	0.031
**ULC146-bin6**	0.008	0.002	0.000	0.012	0.002	0.001	0.000	0.029
**ULC146-bin7**	0.004	0.003	0.000	0.011	0.002	0.003	0.001	0.088
**ULC146-nobin**	0.009	0.005	0.001	0.030	0.005	0.005	0.004	0.026
**ULC165-bin1**	0.006	0.003	0.000	0.009	0.004	0.004	0.000	0.007
**ULC165-bin2**	0.070	0.004	0.001	0.025	0.004	0.003	0.001	0.056
**ULC165-bin3**	0.003	0.003	0.000	0.013	0.005	0.017	0.001	0.007
**ULC165-bin4**				0.005	0.041	0.003	0.032	0.000
**ULC165-nobin**	0.005	0.002	0.001	0.014	0.015	0.007	0.008	0.014
**ULC179-bin1**	0.049	0.005	0.001	0.022	0.003	0.003	0.000	0.024
**ULC179-bin2**	0.008	0.007	0.000	0.032	0.003	0.004	0.000	0.013
**ULC179-bin3**	0.010	0.003	0.002	0.019	0.003	0.004	0.001	0.021
**ULC179-bin4**	0.000	0.000	0.000	0.005	0.002	0.001	0.000	0.001
**ULC179-bin5**		0.006	0.001	0.023	0.003	0.002	0.001	0.024
**ULC179-bin6**	0.013		0.001	0.022	0.003	0.008	0.001	0.013
**ULC179-bin7**	0.003	0.001		0.020			0.001	0.003
**ULC179-nobin**	0.005	0.003	0.001		0.004	0.004	0.002	0.008
**ULC186-bin1**	0.001	0.000		0.006		0.007	0.019	0.002
**ULC186-nobin**	0.002	0.002		0.012	0.020		0.003	0.010
**ULC187-bin1**	0.000	0.000	0.000	0.003	0.021	0.002		0.005
**ULC187-nobin**	0.015	0.004	0.001	0.021	0.004	0.010	0.010	

	ULC335-bin1	ULC335-bin2	ULC335-bin3	ULC335-bin4	ULC335-nobin	ULC007-bin1	ULC007-bin2	ULC007-nobin
**ULC335-bin1**								
**ULC335-bin2**	0.562							
**ULC335-bin3**	0.000	0.000						
**ULC335-bin4**	0.000	0.000	0.000					
**ULC335-nobin**	0.372	0.157	0.000	0.000				
**ULC007-bin1**	0.000	0.000	0.000	0.000	0.000			
**ULC007-bin2**	0.000	0.000	0.000	0.000	0.000	0.000		
**ULC007-nobin**	0.000	0.000	0.000	0.000	0.213	1.063	0.106	
**ULC027-bin1**	0.000	0.000	0.000	0.000	0.000	0.000	0.000	0.106
**ULC027-bin2**	0.000	0.000	0.000	0.000	0.000	0.000	0.000	0.106
**ULC027-bin3**	0.000	0.000	0.000	0.000	0.000	0.000	0.000	0.106
**ULC027-bin4**	0.000	0.000	0.000	0.000	0.000	0.000	0.000	0.000
**ULC027-nobin**	0.000	0.000	0.000	0.000	0.008	0.005	0.000	0.106
**ULC041-bin1**	0.000	0.000	0.000	0.000	0.000	0.000	0.000	0.850
**ULC041-bin2**	0.000	0.000	0.000	0.000	0.000	0.000	0.000	0.106
**ULC041-nobin**	0.000	0.000	0.000	0.000	0.071	0.142	0.000	0.106
**ULC065-bin1**	0.000	0.000	0.000	0.000	0.000	0.000	0.000	0.531
**ULC065-bin2**	0.000	0.000	0.000	0.000	0.010	0.000	0.000	0.000
**ULC065-nobin**	0.000	0.000	0.000	0.000	0.011	0.010	0.000	0.319
**ULC066-bin1**	0.000	0.000	0.000	0.000	0.000	0.000	0.000	2.019
**ULC066-bin2**	0.000	0.000	0.000	0.000	0.000	0.000	0.000	0.213
**ULC066-bin3**	0.000	0.000	0.000	0.000	0.000	0.000	0.000	0.000
**ULC066-nobin**	0.000	0.000	0.000	0.000	0.006	0.010	0.000	0.425
**ULC068-bin1**	0.000	0.000	0.000	0.000	0.000	0.000	0.000	0.850
**ULC068-bin2**	0.000	0.000	0.000	0.000	0.000	0.000	0.000	0.000
**ULC068-nobin**	0.000	0.000	0.000	0.000	0.008	0.025	0.000	0.106
**ULC073-bin1**	0.000	0.000	0.000	0.000	0.000	0.000	0.000	0.319
**ULC073-bin2**	0.000	0.000	0.000	0.000	0.000	0.000	0.000	0.106
**ULC073-bin3**	0.000	0.000	0.000	0.000	0.000	0.000	0.000	0.106
**ULC073-bin4**	0.000	0.000	0.000	0.000	0.000	0.000	0.000	0.000
**ULC073-bin5**	0.000	0.000	0.000	0.000	0.000	0.000	0.000	0.000
**ULC073-bin6**	0.000	0.000	0.000	0.000	0.000	0.000	0.000	0.000
**ULC073-nobin**	0.000	0.000	0.000	0.000	0.007	0.010	0.000	0.213
**ULC077-bin1**	0.000	0.000	0.000	0.000	0.000	0.000	0.000	0.531
**ULC077-nobin**	0.000	0.000	0.000	0.000	0.009	0.010	0.000	3.613
**ULC082-bin1**	0.000	0.000	0.000	0.000	0.000	0.000	0.000	0.000
**ULC082-bin2**	0.000	0.000	0.000	0.025	0.011	0.000	0.000	0.000
**ULC082-bin3**	0.000	0.000	0.000	0.000	0.000	0.000	0.000	0.000
**ULC082-bin4**	0.000	0.000	0.000	0.000	0.000	0.000	0.000	0.000
**ULC082-bin5**	0.000	0.000	0.000	0.000	0.000	0.000	0.000	0.000
**ULC082-nobin**	0.000	0.000	0.000	0.025	0.048	0.000	0.000	0.213
**ULC084-bin1**	0.000	0.000	0.000	0.000	0.000	0.000	0.000	0.000
**ULC084-bin2**	0.000	0.000	0.000	0.000	0.000	0.000	0.000	0.000
**ULC084-bin3**	0.000	0.000	0.000	0.000	0.000	0.000	0.000	0.000
**ULC084-nobin**	0.000	0.000	0.009	0.000	0.092	0.000	0.000	0.213
**ULC129-bin1**	0.000	0.000	0.000	0.000	0.000	0.000	0.000	0.000
**ULC129-nobin**	0.005	0.000	0.000	0.000	0.048	0.000	0.000	0.213
**ULC146-bin1**	0.000	0.000	0.000	0.000	0.046	0.000	0.000	0.000
**ULC146-bin2**	0.000	0.000	0.000	0.000	0.000	0.000	0.000	0.000
**ULC146-bin3**	0.000	0.000	0.000	0.000	0.000	0.000	0.000	0.106
**ULC146-bin4**	0.000	0.000	0.000	0.000	0.000	0.000	0.000	0.106
**ULC146-bin5**	0.000	0.000	0.000	0.000	0.011	0.000	0.000	0.000
**ULC146-bin6**	0.000	0.000	0.000	0.000	0.000	0.000	0.000	0.000
**ULC146-bin7**	0.000	0.000	0.000	0.000	0.000	0.000	0.000	0.000
**ULC146-nobin**	0.000	0.000	0.000	0.000	0.065	0.000	0.000	0.213
**ULC165-bin1**	0.000	0.000	0.000	0.000	0.008	0.000	0.000	0.000
**ULC165-bin2**	0.000	0.000	0.000	0.000	0.000	0.000	0.000	0.000
**ULC165-bin3**	0.000	0.000	0.000	0.000	0.064	0.000	0.000	0.000
**ULC165-bin4**	0.000	0.000	0.000	0.000	0.000	0.000	0.000	0.000
**ULC165-nobin**	0.005	0.017	0.000	0.000	0.043	0.000	0.000	0.319
**ULC179-bin1**	0.000	0.000	0.000	0.000	0.000	0.000	0.000	0.000
**ULC179-bin2**	0.000	0.000	0.000	0.000	0.000	0.000	0.000	0.000
**ULC179-bin3**	0.000	0.000	0.000	0.000	0.000	0.000	0.000	0.000
**ULC179-bin4**	0.000	0.000	0.000	0.000	0.000	0.000	0.000	0.000
**ULC179-bin5**	0.000	0.000	0.000	0.000	0.000	0.000	0.000	0.000
**ULC179-bin6**	0.000	0.000	0.000	0.000	0.000	0.000	0.000	0.000
**ULC179-bin7**	0.000	0.000	0.000	0.000	0.000	0.000	0.000	0.000
**ULC179-nobin**	0.000	0.000	0.000	0.000	0.014	0.000	0.000	0.213
**ULC186-bin1**	0.000	0.000	0.000	0.000	0.005	0.000	0.000	0.000
**ULC186-nobin**	0.022	0.022	0.000	0.000	0.345	0.000	0.000	0.213
**ULC187-bin1**	0.000	0.000	0.000	0.000	0.006	0.000	0.000	0.000
**ULC187-nobin**	0.000	0.000	0.000	0.000	0.112	0.000	0.000	0.213

	ULC027-bin1	ULC027-bin2	ULC027-bin3	ULC027-bin4	ULC027-nobin	ULC041-bin1	ULC041-bin2	ULC041-nobin
**ULC335-bin1**								
**ULC335-bin2**								
**ULC335-bin3**								
**ULC335-bin4**								
**ULC335-nobin**								
**ULC007-bin1**								
**ULC007-bin2**								
**ULC007-nobin**								
**ULC027-bin1**								
**ULC027-bin2**	0.000							
**ULC027-bin3**	0.000	0.000						
**ULC027-bin4**	0.000	0.000	0.000					
**ULC027-nobin**	0.059	0.007	0.000	0.000				
**ULC041-bin1**	0.000	0.000	0.000	0.000	0.005			
**ULC041-bin2**	0.000	0.000	0.000	0.000	0.000	0.145		
**ULC041-nobin**	0.000	0.071	0.071	0.000	0.212	0.921	0.496	
**ULC065-bin1**	0.000	0.000	0.000	0.000	0.024	0.000	0.000	0.071
**ULC065-bin2**	0.000	0.000	0.000	0.000	0.000	0.000	0.000	0.000
**ULC065-nobin**	0.005	0.007	19.065	14.226	2.087	0.005	0.097	0.212
**ULC066-bin1**	0.000	0.000	0.000	0.000	0.019	0.000	0.000	0.850
**ULC066-bin2**	0.000	0.000	0.000	0.000	0.000	0.000	0.000	0.000
**ULC066-bin3**	0.000	0.000	0.000	0.000	14.251	0.000	0.000	0.000
**ULC066-nobin**	0.005	36.297	15.940	11.758	3.070	0.005	0.000	0.142
**ULC068-bin1**	0.000	0.000	0.000	0.000	0.011	0.000	0.000	0.354
**ULC068-bin2**	0.000	0.000	0.000	0.000	0.000	0.000	0.000	0.000
**ULC068-nobin**	0.000	3.580	0.114	0.028	1.047	0.000	0.000	0.071
**ULC073-bin1**	0.006	0.000	0.000	0.000	0.006	19.619	7.081	5.312
**ULC073-bin2**	0.000	0.000	0.000	0.000	16.106	0.000	0.000	0.071
**ULC073-bin3**	0.000	0.000	55.001	43.844	7.815	0.000	0.000	0.000
**ULC073-bin4**	0.000	0.000	0.000	0.967	21.787	0.000	0.000	0.071
**ULC073-bin5**	0.000	0.000	0.000	0.000	0.890	4.982	6.495	1.601
**ULC073-bin6**	0.000	0.091	82.743	0.000	3.633	0.000	0.000	0.000
**ULC073-nobin**	0.000	0.007	10.848	19.440	8.979	1.057	1.552	4.462
**ULC077-bin1**	0.000	0.000	0.000	0.000	0.000	0.000	0.000	0.142
**ULC077-nobin**	0.000	0.140	3.296	2.413	2.361	0.000	0.000	0.142
**ULC082-bin1**	0.000	0.000	0.000	0.000	0.000	0.000	0.000	0.000
**ULC082-bin2**	0.000	0.000	0.000	0.000	0.000	0.000	0.000	0.000
**ULC082-bin3**	0.000	0.000	0.000	0.000	0.000	0.000	0.000	0.000
**ULC082-bin4**	0.000	0.000	0.000	0.000	0.000	0.000	0.000	0.000
**ULC082-bin5**	0.000	0.000	0.000	0.000	0.000	0.000	0.000	0.000
**ULC082-nobin**	0.000	0.000	0.000	0.000	0.077	0.000	0.000	0.071
**ULC084-bin1**	0.000	0.000	0.000	0.000	15.489	0.000	0.000	0.071
**ULC084-bin2**	0.000	0.000	0.000	0.000	0.000	0.000	0.000	0.000
**ULC084-bin3**	0.000	0.000	0.000	0.000	0.000	0.000	0.000	0.000
**ULC084-nobin**	0.000	0.000	23.710	19.052	2.571	0.000	0.000	0.071
**ULC129-bin1**	0.016	0.000	0.000	0.000	0.053	0.000	0.000	0.000
**ULC129-nobin**	0.011	24.048	4.455	2.551	1.769	0.005	0.000	0.071
**ULC146-bin1**	0.000	0.000	0.000	0.000	2.796	0.000	0.000	0.071
**ULC146-bin2**	0.000	0.000	0.000	0.000	0.000	0.000	0.000	0.000
**ULC146-bin3**	0.000	0.014	81.733	60.094	6.862	0.000	0.000	0.071
**ULC146-bin4**	0.000	0.000	0.000	0.000	16.060	0.000	0.000	0.071
**ULC146-bin5**	0.000	0.000	0.000	0.000	0.190	0.000	0.000	0.000
**ULC146-bin6**	0.000	0.000	1.798	0.305	36.421	0.000	0.000	0.000
**ULC146-bin7**	0.000	0.000	32.378	38.737	9.987	0.000	0.000	0.000
**ULC146-nobin**	0.000	0.014	1.415	1.248	6.304	0.000	0.000	0.071
**ULC165-bin1**	0.000	0.000	0.000	0.000	0.000	0.000	0.000	0.000
**ULC165-bin2**	0.000	0.000	0.000	0.000	0.000	0.000	0.000	0.000
**ULC165-bin3**	0.000	0.000	0.000	0.000	2.723	0.000	0.000	0.071
**ULC165-bin4**	0.000	0.000	0.000	0.000	0.000	0.000	0.000	0.000
**ULC165-nobin**	0.000	0.007	4.370	2.856	1.153	0.000	0.000	0.071
**ULC179-bin1**	0.000	0.000	0.000	0.000	0.000	0.000	0.000	0.000
**ULC179-bin2**	0.000	0.000	0.000	0.000	0.000	0.000	0.000	0.000
**ULC179-bin3**	0.000	0.000	0.000	0.000	0.007	0.000	0.000	0.000
**ULC179-bin4**	0.000	0.000	0.000	0.000	0.000	0.000	0.000	0.000
**ULC179-bin5**	0.000	0.000	0.000	0.000	0.000	0.000	0.000	0.000
**ULC179-bin6**	0.000	0.000	0.000	0.000	0.000	0.000	0.000	0.000
**ULC179-bin7**	0.000	0.000	0.000	0.000	0.000	0.000	0.000	0.000
**ULC179-nobin**	0.000	0.014	0.019	0.000	0.007	0.000	0.000	0.071
**ULC186-bin1**	0.000	0.000	0.000	0.000	0.000	0.000	0.000	0.000
**ULC186-nobin**	0.000	0.000	0.000	0.000	0.022	0.000	0.000	0.212
**ULC187-bin1**	0.000	0.000	0.000	0.000	0.000	0.000	0.000	0.000
**ULC187-nobin**	0.000	0.000	15.438	10.261	3.327	0.010	0.000	0.071

	ULC065-bin1	ULC065-bin2	ULC065-nobin	ULC066-bin1	ULC066-bin2	ULC066-bin3	ULC066-nobin	ULC068-bin1
**ULC335-bin1**								
**ULC335-bin2**								
**ULC335-bin3**								
**ULC335-bin4**								
**ULC335-nobin**								
**ULC007-bin1**								
**ULC007-bin2**								
**ULC007-nobin**								
**ULC027-bin1**								
**ULC027-bin2**								
**ULC027-bin3**								
**ULC027-bin4**								
**ULC027-nobin**								
**ULC041-bin1**								
**ULC041-bin2**								
**ULC041-nobin**								
**ULC065-bin1**								
**ULC065-bin2**	0.000							
**ULC065-nobin**	0.016	0.000						
**ULC066-bin1**	0.000	0.000	0.061					
**ULC066-bin2**	0.000	0.000	0.000	0.000				
**ULC066-bin3**	0.000	0.000	0.000	0.000	0.000			
**ULC066-nobin**	0.016	0.000	3.254	0.023	0.020	0.000		
**ULC068-bin1**	0.000	0.000	0.017	0.000	0.000	0.000	0.000	
**ULC068-bin2**	0.000	0.000	0.000	0.000	0.000	0.000	0.000	0.369
**ULC068-nobin**	0.025	14.313	3.597	0.051	0.010	0.000	2.103	0.068
**ULC073-bin1**	0.000	0.000	0.000	0.000	0.000	0.000	0.006	0.000
**ULC073-bin2**	0.000	0.000	0.015	0.000	0.000	76.525	46.873	0.000
**ULC073-bin3**	0.000	0.000	14.180	0.000	0.000	0.000	11.449	0.000
**ULC073-bin4**	0.000	0.000	0.092	0.000	0.000	0.000	0.138	0.000
**ULC073-bin5**	0.000	0.000	0.000	0.000	0.000	0.801	2.046	0.000
**ULC073-bin6**	0.000	0.000	25.704	0.000	0.000	0.000	23.161	0.000
**ULC073-nobin**	0.024	0.000	2.161	0.037	0.000	1.367	2.653	0.000
**ULC077-bin1**	0.000	0.000	0.005	0.000	0.000	0.000	0.005	0.000
**ULC077-nobin**	0.000	30.895	2.068	0.037	0.000	29.283	9.116	0.017
**ULC082-bin1**	0.000	0.000	0.000	0.000	0.000	0.000	0.000	0.000
**ULC082-bin2**	0.000	0.000	0.022	0.000	0.000	0.000	0.000	0.000
**ULC082-bin3**	0.000	0.000	0.000	0.000	0.000	0.000	0.000	0.000
**ULC082-bin4**	0.000	0.000	0.000	0.000	0.000	0.000	0.000	0.000
**ULC082-bin5**	0.000	0.000	0.000	0.000	0.000	0.000	0.000	0.000
**ULC082-nobin**	0.000	0.000	0.025	0.000	0.000	0.000	0.006	0.000
**ULC084-bin1**	0.000	0.000	0.017	0.000	0.000	66.081	44.261	0.000
**ULC084-bin2**	0.000	0.000	7.492	0.000	0.000	0.000	0.000	0.000
**ULC084-bin3**	0.000	0.000	0.000	0.000	0.000	0.000	0.000	0.000
**ULC084-nobin**	0.000	0.000	2.723	0.000	0.000	5.661	6.817	0.000
**ULC129-bin1**	0.000	0.000	0.000	0.000	0.000	0.000	0.000	0.000
**ULC129-nobin**	0.000	0.010	0.667	0.000	0.000	20.400	8.685	0.000
**ULC146-bin1**	0.000	0.000	9.519	0.000	0.000	0.000	1.054	0.000
**ULC146-bin2**	0.000	0.000	0.000	0.000	0.000	0.000	0.000	0.000
**ULC146-bin3**	0.000	0.000	18.905	0.000	0.000	0.000	15.535	0.000
**ULC146-bin4**	0.000	0.000	0.015	0.000	0.000	75.549	46.542	0.000
**ULC146-bin5**	0.000	0.000	0.000	0.000	0.000	0.000	0.000	0.000
**ULC146-bin6**	0.000	0.000	0.083	0.000	0.000	0.000	0.028	0.000
**ULC146-bin7**	0.000	0.000	16.480	0.000	0.000	0.000	13.793	0.000
**ULC146-nobin**	0.000	0.000	1.057	0.000	0.000	2.001	1.503	0.000
**ULC165-bin1**	0.000	91.192	9.267	0.000	0.000	0.000	0.000	0.000
**ULC165-bin2**	0.000	0.000	7.601	0.000	0.000	0.000	0.000	0.000
**ULC165-bin3**	0.000	0.000	9.389	0.000	0.000	0.000	1.002	0.000
**ULC165-bin4**	0.000	0.000	0.041	0.000	0.000	0.000	0.000	0.000
**ULC165-nobin**	0.000	0.747	2.785	0.000	0.000	0.000	0.414	0.006
**ULC179-bin1**	0.000	0.000	0.000	0.000	0.000	0.000	0.000	0.000
**ULC179-bin2**	0.000	0.000	0.000	0.000	0.000	0.000	0.000	0.000
**ULC179-bin3**	0.000	0.000	0.007	0.000	0.000	0.000	0.000	0.000
**ULC179-bin4**	0.000	0.000	0.000	0.000	0.000	0.000	0.000	0.000
**ULC179-bin5**	0.000	0.000	0.000	0.000	0.000	0.000	0.000	0.000
**ULC179-bin6**	0.000	0.000	0.000	0.000	0.000	0.000	0.000	0.000
**ULC179-bin7**	0.000	0.000	0.000	0.000	0.000	0.000	0.000	0.000
**ULC179-nobin**	0.000	0.000	0.014	0.000	0.000	0.000	0.014	0.000
**ULC186-bin1**	0.000	0.000	0.000	0.000	0.000	0.000	0.000	0.000
**ULC186-nobin**	0.000	0.000	0.022	0.011	0.000	0.049	0.022	0.000
**ULC187-bin1**	0.000	0.000	0.000	0.000	0.000	0.000	0.000	0.000
**ULC187-nobin**	0.000	0.010	2.860	0.000	0.000	0.000	1.978	0.000

	ULC068-bin2	ULC068-nobin	ULC073-bin1	ULC073-bin2	ULC073-bin3	ULC073-bin4	ULC073-bin5	ULC073-bin6
**ULC335-bin1**								
**ULC335-bin2**								
**ULC335-bin3**								
**ULC335-bin4**								
**ULC335-nobin**								
**ULC007-bin1**								
**ULC007-bin2**								
**ULC007-nobin**								
**ULC027-bin1**								
**ULC027-bin2**								
**ULC027-bin3**								
**ULC027-bin4**								
**ULC027-nobin**								
**ULC041-bin1**								
**ULC041-bin2**								
**ULC041-nobin**								
**ULC065-bin1**								
**ULC065-bin2**								
**ULC065-nobin**								
**ULC066-bin1**								
**ULC066-bin2**								
**ULC066-bin3**								
**ULC066-nobin**								
**ULC068-bin1**								
**ULC068-bin2**								
**ULC068-nobin**	0.000							
**ULC073-bin1**	0.000	0.000						
**ULC073-bin2**	0.000	0.084	0.000					
**ULC073-bin3**	0.000	0.034	0.000	0.000				
**ULC073-bin4**	0.000	0.000	0.000	0.000	0.000			
**ULC073-bin5**	0.000	0.000	0.267	0.000	0.000	0.000		
**ULC073-bin6**	0.000	1.181	0.000	0.000	0.000	0.000	0.000	
**ULC073-nobin**	0.000	1.385	0.083	0.038	0.000	0.000	0.089	0.000
**ULC077-bin1**	0.000	0.025	0.000	0.000	0.000	0.000	0.000	0.000
**ULC077-nobin**	0.000	4.205	0.000	37.470	2.328	0.276	2.046	5.904
**ULC082-bin1**	0.000	0.000	0.000	0.000	0.000	0.000	0.000	0.000
**ULC082-bin2**	0.000	0.011	0.000	0.000	0.000	0.000	0.000	0.000
**ULC082-bin3**	0.000	0.000	0.000	0.000	0.000	0.000	0.000	0.000
**ULC082-bin4**	0.000	0.000	0.000	0.000	0.000	0.000	0.000	0.000
**ULC082-bin5**	0.000	0.000	0.000	0.000	0.000	0.000	0.000	0.000
**ULC082-nobin**	0.000	0.017	0.000	0.000	0.000	0.000	0.000	0.000
**ULC084-bin1**	0.000	0.068	0.000	89.160	0.000	0.000	0.000	0.000
**ULC084-bin2**	0.000	2.656	0.000	0.000	0.000	0.000	0.000	0.000
**ULC084-bin3**	0.000	0.000	0.000	0.000	0.000	0.000	0.000	0.000
**ULC084-nobin**	0.000	0.135	0.000	8.430	17.515	0.000	3.826	32.243
**ULC129-bin1**	0.000	0.000	0.000	0.000	0.000	0.000	0.000	0.000
**ULC129-nobin**	0.000	1.191	0.000	25.784	3.609	1.612	1.246	8.174
**ULC146-bin1**	0.000	0.144	0.000	0.000	0.000	0.000	0.000	0.000
**ULC146-bin2**	0.000	0.000	0.000	0.000	0.000	0.000	0.000	0.000
**ULC146-bin3**	0.000	0.042	0.000	0.000	61.553	0.230	0.000	78.202
**ULC146-bin4**	0.000	0.084	0.000	92.403	0.000	0.000	0.000	0.000
**ULC146-bin5**	0.000	0.000	0.000	0.000	0.000	0.000	0.000	0.000
**ULC146-bin6**	0.000	0.028	0.000	0.000	0.581	0.092	0.000	0.000
**ULC146-bin7**	0.000	0.000	0.000	0.000	54.322	0.000	0.000	0.000
**ULC146-nobin**	0.000	1.816	0.000	1.978	2.667	11.792	4.270	11.444
**ULC165-bin1**	0.000	15.352	0.000	0.000	0.000	0.000	0.000	0.000
**ULC165-bin2**	0.000	2.706	0.000	0.000	0.000	0.000	0.000	0.000
**ULC165-bin3**	0.000	0.103	0.000	0.000	0.000	0.000	0.000	0.000
**ULC165-bin4**	0.000	0.000	0.000	0.000	0.000	0.000	0.000	0.000
**ULC165-nobin**	0.000	0.498	0.000	0.000	3.875	4.975	0.000	8.356
**ULC179-bin1**	0.000	0.000	0.000	0.000	0.000	0.000	0.000	0.000
**ULC179-bin2**	0.000	0.000	0.000	0.000	0.000	0.000	0.000	0.000
**ULC179-bin3**	0.000	0.000	0.000	0.000	0.000	0.000	0.000	0.000
**ULC179-bin4**	0.000	0.000	0.000	0.000	0.000	0.000	0.000	0.000
**ULC179-bin5**	0.000	0.000	0.000	0.000	0.000	0.000	0.000	0.000
**ULC179-bin6**	0.000	0.000	0.000	0.000	0.000	0.000	0.000	0.000
**ULC179-bin7**	0.000	0.000	0.000	0.000	0.000	0.000	0.000	0.000
**ULC179-nobin**	0.000	0.008	0.000	0.000	0.008	0.000	0.000	0.091
**ULC186-bin1**	0.000	0.000	0.000	0.000	0.000	0.000	0.000	0.000
**ULC186-nobin**	0.000	0.011	0.000	0.011	0.000	0.000	0.000	0.000
**ULC187-bin1**	0.123	0.000	0.000	0.000	0.000	0.000	0.000	0.000
**ULC187-nobin**	0.000	0.771	0.000	0.010	13.703	4.376	0.000	21.072

	ULC073-nobin	ULC077-bin1	ULC077-nobin	ULC082-bin1	ULC082-bin2	ULC082-bin3	ULC082-bin4	ULC082-bin5
**ULC335-bin1**								
**ULC335-bin2**								
**ULC335-bin3**								
**ULC335-bin4**								
**ULC335-nobin**								
**ULC007-bin1**								
**ULC007-bin2**								
**ULC007-nobin**								
**ULC027-bin1**								
**ULC027-bin2**								
**ULC027-bin3**								
**ULC027-bin4**								
**ULC027-nobin**								
**ULC041-bin1**								
**ULC041-bin2**								
**ULC041-nobin**								
**ULC065-bin1**								
**ULC065-bin2**								
**ULC065-nobin**								
**ULC066-bin1**								
**ULC066-bin2**								
**ULC066-bin3**								
**ULC066-nobin**								
**ULC068-bin1**								
**ULC068-bin2**								
**ULC068-nobin**								
**ULC073-bin1**								
**ULC073-bin2**								
**ULC073-bin3**								
**ULC073-bin4**								
**ULC073-bin5**								
**ULC073-bin6**								
**ULC073-nobin**								
**ULC077-bin1**	0.000							
**ULC077-nobin**	1.473	0.970						
**ULC082-bin1**	0.000	0.000	0.000					
**ULC082-bin2**	0.000	0.000	0.044	0.000				
**ULC082-bin3**	0.000	0.000	0.000	0.000	0.000			
**ULC082-bin4**	0.000	0.000	0.187	0.000	0.000	0.000		
**ULC082-bin5**	0.000	0.000	0.000	0.000	0.000	0.000	0.000	
**ULC082-nobin**	0.021	0.000	0.031	0.000	0.000	0.000	0.000	0.000
**ULC084-bin1**	1.286	0.000	35.431	0.000	0.000	0.000	0.000	0.000
**ULC084-bin2**	0.000	0.000	8.952	0.000	0.229	0.000	1.185	0.000
**ULC084-bin3**	0.000	0.000	0.000	21.352	0.000	0.000	0.000	0.000
**ULC084-nobin**	5.004	0.000	4.569	0.424	0.044	0.000	0.000	0.000
**ULC129-bin1**	0.000	0.000	0.000	0.000	0.000	0.000	0.000	0.000
**ULC129-nobin**	1.846	0.000	3.626	0.009	0.000	0.000	0.000	0.000
**ULC146-bin1**	8.131	0.000	0.247	0.000	0.000	0.000	0.000	0.000
**ULC146-bin2**	0.000	0.000	0.000	0.000	0.000	0.000	0.000	0.000
**ULC146-bin3**	16.232	0.000	3.169	0.000	0.000	0.000	0.000	0.000
**ULC146-bin4**	1.631	0.000	37.130	0.000	0.000	0.000	0.000	0.000
**ULC146-bin5**	0.000	0.000	0.022	0.000	0.000	0.000	0.000	0.000
**ULC146-bin6**	28.042	0.000	0.249	0.000	0.000	0.000	0.000	0.000
**ULC146-bin7**	18.451	0.000	2.732	0.000	0.000	0.000	0.000	0.000
**ULC146-nobin**	6.834	0.000	1.420	0.009	0.000	0.000	0.000	0.000
**ULC165-bin1**	0.000	0.000	31.584	0.000	0.000	0.000	0.000	0.000
**ULC165-bin2**	0.000	0.000	9.015	0.000	0.283	0.000	1.247	0.000
**ULC165-bin3**	8.490	0.000	0.257	0.000	0.000	0.000	0.000	0.000
**ULC165-bin4**	0.021	0.000	0.000	0.000	0.000	0.000	0.000	0.000
**ULC165-nobin**	2.159	0.000	0.555	0.009	0.000	0.000	0.000	0.000
**ULC179-bin1**	0.000	0.000	0.000	0.000	0.000	0.000	0.000	0.000
**ULC179-bin2**	0.000	0.000	0.000	0.000	0.000	0.000	0.000	0.000
**ULC179-bin3**	0.000	0.000	0.000	0.000	0.000	0.000	0.000	0.000
**ULC179-bin4**	0.000	0.000	0.000	0.000	0.000	0.000	0.000	0.000
**ULC179-bin5**	0.000	0.000	0.000	0.000	0.000	0.000	0.000	0.000
**ULC179-bin6**	0.000	0.000	0.000	0.000	0.000	0.000	0.000	0.000
**ULC179-bin7**	0.000	0.000	0.000	0.000	0.000	0.000	0.000	0.000
**ULC179-nobin**	0.007	0.000	0.009	0.000	0.000	0.000	0.000	0.000
**ULC186-bin1**	0.000	0.000	0.000	0.000	0.000	0.000	0.000	0.000
**ULC186-nobin**	0.032	0.000	0.022	0.000	0.000	0.026	0.000	0.000
**ULC187-bin1**	0.000	0.000	0.000	0.000	0.000	0.000	0.000	0.000
**ULC187-nobin**	6.441	0.000	0.882	0.010	0.022	0.000	0.125	0.000

	ULC082-nobin	ULC084-bin1	ULC084-bin2	ULC084-bin3	ULC084-nobin	ULC129-bin1	ULC129-nobin	ULC146-bin1
**ULC335-bin1**								
**ULC335-bin2**								
**ULC335-bin3**								
**ULC335-bin4**								
**ULC335-nobin**								
**ULC007-bin1**								
**ULC007-bin2**								
**ULC007-nobin**								
**ULC027-bin1**								
**ULC027-bin2**								
**ULC027-bin3**								
**ULC027-bin4**								
**ULC027-nobin**								
**ULC041-bin1**								
**ULC041-bin2**								
**ULC041-nobin**								
**ULC065-bin1**								
**ULC065-bin2**								
**ULC065-nobin**								
**ULC066-bin1**								
**ULC066-bin2**								
**ULC066-bin3**								
**ULC066-nobin**								
**ULC068-bin1**								
**ULC068-bin2**								
**ULC068-nobin**								
**ULC073-bin1**								
**ULC073-bin2**								
**ULC073-bin3**								
**ULC073-bin4**								
**ULC073-bin5**								
**ULC073-bin6**								
**ULC073-nobin**								
**ULC077-bin1**								
**ULC077-nobin**								
**ULC082-bin1**								
**ULC082-bin2**								
**ULC082-bin3**								
**ULC082-bin4**								
**ULC082-bin5**								
**ULC082-nobin**								
**ULC084-bin1**	0.000							
**ULC084-bin2**	0.044	0.000						
**ULC084-bin3**	0.313	0.000	0.000					
**ULC084-nobin**	0.297	0.017	0.000	0.018				
**ULC129-bin1**	0.000	0.000	0.000	0.000	0.000			
**ULC129-nobin**	0.063	24.625	0.009	0.009	3.356	0.053		
**ULC146-bin1**	0.010	0.000	0.000	0.000	0.000	0.000	6.512	
**ULC146-bin2**	0.000	0.000	0.000	0.000	0.000	0.000	0.000	0.000
**ULC146-bin3**	0.000	0.000	0.000	0.000	24.100	0.000	4.124	0.000
**ULC146-bin4**	0.000	88.752	0.000	0.000	7.705	0.000	25.703	0.000
**ULC146-bin5**	0.045	0.000	0.000	0.000	0.022	0.000	0.000	0.000
**ULC146-bin6**	0.000	0.000	0.000	0.000	0.000	0.000	0.000	0.000
**ULC146-bin7**	0.000	0.000	0.000	0.000	21.093	0.000	2.956	0.000
**ULC146-nobin**	0.178	0.971	0.000	0.000	3.105	0.000	1.397	0.041
**ULC165-bin1**	0.000	0.000	0.000	0.000	0.000	0.000	0.008	0.000
**ULC165-bin2**	0.138	0.000	87.311	0.000	3.215	0.000	0.009	0.000
**ULC165-bin3**	0.000	0.000	0.000	0.000	0.000	0.000	6.358	84.601
**ULC165-bin4**	0.021	0.000	0.000	0.000	0.000	0.000	0.041	0.000
**ULC165-nobin**	0.109	0.000	0.871	0.009	1.661	0.005	1.662	35.053
**ULC179-bin1**	0.000	0.000	0.000	0.000	0.000	0.000	0.000	0.000
**ULC179-bin2**	0.000	0.000	0.000	0.000	0.000	0.000	0.000	0.000
**ULC179-bin3**	0.000	0.000	0.000	0.000	0.000	0.000	0.000	0.000
**ULC179-bin4**	0.000	0.000	0.000	0.000	0.000	0.000	0.000	0.000
**ULC179-bin5**	0.000	0.000	0.000	0.000	0.000	0.000	0.000	0.000
**ULC179-bin6**	0.000	0.000	0.000	0.000	0.000	0.000	0.000	0.000
**ULC179-bin7**	0.000	0.000	0.000	0.000	0.000	0.000	0.000	0.000
**ULC179-nobin**	0.014	0.000	0.000	0.000	0.026	0.000	0.011	0.005
**ULC186-bin1**	0.030	0.000	0.000	0.000	0.020	0.000	0.030	0.000
**ULC186-nobin**	0.194	0.011	0.011	0.022	0.183	0.000	0.172	0.011
**ULC187-bin1**	0.017	0.000	0.000	0.000	0.020	0.000	0.017	0.000
**ULC187-nobin**	0.122	0.010	8.723	0.020	3.175	0.000	1.177	0.000

	ULC146-bin2	ULC146-bin3	ULC146-bin4	ULC146-bin5	ULC146-bin6	ULC146-bin7	ULC146-nobin	ULC165-bin1
**ULC335-bin1**								
**ULC335-bin2**								
**ULC335-bin3**								
**ULC335-bin4**								
**ULC335-nobin**								
**ULC007-bin1**								
**ULC007-bin2**								
**ULC007-nobin**								
**ULC027-bin1**								
**ULC027-bin2**								
**ULC027-bin3**								
**ULC027-bin4**								
**ULC027-nobin**								
**ULC041-bin1**								
**ULC041-bin2**								
**ULC041-nobin**								
**ULC065-bin1**								
**ULC065-bin2**								
**ULC065-nobin**								
**ULC066-bin1**								
**ULC066-bin2**								
**ULC066-bin3**								
**ULC066-nobin**								
**ULC068-bin1**								
**ULC068-bin2**								
**ULC068-nobin**								
**ULC073-bin1**								
**ULC073-bin2**								
**ULC073-bin3**								
**ULC073-bin4**								
**ULC073-bin5**								
**ULC073-bin6**								
**ULC073-nobin**								
**ULC077-bin1**								
**ULC077-nobin**								
**ULC082-bin1**								
**ULC082-bin2**								
**ULC082-bin3**								
**ULC082-bin4**								
**ULC082-bin5**								
**ULC082-nobin**								
**ULC084-bin1**								
**ULC084-bin2**								
**ULC084-bin3**								
**ULC084-nobin**								
**ULC129-bin1**								
**ULC129-nobin**								
**ULC146-bin1**								
**ULC146-bin2**								
**ULC146-bin3**	0.000							
**ULC146-bin4**	0.000	0.000						
**ULC146-bin5**	0.000	0.000	0.000					
**ULC146-bin6**	0.000	0.000	0.000	0.000				
**ULC146-bin7**	0.000	0.000	0.000	0.000	0.000			
**ULC146-nobin**	0.006	0.007	0.077	0.000	0.000	0.000		
**ULC165-bin1**	0.000	0.000	0.000	0.000	0.000	0.000	0.000	
**ULC165-bin2**	0.000	0.000	0.000	0.000	0.000	0.000	0.000	0.000
**ULC165-bin3**	0.000	0.000	0.000	0.000	0.000	0.000	0.437	0.000
**ULC165-bin4**	0.000	0.000	0.000	0.000	0.000	0.000	0.267	0.000
**ULC165-nobin**	0.006	4.124	0.000	0.168	4.342	3.269	1.334	0.016
**ULC179-bin1**	0.000	0.000	0.000	0.000	0.000	0.000	0.000	0.000
**ULC179-bin2**	0.000	0.000	0.000	0.000	0.000	0.000	6.177	0.000
**ULC179-bin3**	0.000	0.000	0.000	0.000	0.000	0.000	0.007	0.000
**ULC179-bin4**	0.000	0.000	0.000	0.000	0.000	0.000	0.000	0.000
**ULC179-bin5**	0.000	0.000	0.000	0.000	0.000	0.000	0.172	0.000
**ULC179-bin6**	0.000	0.000	0.000	0.000	0.000	0.000	2.205	0.000
**ULC179-bin7**	0.000	0.000	0.000	0.000	0.000	0.000	0.053	0.000
**ULC179-nobin**	0.006	0.022	0.000	0.000	0.000	0.000	0.735	0.000
**ULC186-bin1**	0.000	0.000	0.000	0.000	0.000	0.000	0.015	0.000
**ULC186-nobin**	0.000	0.000	0.000	0.000	0.000	0.000	0.205	0.000
**ULC187-bin1**	0.000	0.000	0.000	0.000	0.000	0.000	0.017	0.000
**ULC187-nobin**	0.010	19.860	0.010	0.011	0.332	11.464	2.475	0.010

	ULC165-bin2	ULC165-bin3	ULC165-bin4	ULC165-nobin	ULC179-bin1	ULC179-bin2	ULC179-bin3	ULC179-bin4
**ULC335-bin1**								
**ULC335-bin2**								
**ULC335-bin3**								
**ULC335-bin4**								
**ULC335-nobin**								
**ULC007-bin1**								
**ULC007-bin2**								
**ULC007-nobin**								
**ULC027-bin1**								
**ULC027-bin2**								
**ULC027-bin3**								
**ULC027-bin4**								
**ULC027-nobin**								
**ULC041-bin1**								
**ULC041-bin2**								
**ULC041-nobin**								
**ULC065-bin1**								
**ULC065-bin2**								
**ULC065-nobin**								
**ULC066-bin1**								
**ULC066-bin2**								
**ULC066-bin3**								
**ULC066-nobin**								
**ULC068-bin1**								
**ULC068-bin2**								
**ULC068-nobin**								
**ULC073-bin1**								
**ULC073-bin2**								
**ULC073-bin3**								
**ULC073-bin4**								
**ULC073-bin5**								
**ULC073-bin6**								
**ULC073-nobin**								
**ULC077-bin1**								
**ULC077-nobin**								
**ULC082-bin1**								
**ULC082-bin2**								
**ULC082-bin3**								
**ULC082-bin4**								
**ULC082-bin5**								
**ULC082-nobin**								
**ULC084-bin1**								
**ULC084-bin2**								
**ULC084-bin3**								
**ULC084-nobin**								
**ULC129-bin1**								
**ULC129-nobin**								
**ULC146-bin1**								
**ULC146-bin2**								
**ULC146-bin3**								
**ULC146-bin4**								
**ULC146-bin5**								
**ULC146-bin6**								
**ULC146-bin7**								
**ULC146-nobin**								
**ULC165-bin1**								
**ULC165-bin2**								
**ULC165-bin3**	0.000							
**ULC165-bin4**	0.000	0.000						
**ULC165-nobin**	0.009	0.000	0.000					
**ULC179-bin1**	0.000	0.000	0.000	0.000				
**ULC179-bin2**	0.000	0.000	0.000	0.000	0.000			
**ULC179-bin3**	0.000	0.000	0.000	0.000	0.000	0.000		
**ULC179-bin4**	0.000	0.000	0.000	0.000	0.000	0.000	0.000	
**ULC179-bin5**	0.000	0.000	0.000	0.000	0.057	0.000	0.000	0.000
**ULC179-bin6**	0.000	0.000	0.000	0.000	0.000	0.000	0.000	0.000
**ULC179-bin7**	0.000	0.000	0.000	0.000	0.000	0.000	0.000	0.000
**ULC179-nobin**	0.000	0.000	0.000	0.021	0.005	0.005	0.033	0.000
**ULC186-bin1**	0.000	0.000	0.000	0.020	0.000	0.000	0.000	0.000
**ULC186-nobin**	0.011	0.000	0.000	0.259	0.000	0.000	0.000	0.000
**ULC187-bin1**	0.000	0.000	0.000	0.011	0.000	0.000	0.000	0.000
**ULC187-nobin**	9.058	0.000	0.000	0.700	0.000	0.000	0.000	0.000

	ULC179-bin5	ULC179-bin6	ULC179-bin7	ULC179-nobin	ULC186-bin1	ULC186-nobin	ULC187-bin1	ULC187-nobin
**ULC335-bin1**								
**ULC335-bin2**								
**ULC335-bin3**								
**ULC335-bin4**								
**ULC335-nobin**								
**ULC007-bin1**								
**ULC007-bin2**								
**ULC007-nobin**								
**ULC027-bin1**								
**ULC027-bin2**								
**ULC027-bin3**								
**ULC027-bin4**								
**ULC027-nobin**								
**ULC041-bin1**								
**ULC041-bin2**								
**ULC041-nobin**								
**ULC065-bin1**								
**ULC065-bin2**								
**ULC065-nobin**								
**ULC066-bin1**								
**ULC066-bin2**								
**ULC066-bin3**								
**ULC066-nobin**								
**ULC068-bin1**								
**ULC068-bin2**								
**ULC068-nobin**								
**ULC073-bin1**								
**ULC073-bin2**								
**ULC073-bin3**								
**ULC073-bin4**								
**ULC073-bin5**								
**ULC073-bin6**								
**ULC073-nobin**								
**ULC077-bin1**								
**ULC077-nobin**								
**ULC082-bin1**								
**ULC082-bin2**								
**ULC082-bin3**								
**ULC082-bin4**								
**ULC082-bin5**								
**ULC082-nobin**								
**ULC084-bin1**								
**ULC084-bin2**								
**ULC084-bin3**								
**ULC084-nobin**								
**ULC129-bin1**								
**ULC129-nobin**								
**ULC146-bin1**								
**ULC146-bin2**								
**ULC146-bin3**								
**ULC146-bin4**								
**ULC146-bin5**								
**ULC146-bin6**								
**ULC146-bin7**								
**ULC146-nobin**								
**ULC165-bin1**								
**ULC165-bin2**								
**ULC165-bin3**								
**ULC165-bin4**								
**ULC165-nobin**								
**ULC179-bin1**								
**ULC179-bin2**								
**ULC179-bin3**								
**ULC179-bin4**								
**ULC179-bin5**								
**ULC179-bin6**	0.000							
**ULC179-bin7**	0.000	0.000						
**ULC179-nobin**	0.000	0.000	0.000					
**ULC186-bin1**	0.000	0.000	0.000	0.000				
**ULC186-nobin**	0.000	0.000	0.000	0.022	0.269			
**ULC187-bin1**	0.000	0.000	0.000	0.000	0.000	0.043		
**ULC187-nobin**	0.000	0.000	0.000	0.030	0.101	0.151	0.091	

Salmonella enterica subsp. enterica serovar Enteritidis str. EC20090531 [1412462]
Salmonella enterica subsp. enterica serovar Enteritidis str. EC20110356 [1243621]
Salmonella enterica subsp. enterica serovar Enteritidis str. CDC_2010K_0968 [886715]
Salmonella enterica subsp. enterica serovar Enteritidis str. 77-1427 [926034]
Salmonella enterica subsp. enterica serovar Enteritidis str. EC20110221 [1412455]
Salmonella enterica subsp. enterica serovar Enteritidis str. EC20100130 [1412464]
Salmonella enterica subsp. enterica serovar Enteritidis str. EC20111174 [1244122]
Salmonella enterica subsp. enterica serovar Enteritidis str. EC20090193 [1412459]
Salmonella enterica subsp. enterica serovar Enteritidis str. EC20110361 [1244119]
Salmonella enterica subsp. enterica serovar Enteritidis str. EC20121175 [1412592]
Salmonella enterica subsp. enterica serovar Montevideo str. 507440-20 [859199]
Salmonella enterica subsp. enterica serovar Schwarzengrund str. CVM19633 [439843]
Salmonella enterica subsp. enterica serovar Tennessee str. TXSC_TXSC08-19 [1003191]
Salmonella enterica subsp. enterica serovar Agona str. SL483 [454166]
Salmonella enterica subsp. enterica serovar Agona str. 24249 [1406860]
Salmonella enterica subsp. enterica serovar Enteritidis str. EC20121178 [1412595]
Salmonella enterica subsp. enterica serovar Enteritidis str. SA20094177 [1412479]
Salmonella enterica subsp. enterica serovar Enteritidis str. EC20120008 [1412470]
Salmonella enterica subsp. enterica serovar Enteritidis str. EC20100103 [1412458]
Salmonella enterica subsp. enterica serovar Enteritidis str. EC20110359 [1244113]
Salmonella enterica subsp. enterica serovar Enteritidis str. EC20100134 [1412465]
Salmonella enterica subsp. enterica serovar Enteritidis str. EC20111175 [1244121]
Salmonella enterica subsp. enterica serovar Enteritidis [149539]
Salmonella enterica subsp. enterica serovar Enteritidis str. SA20084824 [1412478]
Salmonella enterica subsp. enterica serovar Enteritidis str. EC20110358 [1244112]
Salmonella enterica subsp. enterica serovar Pullorum str. S06004 [1298917]
Salmonella enterica subsp. enterica serovar Bareilly str. CFSAN000189 [1173427]
Salmonella enterica subsp. enterica serovar Enteritidis str. EC20110354 [1243619]
Citrobacter rodentium ICC168 [637910]
Citrobacter amalonaticus Y19 [1261127]
Citrobacter koseri ATCC BAA-895 [290338]
Citrobacter freundii CFNIH1 [1333848]
Salmonella bongori N268-08 [1197719]
Salmonella enterica subsp. enterica serovar Paratyphi A str. AKU_12601 [554290]
Salmonella enterica subsp. enterica serovar Paratyphi A [54388]
Salmonella enterica subsp. enterica serovar Typhi str. Ty2 [209261]
Salmonella enterica subsp. enterica serovar Choleraesuis str. SC-B67 [321314]
Salmonella enterica subsp. enterica serovar Abony str. 0014 [1029983]
Salmonella enterica subsp. enterica serovar Newport str. CVM 22513 [796732]
Salmonella enterica subsp. enterica serovar Newport str. CVM 21538 [796731]
Salmonella enterica subsp. enterica serovar Newport str. SL254 [423368]
Salmonella enterica subsp. enterica serovar Typhimurium str. ST4/74 [909946]
Salmonella enterica subsp. enterica serovar Newport str. CVM N1543 [858307]
Salmonella enterica subsp. enterica serovar Typhimurium [90371]
Salmonella enterica subsp. enterica [59201]
Salmonella enterica subsp. enterica serovar Typhimurium str. UK-1 [990282]
Salmonella enterica subsp. enterica serovar Typhimurium str. D23580 [568708]
Salmonella enterica subsp. enterica serovar 4,[5],12:i:- str. 08-1736 [866913]
Salmonella enterica subsp. enterica serovar Typhimurium var. 5- str. CFSAN001921 [1271862]
Salmonella enterica subsp. enterica serovar Abaetetuba str. ATCC 35640 [1208611]
Salmonella enterica subsp. enterica serovar Heidelberg str. CFSAN002069 [1271864]
Salmonella enterica subsp. enterica serovar Typhimurium str. CDC 2011K-0870 [1454642]
Salmonella enterica subsp. enterica serovar Anatum str. CDC 06-0532 [1454592]
Salmonella enterica subsp. enterica serovar Montevideo str. USDA-ARS-USMARC-1903 [1454603]
Salmonella enterica subsp. enterica serovar Newport str. USMARC-S3124.1 [877468]
Salmonella enterica subsp. enterica serovar Newport str. CDC 2010K-2159 [1454627]
Salmonella enterica subsp. enterica serovar Paratyphi C str. RKS4594 [476213]
Salmonella enterica subsp. enterica serovar Paratyphi B str. SPB7 [1016998]
Salmonella enterica subsp. enterica serovar Typhimurium str. USDA-ARS-USMARC-1899 [1454639]
Salmonella enterica subsp. enterica serovar Typhimurium str. 798 [1008297]
Salmonella enterica subsp. enterica serovar Anatum str. USDA-ARS-USMARC-1175 [1454590]
Salmonella enterica subsp. enterica serovar Anatum str. USDA-ARS-USMARC-1735 [1454585]
Salmonella enterica subsp. enterica serovar Montevideo str. CDC 86-0391 [1454606]
Salmonella enterica subsp. enterica serovar Newport str. USDA-ARS-USMARC-1927 [1454620]
Salmonella enterica subsp. enterica serovar Gallinarum/pullorum str. CDC1983-67 [1225522]
Salmonella enterica subsp. enterica serovar Gallinarum/pullorum str. RKS5078 [1081093]
Salmonella enterica subsp. enterica serovar Typhi str. P-stx-12 [1132507]
Salmonella enterica subsp. enterica serovar Infantis [595]
Salmonella enterica subsp. enterica serovar Typhimurium str. L-3553 [996633]
Salmonella enterica subsp. enterica serovar Heidelberg str. B182 [1160717]
Salmonella enterica subsp. enterica serovar Newport str. CVM 21550 [796733]
Salmonella enterica subsp. enterica serovar Heidelberg str. 41578 [1124936]
Salmonella enterica subsp. enterica serovar Newport str. CVM N18486 [858306]
Salmonella enterica subsp. enterica serovar Heidelberg str. CFSAN002064 [1299044]
Salmonella enterica subsp. enterica serovar Typhimurium str. 14028S [588858]
Salmonella enterica subsp. enterica serovar Heidelberg str. SL476 [454169]
Salmonella enterica subsp. enterica serovar Typhimurium str. LT2 [99287]
Salmonella enterica subsp. enterica serovar Typhimurium str. DT2 [568709]
Salmonella enterica subsp. enterica serovar Enteritidis str. P125109 [550537]
Salmonella enterica subsp. enterica serovar Typhimurium str. SL1344 [216597]
Salmonella enterica subsp. enterica serovar Typhimurium str. DT104 [85569]
Salmonella enterica subsp. enterica serovar Typhi str. Ty21a [527001]
Salmonella enterica subsp. enterica serovar Cubana str. CFSAN002050 [1271863]

Salmonella enterica subsp. enterica serovar Thompson str. RM6836 [1064551]
Salmonella enterica subsp. enterica serovar Enteritidis str. EC20121179 [1412596]
Salmonella enterica subsp. enterica serovar Enteritidis str. EC20121176 [1412593]
Salmonella enterica subsp. enterica serovar Enteritidis str. EC20121180 [1412597]
Salmonella enterica subsp. enterica serovar Thompson str. ATCC 8391 [935705]
Salmonella enterica subsp. enterica serovar Dublin str. CT_02021853 [439851]
Salmonella enterica subsp. enterica serovar Enteritidis str. EC20090641 [1412453]
Salmonella enterica subsp. enterica serovar Enteritidis str. EC20111095 [1244120]
Salmonella enterica subsp. enterica serovar Enteritidis str. EC20090135 [1412460]
Salmonella enterica subsp. enterica serovar Anatum str. ATCC BAA-1592 [984211]
Salmonella enterica subsp. enterica serovar Javiana str. CFSAN001992 [1267753]
Salmonella enterica subsp. enterica serovar Agona str. 460004 2-1 [1124917]
Salmonella enterica subsp. enterica serovar Bredeney str. CFSAN001080 [1194154]
Salmonella enterica subsp. enterica serovar Enteritidis str. SA20094301 [1412505]
Salmonella enterica subsp. enterica serovar Enteritidis str. EC20090332 [1412461]
Salmonella enterica subsp. enterica serovar Enteritidis str. EC20120009 [1412471]
Salmonella enterica subsp. enterica serovar Enteritidis str. EC20110357 [1244111]
Salmonella enterica subsp. enterica serovar Enteritidis str. EC20120005 [1412468]
Salmonella enterica subsp. enterica serovar Enteritidis str. EC20090698 [1412454]
Salmonella enterica subsp. enterica serovar Enteritidis str. EC20110360 [1244118]
Salmonella enterica subsp. enterica serovar Enteritidis str. SA20093266 [1412475]
Salmonella enterica subsp. enterica serovar Enteritidis str. EC20120929 [1412618]
Salmonella enterica subsp. enterica serovar Enteritidis str. EC20100101 [1412457]
Salmonella enterica subsp. enterica serovar Enteritidis str. EC20110223 [1412451]
Salmonella enterica subsp. enterica serovar Enteritidis str. EC20090884 [1412463]
Salmonella enterica subsp. enterica serovar Enteritidis str. EC20110355 [1243620]
Salmonella enterica subsp. enterica serovar Enteritidis str. EC20120916 [1412570]
Salmonella enterica subsp. enterica serovar Enteritidis str. 18569 [696867]
Salmonella enterica subsp. enterica serovar Gallinarum str. 287/91 [550538]
Salmonella enterica subsp. enterica serovar Enteritidis str. EC20110353 [1243618]
Salmonella enterica subsp. enterica serovar Enteritidis str. SA20084644 [1412480]
Salmonella enterica subsp. enterica serovar Typhimurium str. U288 [1171376]
Salmonella enterica subsp. enterica serovar Newport str. CVM 22425 [796730]
Salmonella enterica subsp. enterica serovar Newport str. CVM 22462 [858305]
Salmonella enterica subsp. enterica serovar Typhimurium str. T000240 [718274]
Salmonella enterica subsp. enterica serovar Paratyphi A str. ATCC 9150 [295319]
Salmonella enterica subsp. enterica serovar Typhi str. CT18 [220341]
Salmonella enterica subsp. enterica serovar Choleraesuis [119912]
Salmonella bongori NCTC 12419 [218493]
Salmonella bongori serovar 48:z41:-- str. RKS3044 [1382510]
Salmonella enterica subsp. arizonae serovar 62:z4,z23:- [41514]
Salmonella enterica subsp. arizonae serovar 62:z36:- str. RKS2983 [1386967]
Escherichia coli O104:H4 str. 2011C-3493 [1133852]
Escherichia coli O104:H4 str. C227-11 [1048254]
Escherichia coli 55989 [585055]
Shigella sonnei [624]
Shigella boydii Sb227 [300268]
Escherichia coli IAI1 [585034]
Escherichia coli APEC O78 [1274814]
Escherichia coli SE11 [409438]
Escherichia coli O139:H28 str. E24377A [331111]
Escherichia coli W [566546]
Escherichia coli KO11FL [595495]
Escherichia coli O145:H28 str. RM13516 [1248915]
Escherichia coli O145:H28 str. RM12761 [1248903]
Escherichia coli O145:H28 str. RM12581 [1248823]
Escherichia coli O145:H28 str. RM13514 [1248902]
Escherichia coli str. K-12 substr. W3110 [316407]
Escherichia coli ETEC H10407 [316401]
synthetic Escherichia coli C321.deltaA [1385755]
Escherichia coli VR50 [941323]
Escherichia coli BW25113 [679895]
Escherichia coli 1303 [745156]
Escherichia coli DH1 [536056]
Escherichia coli str. K-12 substr. DH10B [316385]
Escherichia coli O157:H7 str. EC4115 [444450]
Escherichia coli O157:H7 str. SS52 [1330457]
Escherichia coli O157:H16 [930406]
Escherichia coli [562]
Escherichia coli O55:H7 str. CB9615 [701177]
Escherichia coli UM146 [869729]
Escherichia coli O83:H1 str. NRG 857C [685038]
Escherichia coli UTI89 [364106]
Escherichia coli CFT073 [199310]
Escherichia coli S88 [585035]
Escherichia coli ATCC 25922 [1322345]
Escherichia coli IHE3034 [714962]
Escherichia coli APEC O1 [405955]
Escherichia coli O127:H6 str. E2348/69 [574521]
Escherichia coli 536 [362663]
Escherichia coli O7:K1 str. CE10 [1072459]
Escherichia coli BL21(DE3) [469008]
Escherichia coli BL21-Gold(DE3)pLysS AG [866768]
Escherichia coli B str. REL606 [413997]
Escherichia coli O25b:H4-ST131 [941322]

Escherichia coli JJ1886 [1355100]
Escherichia coli SE15 [431946]
Escherichia coli APEC IMT5155 [1329907]
Escherichia albertii KF1 [1440052]
Escherichia coli Nissle 1917 [316435]
Escherichia coli LF82 [591946]
Escherichia coli ABU 83972 [655817]
Escherichia coli str. clone D i2 [885276]
Escherichia coli str. clone D i14 [885275]
Escherichia coli SMS-3-5 [439855]
Escherichia coli IAI39 [585057]
Escherichia coli 042 [216592]
Escherichia coli K-12 [83333]
Escherichia coli ER2796 [1245474]
Escherichia coli UMNK88 [696406]
Escherichia coli B7A [340184]
Escherichia coli ATCC 8739 [481805]
Escherichia coli KLY [1435461]
Escherichia coli str. K-12 substr. MG1655 [511145]
Escherichia coli O157:H7 str. Sakai [386585]
Escherichia coli O157:H7 str. SS17 [1328859]
Escherichia coli O157:H7 str. EDL933 [155864]
Escherichia coli Xuzhou21 [741093]
Escherichia coli O157:H7 str. TW14359 [544404]
Escherichia coli O55:H7 str. RM12579 [1048689]
Escherichia coli O103:H2 str. 12009 [585395]
Escherichia coli O26:H11 str. 11368 [573235]
Escherichia coli O104:H4 str. 2009EL-2050 [1134782]
Escherichia coli O104:H4 str. 2009EL-2071 [1133853]
Escherichia coli HS [331112]
Escherichia coli O111:H- str. 11128 [585396]
Escherichia coli LY180 [1335916]
Escherichia coli ECC-1470 [758831]
Shigella flexneri 2a str. 2457T [198215]
Shigella flexneri 2a str. 301 [198214]
Shigella flexneri Shi06HN006 [1282358]
Shigella flexneri 2003036 [1282357]
Shigella flexneri [623]
Shigella flexneri 2002017 [591020]
Shigella flexneri 5 str. 8401 [373384]
Escherichia fergusonii ATCC 35469 [585054]
Escherichia coli NA114 [1033813]
Shigella sonnei Ss046 [300269]
Shigella sonnei 53G [216599]
Shigella boydii CDC 3083-94 [344609]
Shigella dysenteriae Sd197 [300267]
Escherichia coli BW2952 [595496]
Escherichia coli str. K-12 substr. MC4100 [1403831]
Escherichia coli P12b [910348]
Escherichia coli str. K-12 substr. MDS42 [1110693]
Klebsiella pneumoniae HK787 [1392500]
Klebsiella pneumoniae JM45 [1380908]
Klebsiella pneumoniae subsp. pneumoniae MGH 78578 [272620]
Klebsiella pneumoniae subsp. pneumoniae 1084 [1193292]
Klebsiella pneumoniae subsp. pneumoniae [72407]
Klebsiella pneumoniae CG43 [1244085]
Klebsiella pneumoniae subsp. pneumoniae PittNDM01 [1406314]
Klebsiella pneumoniae subsp. pneumoniae KPNIH10 [1094170]
Klebsiella pneumoniae [573]
Klebsiella pneumoniae subsp. pneumoniae KPNIH27 [1328324]
Klebsiella pneumoniae subsp. pneumoniae Kp13 [1123862]
Klebsiella pneumoniae 30684/NJST258_2 [1420013]
Klebsiella pneumoniae subsp. pneumoniae KPR0928 [1328325]
Klebsiella pneumoniae subsp. pneumoniae NTUH-K2044 [484021]
Klebsiella pneumoniae KCTC 2242 [1049565]
Klebsiella pneumoniae 30660/NJST258_1 [1420012]
Klebsiella pneumoniae subsp. pneumoniae KPNIH1 [1087440]
Klebsiella pneumoniae subsp. pneumoniae KPNIH24 [1225181]
Klebsiella pneumoniae subsp. pneumoniae 1158 [1392499]
Klebsiella pneumoniae subsp. pneumoniae HS11286 [1125630]
Klebsiella pneumoniae ATCC BAA-2146 [1263871]
Klebsiella variicola At-22 [640131]
Klebsiella pneumoniae 342 [507522]
Klebsiella variicola [244366]
Klebsiella pneumoniae subsp. pneumoniae KP5-1 [1199150]
Klebsiella aerogenes EA1509E [935296]
Klebsiella aerogenes KCTC 2190 [1028307]
Klebsiella michiganensis HKOPL1 [1308980]
Klebsiella michiganensis E718 [1191061]
Klebsiella oxytoca [571]
Klebsiella oxytoca KONIH1 [1333852]
Klebsiella michiganensis KCTC 1686 [1006551]
Raoultella ornithinolytica B6 [1286170]
Raoultella ornithinolytica [54291]
Enterobacter cloacae subsp. cloacae ATCC 13047 [716541]
Enterobacter cloacae subsp. dissolvens SDM [1104326]
Enterobacter cloacae subsp. cloacae ENHKU01 [1211025]
Enterobacter asburiae L1 [1421338]

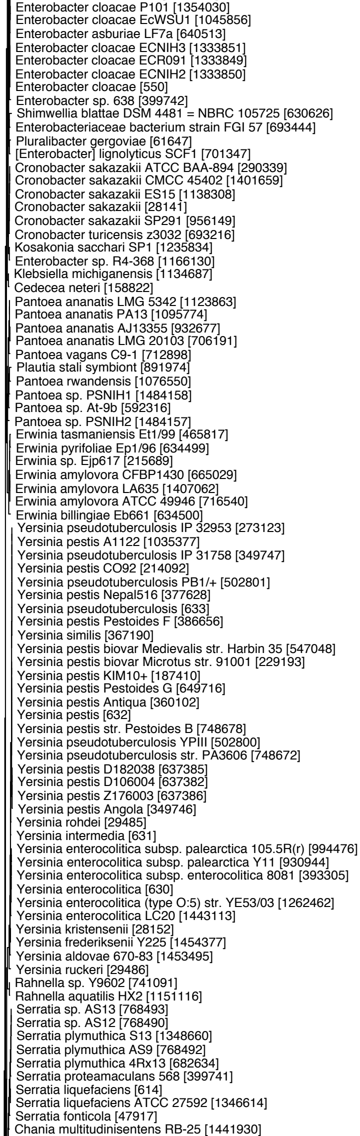
Enterobacter cloacae P101 [1354030]
Enterobacter cloacae EcWSU1 [1045856]
Enterobacter asburiae LF7a [640513]
Enterobacter cloacae ECNIH3 [1333851]
Enterobacter cloacae ECR091 [1333849]
Enterobacter cloacae ECNIH2 [1333850]
Enterobacter cloacae [550]
Enterobacter sp. 638 [399742]
Shimwellia blattae DSM 4481 = NBRC 105725 [630626]
Enterobacteriaceae bacterium strain FGI 57 [693444]
Pluralibacter gergoviae [61647]
[Enterobacter] lignolyticus SCF1 [701347]
Cronobacter sakazakii ATCC BAA-894 [290339]
Cronobacter sakazakii CMCC 45402 [1401659]
Cronobacter sakazakii ES15 [1138308]
Cronobacter sakazakii [28141]
Cronobacter sakazakii SP291 [956149]
Cronobacter turicensis z3032 [693216]
Kosakonia sacchari SP1 [1235834]
Enterobacter sp. R4-368 [1166130]
Klebsiella michiganensis [1134687]
Cedecea neteri [158822]
Pantoea ananatis LMG 5342 [1123863]
Pantoea ananatis PA13 [1095774]
Pantoea ananatis AJ13355 [932677]
Pantoea ananatis LMG 20103 [706191]
Pantoea vagans C9-1 [712898]
Plautia stali symbiont [891974]
Pantoea rwandensis [1076550]
Pantoea sp. PSNIH1 [1484158]
Pantoea sp. At-9b [592316]
Pantoea sp. PSNIH2 [1484157]
Erwinia tasmaniensis Et1/99 [465817]
Erwinia pyrifoliae Ep1/96 [634499]
Erwinia sp. Ejp617 [215689]
Erwinia amylovora CFBP1430 [665029]
Erwinia amylovora LA635 [1407062]
Erwinia amylovora ATCC 49946 [716540]
Erwinia billingiae Eb661 [634500]
Yersinia pseudotuberculosis IP 32953 [273123]
Yersinia pestis A1122 [1035377]
Yersinia pseudotuberculosis IP 31758 [349747]
Yersinia pestis CO92 [214092]
Yersinia pseudotuberculosis PB1/+ [502801]
Yersinia pestis Nepal516 [377628]
Yersinia pseudotuberculosis [633]
Yersinia pestis Pestoides F [386656]
Yersinia similis [367190]
Yersinia pestis biovar Medievalis str. Harbin 35 [547048]
Yersinia pestis biovar Microtus str. 91001 [229193]
Yersinia pestis KIM10+ [187410]
Yersinia pestis Pestoides G [649716]
Yersinia pestis Antiqua [360102]
Yersinia pestis [632]
Yersinia pestis str. Pestoides B [748678]
Yersinia pseudotuberculosis YPIII [502800]
Yersinia pseudotuberculosis str. PA3606 [748672]
Yersinia pestis D182038 [637385]
Yersinia pestis D106004 [637382]
Yersinia pestis Z176003 [637386]
Yersinia pestis Angola [349746]
Yersinia rohdei [29485]
Yersinia intermedia [631]
Yersinia enterocolitica subsp. palearctica 105.5R(r) [994476]
Yersinia enterocolitica subsp. palearctica Y11 [930944]
Yersinia enterocolitica subsp. enterocolitica 8081 [393305]
Yersinia enterocolitica [630]
Yersinia enterocolitica (type O:5) str. YE53/03 [1262462]
Yersinia enterocolitica LC20 [1443113]
Yersinia kristensenii [28152]
Yersinia frederiksenii Y225 [1454377]
Yersinia aldovae 670-83 [1453495]
Yersinia ruckeri [29486]
Rahnella sp. Y9602 [741091]
Rahnella aquatilis HX2 [1151116]
Serratia sp. AS13 [768493]
Serratia sp. AS12 [768490]
Serratia plymuthica S13 [1348660]
Serratia plymuthica AS9 [768492]
Serratia plymuthica 4Rx13 [682634]
Serratia proteamaculans 568 [399741]
Serratia liquefaciens [614]
Serratia liquefaciens ATCC 27592 [1346614]
Serratia fonticola [47917]
Chania multitudinisentens RB-25 [1441930]

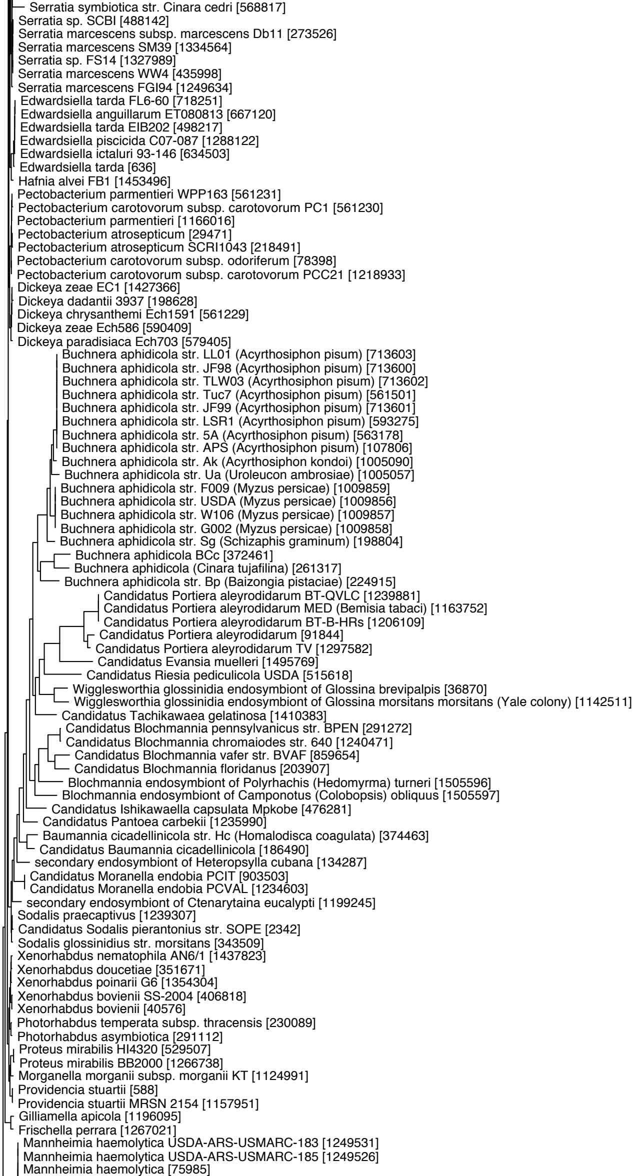
Serratia symbiotica str. Cinara cedri [568817]
Serratia sp. SCBI [488142]
Serratia marcescens subsp. marcescens Db11 [273526]
Serratia marcescens SM39 [1334564]
Serratia sp. FS14 [1327989]
Serratia marcescens WW4 [435998]
Serratia marcescens FGI94 [1249634]
Edwardsiella tarda FL6-60 [718251]
Edwardsiella anguillarum ET080813 [667120]
Edwardsiella tarda EIB202 [498217]
Edwardsiella piscicida C07-087 [1288122]
Edwardsiella ictaluri 93-146 [634503]
Edwardsiella tarda [636]
Hafnia alvei FB1 [1453496]
Pectobacterium parmentieri WPP163 [561231]
Pectobacterium carotovorum subsp. carotovorum PC1 [561230]
Pectobacterium parmentieri [1166016]
Pectobacterium atrosepticum [29471]
Pectobacterium atrosepticum SCRI1043 [218491]
Pectobacterium carotovorum subsp. odoriferum [78398]
Pectobacterium carotovorum subsp. carotovorum PCC21 [1218933]
Dickeya zeae EC1 [1427366]
Dickeya dadantii 3937 [198628]
Dickeya chrysanthemi Ech1591 [561229]
Dickeya zeae Ech586 [590409]
Dickeya paradisiaca Ech703 [579405]
Buchnera aphidicola str. LL01 (Acyrthosiphon pisum) [713603]
Buchnera aphidicola str. JF98 (Acyrthosiphon pisum) [713600]
Buchnera aphidicola str. TLW03 (Acyrthosiphon pisum) [713602]
Buchnera aphidicola str. Tuc7 (Acyrthosiphon pisum) [561501]
Buchnera aphidicola str. JF99 (Acyrthosiphon pisum) [713601]
Buchnera aphidicola str. LSR1 (Acyrthosiphon pisum) [593275]
Buchnera aphidicola str. 5A (Acyrthosiphon pisum) [563178]
Buchnera aphidicola str. APS (Acyrthosiphon pisum) [107806]
Buchnera aphidicola str. Ak (Acyrthosiphon kondoi) [1005090]
Buchnera aphidicola str. Ua (Uroleucon ambrosiae) [1005057]
Buchnera aphidicola str. F009 (Myzus persicae) [1009859]
Buchnera aphidicola str. USDA (Myzus persicae) [1009856]
Buchnera aphidicola str. W106 (Myzus persicae) [1009857]
Buchnera aphidicola str. G002 (Myzus persicae) [1009858]
Buchnera aphidicola str. Sg (Schizaphis graminum) [198804]
Buchnera aphidicola BCc [372461]
Buchnera aphidicola (Cinara tujafilina) [261317]
Buchnera aphidicola str. Bp (Baizongia pistaciae) [224915]
Candidatus Portiera aleyrodidarum BT-QVLC [1239881]
Candidatus Portiera aleyrodidarum MED (Bemisia tabaci) [1163752]
Candidatus Portiera aleyrodidarum BT-B-HRs [1206109]
Candidatus Portiera aleyrodidarum [91844]
Candidatus Portiera aleyrodidarum TV [1297582]
Candidatus Evansia muelleri [1495769]
Candidatus Riesia pediculicola USDA [515618]
Wigglesworthia glossinidia endosymbiont of Glossina brevipalpis [36870]
Wigglesworthia glossinidia endosymbiont of Glossina morsitans morsitans (Yale colony) [1142511]
Candidatus Tachikawaea gelatinosa [1410383]
Candidatus Blochmannia pennsylvanicus str. BPEN [291272]
Candidatus Blochmannia chromaiodes str. 640 [1240471]
Candidatus Blochmannia vafer str. BVAF [859654]
Candidatus Blochmannia floridanus [203907]
Blochmannia endosymbiont of Polyrhachis (Hedomyrma) turneri [1505596]
Blochmannia endosymbiont of Camponotus (Colobopsis) obliquus [1505597]
Candidatus Ishikawaella capsulata Mpkobe [476281]
Candidatus Pantoea carbekii [1235990]
Baumannia cicadellinicola str. Hc (Homalodisca coagulata) [374463]
Candidatus Baumannia cicadellinicola [186490]
secondary endosymbiont of Heteropsylla cubana [134287]
Candidatus Moranella endobia PCIT [903503]
Candidatus Moranella endobia PCVAL [1234603]
secondary endosymbiont of Ctenarytaina eucalypti [1199245]
Sodalis praecaptivus [1239307]
Candidatus Sodalis pierantonius str. SOPE [2342]
Sodalis glossinidius str. morsitans [343509]
Xenorhabdus nematophila AN6/1 [1437823]
Xenorhabdus doucetiae [351671]
Xenorhabdus poinarii G6 [1354304]
Xenorhabdus bovienii SS-2004 [406818]
Xenorhabdus bovienii [40576]
Photorhabdus temperata subsp. thracensis [230089]
Photorhabdus asymbiotica [291112]
Proteus mirabilis HI4320 [529507]
Proteus mirabilis BB2000 [1266738]
Morganella morganii subsp. morganii KT [1124991]
Providencia stuartii [588]
Providencia stuartii MRSN 2154 [1157951]
Gilliamella apicola [1196095]
Frischella perrara [1267021]
Mannheimia haemolytica USDA-ARS-USMARC-183 [1249531]
Mannheimia haemolytica USDA-ARS-USMARC-185 [1249526]
Mannheimia haemolytica [75985]

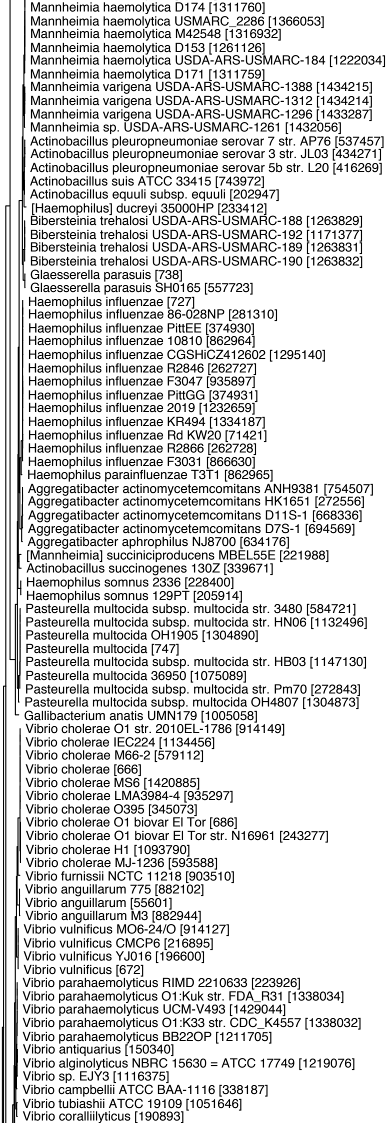
Mannheimia haemolytica D174 [1311760]
Mannheimia haemolytica USMARC_2286 [1366053]
Mannheimia haemolytica M42548 [1316932]
Mannheimia haemolytica D153 [1261126]
Mannheimia haemolytica USDA-ARS-USMARC-184 [1222034]
Mannheimia haemolytica D171 [1311759]
Mannheimia varigena USDA-ARS-USMARC-1388 [1434215]
Mannheimia varigena USDA-ARS-USMARC-1312 [1434214]
Mannheimia varigena USDA-ARS-USMARC-1296 [1433287]
Mannheimia sp. USDA-ARS-USMARC-1261 [1432056]
Actinobacillus pleuropneumoniae serovar 7 str. AP76 [537457]
Actinobacillus pleuropneumoniae serovar 3 str. JL03 [434271]
Actinobacillus pleuropneumoniae serovar 5b str. L20 [416269]
Actinobacillus suis ATCC 33415 [743972]
Actinobacillus equuli subsp. equuli [202947]
[Haemophilus] ducreyi 35000HP [233412]
Bibersteinia trehalosi USDA-ARS-USMARC-188 [1263829]
Bibersteinia trehalosi USDA-ARS-USMARC-192 [1171377]
Bibersteinia trehalosi USDA-ARS-USMARC-189 [1263831]
Bibersteinia trehalosi USDA-ARS-USMARC-190 [1263832]
Glaesserella parasuis [738]
Glaesserella parasuis SH0165 [557723]
Haemophilus influenzae [727]
Haemophilus influenzae 86-028NP [281310]
Haemophilus influenzae PittEE [374930]
Haemophilus influenzae 10810 [862964]
Haemophilus influenzae CGSHiCZ412602 [1295140]
Haemophilus influenzae R2846 [262727]
Haemophilus influenzae F3047 [935897]
Haemophilus influenzae PittGG [374931]
Haemophilus influenzae 2019 [1232659]
Haemophilus influenzae KR494 [1334187]
Haemophilus influenzae Rd KW20 [71421]
Haemophilus influenzae R2866 [262728]
Haemophilus influenzae F3031 [866630]
Haemophilus parainfluenzae T3T1 [862965]
Aggregatibacter actinomycetemcomitans ANH9381 [754507]
Aggregatibacter actinomycetemcomitans HK1651 [272556]
Aggregatibacter actinomycetemcomitans D11S-1 [668336]
Aggregatibacter actinomycetemcomitans D7S-1 [694569]
Aggregatibacter aphrophilus NJ8700 [634176]
[Mannheimia] succiniciproducens MBEL55E [221988]
Actinobacillus succinogenes 130Z [339671]
Haemophilus somnus 2336 [228400]
Haemophilus somnus 129PT [205914]
Pasteurella multocida subsp. multocida str. 3480 [584721]
Pasteurella multocida subsp. multocida str. HN06 [1132496]
Pasteurella multocida OH1905 [1304890]
Pasteurella multocida [747]
Pasteurella multocida subsp. multocida str. HB03 [1147130]
Pasteurella multocida 36950 [1075089]
Pasteurella multocida subsp. multocida str. Pm70 [272843]
Pasteurella multocida subsp. multocida OH4807 [1304873]
Gallibacterium anatis UMN179 [1005058]
Vibrio cholerae O1 str. 2010EL-1786 [914149]
Vibrio cholerae IEC224 [1134456]
Vibrio cholerae M66-2 [579112]
Vibrio cholerae [666]
Vibrio cholerae MS6 [1420885]
Vibrio cholerae LMA3984-4 [935297]
Vibrio cholerae O395 [345073]
Vibrio cholerae O1 biovar El Tor [686]
Vibrio cholerae O1 biovar El Tor str. N16961 [243277]
Vibrio cholerae H1 [1093790]
Vibrio cholerae MJ-1236 [593588]
Vibrio furnissii NCTC 11218 [903510]
Vibrio anguillarum 775 [882102]
Vibrio anguillarum [55601]
Vibrio anguillarum M3 [882944]
Vibrio vulnificus MO6-24/O [914127]
Vibrio vulnificus CMCP6 [216895]
Vibrio vulnificus YJ016 [196600]
Vibrio vulnificus [672]
Vibrio parahaemolyticus RIMD 2210633 [223926]
Vibrio parahaemolyticus O1:Kuk str. FDA_R31 [1338034]
Vibrio parahaemolyticus UCM-V493 [1429044]
Vibrio parahaemolyticus O1:K33 str. CDC_K4557 [1338032]
Vibrio parahaemolyticus BB22OP [1211705]
Vibrio antiquarius [150340]
Vibrio alginolyticus NBRC 15630 = ATCC 17749 [1219076]
Vibrio sp. EJY3 [1116375]
Vibrio campbellii ATCC BAA-1116 [338187]
Vibrio tubiashii ATCC 19109 [1051646]
Vibrio coralliilyticus [190893]

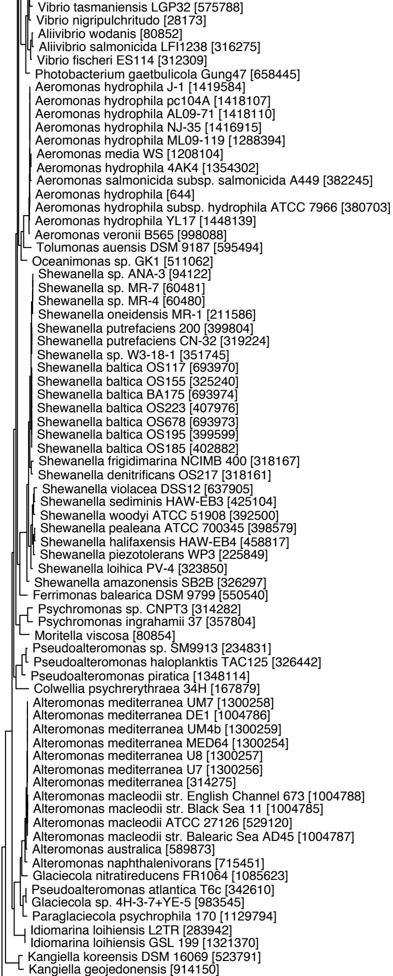
Vibrio tasmaniensis LGP32 [575788]
Vibrio nigripulchritudo [28173]
Aliivibrio wodanis [80852]
Aliivibrio salmonicida LFI1238 [316275]
Vibrio fischeri ES114 [312309]
Photobacterium gaetbulicola Gung47 [658445]
Aeromonas hydrophila J-1 [1419584]
Aeromonas hydrophila pc104A [1418107]
Aeromonas hydrophila AL09-71 [1418110]
Aeromonas hydrophila NJ-35 [1416915]
Aeromonas hydrophila ML09-119 [1288394]
Aeromonas media WS [1208104]
Aeromonas hydrophila 4AK4 [1354302]
Aeromonas salmonicida subsp. salmonicida A449 [382245]
Aeromonas hydrophila [644]
Aeromonas hydrophila subsp. hydrophila ATCC 7966 [380703]
Aeromonas hydrophila YL17 [1448139]
Aeromonas veronii B565 [998088]
Tolumonas auensis DSM 9187 [595494]
Oceanimonas sp. GK1 [511062]
Shewanella sp. ANA-3 [94122]
Shewanella sp. MR-7 [60481]
Shewanella sp. MR-4 [60480]
Shewanella oneidensis MR-1 [211586]
Shewanella putrefaciens 200 [399804]
Shewanella putrefaciens CN-32 [319224]
Shewanella sp. W3-18-1 [351745]
Shewanella baltica OS117 [693970]
Shewanella baltica OS155 [325240]
Shewanella baltica BA175 [693974]
Shewanella baltica OS223 [407976]
Shewanella baltica OS678 [693973]
Shewanella baltica OS195 [399599]
Shewanella baltica OS185 [402882]
Shewanella frigidimarina NCIMB 400 [318167]
Shewanella denitrificans OS217 [318161]
Shewanella violacea DSS12 [637905]
Shewanella sediminis HAW-EB3 [425104]
Shewanella woodyi ATCC 51908 [392500]
Shewanella pealeana ATCC 700345 [398579]
Shewanella halifaxensis HAW-EB4 [458817]
Shewanella piezotolerans WP3 [225849]
Shewanella loihica PV-4 [323850]
Shewanella amazonensis SB2B [326297]
Ferrimonas balearica DSM 9799 [550540]
Psychromonas sp. CNPT3 [314282]
Psychromonas ingrahamii 37 [357804]
Moritella viscosa [80854]
Pseudoalteromonas sp. SM9913 [234831]
Pseudoalteromonas haloplanktis TAC125 [326442]
Pseudoalteromonas piratica [1348114]
Colwellia psychrerythraea 34H [167879]
Alteromonas mediterranea UM7 [1300258]
Alteromonas mediterranea DE1 [1004786]
Alteromonas mediterranea UM4b [1300259]
Alteromonas mediterranea MED64 [1300254]
Alteromonas mediterranea U8 [1300257]
Alteromonas mediterranea U7 [1300256]
Alteromonas mediterranea [314275]
Alteromonas macleodii str. English Channel 673 [1004788]
Alteromonas macleodii str. Black Sea 11 [1004785]
Alteromonas macleodii ATCC 27126 [529120]
Alteromonas macleodii str. Balearic Sea AD45 [1004787]
Alteromonas australica [589873]
Alteromonas naphthalenivorans [715451]
Glaciecola nitratireducens FR1064 [1085623]
Pseudoalteromonas atlantica T6c [342610]
Glaciecola sp. 4H-3-7+YE-5 [983545]
Paraglaciecola psychrophila 170 [1129794]
Idiomarina loihiensis L2TR [283942]
Idiomarina loihiensis GSL 199 [1321370]
Kangiella koreensis DSM 16069 [523791]
Kangiella geojedonensis [914150]

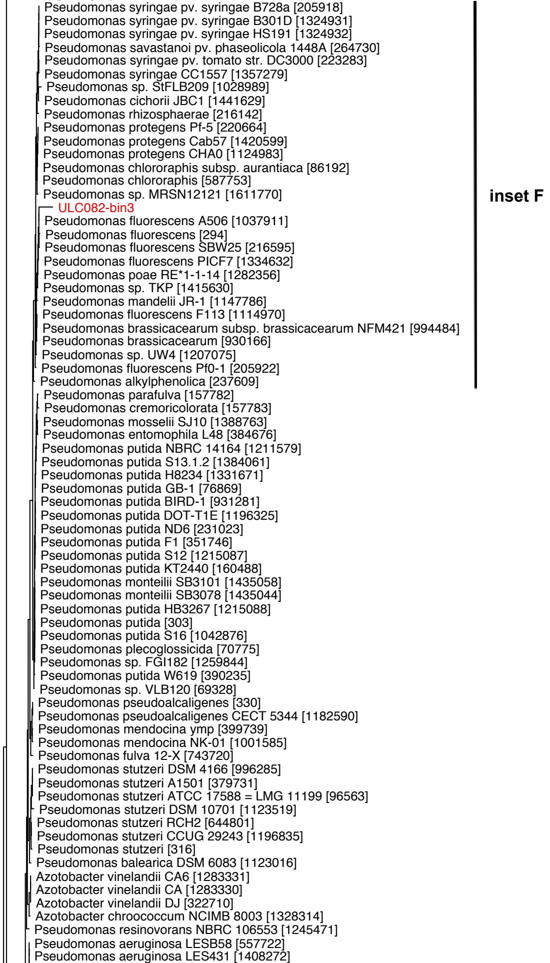
Pseudomonas syringae pv. syringae B728a [205918]
Pseudomonas syringae pv. syringae B301D [1324931]
Pseudomonas syringae pv. syringae HS191 [1324932]
Pseudomonas savastanoi pv. phaseolicola 1448A [264730]
Pseudomonas syringae pv. tomato str. DC3000 [223283]
Pseudomonas syringae CC1557 [1357279]
Pseudomonas sp. StFLB209 [1028989]
Pseudomonas cichorii JBC1 [1441629]
Pseudomonas rhizosphaerae [216142]
Pseudomonas protegens Pf-5 [220664]
Pseudomonas protegens Cab57 [1420599]
Pseudomonas protegens CHA0 [1124983]
Pseudomonas chlororaphis subsp. aurantiaca [86192]
Pseudomonas chlororaphis [587753]
Pseudomonas sp. MRSN12121 [1611770]
ULC082-bin3
Pseudomonas fluorescens A506 [1037911]
Pseudomonas fluorescens [294]
Pseudomonas fluorescens SBW25 [216595]
Pseudomonas fluorescens PICF7 [1334632]
Pseudomonas poae RE*1-1-14 [1282356]
Pseudomonas sp. TKP [1415630]
Pseudomonas mandelii JR-1 [1147786]
Pseudomonas fluorescens F113 [1114970]
Pseudomonas brassicacearum subsp. brassicacearum NFM421 [994484]
Pseudomonas brassicacearum [930166]
Pseudomonas sp. UW4 [1207075]
Pseudomonas fluorescens Pf0-1 [205922]
Pseudomonas alkylphenolica [237609]
Pseudomonas parafulva [157782]
Pseudomonas cremoricolorata [157783]
Pseudomonas mosselii SJ10 [1388763]
Pseudomonas entomophila L48 [384676]
Pseudomonas putida NBRC 14164 [1211579]
Pseudomonas putida S13.1.2 [1384061]
Pseudomonas putida H8234 [1331671]
Pseudomonas putida GB-1 [76869]
Pseudomonas putida BIRD-1 [931281]
Pseudomonas putida DOT-T1E [1196325]
Pseudomonas putida ND6 [231023]
Pseudomonas putida F1 [351746]
Pseudomonas putida S12 [1215087]
Pseudomonas putida KT2440 [160488]
Pseudomonas monteilii SB3101 [1435058]
Pseudomonas monteilii SB3078 [1435044]
Pseudomonas putida HB3267 [1215088]
Pseudomonas putida [303]
Pseudomonas putida S16 [1042876]
Pseudomonas plecoglossicida [70775]
Pseudomonas sp. FGI182 [1259844]
Pseudomonas putida W619 [390235]
Pseudomonas sp. VLB120 [69328]
Pseudomonas pseudoalcaligenes [330]
Pseudomonas pseudoalcaligenes CECT 5344 [1182590]
Pseudomonas mendocina ymp [399739]
Pseudomonas mendocina NK-01 [1001585]
Pseudomonas fulva 12-X [743720]
Pseudomonas stutzeri DSM 4166 [996285]
Pseudomonas stutzeri A1501 [379731]
Pseudomonas stutzeri ATCC 17588 = LMG 11199 [96563]
Pseudomonas stutzeri DSM 10701 [1123519]
Pseudomonas stutzeri RCH2 [644801]
Pseudomonas stutzeri CCUG 29243 [1196835]
Pseudomonas stutzeri [316]
Pseudomonas balearica DSM 6083 [1123016]
Azotobacter vinelandii CA6 [1283331]
Azotobacter vinelandii CA [1283330]
Azotobacter vinelandii DJ [322710]
Azotobacter chroococcum NCIMB 8003 [1328314]
Pseudomonas resinovorans NBRC 106553 [1245471]
Pseudomonas aeruginosa LESB58 [557722]
Pseudomonas aeruginosa LES431 [1408272]
inset F

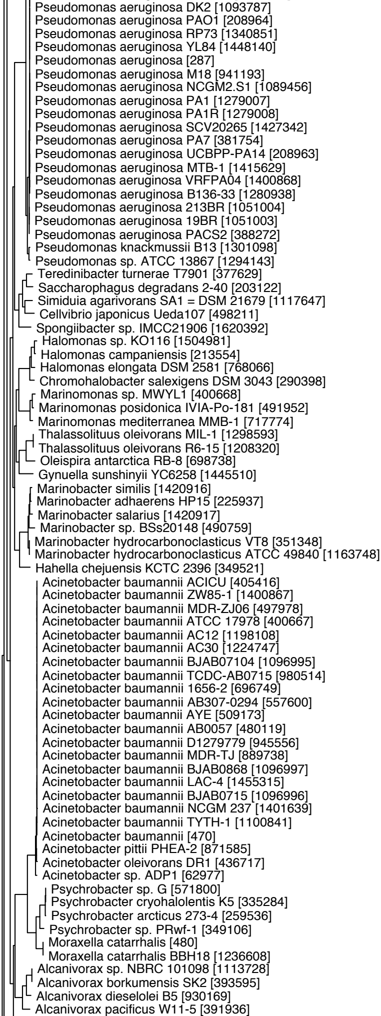
Pseudomonas aeruginosa DK2 [1093787]
Pseudomonas aeruginosa PAO1 [208964]
Pseudomonas aeruginosa RP73 [1340851]
Pseudomonas aeruginosa YL84 [1448140]
Pseudomonas aeruginosa [287]
Pseudomonas aeruginosa M18 [941193]
Pseudomonas aeruginosa NCGM2.S1 [1089456]
Pseudomonas aeruginosa PA1 [1279007]
Pseudomonas aeruginosa PA1R [1279008]
Pseudomonas aeruginosa SCV20265 [1427342]
Pseudomonas aeruginosa PA7 [381754]
Pseudomonas aeruginosa UCBPP-PA14 [208963]
Pseudomonas aeruginosa MTB-1 [1415629]
Pseudomonas aeruginosa VRFPA04 [1400868]
Pseudomonas aeruginosa B136-33 [1280938]
Pseudomonas aeruginosa 213BR [1051004]
Pseudomonas aeruginosa 19BR [1051003]
Pseudomonas aeruginosa PACS2 [388272]
Pseudomonas knackmussii B13 [1301098]
Pseudomonas sp. ATCC 13867 [1294143]
Teredinibacter turnerae T7901 [377629]
Saccharophagus degradans 2-40 [203122]
Simiduia agarivorans SA1 = DSM 21679 [1117647]
Cellvibrio japonicus Ueda107 [498211]
Spongiibacter sp. IMCC21906 [1620392]
Halomonas sp. KO116 [1504981]
Halomonas campaniensis [213554]
Halomonas elongata DSM 2581 [768066]
Chromohalobacter salexigens DSM 3043 [290398]
Marinomonas sp. MWYL1 [400668]
Marinomonas posidonica IVIA-Po-181 [491952]
Marinomonas mediterranea MMB-1 [717774]
Thalassolituus oleivorans MIL-1 [1298593]
Thalassolituus oleivorans R6-15 [1208320]
Oleispira antarctica RB-8 [698738]
Gynuella sunshinyii YC6258 [1445510]
Marinobacter similis [1420916]
Marinobacter adhaerens HP15 [225937]
Marinobacter salarius [1420917]
Marinobacter sp. BSs20148 [490759]
Marinobacter hydrocarbonoclasticus VT8 [351348]
Marinobacter hydrocarbonoclasticus ATCC 49840 [1163748]
Hahella chejuensis KCTC 2396 [349521]
Acinetobacter baumannii ACICU [405416]
Acinetobacter baumannii ZW85-1 [1400867]
Acinetobacter baumannii MDR-ZJ06 [497978]
Acinetobacter baumannii ATCC 17978 [400667]
Acinetobacter baumannii AC12 [1198108]
Acinetobacter baumannii AC30 [1224747]
Acinetobacter baumannii BJAB07104 [1096995]
Acinetobacter baumannii TCDC-AB0715 [980514]
Acinetobacter baumannii 1656-2 [696749]
Acinetobacter baumannii AB307-0294 [557600]
Acinetobacter baumannii AYE [509173]
Acinetobacter baumannii AB0057 [480119]
Acinetobacter baumannii D1279779 [945556]
Acinetobacter baumannii MDR-TJ [889738]
Acinetobacter baumannii BJAB0868 [1096997]
Acinetobacter baumannii LAC-4 [1455315]
Acinetobacter baumannii BJAB0715 [1096996]
Acinetobacter baumannii NCGM 237 [1401639]
Acinetobacter baumannii TYTH-1 [1100841]
Acinetobacter baumannii [470]
Acinetobacter pittii PHEA-2 [871585]
Acinetobacter oleivorans DR1 [436717]
Acinetobacter sp. ADP1 [62977]
Psychrobacter sp. G [571800]
Psychrobacter cryohalolentis K5 [335284]
Psychrobacter arcticus 273-4 [259536]
Psychrobacter sp. PRwf-1 [349106]
Moraxella catarrhalis [480]
Moraxella catarrhalis BBH18 [1236608]
Alcanivorax sp. NBRC 101098 [1113728]
Alcanivorax borkumensis SK2 [393595]
Alcanivorax dieselolei B5 [930169]
Alcanivorax pacificus W11-5 [391936]

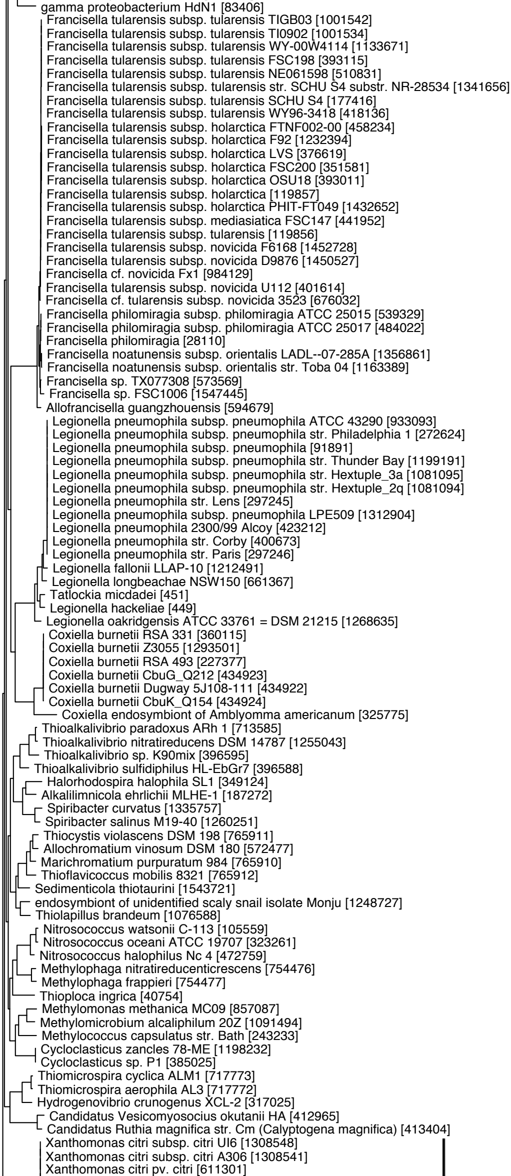

gamma proteobacterium HdN1 [83406]
Francisella tularensis subsp. tularensis TIGB03 [1001542]
Francisella tularensis subsp. tularensis TI0902 [1001534]
Francisella tularensis subsp. tularensis WY-00W4114 [1133671]
Francisella tularensis subsp. tularensis FSC198 [393115]
Francisella tularensis subsp. tularensis NE061598 [510831]
Francisella tularensis subsp. tularensis str. SCHU S4 substr. NR-28534 [1341656]
Francisella tularensis subsp. tularensis SCHU S4 [177416]
Francisella tularensis subsp. tularensis WY96-3418 [418136]
Francisella tularensis subsp. holarctica FTNF002-00 [458234]
Francisella tularensis subsp. holarctica F92 [1232394]
Francisella tularensis subsp. holarctica LVS [376619]
Francisella tularensis subsp. holarctica FSC200 [351581]
Francisella tularensis subsp. holarctica OSU18 [393011]
Francisella tularensis subsp. holarctica [119857]
Francisella tularensis subsp. holarctica PHIT-FT049 [1432652]
Francisella tularensis subsp. mediasiatica FSC147 [441952]
Francisella tularensis subsp. tularensis [119856]
Francisella tularensis subsp. novicida F6168 [1452728]
Francisella tularensis subsp. novicida D9876 [1450527]
Francisella cf. novicida Fx1 [984129]
Francisella tularensis subsp. novicida U112 [401614]
Francisella cf. tularensis subsp. novicida 3523 [676032]
Francisella philomiragia subsp. philomiragia ATCC 25015 [539329]
Francisella philomiragia subsp. philomiragia ATCC 25017 [484022]
Francisella philomiragia [28110]
Francisella noatunensis subsp. orientalis LADL--07-285A [1356861]
Francisella noatunensis subsp. orientalis str. Toba 04 [1163389]
Francisella sp. TX077308 [573569]
Francisella sp. FSC1006 [1547445]
Allofrancisella guangzhouensis [594679]
Legionella pneumophila subsp. pneumophila ATCC 43290 [933093]
Legionella pneumophila subsp. pneumophila str. Philadelphia 1 [272624]
Legionella pneumophila subsp. pneumophila [91891]
Legionella pneumophila subsp. pneumophila str. Thunder Bay [1199191]
Legionella pneumophila subsp. pneumophila str. Hextuple_3a [1081095]
Legionella pneumophila subsp. pneumophila str. Hextuple_2q [1081094]
Legionella pneumophila str. Lens [297245]
Legionella pneumophila subsp. pneumophila LPE509 [1312904]
Legionella pneumophila 2300/99 Alcoy [423212]
Legionella pneumophila str. Corby [400673]
Legionella pneumophila str. Paris [297246]
Legionella fallonii LLAP-10 [1212491]
Legionella longbeachae NSW150 [661367]
Tatlockia micdadei [451]
Legionella hackeliae [449]
Legionella oakridgensis ATCC 33761 = DSM 21215 [1268635]
Coxiella burnetii RSA 331 [360115]
Coxiella burnetii Z3055 [1293501]
Coxiella burnetii RSA 493 [227377]
Coxiella burnetii CbuG_Q212 [434923]
Coxiella burnetii Dugway 5J108-111 [434922]
Coxiella burnetii CbuK_Q154 [434924]
Coxiella endosymbiont of Amblyomma americanum [325775]
Thioalkalivibrio paradoxus ARh 1 [713585]
Thioalkalivibrio nitratireducens DSM 14787 [1255043]
Thioalkalivibrio sp. K90mix [396595]
Thioalkalivibrio sulfidiphilus HL-EbGr7 [396588]
Halorhodospira halophila SL1 [349124]
Alkalilimnicola ehrlichii MLHE-1 [187272]
Spiribacter curvatus [1335757]
Spiribacter salinus M19-40 [1260251]
Thiocystis violascens DSM 198 [765911]
Allochromatium vinosum DSM 180 [572477]
Marichromatium purpuratum 984 [765910]
Thioflavicoccus mobilis 8321 [765912]
Sedimenticola thiotaurini [1543721]
endosymbiont of unidentified scaly snail isolate Monju [1248727]
Thiolapillus brandeum [1076588]
Nitrosococcus watsonii C-113 [105559]
Nitrosococcus oceani ATCC 19707 [323261]
Nitrosococcus halophilus Nc 4 [472759]
Methylophaga nitratireducenticrescens [754476]
Methylophaga frappieri [754477]
Thioploca ingrica [40754]
Methylomonas methanica MC09 [857087]
Methylomicrobium alcaliphilum 20Z [1091494]
Methylococcus capsulatus str. Bath [243233]
Cycloclasticus zancles 78-ME [1198232]
Cycloclasticus sp. P1 [385025]
Thiomicrospira cyclica ALM1 [717773]
Thiomicrospira aerophila AL3 [717772]
Hydrogenovibrio crunogenus XCL-2 [317025]
Candidatus Vesicomyosocius okutanii HA [412965]
Candidatus Ruthia magnifica str. Cm (Calyptogena magnifica) [413404]
Xanthomonas citri subsp. citri UI6 [1308548]
Xanthomonas citri subsp. citri A306 [1308541]
Xanthomonas citri pv. citri [611301]

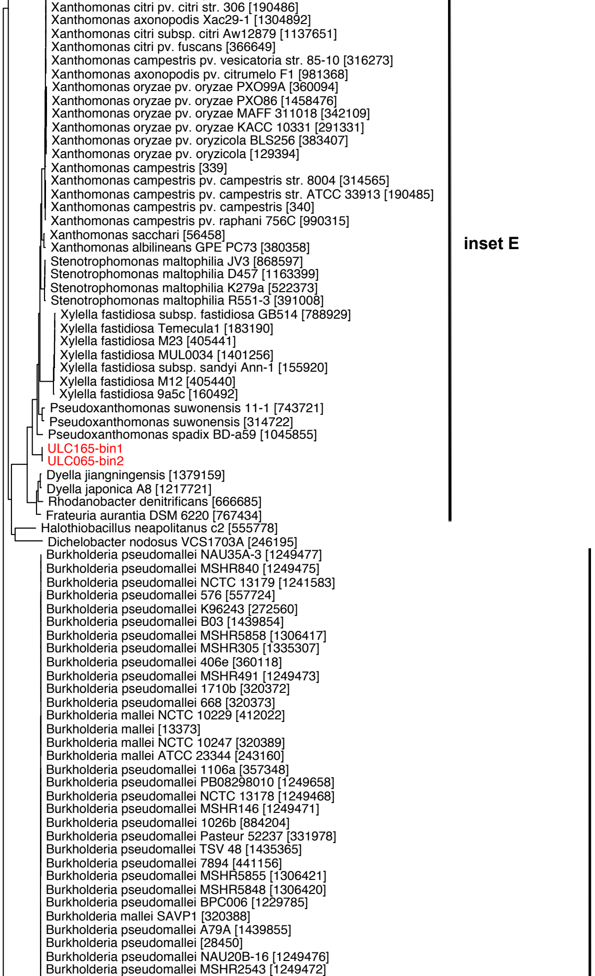
Xanthomonas citri pv. citri str. 306 [190486]
Xanthomonas axonopodis Xac29-1 [1304892]
Xanthomonas citri subsp. citri Aw12879 [1137651]
Xanthomonas citri pv. fuscans [366649]
Xanthomonas campestris pv. vesicatoria str. 85-10 [316273]
Xanthomonas axonopodis pv. citrumelo F1 [981368]
Xanthomonas oryzae pv. oryzae PXO99A [360094]
Xanthomonas oryzae pv. oryzae PXO86 [1458476]
Xanthomonas oryzae pv. oryzae MAFF 311018 [342109]
Xanthomonas oryzae pv. oryzae KACC 10331 [291331]
Xanthomonas oryzae pv. oryzicola BLS256 [383407]
Xanthomonas oryzae pv. oryzicola [129394]
Xanthomonas campestris [339]
Xanthomonas campestris pv. campestris str. 8004 [314565]
Xanthomonas campestris pv. campestris str. ATCC 33913 [190485]
Xanthomonas campestris pv. campestris [340]
Xanthomonas campestris pv. raphani 756C [990315]
Xanthomonas sacchari [56458]
Xanthomonas albilineans GPE PC73 [380358]
Stenotrophomonas maltophilia JV3 [868597]
Stenotrophomonas maltophilia D457 [1163399]
Stenotrophomonas maltophilia K279a [522373]
Stenotrophomonas maltophilia R551-3 [391008]
Xylella fastidiosa subsp. fastidiosa GB514 [788929]
Xylella fastidiosa Temecula1 [183190]
Xylella fastidiosa M23 [405441]
Xylella fastidiosa MUL0034 [1401256]
Xylella fastidiosa subsp. sandyi Ann-1 [155920]
Xylella fastidiosa M12 [405440]
Xylella fastidiosa 9a5c [160492]
Pseudoxanthomonas suwonensis 11-1 [743721]
Pseudoxanthomonas suwonensis [314722]
Pseudoxanthomonas spadix BD-a59 [1045855]
ULC165-bin1
ULC065-bin2
Dyella jiangningensis [1379159]
Dyella japonica A8 [1217721]
Rhodanobacter denitrificans [666685]
Frateuria aurantia DSM 6220 [767434]
Halothiobacillus neapolitanus c2 [555778]
Dichelobacter nodosus VCS1703A [246195]
inset E
Burkholderia pseudomallei NAU35A-3 [1249477]
Burkholderia pseudomallei MSHR840 [1249475]
Burkholderia pseudomallei NCTC 13179 [1241583]
Burkholderia pseudomallei 576 [557724]
Burkholderia pseudomallei K96243 [272560]
Burkholderia pseudomallei B03 [1439854]
Burkholderia pseudomallei MSHR5858 [1306417]
Burkholderia pseudomallei MSHR305 [1335307]
Burkholderia pseudomallei 406e [360118]
Burkholderia pseudomallei MSHR491 [1249473]
Burkholderia pseudomallei 1710b [320372]
Burkholderia pseudomallei 668 [320373]
Burkholderia mallei NCTC 10229 [412022]
Burkholderia mallei [13373]
Burkholderia mallei NCTC 10247 [320389]
Burkholderia mallei ATCC 23344 [243160]
Burkholderia pseudomallei 1106a [357348]
Burkholderia pseudomallei PB08298010 [1249658]
Burkholderia pseudomallei NCTC 13178 [1249468]
Burkholderia pseudomallei MSHR146 [1249471]
Burkholderia pseudomallei 1026b [884204]
Burkholderia pseudomallei Pasteur 52237 [331978]
Burkholderia pseudomallei TSV 48 [1435365]
Burkholderia pseudomallei 7894 [441156]
Burkholderia pseudomallei MSHR5855 [1306421]
Burkholderia pseudomallei MSHR5848 [1306420]
Burkholderia pseudomallei BPC006 [1229785]
Burkholderia mallei SAVP1 [320388]
Burkholderia pseudomallei A79A [1439855]
Burkholderia pseudomallei [28450]
Burkholderia pseudomallei NAU20B-16 [1249476]
Burkholderia pseudomallei MSHR2543 [1249472]

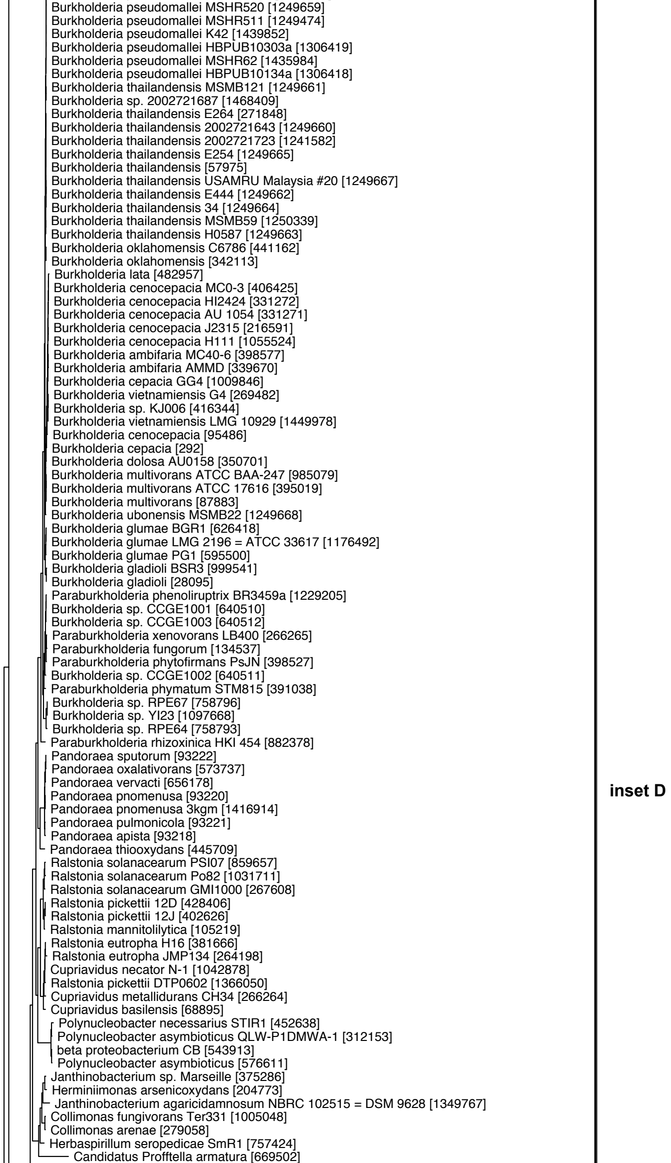
Burkholderia pseudomallei MSHR520 [1249659]
Burkholderia pseudomallei MSHR511 [1249474]
Burkholderia pseudomallei K42 [1439852]
Burkholderia pseudomallei HBPUB10303a [1306419]
Burkholderia pseudomallei MSHR62 [1435984]
Burkholderia pseudomallei HBPUB10134a [1306418]
Burkholderia thailandensis MSMB121 [1249661]
Burkholderia sp. 2002721687 [1468409]
Burkholderia thailandensis E264 [271848]
Burkholderia thailandensis 2002721643 [1249660]
Burkholderia thailandensis 2002721723 [1241582]
Burkholderia thailandensis E254 [1249665]
Burkholderia thailandensis [57975]
Burkholderia thailandensis USAMRU Malaysia #20 [1249667]
Burkholderia thailandensis E444 [1249662]
Burkholderia thailandensis 34 [1249664]
Burkholderia thailandensis MSMB59 [1250339]
Burkholderia thailandensis H0587 [1249663]
Burkholderia oklahomensis C6786 [441162]
Burkholderia oklahomensis [342113]
Burkholderia lata [482957]
Burkholderia cenocepacia MC0-3 [406425]
Burkholderia cenocepacia HI2424 [331272]
Burkholderia cenocepacia AU 1054 [331271]
Burkholderia cenocepacia J2315 [216591]
Burkholderia cenocepacia H111 [1055524]
Burkholderia ambifaria MC40-6 [398577]
Burkholderia ambifaria AMMD [339670]
Burkholderia cepacia GG4 [1009846]
Burkholderia vietnamiensis G4 [269482]
Burkholderia sp. KJ006 [416344]
Burkholderia vietnamiensis LMG 10929 [1449978]
Burkholderia cenocepacia [95486]
Burkholderia cepacia [292]
Burkholderia dolosa AU0158 [350701]
Burkholderia multivorans ATCC BAA-247 [985079]
Burkholderia multivorans ATCC 17616 [395019]
Burkholderia multivorans [87883]
Burkholderia ubonensis MSMB22 [1249668]
Burkholderia glumae BGR1 [626418]
Burkholderia glumae LMG 2196 = ATCC 33617 [1176492]
Burkholderia glumae PG1 [595500]
Burkholderia gladioli BSR3 [999541]
Burkholderia gladioli [28095]
Paraburkholderia phenoliruptrix BR3459a [1229205]
Burkholderia sp. CCGE1001 [640510]
Burkholderia sp. CCGE1003 [640512]
Paraburkholderia xenovorans LB400 [266265]
Paraburkholderia fungorum [134537]
Paraburkholderia phytofirmans PsJN [398527]
Burkholderia sp. CCGE1002 [640511]
Paraburkholderia phymatum STM815 [391038]
Burkholderia sp. RPE67 [758796]
Burkholderia sp. YI23 [1097668]
Burkholderia sp. RPE64 [758793]
Paraburkholderia rhizoxinica HKI 454 [882378]
Pandoraea sputorum [93222]
Pandoraea oxalativorans [573737]
Pandoraea vervacti [656178]
Pandoraea pnomenusa [93220]
Pandoraea pnomenusa 3kgm [1416914]
Pandoraea pulmonicola [93221]
Pandoraea apista [93218]
Pandoraea thiooxydans [445709]
Ralstonia solanacearum PSI07 [859657]
Ralstonia solanacearum Po82 [1031711]
Ralstonia solanacearum GMI1000 [267608]
Ralstonia pickettii 12D [428406]
Ralstonia pickettii 12J [402626]
Ralstonia mannitolilytica [105219]
Ralstonia eutropha H16 [381666]
Ralstonia eutropha JMP134 [264198]
Cupriavidus necator N-1 [1042878]
Ralstonia pickettii DTP0602 [1366050]
Cupriavidus metallidurans CH34 [266264]
Cupriavidus basilensis [68895]
Polynucleobacter necessarius STIR1 [452638]
Polynucleobacter asymbioticus QLW-P1DMWA-1 [312153]
beta proteobacterium CB [543913]
Polynucleobacter asymbioticus [576611]
Janthinobacterium sp. Marseille [375286]
Herminiimonas arsenicoxydans [204773]
Janthinobacterium agaricidamnosum NBRC 102515 = DSM 9628 [1349767]
Collimonas fungivorans Ter331 [1005048]
Collimonas arenae [279058]
Herbaspirillum seropedicae SmR1 [757424]
Candidatus Profftella armatura [669502]
inset D

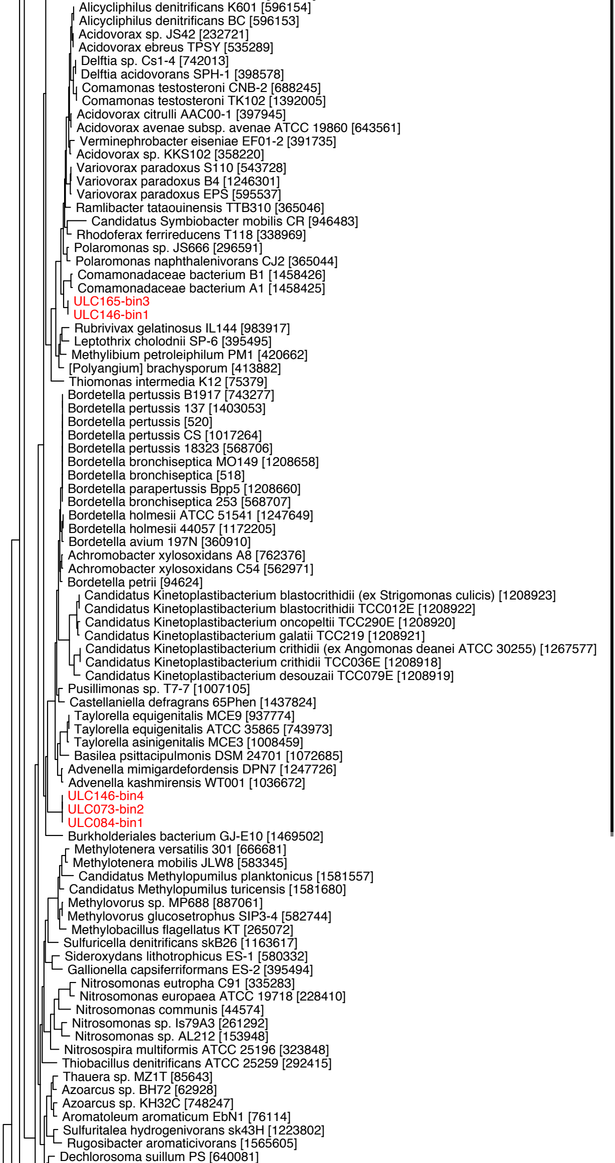

Alicycliphilus denitrificans K601 [596154]
Alicycliphilus denitrificans BC [596153]
Acidovorax sp. JS42 [232721]
Acidovorax ebreus TPSY [535289]
Delftia sp. Cs1-4 [742013]
Delftia acidovorans SPH-1 [398578]
Comamonas testosteroni CNB-2 [688245]
Comamonas testosteroni TK102 [1392005]
Acidovorax citrulli AAC00-1 [397945]
Acidovorax avenae subsp. avenae ATCC 19860 [643561]
Verminephrobacter eiseniae EF01-2 [391735]
Acidovorax sp. KKS102 [358220]
Variovorax paradoxus S110 [543728]
Variovorax paradoxus B4 [1246301]
Variovorax paradoxus EPS [595537]
Ramlibacter tataouinensis TTB310 [365046]
Candidatus Symbiobacter mobilis CR [946483]
Rhodoferax ferrireducens T118 [338969]
Polaromonas sp. JS666 [296591]
Polaromonas naphthalenivorans CJ2 [365044]
Comamonadaceae bacterium B1 [1458426]
Comamonadaceae bacterium A1 [1458425]
ULC165-bin3
ULC146-bin1
Rubrivivax gelatinosus IL144 [983917]
Leptothrix cholodnii SP-6 [395495]
Methylibium petroleiphilum PM1 [420662]
[Polyangium] brachysporum [413882]
Thiomonas intermedia K12 [75379]
Bordetella pertussis B1917 [743277]
Bordetella pertussis 137 [1403053]
Bordetella pertussis [520]
Bordetella pertussis CS [1017264]
Bordetella pertussis 18323 [568706]
Bordetella bronchiseptica MO149 [1208658]
Bordetella bronchiseptica [518]
Bordetella parapertussis Bpp5 [1208660]
Bordetella bronchiseptica 253 [568707]
Bordetella holmesii ATCC 51541 [1247649]
Bordetella holmesii 44057 [1172205]
Bordetella avium 197N [360910]
Achromobacter xylosoxidans A8 [762376]
Achromobacter xylosoxidans C54 [562971]
Bordetella petrii [94624]
Candidatus Kinetoplastibacterium blastocrithidii (ex Strigomonas culicis) [1208923]
Candidatus Kinetoplastibacterium blastocrithidii TCC012E [1208922]
Candidatus Kinetoplastibacterium oncopeltii TCC290E [1208920]
Candidatus Kinetoplastibacterium galatii TCC219 [1208921]
Candidatus Kinetoplastibacterium crithidii (ex Angomonas deanei ATCC 30255) [1267577]
Candidatus Kinetoplastibacterium crithidii TCC036E [1208918]
Candidatus Kinetoplastibacterium desouzaii TCC079E [1208919]
Pusillimonas sp. T7-7 [1007105]
Castellaniella defragrans 65Phen [1437824]
Taylorella equigenitalis MCE9 [937774]
Taylorella equigenitalis ATCC 35865 [743973]
Taylorella asinigenitalis MCE3 [1008459]
Basilea psittacipulmonis DSM 24701 [1072685]
Advenella mimigardefordensis DPN7 [1247726]
Advenella kashmirensis WT001 [1036672]
ULC146-bin4
ULC073-bin2
ULC084-bin1
Burkholderiales bacterium GJ-E10 [1469502]
Methylotenera versatilis 301 [666681]
Methylotenera mobilis JLW8 [583345]
Candidatus Methylopumilus planktonicus [1581557]
Candidatus Methylopumilus turicensis [1581680]
Methylovorus sp. MP688 [887061]
Methylovorus glucosetrophus SIP3-4 [582744]
Methylobacillus flagellatus KT [265072]
Sulfuricella denitrificans skB26 [1163617]
Sideroxydans lithotrophicus ES-1 [580332]
Gallionella capsiferriformans ES-2 [395494]
Nitrosomonas eutropha C91 [335283]
Nitrosomonas europaea ATCC 19718 [228410]
Nitrosomonas communis [44574]
Nitrosomonas sp. Is79A3 [261292]
Nitrosomonas sp. AL212 [153948]
Nitrosospira multiformis ATCC 25196 [323848]
Thiobacillus denitrificans ATCC 25259 [292415]
Thauera sp. MZ1T [85643]
Azoarcus sp. BH72 [62928]
Azoarcus sp. KH32C [748247]
Aromatoleum aromaticum EbN1 [76114]
Sulfuritalea hydrogenivorans sk43H [1223802]
Rugosibacter aromaticivorans [1565605]
Dechlorosoma suillum PS [640081]

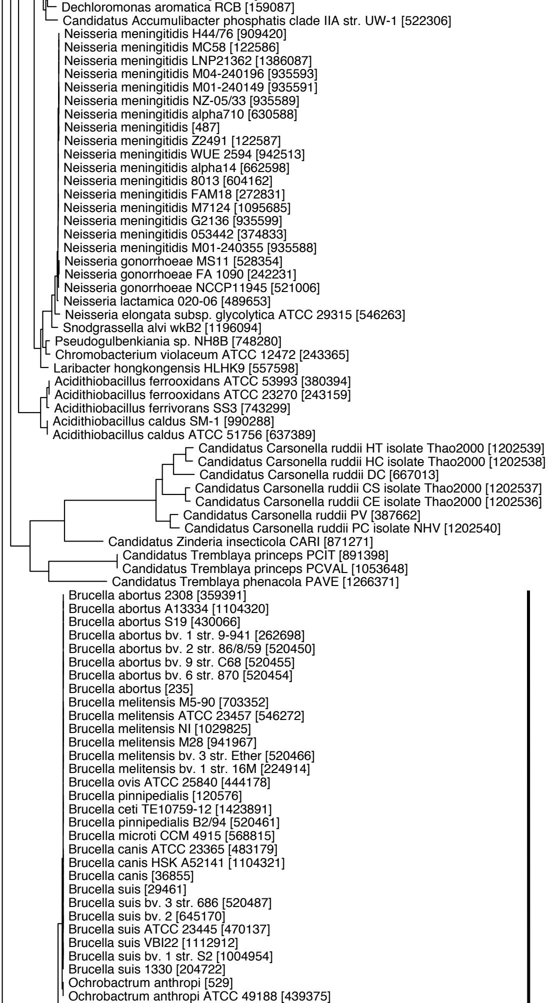

Dechloromonas aromatica RCB [159087]
Candidatus Accumulibacter phosphatis clade IIA str. UW-1 [522306]
Neisseria meningitidis H44/76 [909420]
Neisseria meningitidis MC58 [122586]
Neisseria meningitidis LNP21362 [1386087]
Neisseria meningitidis M04-240196 [935593]
Neisseria meningitidis M01-240149 [935591]
Neisseria meningitidis NZ-05/33 [935589]
Neisseria meningitidis alpha710 [630588]
Neisseria meningitidis [487]
Neisseria meningitidis Z2491 [122587]
Neisseria meningitidis WUE 2594 [942513]
Neisseria meningitidis alpha14 [662598]
Neisseria meningitidis 8013 [604162]
Neisseria meningitidis FAM18 [272831]
Neisseria meningitidis M7124 [1095685]
Neisseria meningitidis G2136 [935599]
Neisseria meningitidis 053442 [374833]
Neisseria meningitidis M01-240355 [935588]
Neisseria gonorrhoeae MS11 [528354]
Neisseria gonorrhoeae FA 1090 [242231]
Neisseria gonorrhoeae NCCP11945 [521006]
Neisseria lactamica 020-06 [489653]
Neisseria elongata subsp. glycolytica ATCC 29315 [546263]
Snodgrassella alvi wkB2 [1196094]
Pseudogulbenkiania sp. NH8B [748280]
Chromobacterium violaceum ATCC 12472 [243365]
Laribacter hongkongensis HLHK9 [557598]
Acidithiobacillus ferrooxidans ATCC 53993 [380394]
Acidithiobacillus ferrooxidans ATCC 23270 [243159]
Acidithiobacillus ferrivorans SS3 [743299]
Acidithiobacillus caldus SM-1 [990288]
Acidithiobacillus caldus ATCC 51756 [637389]
Candidatus Carsonella ruddii HT isolate Thao2000 [1202539]
Candidatus Carsonella ruddii HC isolate Thao2000 [1202538]
Candidatus Carsonella ruddii DC [667013]
Candidatus Carsonella ruddii CS isolate Thao2000 [1202537]
Candidatus Carsonella ruddii CE isolate Thao2000 [1202536]
Candidatus Carsonella ruddii PV [387662]
Candidatus Carsonella ruddii PC isolate NHV [1202540]
Candidatus Zinderia insecticola CARI [871271]
Candidatus Tremblaya princeps PCIT [891398]
Candidatus Tremblaya princeps PCVAL [1053648]
Candidatus Tremblaya phenacola PAVE [1266371]
Brucella abortus 2308 [359391]
Brucella abortus A13334 [1104320]
Brucella abortus S19 [430066]
Brucella abortus bv. 1 str. 9-941 [262698]
Brucella abortus bv. 2 str. 86/8/59 [520450]
Brucella abortus bv. 9 str. C68 [520455]
Brucella abortus bv. 6 str. 870 [520454]
Brucella abortus [235]
Brucella melitensis M5-90 [703352]
Brucella melitensis ATCC 23457 [546272]
Brucella melitensis NI [1029825]
Brucella melitensis M28 [941967]
Brucella melitensis bv. 3 str. Ether [520466]
Brucella melitensis bv. 1 str. 16M [224914]
Brucella ovis ATCC 25840 [444178]
Brucella pinnipedialis [120576]
Brucella ceti TE10759-12 [1423891]
Brucella pinnipedialis B2/94 [520461]
Brucella microti CCM 4915 [568815]
Brucella canis ATCC 23365 [483179]
Brucella canis HSK A52141 [1104321]
Brucella canis [36855]
Brucella suis [29461]
Brucella suis bv. 3 str. 686 [520487]
Brucella suis bv. 2 [645170]
Brucella suis ATCC 23445 [470137]
Brucella suis VBI22 [1112912]
Brucella suis bv. 1 str. S2 [1004954]
Brucella suis 1330 [204722]
Ochrobactrum anthropi [529]
Ochrobactrum anthropi ATCC 49188 [439375]

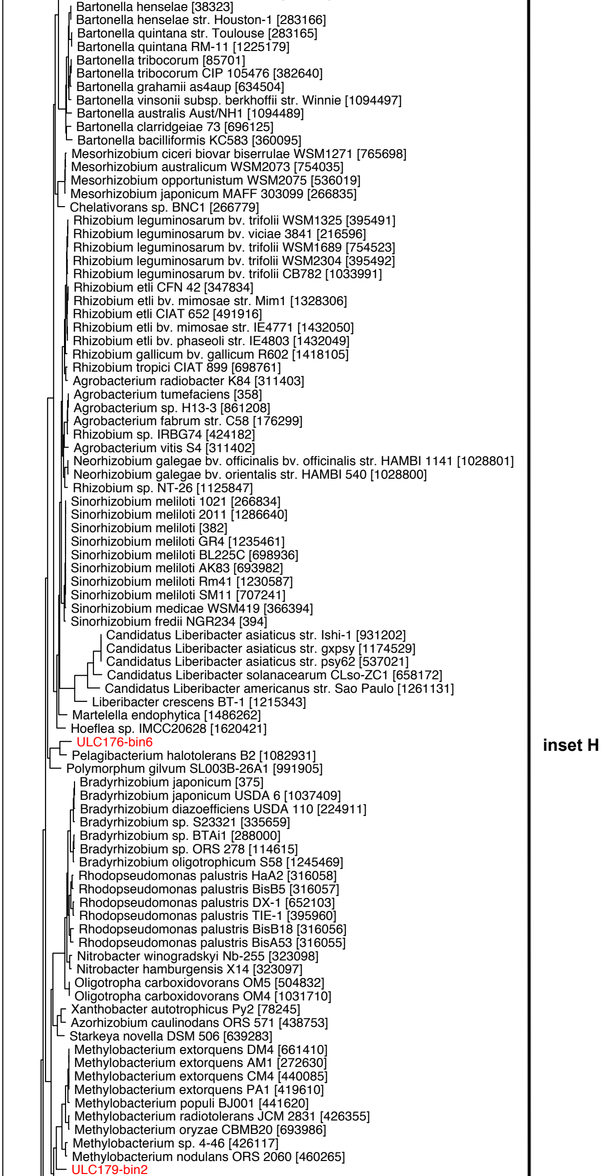

Bartonella henselae [38323]
Bartonella henselae str. Houston-1 [283166]
Bartonella quintana str. Toulouse [283165]
Bartonella quintana RM-11 [1225179]
Bartonella tribocorum [85701]
Bartonella tribocorum CIP 105476 [382640]
Bartonella grahamii as4aup [634504]
Bartonella vinsonii subsp. berkhoffii str. Winnie [1094497]
Bartonella australis Aust/NH1 [1094489]
Bartonella clarridgeiae 73 [696125]
Bartonella bacilliformis KC583 [360095]
Mesorhizobium ciceri biovar biserrulae WSM1271 [765698]
Mesorhizobium australicum WSM2073 [754035]
Mesorhizobium opportunistum WSM2075 [536019]
Mesorhizobium japonicum MAFF 303099 [266835]
Chelativorans sp. BNC1 [266779]
Rhizobium leguminosarum bv. trifolii WSM1325 [395491]
Rhizobium leguminosarum bv. viciae 3841 [216596]
Rhizobium leguminosarum bv. trifolii WSM1689 [754523]
Rhizobium leguminosarum bv. trifolii WSM2304 [395492]
Rhizobium leguminosarum bv. trifolii CB782 [1033991]
Rhizobium etli CFN 42 [347834]
Rhizobium etli bv. mimosae str. Mim1 [1328306]
Rhizobium etli CIAT 652 [491916]
Rhizobium etli bv. mimosae str. IE4771 [1432050]
Rhizobium etli bv. phaseoli str. IE4803 [1432049]
Rhizobium gallicum bv. gallicum R602 [1418105]
Rhizobium tropici CIAT 899 [698761]
Agrobacterium radiobacter K84 [311403]
Agrobacterium tumefaciens [358]
Agrobacterium sp. H13-3 [861208]
Agrobacterium fabrum str. C58 [176299]
Rhizobium sp. IRBG74 [424182]
Agrobacterium vitis S4 [311402]
Neorhizobium galegae bv. officinalis bv. officinalis str. HAMBI 1141 [1028801]
Neorhizobium galegae bv. orientalis str. HAMBI 540 [1028800]
Rhizobium sp. NT-26 [1125847]
Sinorhizobium meliloti 1021 [266834]
Sinorhizobium meliloti 2011 [1286640]
Sinorhizobium meliloti [382]
Sinorhizobium meliloti GR4 [1235461]
Sinorhizobium meliloti BL225C [698936]
Sinorhizobium meliloti AK83 [693982]
Sinorhizobium meliloti Rm41 [1230587]
Sinorhizobium meliloti SM11 [707241]
Sinorhizobium medicae WSM419 [366394]
Sinorhizobium fredii NGR234 [394]
Candidatus Liberibacter asiaticus str. Ishi-1 [931202]
Candidatus Liberibacter asiaticus str. gxpsy [1174529]
Candidatus Liberibacter asiaticus str. psy62 [537021]
Candidatus Liberibacter solanacearum CLso-ZC1 [658172]
Candidatus Liberibacter americanus str. Sao Paulo [1261131]
Liberibacter crescens BT-1 [1215343]
Martelella endophytica [1486262]
Hoeflea sp. IMCC20628 [1620421]
ULC176-bin6
Pelagibacterium halotolerans B2 [1082931]
Polymorphum gilvum SL003B-26A1 [991905]
Bradyrhizobium japonicum [375]
Bradyrhizobium japonicum USDA 6 [1037409]
Bradyrhizobium diazoefficiens USDA 110 [224911]
Bradyrhizobium sp. S23321 [335659]
Bradyrhizobium sp. BTAi1 [288000]
Bradyrhizobium sp. ORS 278 [114615]
Bradyrhizobium oligotrophicum S58 [1245469]
Rhodopseudomonas palustris HaA2 [316058]
Rhodopseudomonas palustris BisB5 [316057]
Rhodopseudomonas palustris DX-1 [652103]
Rhodopseudomonas palustris TIE-1 [395960]
Rhodopseudomonas palustris BisB18 [316056]
Rhodopseudomonas palustris BisA53 [316055]
Nitrobacter winogradskyi Nb-255 [323098]
Nitrobacter hamburgensis X14 [323097]
Oligotropha carboxidovorans OM5 [504832]
Oligotropha carboxidovorans OM4 [1031710]
Xanthobacter autotrophicus Py2 [78245]
Azorhizobium caulinodans ORS 571 [438753]
Starkeya novella DSM 506 [639283]
Methylobacterium extorquens DM4 [661410]
Methylobacterium extorquens AM1 [272630]
Methylobacterium extorquens CM4 [440085]
Methylobacterium extorquens PA1 [419610]
Methylobacterium populi BJ001 [441620]
Methylobacterium radiotolerans JCM 2831 [426355]
Methylobacterium oryzae CBMB20 [693986]
Methylobacterium sp. 4-46 [426117]
Methylobacterium nodulans ORS 2060 [460265]
ULC179-bin2
inset H

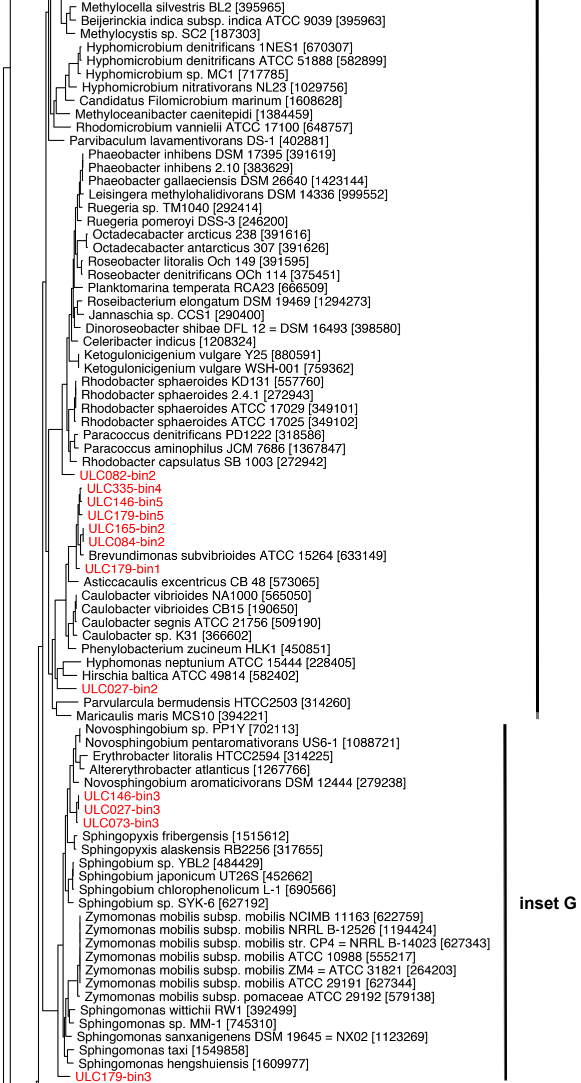
Methylocella silvestris BL2 [395965]
Beijerinckia indica subsp. indica ATCC 9039 [395963]
Methylocystis sp. SC2 [187303]
Hyphomicrobium denitrificans 1NES1 [670307]
Hyphomicrobium denitrificans ATCC 51888 [582899]
Hyphomicrobium sp. MC1 [717785]
Hyphomicrobium nitrativorans NL23 [1029756]
Candidatus Filomicrobium marinum [1608628]
Methyloceanibacter caenitepidi [1384459]
Rhodomicrobium vannielii ATCC 17100 [648757]
Parvibaculum lavamentivorans DS-1 [402881]
Phaeobacter inhibens DSM 17395 [391619]
Phaeobacter inhibens 2.10 [383629]
Phaeobacter gallaeciensis DSM 26640 [1423144]
Leisingera methylohalidivorans DSM 14336 [999552]
Ruegeria sp. TM1040 [292414]
Ruegeria pomeroyi DSS-3 [246200]
Octadecabacter arcticus 238 [391616]
Octadecabacter antarcticus 307 [391626]
Roseobacter litoralis Och 149 [391595]
Roseobacter denitrificans OCh 114 [375451]
Planktomarina temperata RCA23 [666509]
Roseibacterium elongatum DSM 19469 [1294273]
Jannaschia sp. CCS1 [290400]
Dinoroseobacter shibae DFL 12 = DSM 16493 [398580]
Celeribacter indicus [1208324]
Ketogulonicigenium vulgare Y25 [880591]
Ketogulonicigenium vulgare WSH-001 [759362]
Rhodobacter sphaeroides KD131 [557760]
Rhodobacter sphaeroides 2.4.1 [272943]
Rhodobacter sphaeroides ATCC 17029 [349101]
Rhodobacter sphaeroides ATCC 17025 [349102]
Paracoccus denitrificans PD1222 [318586]
Paracoccus aminophilus JCM 7686 [1367847]
Rhodobacter capsulatus SB 1003 [272942]
ULC082-bin2
ULC335-bin4
ULC146-bin5
ULC179-bin5
ULC165-bin2
ULC084-bin2
Brevundimonas subvibrioides ATCC 15264 [633149]
ULC179-bin1
Asticcacaulis excentricus CB 48 [573065]
Caulobacter vibrioides NA1000 [565050]
Caulobacter vibrioides CB15 [190650]
Caulobacter segnis ATCC 21756 [509190]
Caulobacter sp. K31 [366602]
Phenylobacterium zucineum HLK1 [450851]
Hyphomonas neptunium ATCC 15444 [228405]
Hirschia baltica ATCC 49814 [582402]
ULC027-bin2
Parvularcula bermudensis HTCC2503 [314260]
Maricaulis maris MCS10 [394221]
Novosphingobium sp. PP1Y [702113]
Novosphingobium pentaromativorans US6-1 [1088721]
Erythrobacter litoralis HTCC2594 [314225]
Altererythrobacter atlanticus [1267766]
Novosphingobium aromaticivorans DSM 12444 [279238]
ULC146-bin3
ULC027-bin3
ULC073-bin3
Sphingopyxis fribergensis [1515612]
Sphingopyxis alaskensis RB2256 [317655]
Sphingobium sp. YBL2 [484429]
Sphingobium japonicum UT26S [452662]
Sphingobium chlorophenolicum L-1 [690566]
Sphingobium sp. SYK-6 [627192]
Zymomonas mobilis subsp. mobilis NCIMB 11163 [622759]
Zymomonas mobilis subsp. mobilis NRRL B-12526 [1194424]
Zymomonas mobilis subsp. mobilis str. CP4 = NRRL B-14023 [627343]
Zymomonas mobilis subsp. mobilis ATCC 10988 [555217]
Zymomonas mobilis subsp. mobilis ZM4 = ATCC 31821 [264203]
Zymomonas mobilis subsp. mobilis ATCC 29191 [627344]
Zymomonas mobilis subsp. pomaceae ATCC 29192 [579138]
Sphingomonas wittichii RW1 [392499]
Sphingomonas sp. MM-1 [745310]
Sphingomonas sanxanigenens DSM 19645 = NX02 [1123269]
Sphingomonas taxi [1549858]
Sphingomonas hengshuiensis [1609977]
ULC179-bin3
inset G

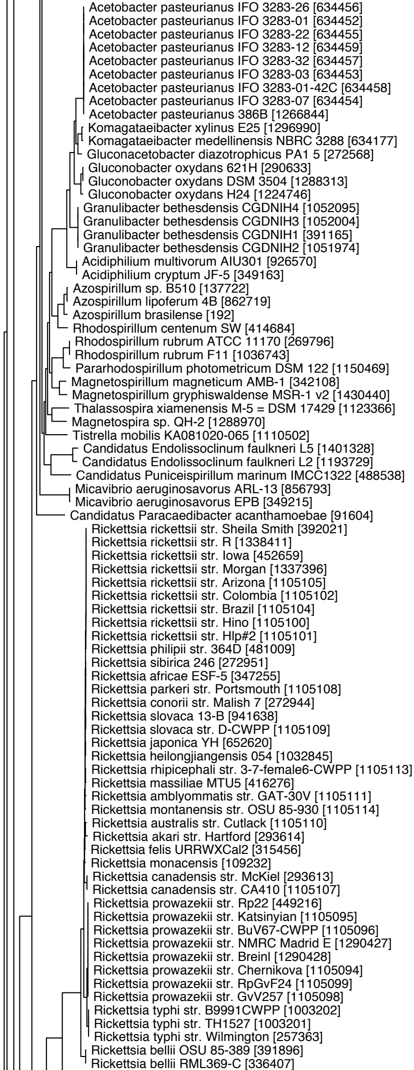
Acetobacter pasteurianus IFO 3283-26 [634456]
Acetobacter pasteurianus IFO 3283-01 [634452]
Acetobacter pasteurianus IFO 3283-22 [634455]
Acetobacter pasteurianus IFO 3283-12 [634459]
Acetobacter pasteurianus IFO 3283-32 [634457]
Acetobacter pasteurianus IFO 3283-03 [634453]
Acetobacter pasteurianus IFO 3283-01-42C [634458]
Acetobacter pasteurianus IFO 3283-07 [634454]
Acetobacter pasteurianus 386B [1266844]
Komagataeibacter xylinus E25 [1296990]
Komagataeibacter medellinensis NBRC 3288 [634177]
Gluconacetobacter diazotrophicus PA1 5 [272568]
Gluconobacter oxydans 621H [290633]
Gluconobacter oxydans DSM 3504 [1288313]
Gluconobacter oxydans H24 [1224746]
Granulibacter bethesdensis CGDNIH4 [1052095]
Granulibacter bethesdensis CGDNIH3 [1052004]
Granulibacter bethesdensis CGDNIH1 [391165]
Granulibacter bethesdensis CGDNIH2 [1051974]
Acidiphilium multivorum AIU301 [926570]
Acidiphilium cryptum JF-5 [349163]
Azospirillum sp. B510 [137722]
Azospirillum lipoferum 4B [862719]
Azospirillum brasilense [192]
Rhodospirillum centenum SW [414684]
Rhodospirillum rubrum ATCC 11170 [269796]
Rhodospirillum rubrum F11 [1036743]
Pararhodospirillum photometricum DSM 122 [1150469]
Magnetospirillum magneticum AMB-1 [342108]
Magnetospirillum gryphiswaldense MSR-1 v2 [1430440]
Thalassospira xiamenensis M-5 = DSM 17429 [1123366]
Magnetospira sp. QH-2 [1288970]
Tistrella mobilis KA081020-065 [1110502]
Candidatus Endolissoclinum faulkneri L5 [1401328]
Candidatus Endolissoclinum faulkneri L2 [1193729]
Candidatus Puniceispirillum marinum IMCC1322 [488538]
Micavibrio aeruginosavorus ARL-13 [856793]
Micavibrio aeruginosavorus EPB [349215]
Candidatus Paracaedibacter acanthamoebae [91604]
Rickettsia rickettsii str. Sheila Smith [392021]
Rickettsia rickettsii str. R [1338411]
Rickettsia rickettsii str. Iowa [452659]
Rickettsia rickettsii str. Morgan [1337396]
Rickettsia rickettsii str. Arizona [1105105]
Rickettsia rickettsii str. Colombia [1105102]
Rickettsia rickettsii str. Brazil [1105104]
Rickettsia rickettsii str. Hino [1105100]
Rickettsia rickettsii str. Hlp#2 [1105101]
Rickettsia philipii str. 364D [481009]
Rickettsia sibirica 246 [272951]
Rickettsia africae ESF-5 [347255]
Rickettsia parkeri str. Portsmouth [1105108]
Rickettsia conorii str. Malish 7 [272944]
Rickettsia slovaca 13-B [941638]
Rickettsia slovaca str. D-CWPP [1105109]
Rickettsia japonica YH [652620]
Rickettsia heilongjiangensis 054 [1032845]
Rickettsia rhipicephali str. 3-7-female6-CWPP [1105113]
Rickettsia massiliae MTU5 [416276]
Rickettsia amblyommatis str. GAT-30V [1105111]
Rickettsia montanensis str. OSU 85-930 [1105114]
Rickettsia australis str. Cutlack [1105110]
Rickettsia akari str. Hartford [293614]
Rickettsia felis URRWXCal2 [315456]
Rickettsia monacensis [109232]
Rickettsia canadensis str. McKiel [293613]
Rickettsia canadensis str. CA410 [1105107]
Rickettsia prowazekii str. Rp22 [449216]
Rickettsia prowazekii str. Katsinyian [1105095]
Rickettsia prowazekii str. BuV67-CWPP [1105096]
Rickettsia prowazekii str. NMRC Madrid E [1290427]
Rickettsia prowazekii str. Breinl [1290428]
Rickettsia prowazekii str. Chernikova [1105094]
Rickettsia prowazekii str. RpGvF24 [1105099]
Rickettsia prowazekii str. GvV257 [1105098]
Rickettsia typhi str. B9991CWPP [1003202]
Rickettsia typhi str. TH1527 [1003201]
Rickettsia typhi str. Wilmington [257363]
Rickettsia bellii OSU 85-389 [391896]
Rickettsia bellii RML369-C [336407]

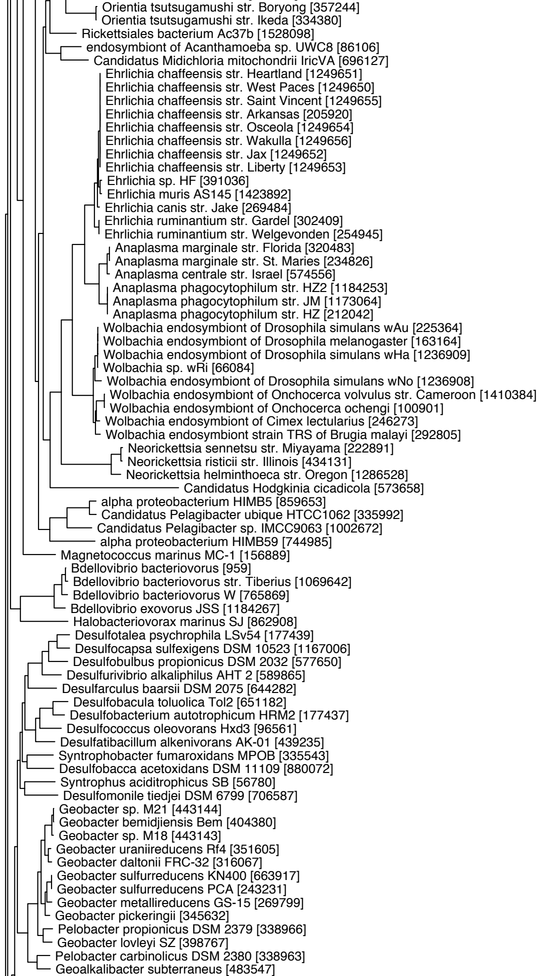
Orientia tsutsugamushi str. Boryong [357244]
Orientia tsutsugamushi str. Ikeda [334380]
Rickettsiales bacterium Ac37b [1528098]
endosymbiont of Acanthamoeba sp. UWC8 [86106]
Candidatus Midichloria mitochondrii IricVA [696127]
Ehrlichia chaffeensis str. Heartland [1249651]
Ehrlichia chaffeensis str. West Paces [1249650]
Ehrlichia chaffeensis str. Saint Vincent [1249655]
Ehrlichia chaffeensis str. Arkansas [205920]
Ehrlichia chaffeensis str. Osceola [1249654]
Ehrlichia chaffeensis str. Wakulla [1249656]
Ehrlichia chaffeensis str. Jax [1249652]
Ehrlichia chaffeensis str. Liberty [1249653]
Ehrlichia sp. HF [391036]
Ehrlichia muris AS145 [1423892]
Ehrlichia canis str. Jake [269484]
Ehrlichia ruminantium str. Gardel [302409]
Ehrlichia ruminantium str. Welgevonden [254945]
Anaplasma marginale str. Florida [320483]
Anaplasma marginale str. St. Maries [234826]
Anaplasma centrale str. Israel [574556]
Anaplasma phagocytophilum str. HZ2 [1184253]
Anaplasma phagocytophilum str. JM [1173064]
Anaplasma phagocytophilum str. HZ [212042]
Wolbachia endosymbiont of Drosophila simulans wAu [225364]
Wolbachia endosymbiont of Drosophila melanogaster [163164]
Wolbachia endosymbiont of Drosophila simulans wHa [1236909]
Wolbachia sp. wRi [66084]
Wolbachia endosymbiont of Drosophila simulans wNo [1236908]
Wolbachia endosymbiont of Onchocerca volvulus str. Cameroon [1410384]
Wolbachia endosymbiont of Onchocerca ochengi [100901]
Wolbachia endosymbiont of Cimex lectularius [246273]
Wolbachia endosymbiont strain TRS of Brugia malayi [292805]
Neorickettsia sennetsu str. Miyayama [222891]
Neorickettsia risticii str. Illinois [434131]
Neorickettsia helminthoeca str. Oregon [1286528]
Candidatus Hodgkinia cicadicola [573658]
alpha proteobacterium HIMB5 [859653]
Candidatus Pelagibacter ubique HTCC1062 [335992]
Candidatus Pelagibacter sp. IMCC9063 [1002672]
alpha proteobacterium HIMB59 [744985]
Magnetococcus marinus MC-1 [156889]
Bdellovibrio bacteriovorus [959]
Bdellovibrio bacteriovorus str. Tiberius [1069642]
Bdellovibrio bacteriovorus W [765869]
Bdellovibrio exovorus JSS [1184267]
Halobacteriovorax marinus SJ [862908]
Desulfotalea psychrophila LSv54 [177439]
Desulfocapsa sulfexigens DSM 10523 [1167006]
Desulfobulbus propionicus DSM 2032 [577650]
Desulfurivibrio alkaliphilus AHT 2 [589865]
Desulfarculus baarsii DSM 2075 [644282]
Desulfobacula toluolica Tol2 [651182]
Desulfobacterium autotrophicum HRM2 [177437]
Desulfococcus oleovorans Hxd3 [96561]
Desulfatibacillum alkenivorans AK-01 [439235]
Syntrophobacter fumaroxidans MPOB [335543]
Desulfobacca acetoxidans DSM 11109 [880072]
Syntrophus aciditrophicus SB [56780]
Desulfomonile tiedjei DSM 6799 [706587]
Geobacter sp. M21 [443144]
Geobacter bemidjiensis Bem [404380]
Geobacter sp. M18 [443143]
Geobacter uraniireducens Rf4 [351605]
Geobacter daltonii FRC-32 [316067]
Geobacter sulfurreducens KN400 [663917]
Geobacter sulfurreducens PCA [243231]
Geobacter metallireducens GS-15 [269799]
Geobacter pickeringii [345632]
Pelobacter propionicus DSM 2379 [338966]
Geobacter lovleyi SZ [398767]
Pelobacter carbinolicus DSM 2380 [338963]
Geoalkalibacter subterraneus [483547]

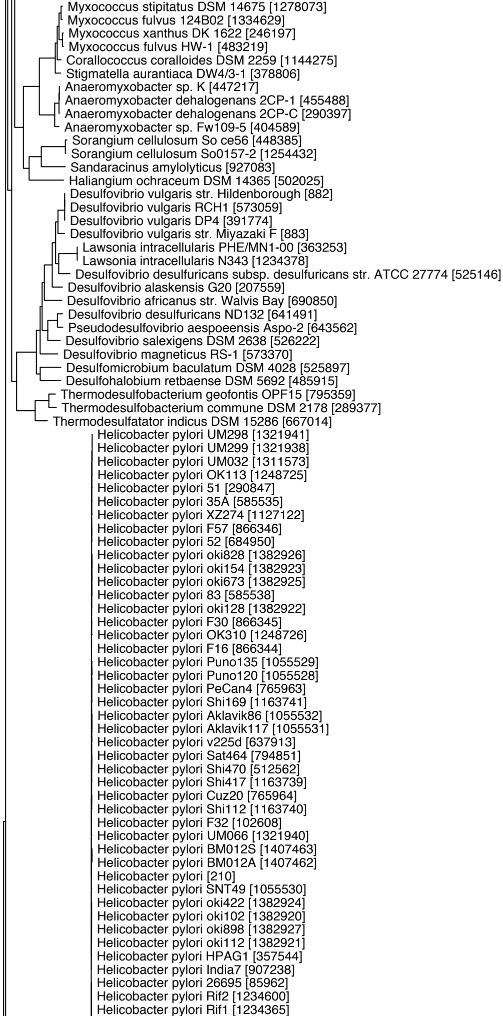
Myxococcus stipitatus DSM 14675 [1278073]
Myxococcus fulvus 124B02 [1334629]
Myxococcus xanthus DK 1622 [246197]
Myxococcus fulvus HW-1 [483219]
Corallococcus coralloides DSM 2259 [1144275]
Stigmatella aurantiaca DW4/3-1 [378806]
Anaeromyxobacter sp. K [447217]
Anaeromyxobacter dehalogenans 2CP-1 [455488]
Anaeromyxobacter dehalogenans 2CP-C [290397]
Anaeromyxobacter sp. Fw109-5 [404589]
Sorangium cellulosum So ce56 [448385]
Sorangium cellulosum So0157-2 [1254432]
Sandaracinus amylolyticus [927083]
Haliangium ochraceum DSM 14365 [502025]
Desulfovibrio vulgaris str. Hildenborough [882]
Desulfovibrio vulgaris RCH1 [573059]
Desulfovibrio vulgaris DP4 [391774]
Desulfovibrio vulgaris str. Miyazaki F [883]
Lawsonia intracellularis PHE/MN1-00 [363253]
Lawsonia intracellularis N343 [1234378]
Desulfovibrio desulfuricans subsp. desulfuricans str. ATCC 27774 [525146]
Desulfovibrio alaskensis G20 [207559]
Desulfovibrio africanus str. Walvis Bay [690850]
Desulfovibrio desulfuricans ND132 [641491]
Pseudodesulfovibrio aespoeensis Aspo-2 [643562]
Desulfovibrio salexigens DSM 2638 [526222]
Desulfovibrio magneticus RS-1 [573370]
Desulfomicrobium baculatum DSM 4028 [525897]
Desulfohalobium retbaense DSM 5692 [485915]
Thermodesulfobacterium geofontis OPF15 [795359]
Thermodesulfobacterium commune DSM 2178 [289377]
Thermodesulfatator indicus DSM 15286 [667014]
Helicobacter pylori UM298 [1321941]
Helicobacter pylori UM299 [1321938]
Helicobacter pylori UM032 [1311573]
Helicobacter pylori OK113 [1248725]
Helicobacter pylori 51 [290847]
Helicobacter pylori 35A [585535]
Helicobacter pylori XZ274 [1127122]
Helicobacter pylori F57 [866346]
Helicobacter pylori 52 [684950]
Helicobacter pylori oki828 [1382926]
Helicobacter pylori oki154 [1382923]
Helicobacter pylori oki673 [1382925]
Helicobacter pylori 83 [585538]
Helicobacter pylori oki128 [1382922]
Helicobacter pylori F30 [866345]
Helicobacter pylori OK310 [1248726]
Helicobacter pylori F16 [866344]
Helicobacter pylori Puno135 [1055529]
Helicobacter pylori Puno120 [1055528]
Helicobacter pylori PeCan4 [765963]
Helicobacter pylori Shi169 [1163741]
Helicobacter pylori Aklavik86 [1055532]
Helicobacter pylori Aklavik117 [1055531]
Helicobacter pylori v225d [637913]
Helicobacter pylori Sat464 [794851]
Helicobacter pylori Shi470 [512562]
Helicobacter pylori Shi417 [1163739]
Helicobacter pylori Cuz20 [765964]
Helicobacter pylori Shi112 [1163740]
Helicobacter pylori F32 [102608]
Helicobacter pylori UM066 [1321940]
Helicobacter pylori BM012S [1407463]
Helicobacter pylori BM012A [1407462]
Helicobacter pylori [210]
Helicobacter pylori SNT49 [1055530]
Helicobacter pylori oki422 [1382924]
Helicobacter pylori oki102 [1382920]
Helicobacter pylori oki898 [1382927]
Helicobacter pylori oki112 [1382921]
Helicobacter pylori HPAG1 [357544]
Helicobacter pylori India7 [907238]
Helicobacter pylori 26695 [85962]
Helicobacter pylori Rif2 [1234600]
Helicobacter pylori Rif1 [1234365]

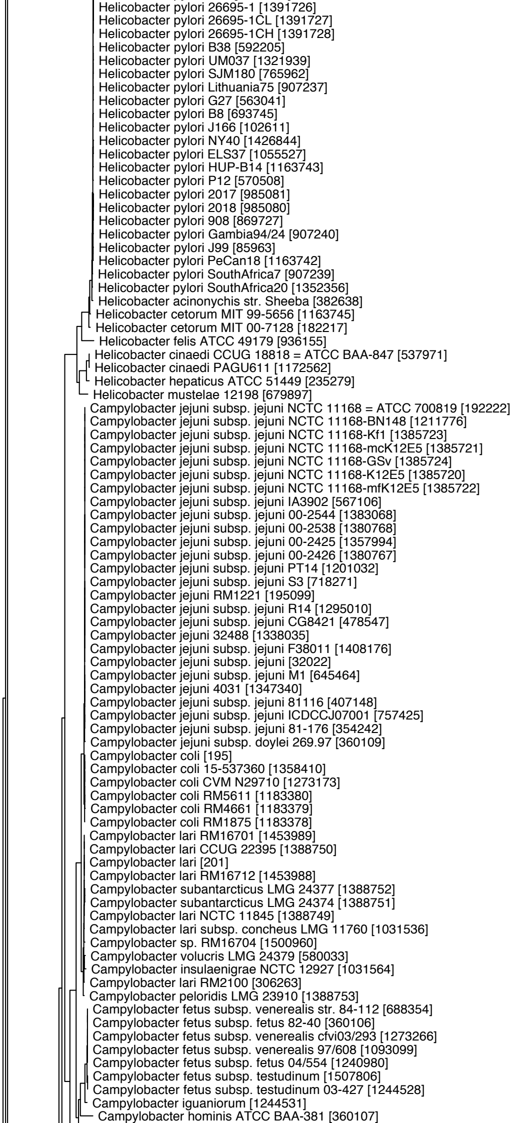

Helicobacter pylori 26695-1 [1391726]
Helicobacter pylori 26695-1CL [1391727]
Helicobacter pylori 26695-1CH [1391728]
Helicobacter pylori B38 [592205]
Helicobacter pylori UM037 [1321939]
Helicobacter pylori SJM180 [765962]
Helicobacter pylori Lithuania75 [907237]
Helicobacter pylori G27 [563041]
Helicobacter pylori B8 [693745]
Helicobacter pylori J166 [102611]
Helicobacter pylori NY40 [1426844]
Helicobacter pylori ELS37 [1055527]
Helicobacter pylori HUP-B14 [1163743]
Helicobacter pylori P12 [570508]
Helicobacter pylori 2017 [985081]
Helicobacter pylori 2018 [985080]
Helicobacter pylori 908 [869727]
Helicobacter pylori Gambia94/24 [907240]
Helicobacter pylori J99 [85963]
Helicobacter pylori PeCan18 [1163742]
Helicobacter pylori SouthAfrica7 [907239]
Helicobacter pylori SouthAfrica20 [1352356]
Helicobacter acinonychis str. Sheeba [382638]
Helicobacter cetorum MIT 99-5656 [1163745]
Helicobacter cetorum MIT 00-7128 [182217]
Helicobacter felis ATCC 49179 [936155]
Helicobacter cinaedi CCUG 18818 = ATCC BAA-847 [537971]
Helicobacter cinaedi PAGU611 [1172562]
Helicobacter hepaticus ATCC 51449 [235279]
Helicobacter mustelae 12198 [679897]
Campylobacter jejuni subsp. jejuni NCTC 11168 = ATCC 700819 [192222]
Campylobacter jejuni subsp. jejuni NCTC 11168-BN148 [1211776]
Campylobacter jejuni subsp. jejuni NCTC 11168-Kf1 [1385723]
Campylobacter jejuni subsp. jejuni NCTC 11168-mcK12E5 [1385721]
Campylobacter jejuni subsp. jejuni NCTC 11168-GSv [1385724]
Campylobacter jejuni subsp. jejuni NCTC 11168-K12E5 [1385720]
Campylobacter jejuni subsp. jejuni NCTC 11168-mfK12E5 [1385722]
Campylobacter jejuni subsp. jejuni IA3902 [567106]
Campylobacter jejuni subsp. jejuni 00-2544 [1383068]
Campylobacter jejuni subsp. jejuni 00-2538 [1380768]
Campylobacter jejuni subsp. jejuni 00-2425 [1357994]
Campylobacter jejuni subsp. jejuni 00-2426 [1380767]
Campylobacter jejuni subsp. jejuni PT14 [1201032]
Campylobacter jejuni subsp. jejuni S3 [718271]
Campylobacter jejuni RM1221 [195099]
Campylobacter jejuni subsp. jejuni R14 [1295010]
Campylobacter jejuni subsp. jejuni CG8421 [478547]
Campylobacter jejuni 32488 [1338035]
Campylobacter jejuni subsp. jejuni F38011 [1408176]
Campylobacter jejuni subsp. jejuni [32022]
Campylobacter jejuni subsp. jejuni M1 [645464]
Campylobacter jejuni 4031 [1347340]
Campylobacter jejuni subsp. jejuni 81116 [407148]
Campylobacter jejuni subsp. jejuni ICDCCJ07001 [757425]
Campylobacter jejuni subsp. jejuni 81-176 [354242]
Campylobacter jejuni subsp. doylei 269.97 [360109]
Campylobacter coli [195]
Campylobacter coli 15-537360 [1358410]
Campylobacter coli CVM N29710 [1273173]
Campylobacter coli RM5611 [1183380]
Campylobacter coli RM4661 [1183379]
Campylobacter coli RM1875 [1183378]
Campylobacter lari RM16701 [1453989]
Campylobacter lari CCUG 22395 [1388750]
Campylobacter lari [201]
Campylobacter lari RM16712 [1453988]
Campylobacter subantarcticus LMG 24377 [1388752]
Campylobacter subantarcticus LMG 24374 [1388751]
Campylobacter lari NCTC 11845 [1388749]
Campylobacter lari subsp. concheus LMG 11760 [1031536]
Campylobacter sp. RM16704 [1500960]
Campylobacter volucris LMG 24379 [580033]
Campylobacter insulaenigrae NCTC 12927 [1031564]
Campylobacter lari RM2100 [306263]
Campylobacter peloridis LMG 23910 [1388753]
Campylobacter fetus subsp. venerealis str. 84-112 [688354]
Campylobacter fetus subsp. fetus 82-40 [360106]
Campylobacter fetus subsp. venerealis cfvi03/293 [1273266]
Campylobacter fetus subsp. venerealis 97/608 [1093099]
Campylobacter fetus subsp. fetus 04/554 [1240980]
Campylobacter fetus subsp. testudinum [1507806]
Campylobacter fetus subsp. testudinum 03-427 [1244528]
Campylobacter iguaniorum [1244531]
Campylobacter hominis ATCC BAA-381 [360107]

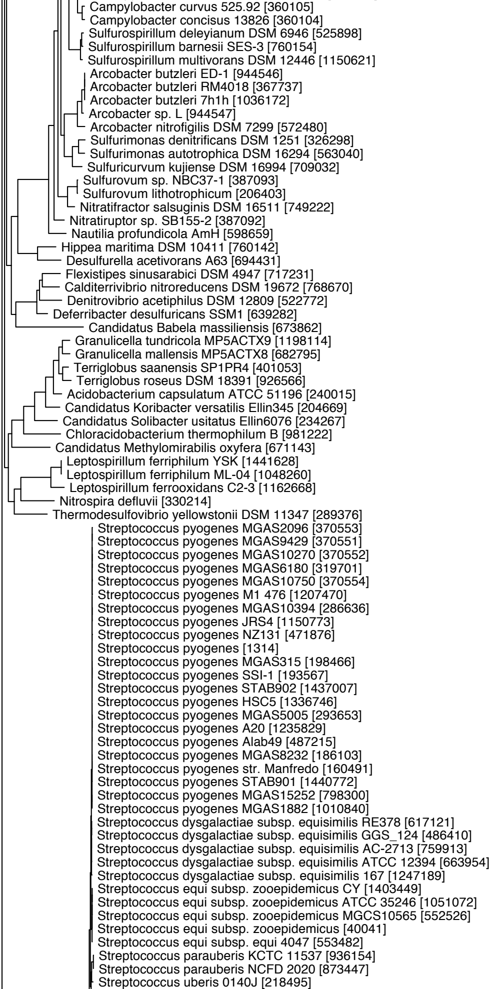
Campylobacter curvus 525.92 [360105]
Campylobacter concisus 13826 [360104]
Sulfurospirillum deleyianum DSM 6946 [525898]
Sulfurospirillum barnesii SES-3 [760154]
Sulfurospirillum multivorans DSM 12446 [1150621]
Arcobacter butzleri ED-1 [944546]
Arcobacter butzleri RM4018 [367737]
Arcobacter butzleri 7h1h [1036172]
Arcobacter sp. L [944547]
Arcobacter nitrofigilis DSM 7299 [572480]
Sulfurimonas denitrificans DSM 1251 [326298]
Sulfurimonas autotrophica DSM 16294 [563040]
Sulfuricurvum kujiense DSM 16994 [709032]
Sulfurovum sp. NBC37-1 [387093]
Sulfurovum lithotrophicum [206403]
Nitratifractor salsuginis DSM 16511 [749222]
Nitratiruptor sp. SB155-2 [387092]
Nautilia profundicola AmH [598659]
Hippea maritima DSM 10411 [760142]
Desulfurella acetivorans A63 [694431]
Flexistipes sinusarabici DSM 4947 [717231]
Calditerrivibrio nitroreducens DSM 19672 [768670]
Denitrovibrio acetiphilus DSM 12809 [522772]
Deferribacter desulfuricans SSM1 [639282]
Candidatus Babela massiliensis [673862]
Granulicella tundricola MP5ACTX9 [1198114]
Granulicella mallensis MP5ACTX8 [682795]
Terriglobus saanensis SP1PR4 [401053]
Terriglobus roseus DSM 18391 [926566]
Acidobacterium capsulatum ATCC 51196 [240015]
Candidatus Koribacter versatilis Ellin345 [204669]
Candidatus Solibacter usitatus Ellin6076 [234267]
Chloracidobacterium thermophilum B [981222]
Candidatus Methylomirabilis oxyfera [671143]
Leptospirillum ferriphilum YSK [1441628]
Leptospirillum ferriphilum ML-04 [1048260]
Leptospirillum ferrooxidans C2-3 [1162668]
Nitrospira defluvii [330214]
Thermodesulfovibrio yellowstonii DSM 11347 [289376]
Streptococcus pyogenes MGAS2096 [370553]
Streptococcus pyogenes MGAS9429 [370551]
Streptococcus pyogenes MGAS10270 [370552]
Streptococcus pyogenes MGAS6180 [319701]
Streptococcus pyogenes MGAS10750 [370554]
Streptococcus pyogenes M1 476 [1207470]
Streptococcus pyogenes MGAS10394 [286636]
Streptococcus pyogenes JRS4 [1150773]
Streptococcus pyogenes NZ131 [471876]
Streptococcus pyogenes [1314]
Streptococcus pyogenes MGAS315 [198466]
Streptococcus pyogenes SSI-1 [193567]
Streptococcus pyogenes STAB902 [1437007]
Streptococcus pyogenes HSC5 [1336746]
Streptococcus pyogenes MGAS5005 [293653]
Streptococcus pyogenes A20 [1235829]
Streptococcus pyogenes Alab49 [487215]
Streptococcus pyogenes MGAS8232 [186103]
Streptococcus pyogenes str. Manfredo [160491]
Streptococcus pyogenes STAB901 [1440772]
Streptococcus pyogenes MGAS15252 [798300]
Streptococcus pyogenes MGAS1882 [1010840]
Streptococcus dysgalactiae subsp. equisimilis RE378 [617121]
Streptococcus dysgalactiae subsp. equisimilis GGS_124 [486410]
Streptococcus dysgalactiae subsp. equisimilis AC-2713 [759913]
Streptococcus dysgalactiae subsp. equisimilis ATCC 12394 [663954]
Streptococcus dysgalactiae subsp. equisimilis 167 [1247189]
Streptococcus equi subsp. zooepidemicus CY [1403449]
Streptococcus equi subsp. zooepidemicus ATCC 35246 [1051072]
Streptococcus equi subsp. zooepidemicus MGCS10565 [552526]
Streptococcus equi subsp. zooepidemicus [40041]
Streptococcus equi subsp. equi 4047 [553482]
Streptococcus parauberis KCTC 11537 [936154]
Streptococcus parauberis NCFD 2020 [873447]
Streptococcus uberis 0140J [218495]

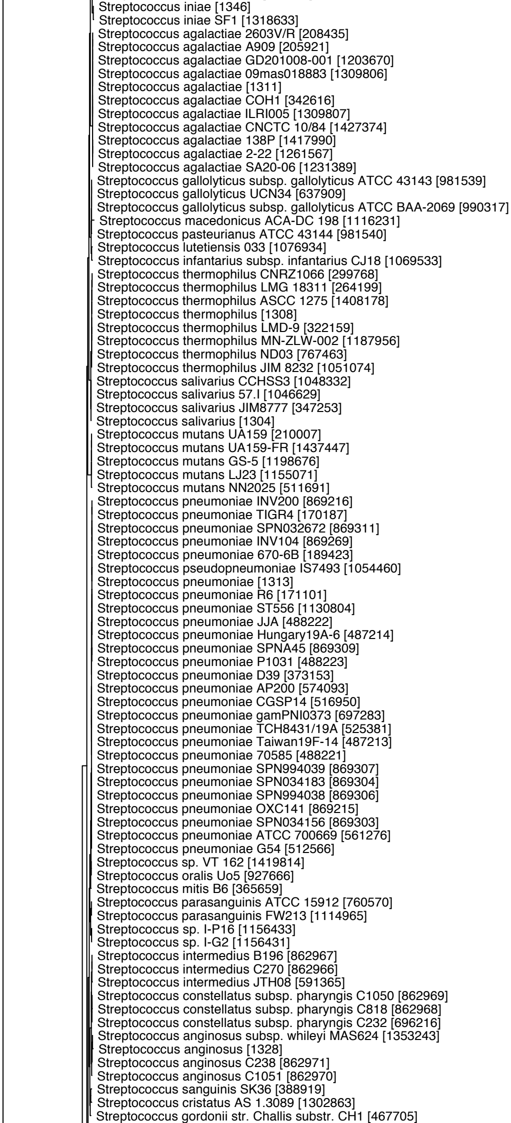
Streptococcus iniae [1346]
Streptococcus iniae SF1 [1318633]
Streptococcus agalactiae 2603V/R [208435]
Streptococcus agalactiae A909 [205921]
Streptococcus agalactiae GD201008-001 [1203670]
Streptococcus agalactiae 09mas018883 [1309806]
Streptococcus agalactiae [1311]
Streptococcus agalactiae COH1 [342616]
Streptococcus agalactiae ILRI005 [1309807]
Streptococcus agalactiae CNCTC 10/84 [1427374]
Streptococcus agalactiae 138P [1417990]
Streptococcus agalactiae 2-22 [1261567]
Streptococcus agalactiae SA20-06 [1231389]
Streptococcus gallolyticus subsp. gallolyticus ATCC 43143 [981539]
Streptococcus gallolyticus UCN34 [637909]
Streptococcus gallolyticus subsp. gallolyticus ATCC BAA-2069 [990317]
Streptococcus macedonicus ACA-DC 198 [1116231]
Streptococcus pasteurianus ATCC 43144 [981540]
Streptococcus lutetiensis 033 [1076934]
Streptococcus infantarius subsp. infantarius CJ18 [1069533]
Streptococcus thermophilus CNRZ1066 [299768]
Streptococcus thermophilus LMG 18311 [264199]
Streptococcus thermophilus ASCC 1275 [1408178]
Streptococcus thermophilus [1308]
Streptococcus thermophilus LMD-9 [322159]
Streptococcus thermophilus MN-ZLW-002 [1187956]
Streptococcus thermophilus ND03 [767463]
Streptococcus thermophilus JIM 8232 [1051074]
Streptococcus salivarius CCHSS3 [1048332]
Streptococcus salivarius 57.I [1046629]
Streptococcus salivarius JIM8777 [347253]
Streptococcus salivarius [1304]
Streptococcus mutans UA159 [210007]
Streptococcus mutans UA159-FR [1437447]
Streptococcus mutans GS-5 [1198676]
Streptococcus mutans LJ23 [1155071]
Streptococcus mutans NN2025 [511691]
Streptococcus pneumoniae INV200 [869216]
Streptococcus pneumoniae TIGR4 [170187]
Streptococcus pneumoniae SPN032672 [869311]
Streptococcus pneumoniae INV104 [869269]
Streptococcus pneumoniae 670-6B [189423]
Streptococcus pseudopneumoniae IS7493 [1054460]
Streptococcus pneumoniae [1313]
Streptococcus pneumoniae R6 [171101]
Streptococcus pneumoniae ST556 [1130804]
Streptococcus pneumoniae JJA [488222]
Streptococcus pneumoniae Hungary19A-6 [487214]
Streptococcus pneumoniae SPNA45 [869309]
Streptococcus pneumoniae P1031 [488223]
Streptococcus pneumoniae D39 [373153]
Streptococcus pneumoniae AP200 [574093]
Streptococcus pneumoniae CGSP14 [516950]
Streptococcus pneumoniae gamPNI0373 [697283]
Streptococcus pneumoniae TCH8431/19A [525381]
Streptococcus pneumoniae Taiwan19F-14 [487213]
Streptococcus pneumoniae 70585 [488221]
Streptococcus pneumoniae SPN994039 [869307]
Streptococcus pneumoniae SPN034183 [869304]
Streptococcus pneumoniae SPN994038 [869306]
Streptococcus pneumoniae OXC141 [869215]
Streptococcus pneumoniae SPN034156 [869303]
Streptococcus pneumoniae ATCC 700669 [561276]
Streptococcus pneumoniae G54 [512566]
Streptococcus sp. VT 162 [1419814]
Streptococcus oralis Uo5 [927666]
Streptococcus mitis B6 [365659]
Streptococcus parasanguinis ATCC 15912 [760570]
Streptococcus parasanguinis FW213 [1114965]
Streptococcus sp. I-P16 [1156433]
Streptococcus sp. I-G2 [1156431]
Streptococcus intermedius B196 [862967]
Streptococcus intermedius C270 [862966]
Streptococcus intermedius JTH08 [591365]
Streptococcus constellatus subsp. pharyngis C1050 [862969]
Streptococcus constellatus subsp. pharyngis C818 [862968]
Streptococcus constellatus subsp. pharyngis C232 [696216]
Streptococcus anginosus subsp. whileyi MAS624 [1353243]
Streptococcus anginosus [1328]
Streptococcus anginosus C238 [862971]
Streptococcus anginosus C1051 [862970]
Streptococcus sanguinis SK36 [388919]
Streptococcus cristatus AS 1.3089 [1302863]
Streptococcus gordonii str. Challis substr. CH1 [467705]

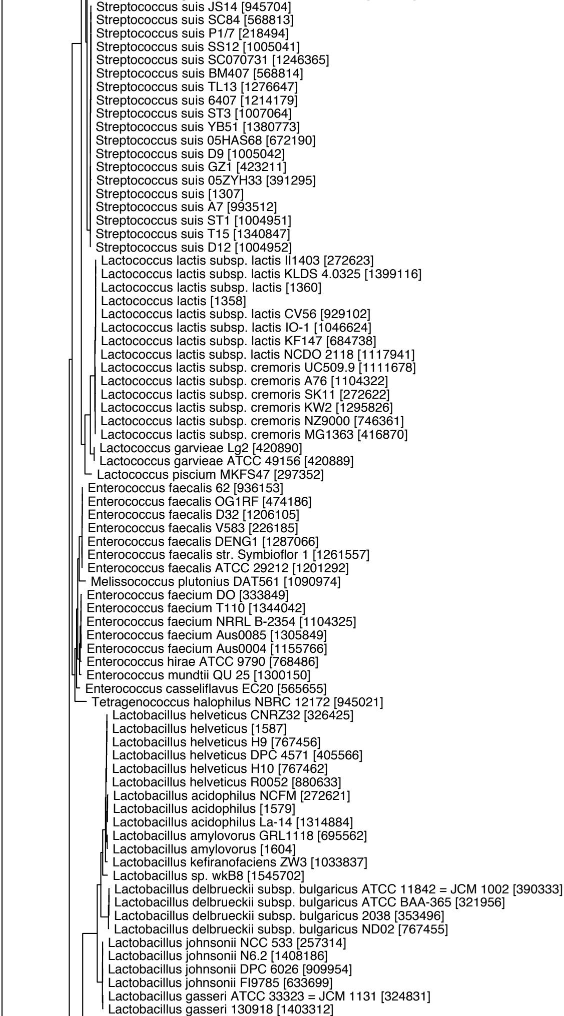

Streptococcus suis JS14 [945704]
Streptococcus suis SC84 [568813]
Streptococcus suis P1/7 [218494]
Streptococcus suis SS12 [1005041]
Streptococcus suis SC070731 [1246365]
Streptococcus suis BM407 [568814]
Streptococcus suis TL13 [1276647]
Streptococcus suis 6407 [1214179]
Streptococcus suis ST3 [1007064]
Streptococcus suis YB51 [1380773]
Streptococcus suis 05HAS68 [672190]
Streptococcus suis D9 [1005042]
Streptococcus suis GZ1 [423211]
Streptococcus suis 05ZYH33 [391295]
Streptococcus suis [1307]
Streptococcus suis A7 [993512]
Streptococcus suis ST1 [1004951]
Streptococcus suis T15 [1340847]
Streptococcus suis D12 [1004952]
Lactococcus lactis subsp. lactis Il1403 [272623]
Lactococcus lactis subsp. lactis KLDS 4.0325 [1399116]
Lactococcus lactis subsp. lactis [1360]
Lactococcus lactis [1358]
Lactococcus lactis subsp. lactis CV56 [929102]
Lactococcus lactis subsp. lactis IO-1 [1046624]
Lactococcus lactis subsp. lactis KF147 [684738]
Lactococcus lactis subsp. lactis NCDO 2118 [1117941]
Lactococcus lactis subsp. cremoris UC509.9 [1111678]
Lactococcus lactis subsp. cremoris A76 [1104322]
Lactococcus lactis subsp. cremoris SK11 [272622]
Lactococcus lactis subsp. cremoris KW2 [1295826]
Lactococcus lactis subsp. cremoris NZ9000 [746361]
Lactococcus lactis subsp. cremoris MG1363 [416870]
Lactococcus garvieae Lg2 [420890]
Lactococcus garvieae ATCC 49156 [420889]
Lactococcus piscium MKFS47 [297352]
Enterococcus faecalis 62 [936153]
Enterococcus faecalis OG1RF [474186]
Enterococcus faecalis D32 [1206105]
Enterococcus faecalis V583 [226185]
Enterococcus faecalis DENG1 [1287066]
Enterococcus faecalis str. Symbioflor 1 [1261557]
Enterococcus faecalis ATCC 29212 [1201292]
Melissococcus plutonius DAT561 [1090974]
Enterococcus faecium DO [333849]
Enterococcus faecium T110 [1344042]
Enterococcus faecium NRRL B-2354 [1104325]
Enterococcus faecium Aus0085 [1305849]
Enterococcus faecium Aus0004 [1155766]
Enterococcus hirae ATCC 9790 [768486]
Enterococcus mundtii QU 25 [1300150]
Enterococcus casseliflavus EC20 [565655]
Tetragenococcus halophilus NBRC 12172 [945021]
Lactobacillus helveticus CNRZ32 [326425]
Lactobacillus helveticus [1587]
Lactobacillus helveticus H9 [767456]
Lactobacillus helveticus DPC 4571 [405566]
Lactobacillus helveticus H10 [767462]
Lactobacillus helveticus R0052 [880633]
Lactobacillus acidophilus NCFM [272621]
Lactobacillus acidophilus [1579]
Lactobacillus acidophilus La-14 [1314884]
Lactobacillus amylovorus GRL1118 [695562]
Lactobacillus amylovorus [1604]
Lactobacillus kefiranofaciens ZW3 [1033837]
Lactobacillus sp. wkB8 [1545702]
Lactobacillus delbrueckii subsp. bulgaricus ATCC 11842 = JCM 1002 [390333]
Lactobacillus delbrueckii subsp. bulgaricus ATCC BAA-365 [321956]
Lactobacillus delbrueckii subsp. bulgaricus 2038 [353496]
Lactobacillus delbrueckii subsp. bulgaricus ND02 [767455]
Lactobacillus johnsonii NCC 533 [257314]
Lactobacillus johnsonii N6.2 [1408186]
Lactobacillus johnsonii DPC 6026 [909954]
Lactobacillus johnsonii FI9785 [633699]
Lactobacillus gasseri ATCC 33323 = JCM 1131 [324831]
Lactobacillus gasseri 130918 [1403312]

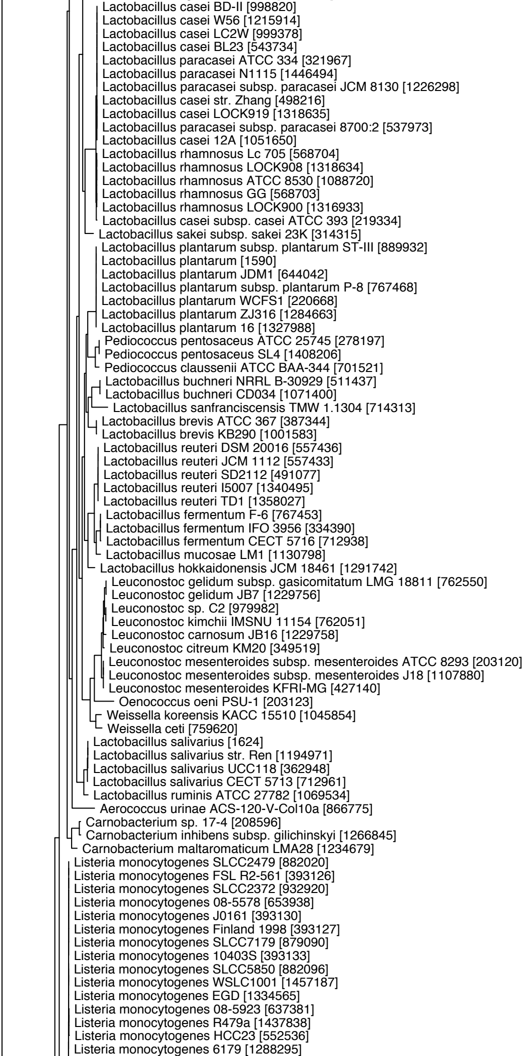
Lactobacillus casei BD-II [998820]
Lactobacillus casei W56 [1215914]
Lactobacillus casei LC2W [999378]
Lactobacillus casei BL23 [543734]
Lactobacillus paracasei ATCC 334 [321967]
Lactobacillus paracasei N1115 [1446494]
Lactobacillus paracasei subsp. paracasei JCM 8130 [1226298]
Lactobacillus casei str. Zhang [498216]
Lactobacillus casei LOCK919 [1318635]
Lactobacillus paracasei subsp. paracasei 8700:2 [537973]
Lactobacillus casei 12A [1051650]
Lactobacillus rhamnosus Lc 705 [568704]
Lactobacillus rhamnosus LOCK908 [1318634]
Lactobacillus rhamnosus ATCC 8530 [1088720]
Lactobacillus rhamnosus GG [568703]
Lactobacillus rhamnosus LOCK900 [1316933]
Lactobacillus casei subsp. casei ATCC 393 [219334]
Lactobacillus sakei subsp. sakei 23K [314315]
Lactobacillus plantarum subsp. plantarum ST-III [889932]
Lactobacillus plantarum [1590]
Lactobacillus plantarum JDM1 [644042]
Lactobacillus plantarum subsp. plantarum P-8 [767468]
Lactobacillus plantarum WCFS1 [220668]
Lactobacillus plantarum ZJ316 [1284663]
Lactobacillus plantarum 16 [1327988]
Pediococcus pentosaceus ATCC 25745 [278197]
Pediococcus pentosaceus SL4 [1408206]
Pediococcus claussenii ATCC BAA-344 [701521]
Lactobacillus buchneri NRRL B-30929 [511437]
Lactobacillus buchneri CD034 [1071400]
Lactobacillus sanfranciscensis TMW 1.1304 [714313]
Lactobacillus brevis ATCC 367 [387344]
Lactobacillus brevis KB290 [1001583]
Lactobacillus reuteri DSM 20016 [557436]
Lactobacillus reuteri JCM 1112 [557433]
Lactobacillus reuteri SD2112 [491077]
Lactobacillus reuteri I5007 [1340495]
Lactobacillus reuteri TD1 [1358027]
Lactobacillus fermentum F-6 [767453]
Lactobacillus fermentum IFO 3956 [334390]
Lactobacillus fermentum CECT 5716 [712938]
Lactobacillus mucosae LM1 [1130798]
Lactobacillus hokkaidonensis JCM 18461 [1291742]
Leuconostoc gelidum subsp. gasicomitatum LMG 18811 [762550]
Leuconostoc gelidum JB7 [1229756]
Leuconostoc sp. C2 [979982]
Leuconostoc kimchii IMSNU 11154 [762051]
Leuconostoc carnosum JB16 [1229758]
Leuconostoc citreum KM20 [349519]
Leuconostoc mesenteroides subsp. mesenteroides ATCC 8293 [203120]
Leuconostoc mesenteroides subsp. mesenteroides J18 [1107880]
Leuconostoc mesenteroides KFRI-MG [427140]
Oenococcus oeni PSU-1 [203123]
Weissella koreensis KACC 15510 [1045854]
Weissella ceti [759620]
Lactobacillus salivarius [1624]
Lactobacillus salivarius str. Ren [1194971]
Lactobacillus salivarius UCC118 [362948]
Lactobacillus salivarius CECT 5713 [712961]
Lactobacillus ruminis ATCC 27782 [1069534]
Aerococcus urinae ACS-120-V-Col10a [866775]
Carnobacterium sp. 17-4 [208596]
Carnobacterium inhibens subsp. gilichinskyi [1266845]
Carnobacterium maltaromaticum LMA28 [1234679]
Listeria monocytogenes SLCC2479 [882020]
Listeria monocytogenes FSL R2-561 [393126]
Listeria monocytogenes SLCC2372 [932920]
Listeria monocytogenes 08-5578 [653938]
Listeria monocytogenes J0161 [393130]
Listeria monocytogenes Finland 1998 [393127]
Listeria monocytogenes SLCC7179 [879090]
Listeria monocytogenes 10403S [393133]
Listeria monocytogenes SLCC5850 [882096]
Listeria monocytogenes WSLC1001 [1457187]
Listeria monocytogenes EGD [1334565]
Listeria monocytogenes 08-5923 [637381]
Listeria monocytogenes R479a [1437838]
Listeria monocytogenes HCC23 [552536]
Listeria monocytogenes 6179 [1288295]

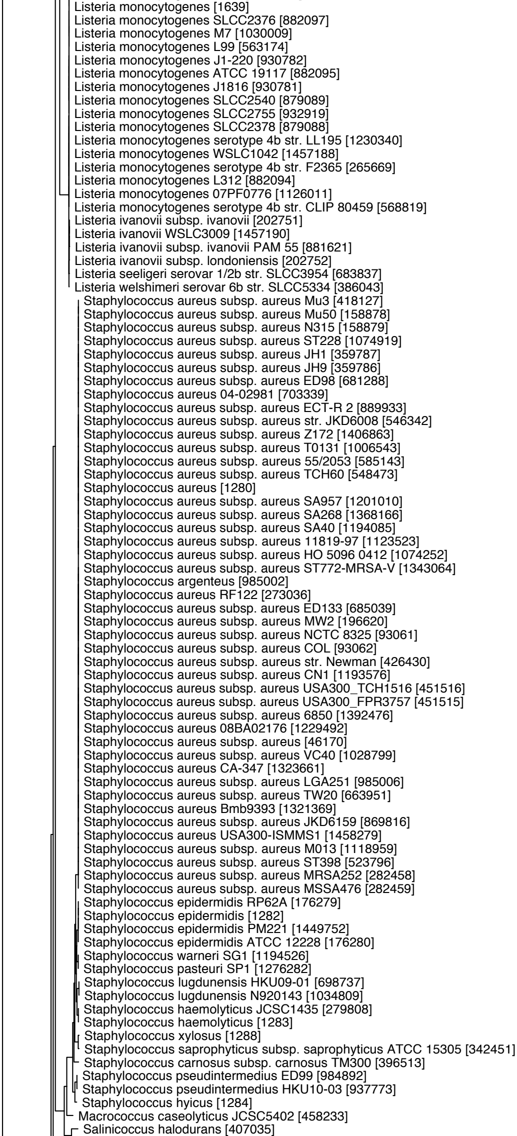
Listeria monocytogenes [1639]
Listeria monocytogenes SLCC2376 [882097]
Listeria monocytogenes M7 [1030009]
Listeria monocytogenes L99 [563174]
Listeria monocytogenes J1-220 [930782]
Listeria monocytogenes ATCC 19117 [882095]
Listeria monocytogenes J1816 [930781]
Listeria monocytogenes SLCC2540 [879089]
Listeria monocytogenes SLCC2755 [932919]
Listeria monocytogenes SLCC2378 [879088]
Listeria monocytogenes serotype 4b str. LL195 [1230340]
Listeria monocytogenes WSLC1042 [1457188]
Listeria monocytogenes serotype 4b str. F2365 [265669]
Listeria monocytogenes L312 [882094]
Listeria monocytogenes 07PF0776 [1126011]
Listeria monocytogenes serotype 4b str. CLIP 80459 [568819]
Listeria ivanovii subsp. ivanovii [202751]
Listeria ivanovii WSLC3009 [1457190]
Listeria ivanovii subsp. ivanovii PAM 55 [881621]
Listeria ivanovii subsp. londoniensis [202752]
Listeria seeligeri serovar 1/2b str. SLCC3954 [683837]
Listeria welshimeri serovar 6b str. SLCC5334 [386043]
Staphylococcus aureus subsp. aureus Mu3 [418127]
Staphylococcus aureus subsp. aureus Mu50 [158878]
Staphylococcus aureus subsp. aureus N315 [158879]
Staphylococcus aureus subsp. aureus ST228 [1074919]
Staphylococcus aureus subsp. aureus JH1 [359787]
Staphylococcus aureus subsp. aureus JH9 [359786]
Staphylococcus aureus subsp. aureus ED98 [681288]
Staphylococcus aureus 04-02981 [703339]
Staphylococcus aureus subsp. aureus ECT-R 2 [889933]
Staphylococcus aureus subsp. aureus str. JKD6008 [546342]
Staphylococcus aureus subsp. aureus Z172 [1406863]
Staphylococcus aureus subsp. aureus T0131 [1006543]
Staphylococcus aureus subsp. aureus 55/2053 [585143]
Staphylococcus aureus subsp. aureus TCH60 [548473]
Staphylococcus aureus [1280]
Staphylococcus aureus subsp. aureus SA957 [1201010]
Staphylococcus aureus subsp. aureus SA268 [1368166]
Staphylococcus aureus subsp. aureus SA40 [1194085]
Staphylococcus aureus subsp. aureus 11819-97 [1123523]
Staphylococcus aureus subsp. aureus HO 5096 0412 [1074252]
Staphylococcus aureus subsp. aureus ST772-MRSA-V [1343064]
Staphylococcus argenteus [985002]
Staphylococcus aureus RF122 [273036]
Staphylococcus aureus subsp. aureus ED133 [685039]
Staphylococcus aureus subsp. aureus MW2 [196620]
Staphylococcus aureus subsp. aureus NCTC 8325 [93061]
Staphylococcus aureus subsp. aureus COL [93062]
Staphylococcus aureus subsp. aureus str. Newman [426430]
Staphylococcus aureus subsp. aureus CN1 [1193576]
Staphylococcus aureus subsp. aureus USA300_TCH1516 [451516]
Staphylococcus aureus subsp. aureus USA300_FPR3757 [451515]
Staphylococcus aureus subsp. aureus 6850 [1392476]
Staphylococcus aureus 08BA02176 [1229492]
Staphylococcus aureus subsp. aureus [46170]
Staphylococcus aureus subsp. aureus VC40 [1028799]
Staphylococcus aureus CA-347 [1323661]
Staphylococcus aureus subsp. aureus LGA251 [985006]
Staphylococcus aureus subsp. aureus TW20 [663951]
Staphylococcus aureus Bmb9393 [1321369]
Staphylococcus aureus subsp. aureus JKD6159 [869816]
Staphylococcus aureus USA300-ISMMS1 [1458279]
Staphylococcus aureus subsp. aureus M013 [1118959]
Staphylococcus aureus subsp. aureus ST398 [523796]
Staphylococcus aureus subsp. aureus MRSA252 [282458]
Staphylococcus aureus subsp. aureus MSSA476 [282459]
Staphylococcus epidermidis RP62A [176279]
Staphylococcus epidermidis [1282]
Staphylococcus epidermidis PM221 [1449752]
Staphylococcus epidermidis ATCC 12228 [176280]
Staphylococcus warneri SG1 [1194526]
Staphylococcus pasteuri SP1 [1276282]
Staphylococcus lugdunensis HKU09-01 [698737]
Staphylococcus lugdunensis N920143 [1034809]
Staphylococcus haemolyticus JCSC1435 [279808]
Staphylococcus haemolyticus [1283]
Staphylococcus xylosus [1288]
Staphylococcus saprophyticus subsp. saprophyticus ATCC 15305 [342451]
Staphylococcus carnosus subsp. carnosus TM300 [396513]
Staphylococcus pseudintermedius ED99 [984892]
Staphylococcus pseudintermedius HKU10-03 [937773]
Staphylococcus hyicus [1284]
Macrococcus caseolyticus JCSC5402 [458233]
Salinicoccus halodurans [407035]

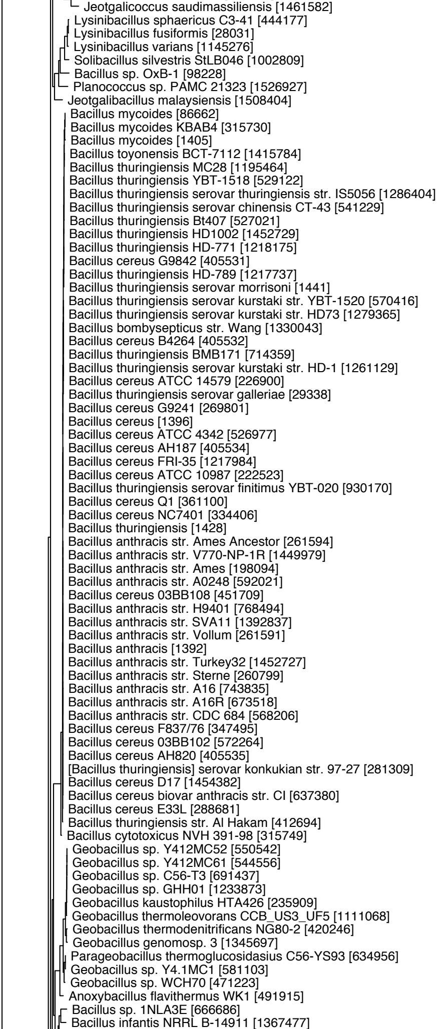
Jeotgalicoccus saudimassiliensis [1461582]
Lysinibacillus sphaericus C3-41 [444177]
Lysinibacillus fusiformis [28031]
Lysinibacillus varians [1145276]
Solibacillus silvestris StLB046 [1002809]
Bacillus sp. OxB-1 [98228]
Planococcus sp. PAMC 21323 [1526927]
Jeotgalibacillus malaysiensis [1508404]
Bacillus mycoides [86662]
Bacillus mycoides KBAB4 [315730]
Bacillus mycoides [1405]
Bacillus toyonensis BCT-7112 [1415784]
Bacillus thuringiensis MC28 [1195464]
Bacillus thuringiensis YBT-1518 [529122]
Bacillus thuringiensis serovar thuringiensis str. IS5056 [1286404]
Bacillus thuringiensis serovar chinensis CT-43 [541229]
Bacillus thuringiensis Bt407 [527021]
Bacillus thuringiensis HD1002 [1452729]
Bacillus thuringiensis HD-771 [1218175]
Bacillus cereus G9842 [405531]
Bacillus thuringiensis HD-789 [1217737]
Bacillus thuringiensis serovar morrisoni [1441]
Bacillus thuringiensis serovar kurstaki str. YBT-1520 [570416]
Bacillus thuringiensis serovar kurstaki str. HD73 [1279365]
Bacillus bombysepticus str. Wang [1330043]
Bacillus cereus B4264 [405532]
Bacillus thuringiensis BMB171 [714359]
Bacillus thuringiensis serovar kurstaki str. HD-1 [1261129]
Bacillus cereus ATCC 14579 [226900]
Bacillus thuringiensis serovar galleriae [29338]
Bacillus cereus G9241 [269801]
Bacillus cereus [1396]
Bacillus cereus ATCC 4342 [526977]
Bacillus cereus AH187 [405534]
Bacillus cereus FRI-35 [1217984]
Bacillus cereus ATCC 10987 [222523]
Bacillus thuringiensis serovar finitimus YBT-020 [930170]
Bacillus cereus Q1 [361100]
Bacillus cereus NC7401 [334406]
Bacillus thuringiensis [1428]
Bacillus anthracis str. Ames Ancestor [261594]
Bacillus anthracis str. V770-NP-1R [1449979]
Bacillus anthracis str. Ames [198094]
Bacillus anthracis str. A0248 [592021]
Bacillus cereus 03BB108 [451709]
Bacillus anthracis str. H9401 [768494]
Bacillus anthracis str. SVA11 [1392837]
Bacillus anthracis str. Vollum [261591]
Bacillus anthracis [1392]
Bacillus anthracis str. Turkey32 [1452727]
Bacillus anthracis str. Sterne [260799]
Bacillus anthracis str. A16 [743835]
Bacillus anthracis str. A16R [673518]
Bacillus anthracis str. CDC 684 [568206]
Bacillus cereus F837/76 [347495]
Bacillus cereus 03BB102 [572264]
Bacillus cereus AH820 [405535]
[Bacillus thuringiensis] serovar konkukian str. 97-27 [281309]
Bacillus cereus D17 [1454382]
Bacillus cereus biovar anthracis str. CI [637380]
Bacillus cereus E33L [288681]
Bacillus thuringiensis str. Al Hakam [412694]
Bacillus cytotoxicus NVH 391-98 [315749]
Geobacillus sp. Y412MC52 [550542]
Geobacillus sp. Y412MC61 [544556]
Geobacillus sp. C56-T3 [691437]
Geobacillus sp. GHH01 [1233873]
Geobacillus kaustophilus HTA426 [235909]
Geobacillus thermoleovorans CCB_US3_UF5 [1111068]
Geobacillus thermodenitrificans NG80-2 [420246]
Geobacillus genomosp. 3 [1345697]
Parageobacillus thermoglucosidasius C56-YS93 [634956]
Geobacillus sp. Y4.1MC1 [581103]
Geobacillus sp. WCH70 [471223]
Anoxybacillus flavithermus WK1 [491915]
Bacillus sp. 1NLA3E [666686]
Bacillus infantis NRRL B-14911 [1367477]

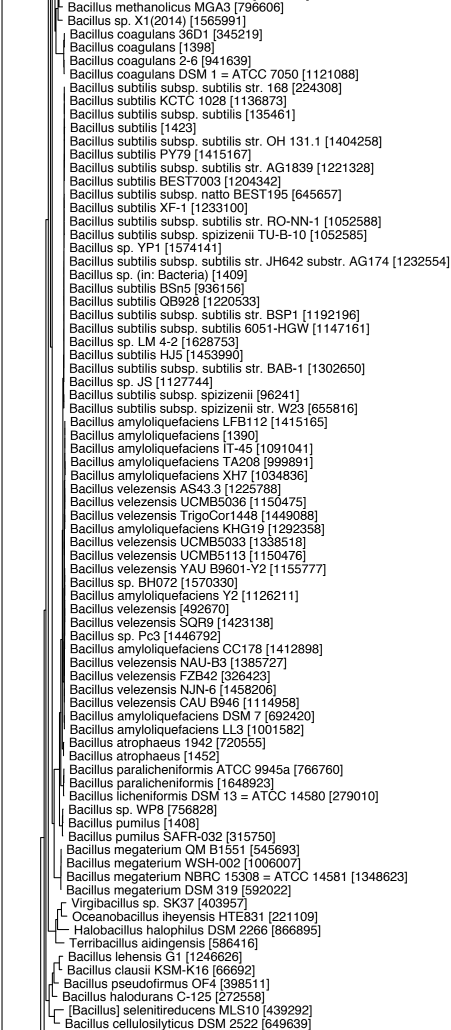
Bacillus methanolicus MGA3 [796606]
Bacillus sp. X1(2014) [1565991]
Bacillus coagulans 36D1 [345219]
Bacillus coagulans [1398]
Bacillus coagulans 2-6 [941639]
Bacillus coagulans DSM 1 = ATCC 7050 [1121088]
Bacillus subtilis subsp. subtilis str. 168 [224308]
Bacillus subtilis KCTC 1028 [1136873]
Bacillus subtilis subsp. subtilis [135461]
Bacillus subtilis [1423]
Bacillus subtilis subsp. subtilis str. OH 131.1 [1404258]
Bacillus subtilis PY79 [1415167]
Bacillus subtilis subsp. subtilis str. AG1839 [1221328]
Bacillus subtilis BEST7003 [1204342]
Bacillus subtilis subsp. natto BEST195 [645657]
Bacillus subtilis XF-1 [1233100]
Bacillus subtilis subsp. subtilis str. RO-NN-1 [1052588]
Bacillus subtilis subsp. spizizenii TU-B-10 [1052585]
Bacillus sp. YP1 [1574141]
Bacillus subtilis subsp. subtilis str. JH642 substr. AG174 [1232554]
Bacillus sp. (in: Bacteria) [1409]
Bacillus subtilis BSn5 [936156]
Bacillus subtilis QB928 [1220533]
Bacillus subtilis subsp. subtilis str. BSP1 [1192196]
Bacillus subtilis subsp. subtilis 6051-HGW [1147161]
Bacillus sp. LM 4-2 [1628753]
Bacillus subtilis HJ5 [1453990]
Bacillus subtilis subsp. subtilis str. BAB-1 [1302650]
Bacillus sp. JS [1127744]
Bacillus subtilis subsp. spizizenii [96241]
Bacillus subtilis subsp. spizizenii str. W23 [655816]
Bacillus amyloliquefaciens LFB112 [1415165]
Bacillus amyloliquefaciens [1390]
Bacillus amyloliquefaciens IT-45 [1091041]
Bacillus amyloliquefaciens TA208 [999891]
Bacillus amyloliquefaciens XH7 [1034836]
Bacillus velezensis AS43.3 [1225788]
Bacillus velezensis UCMB5036 [1150475]
Bacillus velezensis TrigoCor1448 [1449088]
Bacillus amyloliquefaciens KHG19 [1292358]
Bacillus velezensis UCMB5033 [1338518]
Bacillus velezensis UCMB5113 [1150476]
Bacillus velezensis YAU B9601-Y2 [1155777]
Bacillus sp. BH072 [1570330]
Bacillus amyloliquefaciens Y2 [1126211]
Bacillus velezensis [492670]
Bacillus velezensis SQR9 [1423138]
Bacillus sp. Pc3 [1446792]
Bacillus amyloliquefaciens CC178 [1412898]
Bacillus velezensis NAU-B3 [1385727]
Bacillus velezensis FZB42 [326423]
Bacillus velezensis NJN-6 [1458206]
Bacillus velezensis CAU B946 [1114958]
Bacillus amyloliquefaciens DSM 7 [692420]
Bacillus amyloliquefaciens LL3 [1001582]
Bacillus atrophaeus 1942 [720555]
Bacillus atrophaeus [1452]
Bacillus paralicheniformis ATCC 9945a [766760]
Bacillus paralicheniformis [1648923]
Bacillus licheniformis DSM 13 = ATCC 14580 [279010]
Bacillus sp. WP8 [756828]
Bacillus pumilus [1408]
Bacillus pumilus SAFR-032 [315750]
Bacillus megaterium QM B1551 [545693]
Bacillus megaterium WSH-002 [1006007]
Bacillus megaterium NBRC 15308 = ATCC 14581 [1348623]
Bacillus megaterium DSM 319 [592022]
Virgibacillus sp. SK37 [403957]
Oceanobacillus iheyensis HTE831 [221109]
Halobacillus halophilus DSM 2266 [866895]
Terribacillus aidingensis [586416]
Bacillus lehensis G1 [1246626]
Bacillus clausii KSM-K16 [66692]
Bacillus pseudofirmus OF4 [398511]
Bacillus halodurans C-125 [272558]
[Bacillus] selenitireducens MLS10 [439292]
Bacillus cellulosilyticus DSM 2522 [649639]

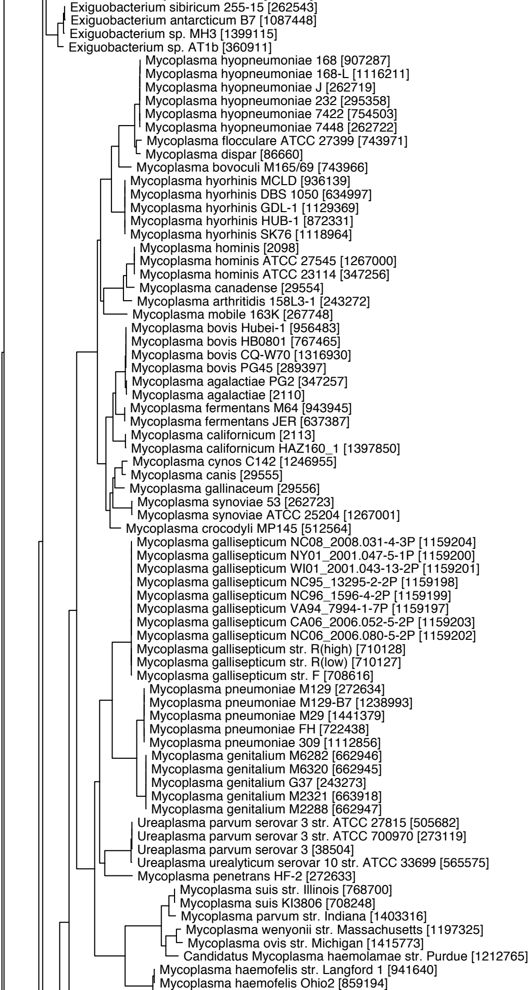

Exiguobacterium sibiricum 255-15 [262543]
Exiguobacterium antarcticum B7 [1087448]
Exiguobacterium sp. MH3 [1399115]
Exiguobacterium sp. AT1b [360911]
Mycoplasma hyopneumoniae 168 [907287]
Mycoplasma hyopneumoniae 168-L [1116211]
Mycoplasma hyopneumoniae J [262719]
Mycoplasma hyopneumoniae 232 [295358]
Mycoplasma hyopneumoniae 7422 [754503]
Mycoplasma hyopneumoniae 7448 [262722]
Mycoplasma flocculare ATCC 27399 [743971]
Mycoplasma dispar [86660]
Mycoplasma bovoculi M165/69 [743966]
Mycoplasma hyorhinis MCLD [936139]
Mycoplasma hyorhinis DBS 1050 [634997]
Mycoplasma hyorhinis GDL-1 [1129369]
Mycoplasma hyorhinis HUB-1 [872331]
Mycoplasma hyorhinis SK76 [1118964]
Mycoplasma hominis [2098]
Mycoplasma hominis ATCC 27545 [1267000]
Mycoplasma hominis ATCC 23114 [347256]
Mycoplasma canadense [29554]
Mycoplasma arthritidis 158L3-1 [243272]
Mycoplasma mobile 163K [267748]
Mycoplasma bovis Hubei-1 [956483]
Mycoplasma bovis HB0801 [767465]
Mycoplasma bovis CQ-W70 [1316930]
Mycoplasma bovis PG45 [289397]
Mycoplasma agalactiae PG2 [347257]
Mycoplasma agalactiae [2110]
Mycoplasma fermentans M64 [943945]
Mycoplasma fermentans JER [637387]
Mycoplasma californicum [2113]
Mycoplasma californicum HAZ160_1 [1397850]
Mycoplasma cynos C142 [1246955]
Mycoplasma canis [29555]
Mycoplasma gallinaceum [29556]
Mycoplasma synoviae 53 [262723]
Mycoplasma synoviae ATCC 25204 [1267001]
Mycoplasma crocodyli MP145 [512564]
Mycoplasma gallisepticum NC08_2008.031-4-3P [1159204]
Mycoplasma gallisepticum NY01_2001.047-5-1P [1159200]
Mycoplasma gallisepticum WI01_2001.043-13-2P [1159201]
Mycoplasma gallisepticum NC95_13295-2-2P [1159198]
Mycoplasma gallisepticum NC96_1596-4-2P [1159199]
Mycoplasma gallisepticum VA94_7994-1-7P [1159197]
Mycoplasma gallisepticum CA06_2006.052-5-2P [1159203]
Mycoplasma gallisepticum NC06_2006.080-5-2P [1159202]
Mycoplasma gallisepticum str. R(high) [710128]
Mycoplasma gallisepticum str. R(low) [710127]
Mycoplasma gallisepticum str. F [708616]
Mycoplasma pneumoniae M129 [272634]
Mycoplasma pneumoniae M129-B7 [1238993]
Mycoplasma pneumoniae M29 [1441379]
Mycoplasma pneumoniae FH [722438]
Mycoplasma pneumoniae 309 [1112856]
Mycoplasma genitalium M6282 [662946]
Mycoplasma genitalium M6320 [662945]
Mycoplasma genitalium G37 [243273]
Mycoplasma genitalium M2321 [663918]
Mycoplasma genitalium M2288 [662947]
Ureaplasma parvum serovar 3 str. ATCC 27815 [505682]
Ureaplasma parvum serovar 3 str. ATCC 700970 [273119]
Ureaplasma parvum serovar 3 [38504]
Ureaplasma urealyticum serovar 10 str. ATCC 33699 [565575]
Mycoplasma penetrans HF-2 [272633]
Mycoplasma suis str. Illinois [768700]
Mycoplasma suis KI3806 [708248]
Mycoplasma parvum str. Indiana [1403316]
Mycoplasma wenyonii str. Massachusetts [1197325]
Mycoplasma ovis str. Michigan [1415773]
Candidatus Mycoplasma haemolamae str. Purdue [1212765]
Mycoplasma haemofelis str. Langford 1 [941640]
Mycoplasma haemofelis Ohio2 [859194]

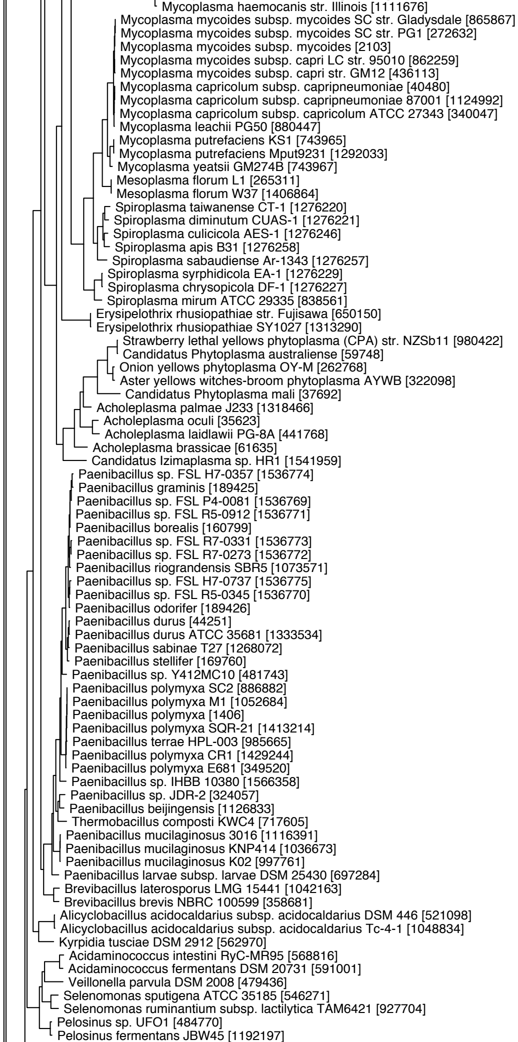
Mycoplasma haemocanis str. Illinois [1111676]
Mycoplasma mycoides subsp. mycoides SC str. Gladysdale [865867]
Mycoplasma mycoides subsp. mycoides SC str. PG1 [272632]
Mycoplasma mycoides subsp. mycoides [2103]
Mycoplasma mycoides subsp. capri LC str. 95010 [862259]
Mycoplasma mycoides subsp. capri str. GM12 [436113]
Mycoplasma capricolum subsp. capripneumoniae [40480]
Mycoplasma capricolum subsp. capripneumoniae 87001 [1124992]
Mycoplasma capricolum subsp. capricolum ATCC 27343 [340047]
Mycoplasma leachii PG50 [880447]
Mycoplasma putrefaciens KS1 [743965]
Mycoplasma putrefaciens Mput9231 [1292033]
Mycoplasma yeatsii GM274B [743967]
Mesoplasma florum L1 [265311]
Mesoplasma florum W37 [1406864]
Spiroplasma taiwanense CT-1 [1276220]
Spiroplasma diminutum CUAS-1 [1276221]
Spiroplasma culicicola AES-1 [1276246]
Spiroplasma apis B31 [1276258]
Spiroplasma sabaudiense Ar-1343 [1276257]
Spiroplasma syrphidicola EA-1 [1276229]
Spiroplasma chrysopicola DF-1 [1276227]
Spiroplasma mirum ATCC 29335 [838561]
Erysipelothrix rhusiopathiae str. Fujisawa [650150]
Erysipelothrix rhusiopathiae SY1027 [1313290]
Strawberry lethal yellows phytoplasma (CPA) str. NZSb11 [980422]
Candidatus Phytoplasma australiense [59748]
Onion yellows phytoplasma OY-M [262768]
Aster yellows witches-broom phytoplasma AYWB [322098]
Candidatus Phytoplasma mali [37692]
Acholeplasma palmae J233 [1318466]
Acholeplasma oculi [35623]
Acholeplasma laidlawii PG-8A [441768]
Acholeplasma brassicae [61635]
Candidatus Izimaplasma sp. HR1 [1541959]
Paenibacillus sp. FSL H7-0357 [1536774]
Paenibacillus graminis [189425]
Paenibacillus sp. FSL P4-0081 [1536769]
Paenibacillus sp. FSL R5-0912 [1536771]
Paenibacillus borealis [160799]
Paenibacillus sp. FSL R7-0331 [1536773]
Paenibacillus sp. FSL R7-0273 [1536772]
Paenibacillus riograndensis SBR5 [1073571]
Paenibacillus sp. FSL H7-0737 [1536775]
Paenibacillus sp. FSL R5-0345 [1536770]
Paenibacillus odorifer [189426]
Paenibacillus durus [44251]
Paenibacillus durus ATCC 35681 [1333534]
Paenibacillus sabinae T27 [1268072]
Paenibacillus stellifer [169760]
Paenibacillus sp. Y412MC10 [481743]
Paenibacillus polymyxa SC2 [886882]
Paenibacillus polymyxa M1 [1052684]
Paenibacillus polymyxa [1406]
Paenibacillus polymyxa SQR-21 [1413214]
Paenibacillus terrae HPL-003 [985665]
Paenibacillus polymyxa CR1 [1429244]
Paenibacillus polymyxa E681 [349520]
Paenibacillus sp. IHBB 10380 [1566358]
Paenibacillus sp. JDR-2 [324057]
Paenibacillus beijingensis [1126833]
Thermobacillus composti KWC4 [717605]
Paenibacillus mucilaginosus 3016 [1116391]
Paenibacillus mucilaginosus KNP414 [1036673]
Paenibacillus mucilaginosus K02 [997761]
Paenibacillus larvae subsp. larvae DSM 25430 [697284]
Brevibacillus laterosporus LMG 15441 [1042163]
Brevibacillus brevis NBRC 100599 [358681]
Alicyclobacillus acidocaldarius subsp. acidocaldarius DSM 446 [521098]
Alicyclobacillus acidocaldarius subsp. acidocaldarius Tc-4-1 [1048834]
Kyrpidia tusciae DSM 2912 [562970]
Acidaminococcus intestini RyC-MR95 [568816]
Acidaminococcus fermentans DSM 20731 [591001]
Veillonella parvula DSM 2008 [479436]
Selenomonas sputigena ATCC 35185 [546271]
Selenomonas ruminantium subsp. lactilytica TAM6421 [927704]
Pelosinus sp. UFO1 [484770]
Pelosinus fermentans JBW45 [1192197]

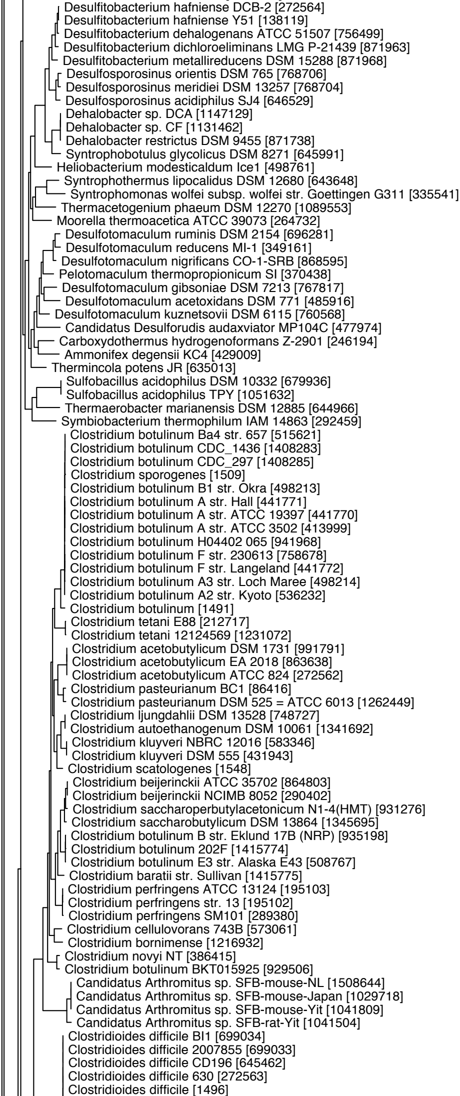
Desulfitobacterium hafniense DCB-2 [272564]
Desulfitobacterium hafniense Y51 [138119]
Desulfitobacterium dehalogenans ATCC 51507 [756499]
Desulfitobacterium dichloroeliminans LMG P-21439 [871963]
Desulfitobacterium metallireducens DSM 15288 [871968]
Desulfosporosinus orientis DSM 765 [768706]
Desulfosporosinus meridiei DSM 13257 [768704]
Desulfosporosinus acidiphilus SJ4 [646529]
Dehalobacter sp. DCA [1147129]
Dehalobacter sp. CF [1131462]
Dehalobacter restrictus DSM 9455 [871738]
Syntrophobotulus glycolicus DSM 8271 [645991]
Heliobacterium modesticaldum Ice1 [498761]
Syntrophothermus lipocalidus DSM 12680 [643648]
Syntrophomonas wolfei subsp. wolfei str. Goettingen G311 [335541]
Thermacetogenium phaeum DSM 12270 [1089553]
Moorella thermoacetica ATCC 39073 [264732]
Desulfotomaculum ruminis DSM 2154 [696281]
Desulfotomaculum reducens MI-1 [349161]
Desulfotomaculum nigrificans CO-1-SRB [868595]
Pelotomaculum thermopropionicum SI [370438]
Desulfotomaculum gibsoniae DSM 7213 [767817]
Desulfotomaculum acetoxidans DSM 771 [485916]
Desulfotomaculum kuznetsovii DSM 6115 [760568]
Candidatus Desulforudis audaxviator MP104C [477974]
Carboxydothermus hydrogenoformans Z-2901 [246194]
Ammonifex degensii KC4 [429009]
Thermincola potens JR [635013]
Sulfobacillus acidophilus DSM 10332 [679936]
Sulfobacillus acidophilus TPY [1051632]
Thermaerobacter marianensis DSM 12885 [644966]
Symbiobacterium thermophilum IAM 14863 [292459]
Clostridium botulinum Ba4 str. 657 [515621]
Clostridium botulinum CDC_1436 [1408283]
Clostridium botulinum CDC_297 [1408285]
Clostridium sporogenes [1509]
Clostridium botulinum B1 str. Okra [498213]
Clostridium botulinum A str. Hall [441771]
Clostridium botulinum A str. ATCC 19397 [441770]
Clostridium botulinum A str. ATCC 3502 [413999]
Clostridium botulinum H04402 065 [941968]
Clostridium botulinum F str. 230613 [758678]
Clostridium botulinum F str. Langeland [441772]
Clostridium botulinum A3 str. Loch Maree [498214]
Clostridium botulinum A2 str. Kyoto [536232]
Clostridium botulinum [1491]
Clostridium tetani E88 [212717]
Clostridium tetani 12124569 [1231072]
Clostridium acetobutylicum DSM 1731 [991791]
Clostridium acetobutylicum EA 2018 [863638]
Clostridium acetobutylicum ATCC 824 [272562]
Clostridium pasteurianum BC1 [86416]
Clostridium pasteurianum DSM 525 = ATCC 6013 [1262449]
Clostridium ljungdahlii DSM 13528 [748727]
Clostridium autoethanogenum DSM 10061 [1341692]
Clostridium kluyveri NBRC 12016 [583346]
Clostridium kluyveri DSM 555 [431943]
Clostridium scatologenes [1548]
Clostridium beijerinckii ATCC 35702 [864803]
Clostridium beijerinckii NCIMB 8052 [290402]
Clostridium saccharoperbutylacetonicum N1-4(HMT) [931276]
Clostridium saccharobutylicum DSM 13864 [1345695]
Clostridium botulinum B str. Eklund 17B (NRP) [935198]
Clostridium botulinum 202F [1415774]
Clostridium botulinum E3 str. Alaska E43 [508767]
Clostridium baratii str. Sullivan [1415775]
Clostridium perfringens ATCC 13124 [195103]
Clostridium perfringens str. 13 [195102]
Clostridium perfringens SM101 [289380]
Clostridium cellulovorans 743B [573061]
Clostridium bornimense [1216932]
Clostridium novyi NT [386415]
Clostridium botulinum BKT015925 [929506]
Candidatus Arthromitus sp. SFB-mouse-NL [1508644]
Candidatus Arthromitus sp. SFB-mouse-Japan [1029718]
Candidatus Arthromitus sp. SFB-mouse-Yit [1041809]
Candidatus Arthromitus sp. SFB-rat-Yit [1041504]
Clostridioides difficile BI1 [699034]
Clostridioides difficile 2007855 [699033]
Clostridioides difficile CD196 [645462]
Clostridioides difficile 630 [272563]
Clostridioides difficile [1496]

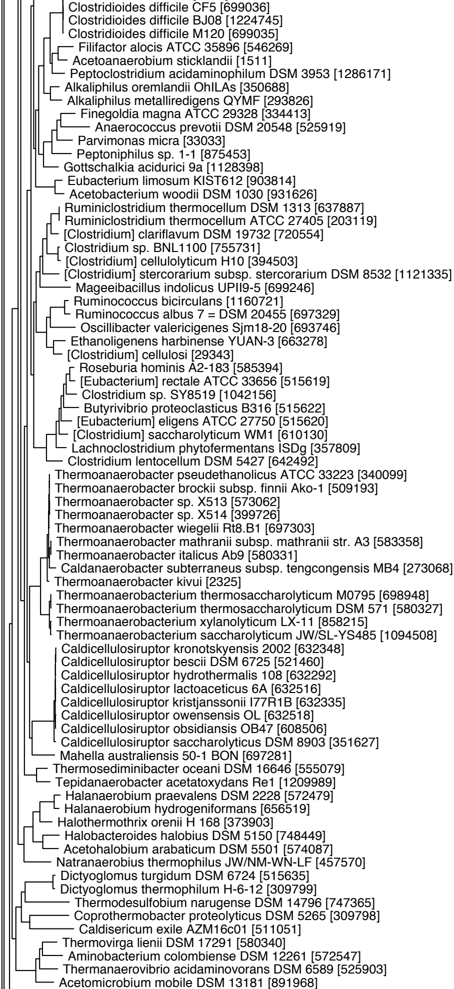
Clostridioides difficile CF5 [699036]
Clostridioides difficile BJ08 [1224745]
Clostridioides difficile M120 [699035]
Filifactor alocis ATCC 35896 [546269]
Acetoanaerobium sticklandii [1511]
Peptoclostridium acidaminophilum DSM 3953 [1286171]
Alkaliphilus oremlandii OhILAs [350688]
Alkaliphilus metalliredigens QYMF [293826]
Finegoldia magna ATCC 29328 [334413]
Anaerococcus prevotii DSM 20548 [525919]
Parvimonas micra [33033]
Peptoniphilus sp. 1-1 [875453]
Gottschalkia acidurici 9a [1128398]
Eubacterium limosum KIST612 [903814]
Acetobacterium woodii DSM 1030 [931626]
Ruminiclostridium thermocellum DSM 1313 [637887]
Ruminiclostridium thermocellum ATCC 27405 [203119]
[Clostridium] clariflavum DSM 19732 [720554]
Clostridium sp. BNL1100 [755731]
[Clostridium] cellulolyticum H10 [394503]
[Clostridium] stercorarium subsp. stercorarium DSM 8532 [1121335]
Mageeibacillus indolicus UPII9-5 [699246]
Ruminococcus bicirculans [1160721]
Ruminococcus albus 7 = DSM 20455 [697329]
Oscillibacter valericigenes Sjm18-20 [693746]
Ethanoligenens harbinense YUAN-3 [663278]
[Clostridium] cellulosi [29343]
Roseburia hominis A2-183 [585394]
[Eubacterium] rectale ATCC 33656 [515619]
Clostridium sp. SY8519 [1042156]
Butyrivibrio proteoclasticus B316 [515622]
[Eubacterium] eligens ATCC 27750 [515620]
[Clostridium] saccharolyticum WM1 [610130]
Lachnoclostridium phytofermentans ISDg [357809]
Clostridium lentocellum DSM 5427 [642492]
Thermoanaerobacter pseudethanolicus ATCC 33223 [340099]
Thermoanaerobacter brockii subsp. finnii Ako-1 [509193]
Thermoanaerobacter sp. X513 [573062]
Thermoanaerobacter sp. X514 [399726]
Thermoanaerobacter wiegelii Rt8.B1 [697303]
Thermoanaerobacter mathranii subsp. mathranii str. A3 [583358]
Thermoanaerobacter italicus Ab9 [580331]
Caldanaerobacter subterraneus subsp. tengcongensis MB4 [273068]
Thermoanaerobacter kivui [2325]
Thermoanaerobacterium thermosaccharolyticum M0795 [698948]
Thermoanaerobacterium thermosaccharolyticum DSM 571 [580327]
Thermoanaerobacterium xylanolyticum LX-11 [858215]
Thermoanaerobacterium saccharolyticum JW/SL-YS485 [1094508]
Caldicellulosiruptor kronotskyensis 2002 [632348]
Caldicellulosiruptor bescii DSM 6725 [521460]
Caldicellulosiruptor hydrothermalis 108 [632292]
Caldicellulosiruptor lactoaceticus 6A [632516]
Caldicellulosiruptor kristjanssonii I77R1B [632335]
Caldicellulosiruptor owensensis OL [632518]
Caldicellulosiruptor obsidiansis OB47 [608506]
Caldicellulosiruptor saccharolyticus DSM 8903 [351627]
Mahella australiensis 50-1 BON [697281]
Thermosediminibacter oceani DSM 16646 [555079]
Tepidanaerobacter acetatoxydans Re1 [1209989]
Halanaerobium praevalens DSM 2228 [572479]
Halanaerobium hydrogeniformans [656519]
Halothermothrix orenii H 168 [373903]
Halobacteroides halobius DSM 5150 [748449]
Acetohalobium arabaticum DSM 5501 [574087]
Natranaerobius thermophilus JW/NM-WN-LF [457570]
Dictyoglomus turgidum DSM 6724 [515635]
Dictyoglomus thermophilum H-6-12 [309799]
Thermodesulfobium narugense DSM 14796 [747365]
Coprothermobacter proteolyticus DSM 5265 [309798]
Caldisericum exile AZM16c01 [511051]
Thermovirga lienii DSM 17291 [580340]
Aminobacterium colombiense DSM 12261 [572547]
Thermanaerovibrio acidaminovorans DSM 6589 [525903]
Acetomicrobium mobile DSM 13181 [891968]

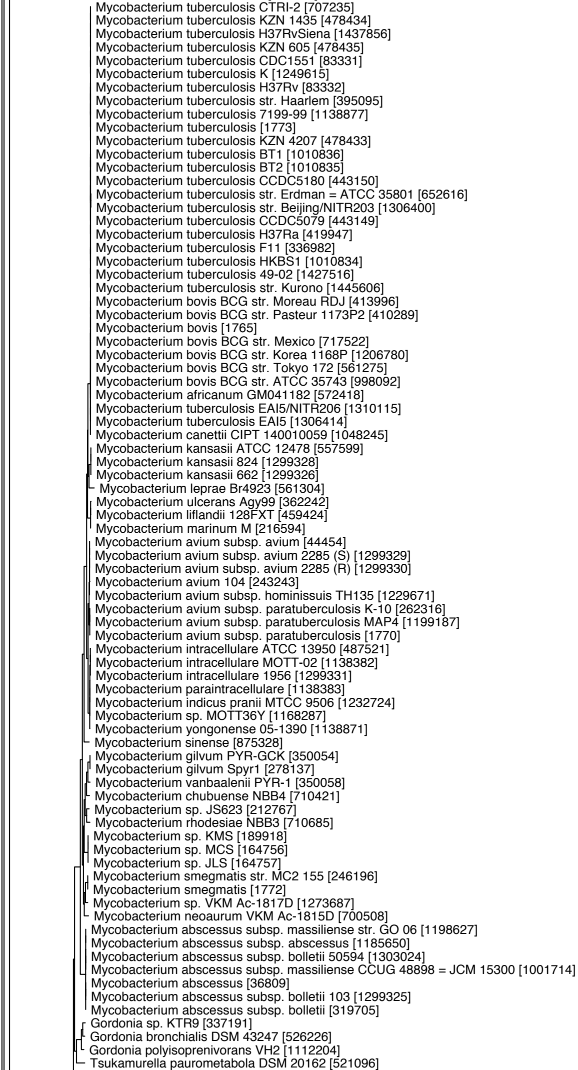
Mycobacterium tuberculosis CTRI-2 [707235]
Mycobacterium tuberculosis KZN 1435 [478434]
Mycobacterium tuberculosis H37RvSiena [1437856]
Mycobacterium tuberculosis KZN 605 [478435]
Mycobacterium tuberculosis CDC1551 [83331]
Mycobacterium tuberculosis K [1249615]
Mycobacterium tuberculosis H37Rv [83332]
Mycobacterium tuberculosis str. Haarlem [395095]
Mycobacterium tuberculosis 7199-99 [1138877]
Mycobacterium tuberculosis [1773]
Mycobacterium tuberculosis KZN 4207 [478433]
Mycobacterium tuberculosis BT1 [1010836]
Mycobacterium tuberculosis BT2 [1010835]
Mycobacterium tuberculosis CCDC5180 [443150]
Mycobacterium tuberculosis str. Erdman = ATCC 35801 [652616]
Mycobacterium tuberculosis str. Beijing/NITR203 [1306400]
Mycobacterium tuberculosis CCDC5079 [443149]
Mycobacterium tuberculosis H37Ra [419947]
Mycobacterium tuberculosis F11 [336982]
Mycobacterium tuberculosis HKBS1 [1010834]
Mycobacterium tuberculosis 49-02 [1427516]
Mycobacterium tuberculosis str. Kurono [1445606]
Mycobacterium bovis BCG str. Moreau RDJ [413996]
Mycobacterium bovis BCG str. Pasteur 1173P2 [410289]
Mycobacterium bovis [1765]
Mycobacterium bovis BCG str. Mexico [717522]
Mycobacterium bovis BCG str. Korea 1168P [1206780]
Mycobacterium bovis BCG str. Tokyo 172 [561275]
Mycobacterium bovis BCG str. ATCC 35743 [998092]
Mycobacterium africanum GM041182 [572418]
Mycobacterium tuberculosis EAI5/NITR206 [1310115]
Mycobacterium tuberculosis EAI5 [1306414]
Mycobacterium canettii CIPT 140010059 [1048245]
Mycobacterium kansasii ATCC 12478 [557599]
Mycobacterium kansasii 824 [1299328]
Mycobacterium kansasii 662 [1299326]
Mycobacterium leprae Br4923 [561304]
Mycobacterium ulcerans Agy99 [362242]
Mycobacterium liflandii 128FXT [459424]
Mycobacterium marinum M [216594]
Mycobacterium avium subsp. avium [44454]
Mycobacterium avium subsp. avium 2285 (S) [1299329]
Mycobacterium avium subsp. avium 2285 (R) [1299330]
Mycobacterium avium 104 [243243]
Mycobacterium avium subsp. hominissuis TH135 [1229671]
Mycobacterium avium subsp. paratuberculosis K-10 [262316]
Mycobacterium avium subsp. paratuberculosis MAP4 [1199187]
Mycobacterium avium subsp. paratuberculosis [1770]
Mycobacterium intracellulare ATCC 13950 [487521]
Mycobacterium intracellulare MOTT-02 [1138382]
Mycobacterium intracellulare 1956 [1299331]
Mycobacterium paraintracellulare [1138383]
Mycobacterium indicus pranii MTCC 9506 [1232724]
Mycobacterium sp. MOTT36Y [1168287]
Mycobacterium yongonense 05-1390 [1138871]
Mycobacterium sinense [875328]
Mycobacterium gilvum PYR-GCK [350054]
Mycobacterium gilvum Spyr1 [278137]
Mycobacterium vanbaalenii PYR-1 [350058]
Mycobacterium chubuense NBB4 [710421]
Mycobacterium sp. JS623 [212767]
Mycobacterium rhodesiae NBB3 [710685]
Mycobacterium sp. KMS [189918]
Mycobacterium sp. MCS [164756]
Mycobacterium sp. JLS [164757]
Mycobacterium smegmatis str. MC2 155 [246196]
Mycobacterium smegmatis [1772]
Mycobacterium sp. VKM Ac-1817D [1273687]
Mycobacterium neoaurum VKM Ac-1815D [700508]
Mycobacterium abscessus subsp. massiliense str. GO 06 [1198627]
Mycobacterium abscessus subsp. abscessus [1185650]
Mycobacterium abscessus subsp. bolletii 50594 [1303024]
Mycobacterium abscessus subsp. massiliense CCUG 48898 = JCM 15300 [1001714]
Mycobacterium abscessus [36809]
Mycobacterium abscessus subsp. bolletii 103 [1299325]
Mycobacterium abscessus subsp. bolletii [319705]
Gordonia sp. KTR9 [337191]
Gordonia bronchialis DSM 43247 [526226]
Gordonia polyisoprenivorans VH2 [1112204]
Tsukamurella paurometabola DSM 20162 [521096]

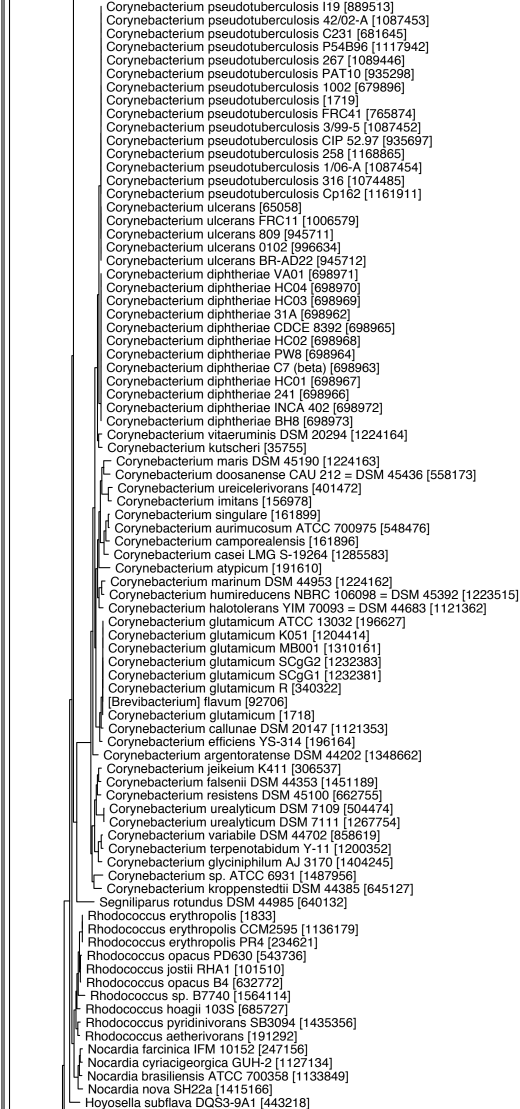
Corynebacterium pseudotuberculosis I19 [889513]
Corynebacterium pseudotuberculosis 42/02-A [1087453]
Corynebacterium pseudotuberculosis C231 [681645]
Corynebacterium pseudotuberculosis P54B96 [1117942]
Corynebacterium pseudotuberculosis 267 [1089446]
Corynebacterium pseudotuberculosis PAT10 [935298]
Corynebacterium pseudotuberculosis 1002 [679896]
Corynebacterium pseudotuberculosis [1719]
Corynebacterium pseudotuberculosis FRC41 [765874]
Corynebacterium pseudotuberculosis 3/99-5 [1087452]
Corynebacterium pseudotuberculosis CIP 52.97 [935697]
Corynebacterium pseudotuberculosis 258 [1168865]
Corynebacterium pseudotuberculosis 1/06-A [1087454]
Corynebacterium pseudotuberculosis 316 [1074485]
Corynebacterium pseudotuberculosis Cp162 [1161911]
Corynebacterium ulcerans [65058]
Corynebacterium ulcerans FRC11 [1006579]
Corynebacterium ulcerans 809 [945711]
Corynebacterium ulcerans 0102 [996634]
Corynebacterium ulcerans BR-AD22 [945712]
Corynebacterium diphtheriae VA01 [698971]
Corynebacterium diphtheriae HC04 [698970]
Corynebacterium diphtheriae HC03 [698969]
Corynebacterium diphtheriae 31A [698962]
Corynebacterium diphtheriae CDCE 8392 [698965]
Corynebacterium diphtheriae HC02 [698968]
Corynebacterium diphtheriae PW8 [698964]
Corynebacterium diphtheriae C7 (beta) [698963]
Corynebacterium diphtheriae HC01 [698967]
Corynebacterium diphtheriae 241 [698966]
Corynebacterium diphtheriae INCA 402 [698972]
Corynebacterium diphtheriae BH8 [698973]
Corynebacterium vitaeruminis DSM 20294 [1224164]
Corynebacterium kutscheri [35755]
Corynebacterium maris DSM 45190 [1224163]
Corynebacterium doosanense CAU 212 = DSM 45436 [558173]
Corynebacterium ureicelerivorans [401472]
Corynebacterium imitans [156978]
Corynebacterium singulare [161899]
Corynebacterium aurimucosum ATCC 700975 [548476]
Corynebacterium camporealensis [161896]
Corynebacterium casei LMG S-19264 [1285583]
Corynebacterium atypicum [191610]
Corynebacterium marinum DSM 44953 [1224162]
Corynebacterium humireducens NBRC 106098 = DSM 45392 [1223515]
Corynebacterium halotolerans YIM 70093 = DSM 44683 [1121362]
Corynebacterium glutamicum ATCC 13032 [196627]
Corynebacterium glutamicum K051 [1204414]
Corynebacterium glutamicum MB001 [1310161]
Corynebacterium glutamicum SCgG2 [1232383]
Corynebacterium glutamicum SCgG1 [1232381]
Corynebacterium glutamicum R [340322]
[Brevibacterium] flavum [92706]
Corynebacterium glutamicum [1718]
Corynebacterium callunae DSM 20147 [1121353]
Corynebacterium efficiens YS-314 [196164]
Corynebacterium argentoratense DSM 44202 [1348662]
Corynebacterium jeikeium K411 [306537]
Corynebacterium falsenii DSM 44353 [1451189]
Corynebacterium resistens DSM 45100 [662755]
Corynebacterium urealyticum DSM 7109 [504474]
Corynebacterium urealyticum DSM 7111 [1267754]
Corynebacterium variabile DSM 44702 [858619]
Corynebacterium terpenotabidum Y-11 [1200352]
Corynebacterium glyciniphilum AJ 3170 [1404245]
Corynebacterium sp. ATCC 6931 [1487956]
Corynebacterium kroppenstedtii DSM 44385 [645127]
Segniliparus rotundus DSM 44985 [640132]
Rhodococcus erythropolis [1833]
Rhodococcus erythropolis CCM2595 [1136179]
Rhodococcus erythropolis PR4 [234621]
Rhodococcus opacus PD630 [543736]
Rhodococcus jostii RHA1 [101510]
Rhodococcus opacus B4 [632772]
Rhodococcus sp. B7740 [1564114]
Rhodococcus hoagii 103S [685727]
Rhodococcus pyridinivorans SB3094 [1435356]
Rhodococcus aetherivorans [191292]
Nocardia farcinica IFM 10152 [247156]
Nocardia cyriacigeorgica GUH-2 [1127134]
Nocardia brasiliensis ATCC 700358 [1133849]
Nocardia nova SH22a [1415166]
Hoyosella subflava DQS3-9A1 [443218]

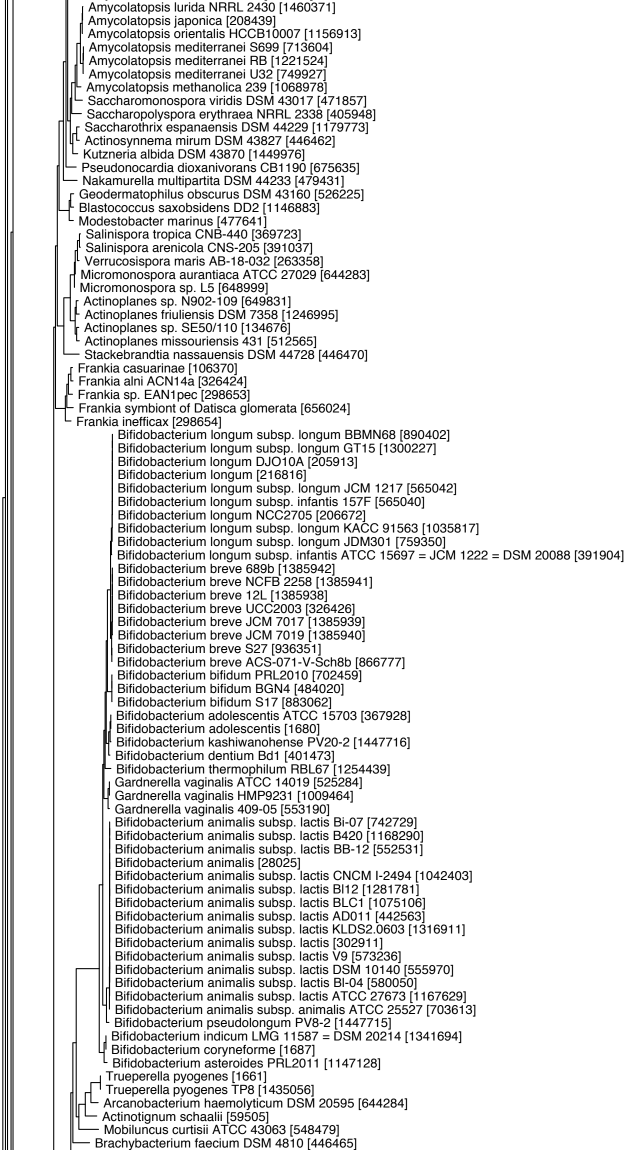
Amycolatopsis lurida NRRL 2430 [1460371]
Amycolatopsis japonica [208439]
Amycolatopsis orientalis HCCB10007 [1156913]
Amycolatopsis mediterranei S699 [713604]
Amycolatopsis mediterranei RB [1221524]
Amycolatopsis mediterranei U32 [749927]
Amycolatopsis methanolica 239 [1068978]
Saccharomonospora viridis DSM 43017 [471857]
Saccharopolyspora erythraea NRRL 2338 [405948]
Saccharothrix espanaensis DSM 44229 [1179773]
Actinosynnema mirum DSM 43827 [446462]
Kutzneria albida DSM 43870 [1449976]
Pseudonocardia dioxanivorans CB1190 [675635]
Nakamurella multipartita DSM 44233 [479431]
Geodermatophilus obscurus DSM 43160 [526225]
Blastococcus saxobsidens DD2 [1146883]
Modestobacter marinus [477641]
Salinispora tropica CNB-440 [369723]
Salinispora arenicola CNS-205 [391037]
Verrucosispora maris AB-18-032 [263358]
Micromonospora aurantiaca ATCC 27029 [644283]
Micromonospora sp. L5 [648999]
Actinoplanes sp. N902-109 [649831]
Actinoplanes friuliensis DSM 7358 [1246995]
Actinoplanes sp. SE50/110 [134676]
Actinoplanes missouriensis 431 [512565]
Stackebrandtia nassauensis DSM 44728 [446470]
Frankia casuarinae [106370]
Frankia alni ACN14a [326424]
Frankia sp. EAN1pec [298653]
Frankia symbiont of Datisca glomerata [656024]
Frankia inefficax [298654]
Bifidobacterium longum subsp. longum BBMN68 [890402]
Bifidobacterium longum subsp. longum GT15 [1300227]
Bifidobacterium longum DJO10A [205913]
Bifidobacterium longum [216816]
Bifidobacterium longum subsp. longum JCM 1217 [565042]
Bifidobacterium longum subsp. infantis 157F [565040]
Bifidobacterium longum NCC2705 [206672]
Bifidobacterium longum subsp. longum KACC 91563 [1035817]
Bifidobacterium longum subsp. longum JDM301 [759350]
Bifidobacterium longum subsp. infantis ATCC 15697 = JCM 1222 = DSM 20088 [391904]
Bifidobacterium breve 689b [1385942]
Bifidobacterium breve NCFB 2258 [1385941]
Bifidobacterium breve 12L [1385938]
Bifidobacterium breve UCC2003 [326426]
Bifidobacterium breve JCM 7017 [1385939]
Bifidobacterium breve JCM 7019 [1385940]
Bifidobacterium breve S27 [936351]
Bifidobacterium breve ACS-071-V-Sch8b [866777]
Bifidobacterium bifidum PRL2010 [702459]
Bifidobacterium bifidum BGN4 [484020]
Bifidobacterium bifidum S17 [883062]
Bifidobacterium adolescentis ATCC 15703 [367928]
Bifidobacterium adolescentis [1680]
Bifidobacterium kashiwanohense PV20-2 [1447716]
Bifidobacterium dentium Bd1 [401473]
Bifidobacterium thermophilum RBL67 [1254439]
Gardnerella vaginalis ATCC 14019 [525284]
Gardnerella vaginalis HMP9231 [1009464]
Gardnerella vaginalis 409-05 [553190]
Bifidobacterium animalis subsp. lactis Bi-07 [742729]
Bifidobacterium animalis subsp. lactis B420 [1168290]
Bifidobacterium animalis subsp. lactis BB-12 [552531]
Bifidobacterium animalis [28025]
Bifidobacterium animalis subsp. lactis CNCM I-2494 [1042403]
Bifidobacterium animalis subsp. lactis Bl12 [1281781]
Bifidobacterium animalis subsp. lactis BLC1 [1075106]
Bifidobacterium animalis subsp. lactis AD011 [442563]
Bifidobacterium animalis subsp. lactis KLDS2.0603 [1316911]
Bifidobacterium animalis subsp. lactis [302911]
Bifidobacterium animalis subsp. lactis V9 [573236]
Bifidobacterium animalis subsp. lactis DSM 10140 [555970]
Bifidobacterium animalis subsp. lactis Bl-04 [580050]
Bifidobacterium animalis subsp. lactis ATCC 27673 [1167629]
Bifidobacterium animalis subsp. animalis ATCC 25527 [703613]
Bifidobacterium pseudolongum PV8-2 [1447715]
Bifidobacterium indicum LMG 11587 = DSM 20214 [1341694]
Bifidobacterium coryneforme [1687]
Bifidobacterium asteroides PRL2011 [1147128]
Trueperella pyogenes [1661]
Trueperella pyogenes TP8 [1435056]
Arcanobacterium haemolyticum DSM 20595 [644284]
Actinotignum schaalii [59505]
Mobiluncus curtisii ATCC 43063 [548479]
Brachybacterium faecium DSM 4810 [446465]

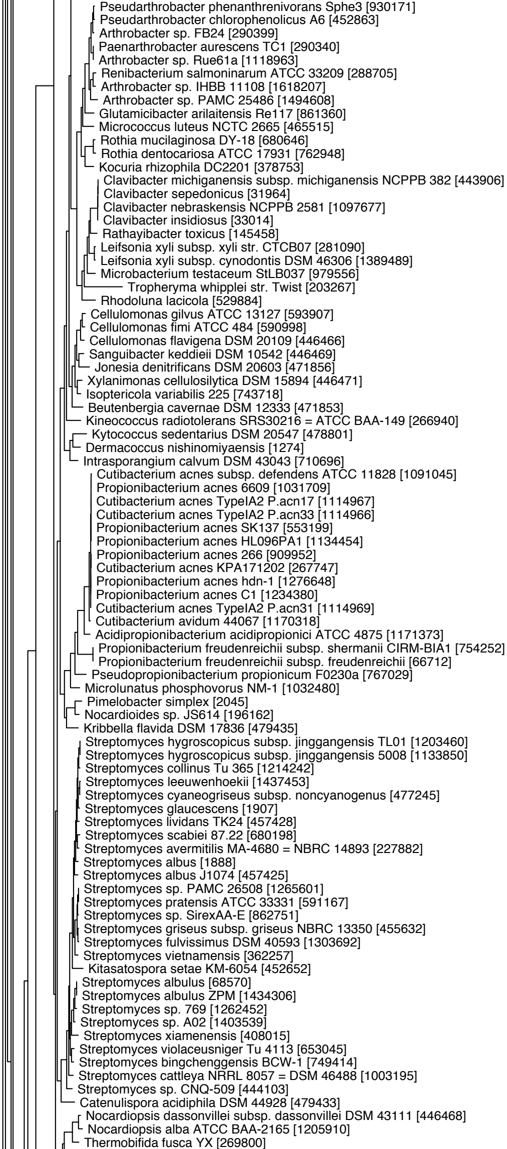

Pseudarthrobacter phenanthrenivorans Sphe3 [930171]
Pseudarthrobacter chlorophenolicus A6 [452863]
Arthrobacter sp. FB24 [290399]
Paenarthrobacter aurescens TC1 [290340]
Arthrobacter sp. Rue61a [1118963]
Renibacterium salmoninarum ATCC 33209 [288705]
Arthrobacter sp. IHBB 11108 [1618207]
Arthrobacter sp. PAMC 25486 [1494608]
Glutamicibacter arilaitensis Re117 [861360]
Micrococcus luteus NCTC 2665 [465515]
Rothia mucilaginosa DY-18 [680646]
Rothia dentocariosa ATCC 17931 [762948]
Kocuria rhizophila DC2201 [378753]
Clavibacter michiganensis subsp. michiganensis NCPPB 382 [443906]
Clavibacter sepedonicus [31964]
Clavibacter nebraskensis NCPPB 2581 [1097677]
Clavibacter insidiosus [33014]
Rathayibacter toxicus [145458]
Leifsonia xyli subsp. xyli str. CTCB07 [281090]
Leifsonia xyli subsp. cynodontis DSM 46306 [1389489]
Microbacterium testaceum StLB037 [979556]
Tropheryma whipplei str. Twist [203267]
Rhodoluna lacicola [529884]
Cellulomonas gilvus ATCC 13127 [593907]
Cellulomonas fimi ATCC 484 [590998]
Cellulomonas flavigena DSM 20109 [446466]
Sanguibacter keddieii DSM 10542 [446469]
Jonesia denitrificans DSM 20603 [471856]
Xylanimonas cellulosilytica DSM 15894 [446471]
Isoptericola variabilis 225 [743718]
Beutenbergia cavernae DSM 12333 [471853]
Kineococcus radiotolerans SRS30216 = ATCC BAA-149 [266940]
Kytococcus sedentarius DSM 20547 [478801]
Dermacoccus nishinomiyaensis [1274]
Intrasporangium calvum DSM 43043 [710696]
Cutibacterium acnes subsp. defendens ATCC 11828 [1091045]
Propionibacterium acnes 6609 [1031709]
Cutibacterium acnes TypeIA2 P.acn17 [1114967]
Cutibacterium acnes TypeIA2 P.acn33 [1114966]
Propionibacterium acnes SK137 [553199]
Propionibacterium acnes HL096PA1 [1134454]
Propionibacterium acnes 266 [909952]
Cutibacterium acnes KPA171202 [267747]
Propionibacterium acnes hdn-1 [1276648]
Propionibacterium acnes C1 [1234380]
Cutibacterium acnes TypeIA2 P.acn31 [1114969]
Cutibacterium avidum 44067 [1170318]
Acidipropionibacterium acidipropionici ATCC 4875 [1171373]
Propionibacterium freudenreichii subsp. shermanii CIRM-BIA1 [754252]
Propionibacterium freudenreichii subsp. freudenreichii [66712]
Pseudopropionibacterium propionicum F0230a [767029]
Microlunatus phosphovorus NM-1 [1032480]
Pimelobacter simplex [2045]
Nocardioides sp. JS614 [196162]
Kribbella flavida DSM 17836 [479435]
Streptomyces hygroscopicus subsp. jinggangensis TL01 [1203460]
Streptomyces hygroscopicus subsp. jinggangensis 5008 [1133850]
Streptomyces collinus Tu 365 [1214242]
Streptomyces leeuwenhoekii [1437453]
Streptomyces cyaneogriseus subsp. noncyanogenus [477245]
Streptomyces glaucescens [1907]
Streptomyces lividans TK24 [457428]
Streptomyces scabiei 87.22 [680198]
Streptomyces avermitilis MA-4680 = NBRC 14893 [227882]
Streptomyces albus [1888]
Streptomyces albus J1074 [457425]
Streptomyces sp. PAMC 26508 [1265601]
Streptomyces pratensis ATCC 33331 [591167]
Streptomyces sp. SirexAA-E [862751]
Streptomyces griseus subsp. griseus NBRC 13350 [455632]
Streptomyces fulvissimus DSM 40593 [1303692]
Streptomyces vietnamensis [362257]
Kitasatospora setae KM-6054 [452652]
Streptomyces albulus [68570]
Streptomyces albulus ZPM [1434306]
Streptomyces sp. 769 [1262452]
Streptomyces sp. A02 [1403539]
Streptomyces xiamenensis [408015]
Streptomyces violaceusniger Tu 4113 [653045]
Streptomyces bingchenggensis BCW-1 [749414]
Streptomyces cattleya NRRL 8057 = DSM 46488 [1003195]
Streptomyces sp. CNQ-509 [444103]
Catenulispora acidiphila DSM 44928 [479433]
Nocardiopsis dassonvillei subsp. dassonvillei DSM 43111 [446468]
Nocardiopsis alba ATCC BAA-2165 [1205910]
Thermobifida fusca YX [269800]

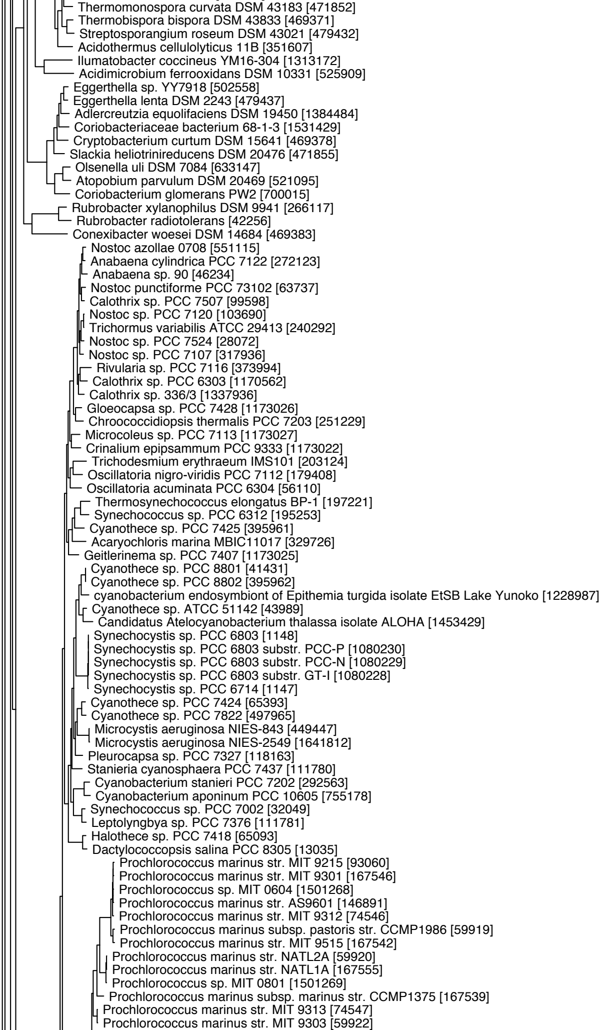
Thermomonospora curvata DSM 43183 [471852]
Thermobispora bispora DSM 43833 [469371]
Streptosporangium roseum DSM 43021 [479432]
Acidothermus cellulolyticus 11B [351607]
Ilumatobacter coccineus YM16-304 [1313172]
Acidimicrobium ferrooxidans DSM 10331 [525909]
Eggerthella sp. YY7918 [502558]
Eggerthella lenta DSM 2243 [479437]
Adlercreutzia equolifaciens DSM 19450 [1384484]
Coriobacteriaceae bacterium 68-1-3 [1531429]
Cryptobacterium curtum DSM 15641 [469378]
Slackia heliotrinireducens DSM 20476 [471855]
Olsenella uli DSM 7084 [633147]
Atopobium parvulum DSM 20469 [521095]
Coriobacterium glomerans PW2 [700015]
Rubrobacter xylanophilus DSM 9941 [266117]
Rubrobacter radiotolerans [42256]
Conexibacter woesei DSM 14684 [469383]
Nostoc azollae 0708 [551115]
Anabaena cylindrica PCC 7122 [272123]
Anabaena sp. 90 [46234]
Nostoc punctiforme PCC 73102 [63737]
Calothrix sp. PCC 7507 [99598]
Nostoc sp. PCC 7120 [103690]
Trichormus variabilis ATCC 29413 [240292]
Nostoc sp. PCC 7524 [28072]
Nostoc sp. PCC 7107 [317936]
Rivularia sp. PCC 7116 [373994]
Calothrix sp. PCC 6303 [1170562]
Calothrix sp. 336/3 [1337936]
Gloeocapsa sp. PCC 7428 [1173026]
Chroococcidiopsis thermalis PCC 7203 [251229]
Microcoleus sp. PCC 7113 [1173027]
Crinalium epipsammum PCC 9333 [1173022]
Trichodesmium erythraeum IMS101 [203124]
Oscillatoria nigro-viridis PCC 7112 [179408]
Oscillatoria acuminata PCC 6304 [56110]
Thermosynechococcus elongatus BP-1 [197221]
Synechococcus sp. PCC 6312 [195253]
Cyanothece sp. PCC 7425 [395961]
Acaryochloris marina MBIC11017 [329726]
Geitlerinema sp. PCC 7407 [1173025]
Cyanothece sp. PCC 8801 [41431]
Cyanothece sp. PCC 8802 [395962]
cyanobacterium endosymbiont of Epithemia turgida isolate EtSB Lake Yunoko [1228987]
Cyanothece sp. ATCC 51142 [43989]
Candidatus Atelocyanobacterium thalassa isolate ALOHA [1453429]
Synechocystis sp. PCC 6803 [1148]
Synechocystis sp. PCC 6803 substr. PCC-P [1080230]
Synechocystis sp. PCC 6803 substr. PCC-N [1080229]
Synechocystis sp. PCC 6803 substr. GT-I [1080228]
Synechocystis sp. PCC 6714 [1147]
Cyanothece sp. PCC 7424 [65393]
Cyanothece sp. PCC 7822 [497965]
Microcystis aeruginosa NIES-843 [449447]
Microcystis aeruginosa NIES-2549 [1641812]
Pleurocapsa sp. PCC 7327 [118163]
Stanieria cyanosphaera PCC 7437 [111780]
Cyanobacterium stanieri PCC 7202 [292563]
Cyanobacterium aponinum PCC 10605 [755178]
Synechococcus sp. PCC 7002 [32049]
Leptolyngbya sp. PCC 7376 [111781]
Halothece sp. PCC 7418 [65093]
Dactylococcopsis salina PCC 8305 [13035]
Prochlorococcus marinus str. MIT 9215 [93060]
Prochlorococcus marinus str. MIT 9301 [167546]
Prochlorococcus sp. MIT 0604 [1501268]
Prochlorococcus marinus str. AS9601 [146891]
Prochlorococcus marinus str. MIT 9312 [74546]
Prochlorococcus marinus subsp. pastoris str. CCMP1986 [59919]
Prochlorococcus marinus str. MIT 9515 [167542]
Prochlorococcus marinus str. NATL2A [59920]
Prochlorococcus marinus str. NATL1A [167555]
Prochlorococcus sp. MIT 0801 [1501269]
Prochlorococcus marinus subsp. marinus str. CCMP1375 [167539]
Prochlorococcus marinus str. MIT 9313 [74547]
Prochlorococcus marinus str. MIT 9303 [59922]

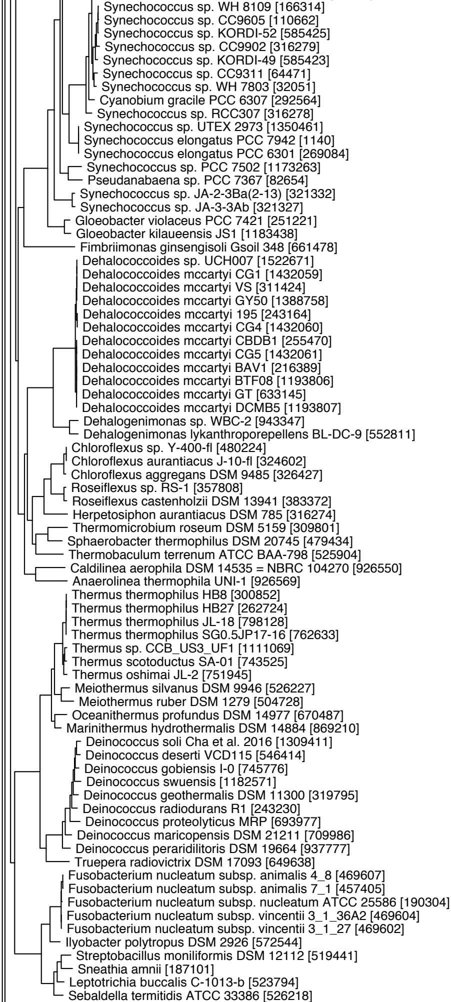

Synechococcus sp. WH 8109 [166314]
Synechococcus sp. CC9605 [110662]
Synechococcus sp. KORDI-52 [585425]
Synechococcus sp. CC9902 [316279]
Synechococcus sp. KORDI-49 [585423]
Synechococcus sp. CC9311 [64471]
Synechococcus sp. WH 7803 [32051]
Cyanobium gracile PCC 6307 [292564]
Synechococcus sp. RCC307 [316278]
Synechococcus sp. UTEX 2973 [1350461]
Synechococcus elongatus PCC 7942 [1140]
Synechococcus elongatus PCC 6301 [269084]
Synechococcus sp. PCC 7502 [1173263]
Pseudanabaena sp. PCC 7367 [82654]
Synechococcus sp. JA-2-3Ba(2-13) [321332]
Synechococcus sp. JA-3-3Ab [321327]
Gloeobacter violaceus PCC 7421 [251221]
Gloeobacter kilaueensis JS1 [1183438]
Fimbriimonas ginsengisoli Gsoil 348 [661478]
Dehalococcoides sp. UCH007 [1522671]
Dehalococcoides mccartyi CG1 [1432059]
Dehalococcoides mccartyi VS [311424]
Dehalococcoides mccartyi GY50 [1388758]
Dehalococcoides mccartyi 195 [243164]
Dehalococcoides mccartyi CG4 [1432060]
Dehalococcoides mccartyi CBDB1 [255470]
Dehalococcoides mccartyi CG5 [1432061]
Dehalococcoides mccartyi BAV1 [216389]
Dehalococcoides mccartyi BTF08 [1193806]
Dehalococcoides mccartyi GT [633145]
Dehalococcoides mccartyi DCMB5 [1193807]
Dehalogenimonas sp. WBC-2 [943347]
Dehalogenimonas lykanthroporepellens BL-DC-9 [552811]
Chloroflexus sp. Y-400-fl [480224]
Chloroflexus aurantiacus J-10-fl [324602]
Chloroflexus aggregans DSM 9485 [326427]
Roseiflexus sp. RS-1 [357808]
Roseiflexus castenholzii DSM 13941 [383372]
Herpetosiphon aurantiacus DSM 785 [316274]
Thermomicrobium roseum DSM 5159 [309801]
Sphaerobacter thermophilus DSM 20745 [479434]
Thermobaculum terrenum ATCC BAA-798 [525904]
Caldilinea aerophila DSM 14535 = NBRC 104270 [926550]
Anaerolinea thermophila UNI-1 [926569]
Thermus thermophilus HB8 [300852]
Thermus thermophilus HB27 [262724]
Thermus thermophilus JL-18 [798128]
Thermus thermophilus SG0.5JP17-16 [762633]
Thermus sp. CCB_US3_UF1 [1111069]
Thermus scotoductus SA-01 [743525]
Thermus oshimai JL-2 [751945]
Meiothermus silvanus DSM 9946 [526227]
Meiothermus ruber DSM 1279 [504728]
Oceanithermus profundus DSM 14977 [670487]
Marinithermus hydrothermalis DSM 14884 [869210]
Deinococcus soli Cha et al. 2016 [1309411]
Deinococcus deserti VCD115 [546414]
Deinococcus gobiensis I-0 [745776]
Deinococcus swuensis [1182571]
Deinococcus geothermalis DSM 11300 [319795]
Deinococcus radiodurans R1 [243230]
Deinococcus proteolyticus MRP [693977]
Deinococcus maricopensis DSM 21211 [709986]
Deinococcus peraridilitoris DSM 19664 [937777]
Truepera radiovictrix DSM 17093 [649638]
Fusobacterium nucleatum subsp. animalis 4_8 [469607]
Fusobacterium nucleatum subsp. animalis 7_1 [457405]
Fusobacterium nucleatum subsp. nucleatum ATCC 25586 [190304]
Fusobacterium nucleatum subsp. vincentii 3_1_36A2 [469604]
Fusobacterium nucleatum subsp. vincentii 3_1_27 [469602]
Ilyobacter polytropus DSM 2926 [572544]
Streptobacillus moniliformis DSM 12112 [519441]
Sneathia amnii [187101]
Leptotrichia buccalis C-1013-b [523794]
Sebaldella termitidis ATCC 33386 [526218]

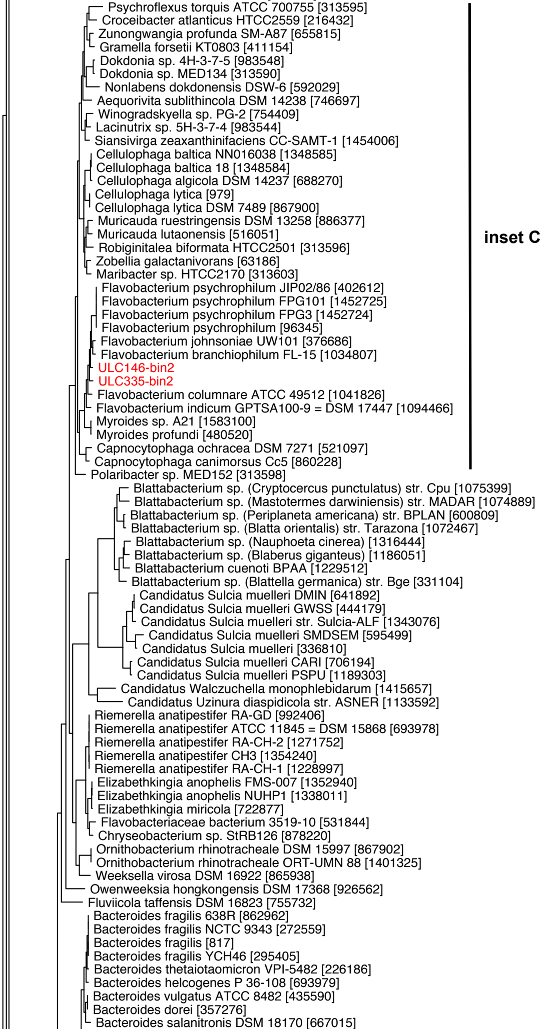
Psychroflexus torquis ATCC 700755 [313595]
Croceibacter atlanticus HTCC2559 [216432]
Zunongwangia profunda SM-A87 [655815]
Gramella forsetii KT0803 [411154]
Dokdonia sp. 4H-3-7-5 [983548]
Dokdonia sp. MED134 [313590]
Nonlabens dokdonensis DSW-6 [592029]
Aequorivita sublithincola DSM 14238 [746697]
Winogradskyella sp. PG-2 [754409]
Lacinutrix sp. 5H-3-7-4 [983544]
Siansivirga zeaxanthinifaciens CC-SAMT-1 [1454006]
Cellulophaga baltica NN016038 [1348585]
Cellulophaga baltica 18 [1348584]
Cellulophaga algicola DSM 14237 [688270]
Cellulophaga lytica [979]
Cellulophaga lytica DSM 7489 [867900]
Muricauda ruestringensis DSM 13258 [886377]
Muricauda lutaonensis [516051]
Robiginitalea biformata HTCC2501 [313596]
Zobellia galactanivorans [63186]
Maribacter sp. HTCC2170 [313603]
Flavobacterium psychrophilum JIP02/86 [402612]
Flavobacterium psychrophilum FPG101 [1452725]
Flavobacterium psychrophilum FPG3 [1452724]
Flavobacterium psychrophilum [96345]
Flavobacterium johnsoniae UW101 [376686]
Flavobacterium branchiophilum FL-15 [1034807]
ULC146-bin2
ULC335-bin2
Flavobacterium columnare ATCC 49512 [1041826]
Flavobacterium indicum GPTSA100-9 = DSM 17447 [1094466]
Myroides sp. A21 [1583100]
Myroides profundi [480520]
Capnocytophaga ochracea DSM 7271 [521097]
Capnocytophaga canimorsus Cc5 [860228]
inset C
Polaribacter sp. MED152 [313598]
Blattabacterium sp. (Cryptocercus punctulatus) str. Cpu [1075399]
Blattabacterium sp. (Mastotermes darwiniensis) str. MADAR [1074889]
Blattabacterium sp. (Periplaneta americana) str. BPLAN [600809]
Blattabacterium sp. (Blatta orientalis) str. Tarazona [1072467]
Blattabacterium sp. (Nauphoeta cinerea) [1316444]
Blattabacterium sp. (Blaberus giganteus) [1186051]
Blattabacterium cuenoti BPAA [1229512]
Blattabacterium sp. (Blattella germanica) str. Bge [331104]
Candidatus Sulcia muelleri DMIN [641892]
Candidatus Sulcia muelleri GWSS [444179]
Candidatus Sulcia muelleri str. Sulcia-ALF [1343076]
Candidatus Sulcia muelleri SMDSEM [595499]
Candidatus Sulcia muelleri [336810]
Candidatus Sulcia muelleri CARI [706194]
Candidatus Sulcia muelleri PSPU [1189303]
Candidatus Walczuchella monophlebidarum [1415657]
Candidatus Uzinura diaspidicola str. ASNER [1133592]
Riemerella anatipestifer RA-GD [992406]
Riemerella anatipestifer ATCC 11845 = DSM 15868 [693978]
Riemerella anatipestifer RA-CH-2 [1271752]
Riemerella anatipestifer CH3 [1354240]
Riemerella anatipestifer RA-CH-1 [1228997]
Elizabethkingia anophelis FMS-007 [1352940]
Elizabethkingia anophelis NUHP1 [1338011]
Elizabethkingia miricola [722877]
Flavobacteriaceae bacterium 3519-10 [531844]
Chryseobacterium sp. StRB126 [878220]
Ornithobacterium rhinotracheale DSM 15997 [867902]
Ornithobacterium rhinotracheale ORT-UMN 88 [1401325]
Weeksella virosa DSM 16922 [865938]
Owenweeksia hongkongensis DSM 17368 [926562]
Fluviicola taffensis DSM 16823 [755732]
Bacteroides fragilis 638R [862962]
Bacteroides fragilis NCTC 9343 [272559]
Bacteroides fragilis [817]
Bacteroides fragilis YCH46 [295405]
Bacteroides thetaiotaomicron VPI-5482 [226186]
Bacteroides helcogenes P 36-108 [693979]
Bacteroides vulgatus ATCC 8482 [435590]
Bacteroides dorei [357276]
Bacteroides salanitronis DSM 18170 [667015]

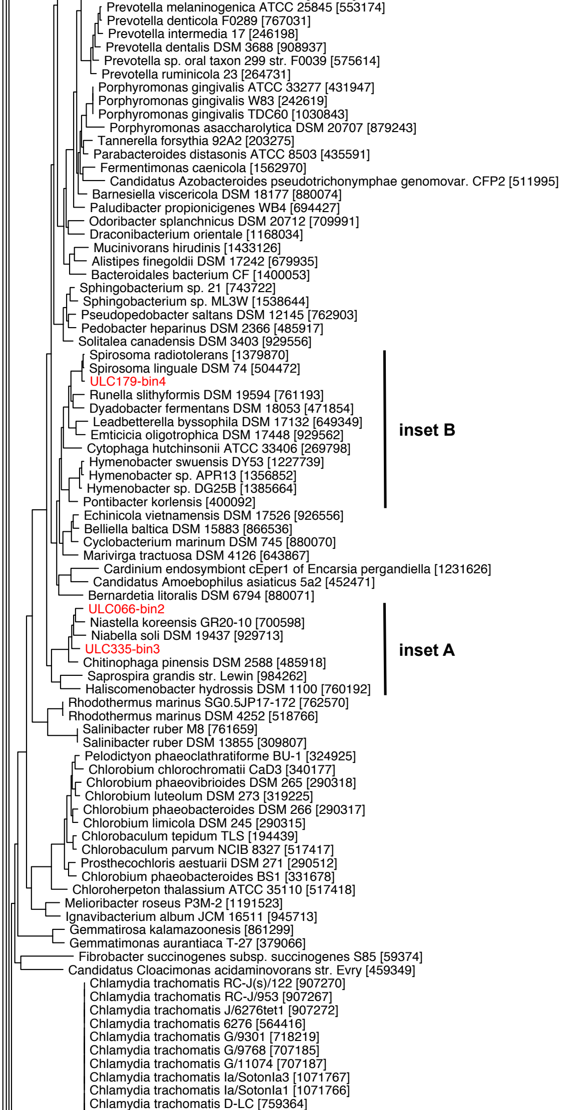
Prevotella melaninogenica ATCC 25845 [553174]
Prevotella denticola F0289 [767031]
Prevotella intermedia 17 [246198]
Prevotella dentalis DSM 3688 [908937]
Prevotella sp. oral taxon 299 str. F0039 [575614]
Prevotella ruminicola 23 [264731]
Porphyromonas gingivalis ATCC 33277 [431947]
Porphyromonas gingivalis W83 [242619]
Porphyromonas gingivalis TDC60 [1030843]
Porphyromonas asaccharolytica DSM 20707 [879243]
Tannerella forsythia 92A2 [203275]
Parabacteroides distasonis ATCC 8503 [435591]
Fermentimonas caenicola [1562970]
Candidatus Azobacteroides pseudotrichonymphae genomovar. CFP2 [511995]
Barnesiella viscericola DSM 18177 [880074]
Paludibacter propionicigenes WB4 [694427]
Odoribacter splanchnicus DSM 20712 [709991]
Draconibacterium orientale [1168034]
Mucinivorans hirudinis [1433126]
Alistipes finegoldii DSM 17242 [679935]
Bacteroidales bacterium CF [1400053]
Sphingobacterium sp. 21 [743722]
Sphingobacterium sp. ML3W [1538644]
Pseudopedobacter saltans DSM 12145 [762903]
Pedobacter heparinus DSM 2366 [485917]
Solitalea canadensis DSM 3403 [929556]
Spirosoma radiotolerans [1379870]
Spirosoma linguale DSM 74 [504472]
ULC179-bin4
Runella slithyformis DSM 19594 [761193]
Dyadobacter fermentans DSM 18053 [471854]
Leadbetterella byssophila DSM 17132 [649349]
Emticicia oligotrophica DSM 17448 [929562]
Cytophaga hutchinsonii ATCC 33406 [269798]
Hymenobacter swuensis DY53 [1227739]
Hymenobacter sp. APR13 [1356852]
Hymenobacter sp. DG25B [1385664]
Pontibacter korlensis [400092]
inset B
Echinicola vietnamensis DSM 17526 [926556]
Belliella baltica DSM 15883 [866536]
Cyclobacterium marinum DSM 745 [880070]
Marivirga tractuosa DSM 4126 [643867]
Cardinium endosymbiont cEper1 of Encarsia pergandiella [1231626]
Candidatus Amoebophilus asiaticus 5a2 [452471]
Bernardetia litoralis DSM 6794 [880071]
ULC066-bin2
Niastella koreensis GR20-10 [700598]
Niabella soli DSM 19437 [929713]
ULC335-bin3
Chitinophaga pinensis DSM 2588 [485918]
Saprospira grandis str. Lewin [984262]
Haliscomenobacter hydrossis DSM 1100 [760192]
inset A
Rhodothermus marinus SG0.5JP17-172 [762570]
Rhodothermus marinus DSM 4252 [518766]
Salinibacter ruber M8 [761659]
Salinibacter ruber DSM 13855 [309807]
Pelodictyon phaeoclathratiforme BU-1 [324925]
Chlorobium chlorochromatii CaD3 [340177]
Chlorobium phaeovibrioides DSM 265 [290318]
Chlorobium luteolum DSM 273 [319225]
Chlorobium phaeobacteroides DSM 266 [290317]
Chlorobium limicola DSM 245 [290315]
Chlorobaculum tepidum TLS [194439]
Chlorobaculum parvum NCIB 8327 [517417]
Prosthecochloris aestuarii DSM 271 [290512]
Chlorobium phaeobacteroides BS1 [331678]
Chloroherpeton thalassium ATCC 35110 [517418]
Melioribacter roseus P3M-2 [1191523]
Ignavibacterium album JCM 16511 [945713]
Gemmatirosa kalamazoonesis [861299]
Gemmatimonas aurantiaca T-27 [379066]
Fibrobacter succinogenes subsp. succinogenes S85 [59374]
Candidatus Cloacimonas acidaminovorans str. Evry [459349]
Chlamydia trachomatis RC-J(s)/122 [907270]
Chlamydia trachomatis RC-J/953 [907267]
Chlamydia trachomatis J/6276tet1 [907272]
Chlamydia trachomatis 6276 [564416]
Chlamydia trachomatis G/9301 [718219]
Chlamydia trachomatis G/9768 [707185]
Chlamydia trachomatis G/11074 [707187]
Chlamydia trachomatis Ia/SotonIa3 [1071767]
Chlamydia trachomatis Ia/SotonIa1 [1071766]
Chlamydia trachomatis D-LC [759364]

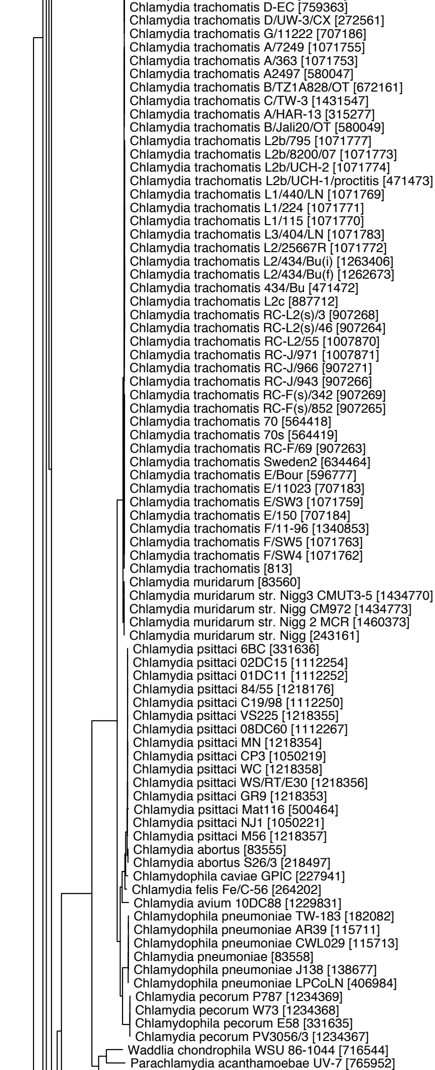

Chlamydia trachomatis D-EC [759363]
Chlamydia trachomatis D/UW-3/CX [272561]
Chlamydia trachomatis G/11222 [707186]
Chlamydia trachomatis A/7249 [1071755]
Chlamydia trachomatis A/363 [1071753]
Chlamydia trachomatis A2497 [580047]
Chlamydia trachomatis B/TZ1A828/OT [672161]
Chlamydia trachomatis C/TW-3 [1431547]
Chlamydia trachomatis A/HAR-13 [315277]
Chlamydia trachomatis B/Jali20/OT [580049]
Chlamydia trachomatis L2b/795 [1071777]
Chlamydia trachomatis L2b/8200/07 [1071773]
Chlamydia trachomatis L2b/UCH-2 [1071774]
Chlamydia trachomatis L2b/UCH-1/proctitis [471473]
Chlamydia trachomatis L1/440/LN [1071769]
Chlamydia trachomatis L1/224 [1071771]
Chlamydia trachomatis L1/115 [1071770]
Chlamydia trachomatis L3/404/LN [1071783]
Chlamydia trachomatis L2/25667R [1071772]
Chlamydia trachomatis L2/434/Bu(i) [1263406]
Chlamydia trachomatis L2/434/Bu(f) [1262673]
Chlamydia trachomatis 434/Bu [471472]
Chlamydia trachomatis L2c [887712]
Chlamydia trachomatis RC-L2(s)/3 [907268]
Chlamydia trachomatis RC-L2(s)/46 [907264]
Chlamydia trachomatis RC-L2/55 [1007870]
Chlamydia trachomatis RC-J/971 [1007871]
Chlamydia trachomatis RC-J/966 [907271]
Chlamydia trachomatis RC-J/943 [907266]
Chlamydia trachomatis RC-F(s)/342 [907269]
Chlamydia trachomatis RC-F(s)/852 [907265]
Chlamydia trachomatis 70 [564418]
Chlamydia trachomatis 70s [564419]
Chlamydia trachomatis RC-F/69 [907263]
Chlamydia trachomatis Sweden2 [634464]
Chlamydia trachomatis E/Bour [596777]
Chlamydia trachomatis E/11023 [707183]
Chlamydia trachomatis E/SW3 [1071759]
Chlamydia trachomatis E/150 [707184]
Chlamydia trachomatis F/11-96 [1340853]
Chlamydia trachomatis F/SW5 [1071763]
Chlamydia trachomatis F/SW4 [1071762]
Chlamydia trachomatis [813]
Chlamydia muridarum [83560]
Chlamydia muridarum str. Nigg3 CMUT3-5 [1434770]
Chlamydia muridarum str. Nigg CM972 [1434773]
Chlamydia muridarum str. Nigg 2 MCR [1460373]
Chlamydia muridarum str. Nigg [243161]
Chlamydia psittaci 6BC [331636]
Chlamydia psittaci 02DC15 [1112254]
Chlamydia psittaci 01DC11 [1112252]
Chlamydia psittaci 84/55 [1218176]
Chlamydia psittaci C19/98 [1112250]
Chlamydia psittaci VS225 [1218355]
Chlamydia psittaci 08DC60 [1112267]
Chlamydia psittaci MN [1218354]
Chlamydia psittaci CP3 [1050219]
Chlamydia psittaci WC [1218358]
Chlamydia psittaci WS/RT/E30 [1218356]
Chlamydia psittaci GR9 [1218353]
Chlamydia psittaci Mat116 [500464]
Chlamydia psittaci NJ1 [1050221]
Chlamydia psittaci M56 [1218357]
Chlamydia abortus [83555]
Chlamydia abortus S26/3 [218497]
Chlamydophila caviae GPIC [227941]
Chlamydia felis Fe/C-56 [264202]
Chlamydia avium 10DC88 [1229831]
Chlamydophila pneumoniae TW-183 [182082]
Chlamydophila pneumoniae AR39 [115711]
Chlamydophila pneumoniae CWL029 [115713]
Chlamydia pneumoniae [83558]
Chlamydophila pneumoniae J138 [138677]
Chlamydophila pneumoniae LPCoLN [406984]
Chlamydia pecorum P787 [1234369]
Chlamydia pecorum W73 [1234368]
Chlamydophila pecorum E58 [331635]
Chlamydia pecorum PV3056/3 [1234367]
Waddlia chondrophila WSU 86-1044 [716544]
Parachlamydia acanthamoebae UV-7 [765952]

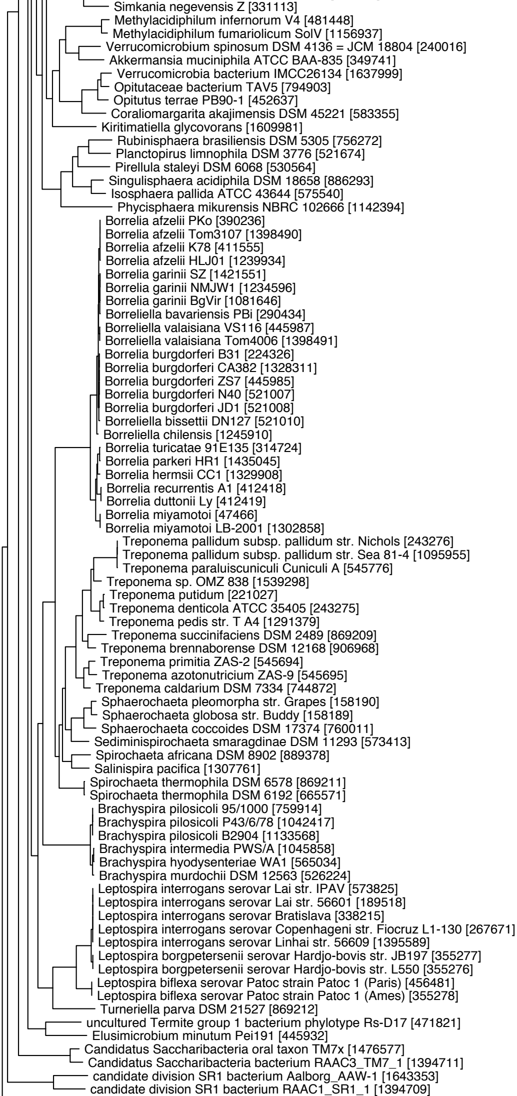
Simkania negevensis Z [331113]
Methylacidiphilum infernorum V4 [481448]
Methylacidiphilum fumariolicum SolV [1156937]
Verrucomicrobium spinosum DSM 4136 = JCM 18804 [240016]
Akkermansia muciniphila ATCC BAA-835 [349741]
Verrucomicrobia bacterium IMCC26134 [1637999]
Opitutaceae bacterium TAV5 [794903]
Opitutus terrae PB90-1 [452637]
Coraliomargarita akajimensis DSM 45221 [583355]
Kiritimatiella glycovorans [1609981]
Rubinisphaera brasiliensis DSM 5305 [756272]
Planctopirus limnophila DSM 3776 [521674]
Pirellula staleyi DSM 6068 [530564]
Singulisphaera acidiphila DSM 18658 [886293]
Isosphaera pallida ATCC 43644 [575540]
Phycisphaera mikurensis NBRC 102666 [1142394]
Borrelia afzelii PKo [390236]
Borrelia afzelii Tom3107 [1398490]
Borrelia afzelii K78 [411555]
Borrelia afzelii HLJ01 [1239934]
Borrelia garinii SZ [1421551]
Borrelia garinii NMJW1 [1234596]
Borrelia garinii BgVir [1081646]
Borreliella bavariensis PBi [290434]
Borreliella valaisiana VS116 [445987]
Borreliella valaisiana Tom4006 [1398491]
Borrelia burgdorferi B31 [224326]
Borrelia burgdorferi CA382 [1328311]
Borrelia burgdorferi ZS7 [445985]
Borrelia burgdorferi N40 [521007]
Borrelia burgdorferi JD1 [521008]
Borreliella bissettii DN127 [521010]
Borreliella chilensis [1245910]
Borrelia turicatae 91E135 [314724]
Borrelia parkeri HR1 [1435045]
Borrelia hermsii CC1 [1329908]
Borrelia recurrentis A1 [412418]
Borrelia duttonii Ly [412419]
Borrelia miyamotoi [47466]
Borrelia miyamotoi LB-2001 [1302858]
Treponema pallidum subsp. pallidum str. Nichols [243276]
Treponema pallidum subsp. pallidum str. Sea 81-4 [1095955]
Treponema paraluiscuniculi Cuniculi A [545776]
Treponema sp. OMZ 838 [1539298]
Treponema putidum [221027]
Treponema denticola ATCC 35405 [243275]
Treponema pedis str. T A4 [1291379]
Treponema succinifaciens DSM 2489 [869209]
Treponema brennaborense DSM 12168 [906968]
Treponema primitia ZAS-2 [545694]
Treponema azotonutricium ZAS-9 [545695]
Treponema caldarium DSM 7334 [744872]
Sphaerochaeta pleomorpha str. Grapes [158190]
Sphaerochaeta globosa str. Buddy [158189]
Sphaerochaeta coccoides DSM 17374 [760011]
Sediminispirochaeta smaragdinae DSM 11293 [573413]
Spirochaeta africana DSM 8902 [889378]
Salinispira pacifica [1307761]
Spirochaeta thermophila DSM 6578 [869211]
Spirochaeta thermophila DSM 6192 [665571]
Brachyspira pilosicoli 95/1000 [759914]
Brachyspira pilosicoli P43/6/78 [1042417]
Brachyspira pilosicoli B2904 [1133568]
Brachyspira intermedia PWS/A [1045858]
Brachyspira hyodysenteriae WA1 [565034]
Brachyspira murdochii DSM 12563 [526224]
Leptospira interrogans serovar Lai str. IPAV [573825]
Leptospira interrogans serovar Lai str. 56601 [189518]
Leptospira interrogans serovar Bratislava [338215]
Leptospira interrogans serovar Copenhageni str. Fiocruz L1-130 [267671]
Leptospira interrogans serovar Linhai str. 56609 [1395589]
Leptospira borgpetersenii serovar Hardjo-bovis str. JB197 [355277]
Leptospira borgpetersenii serovar Hardjo-bovis str. L550 [355276]
Leptospira biflexa serovar Patoc strain Patoc 1 (Paris) [456481]
Leptospira biflexa serovar Patoc strain Patoc 1 (Ames) [355278]
Turneriella parva DSM 21527 [869212]
uncultured Termite group 1 bacterium phylotype Rs-D17 [471821]
Elusimicrobium minutum Pei191 [445932]
Candidatus Saccharibacteria oral taxon TM7x [1476577]
Candidatus Saccharibacteria bacterium RAAC3_TM7_1 [1394711]
candidate division SR1 bacterium Aalborg_AAW-1 [1643353]
candidate division SR1 bacterium RAAC1_SR1_1 [1394709]

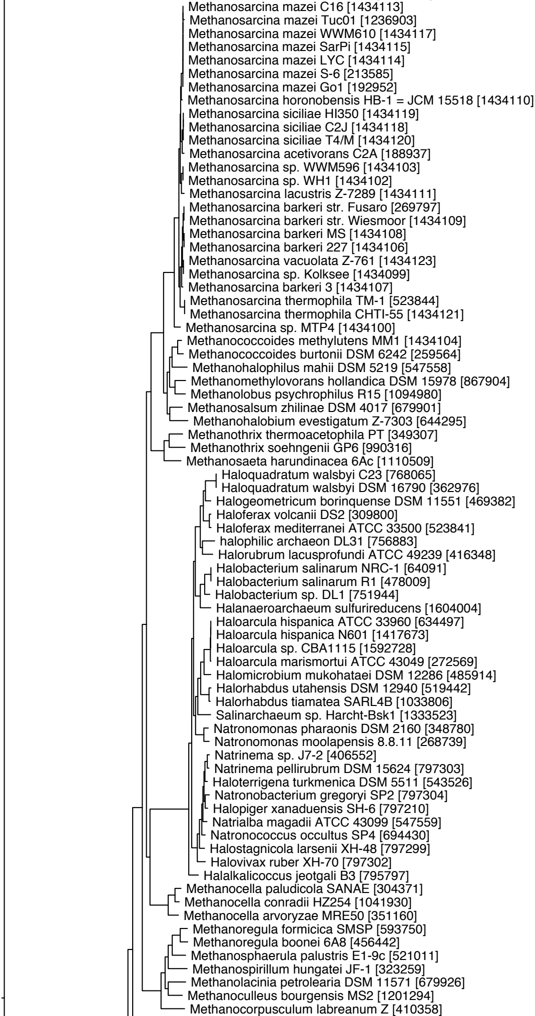

Methanosarcina mazei C16 [1434113]
Methanosarcina mazei Tuc01 [1236903]
Methanosarcina mazei WWM610 [1434117]
Methanosarcina mazei SarPi [1434115]
Methanosarcina mazei LYC [1434114]
Methanosarcina mazei S-6 [213585]
Methanosarcina mazei Go1 [192952]
Methanosarcina horonobensis HB-1 = JCM 15518 [1434110]
Methanosarcina siciliae HI350 [1434119]
Methanosarcina siciliae C2J [1434118]
Methanosarcina siciliae T4/M [1434120]
Methanosarcina acetivorans C2A [188937]
Methanosarcina sp. WWM596 [1434103]
Methanosarcina sp. WH1 [1434102]
Methanosarcina lacustris Z-7289 [1434111]
Methanosarcina barkeri str. Fusaro [269797]
Methanosarcina barkeri str. Wiesmoor [1434109]
Methanosarcina barkeri MS [1434108]
Methanosarcina barkeri 227 [1434106]
Methanosarcina vacuolata Z-761 [1434123]
Methanosarcina sp. Kolksee [1434099]
Methanosarcina barkeri 3 [1434107]
Methanosarcina thermophila TM-1 [523844]
Methanosarcina thermophila CHTI-55 [1434121]
Methanosarcina sp. MTP4 [1434100]
Methanococcoides methylutens MM1 [1434104]
Methanococcoides burtonii DSM 6242 [259564]
Methanohalophilus mahii DSM 5219 [547558]
Methanomethylovorans hollandica DSM 15978 [867904]
Methanolobus psychrophilus R15 [1094980]
Methanosalsum zhilinae DSM 4017 [679901]
Methanohalobium evestigatum Z-7303 [644295]
Methanothrix thermoacetophila PT [349307]
Methanothrix soehngenii GP6 [990316]
Methanosaeta harundinacea 6Ac [1110509]
Haloquadratum walsbyi C23 [768065]
Haloquadratum walsbyi DSM 16790 [362976]
Halogeometricum borinquense DSM 11551 [469382]
Haloferax volcanii DS2 [309800]
Haloferax mediterranei ATCC 33500 [523841]
halophilic archaeon DL31 [756883]
Halorubrum lacusprofundi ATCC 49239 [416348]
Halobacterium salinarum NRC-1 [64091]
Halobacterium salinarum R1 [478009]
Halobacterium sp. DL1 [751944]
Halanaeroarchaeum sulfurireducens [1604004]
Haloarcula hispanica ATCC 33960 [634497]
Haloarcula hispanica N601 [1417673]
Haloarcula sp. CBA1115 [1592728]
Haloarcula marismortui ATCC 43049 [272569]
Halomicrobium mukohataei DSM 12286 [485914]
Halorhabdus utahensis DSM 12940 [519442]
Halorhabdus tiamatea SARL4B [1033806]
Salinarchaeum sp. Harcht-Bsk1 [1333523]
Natronomonas pharaonis DSM 2160 [348780]
Natronomonas moolapensis 8.8.11 [268739]
Natrinema sp. J7-2 [406552]
Natrinema pellirubrum DSM 15624 [797303]
Haloterrigena turkmenica DSM 5511 [543526]
Natronobacterium gregoryi SP2 [797304]
Halopiger xanaduensis SH-6 [797210]
Natrialba magadii ATCC 43099 [547559]
Natronococcus occultus SP4 [694430]
Halostagnicola larsenii XH-48 [797299]
Halovivax ruber XH-70 [797302]
Halalkalicoccus jeotgali B3 [795797]
Methanocella paludicola SANAE [304371]
Methanocella conradii HZ254 [1041930]
Methanocella arvoryzae MRE50 [351160]
Methanoregula formicica SMSP [593750]
Methanoregula boonei 6A8 [456442]
Methanosphaerula palustris E1-9c [521011]
Methanospirillum hungatei JF-1 [323259]
Methanolacinia petrolearia DSM 11571 [679926]
Methanoculleus bourgensis MS2 [1201294]
Methanocorpusculum labreanum Z [410358]

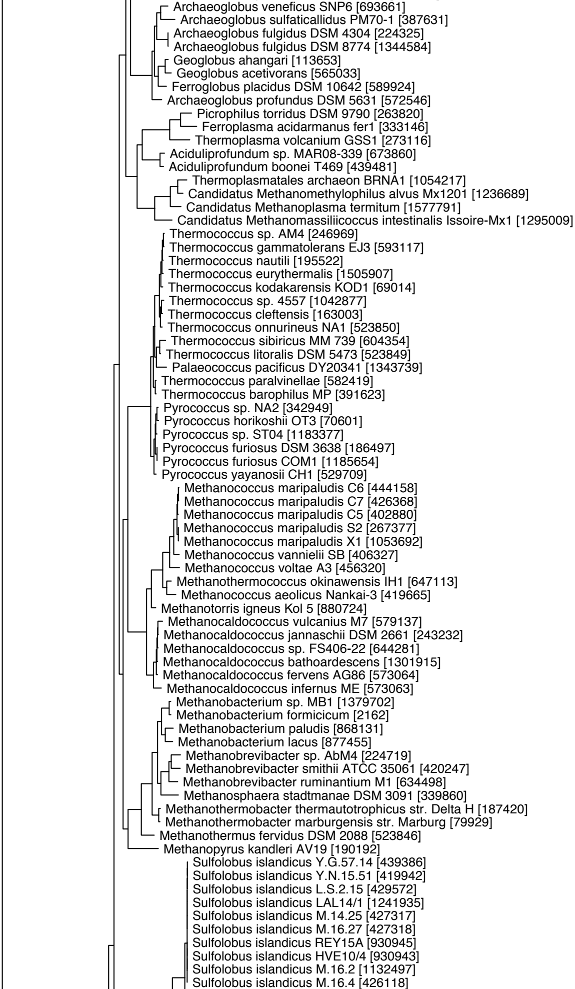
Archaeoglobus veneficus SNP6 [693661]
Archaeoglobus sulfaticallidus PM70-1 [387631]
Archaeoglobus fulgidus DSM 4304 [224325]
Archaeoglobus fulgidus DSM 8774 [1344584]
Geoglobus ahangari [113653]
Geoglobus acetivorans [565033]
Ferroglobus placidus DSM 10642 [589924]
Archaeoglobus profundus DSM 5631 [572546]
Picrophilus torridus DSM 9790 [263820]
Ferroplasma acidarmanus fer1 [333146]
Thermoplasma volcanium GSS1 [273116]
Aciduliprofundum sp. MAR08-339 [673860]
Aciduliprofundum boonei T469 [439481]
Thermoplasmatales archaeon BRNA1 [1054217]
Candidatus Methanomethylophilus alvus Mx1201 [1236689]
Candidatus Methanoplasma termitum [1577791]
Candidatus Methanomassiliicoccus intestinalis Issoire-Mx1 [1295009]
Thermococcus sp. AM4 [246969]
Thermococcus gammatolerans EJ3 [593117]
Thermococcus nautili [195522]
Thermococcus eurythermalis [1505907]
Thermococcus kodakarensis KOD1 [69014]
Thermococcus sp. 4557 [1042877]
Thermococcus cleftensis [163003]
Thermococcus onnurineus NA1 [523850]
Thermococcus sibiricus MM 739 [604354]
Thermococcus litoralis DSM 5473 [523849]
Palaeococcus pacificus DY20341 [1343739]
Thermococcus paralvinellae [582419]
Thermococcus barophilus MP [391623]
Pyrococcus sp. NA2 [342949]
Pyrococcus horikoshii OT3 [70601]
Pyrococcus sp. ST04 [1183377]
Pyrococcus furiosus DSM 3638 [186497]
Pyrococcus furiosus COM1 [1185654]
Pyrococcus yayanosii CH1 [529709]
Methanococcus maripaludis C6 [444158]
Methanococcus maripaludis C7 [426368]
Methanococcus maripaludis C5 [402880]
Methanococcus maripaludis S2 [267377]
Methanococcus maripaludis X1 [1053692]
Methanococcus vannielii SB [406327]
Methanococcus voltae A3 [456320]
Methanothermococcus okinawensis IH1 [647113]
Methanococcus aeolicus Nankai-3 [419665]
Methanotorris igneus Kol 5 [880724]
Methanocaldococcus vulcanius M7 [579137]
Methanocaldococcus jannaschii DSM 2661 [243232]
Methanocaldococcus sp. FS406-22 [644281]
Methanocaldococcus bathoardescens [1301915]
Methanocaldococcus fervens AG86 [573064]
Methanocaldococcus infernus ME [573063]
Methanobacterium sp. MB1 [1379702]
Methanobacterium formicicum [2162]
Methanobacterium paludis [868131]
Methanobacterium lacus [877455]
Methanobrevibacter sp. AbM4 [224719]
Methanobrevibacter smithii ATCC 35061 [420247]
Methanobrevibacter ruminantium M1 [634498]
Methanosphaera stadtmanae DSM 3091 [339860]
Methanothermobacter thermautotrophicus str. Delta H [187420]
Methanothermobacter marburgensis str. Marburg [79929]
Methanothermus fervidus DSM 2088 [523846]
Methanopyrus kandleri AV19 [190192]
Sulfolobus islandicus Y.G.57.14 [439386]
Sulfolobus islandicus Y.N.15.51 [419942]
Sulfolobus islandicus L.S.2.15 [429572]
Sulfolobus islandicus LAL14/1 [1241935]
Sulfolobus islandicus M.14.25 [427317]
Sulfolobus islandicus M.16.27 [427318]
Sulfolobus islandicus REY15A [930945]
Sulfolobus islandicus HVE10/4 [930943]
Sulfolobus islandicus M.16.2 [1132497]
Sulfolobus islandicus M.16.4 [426118]

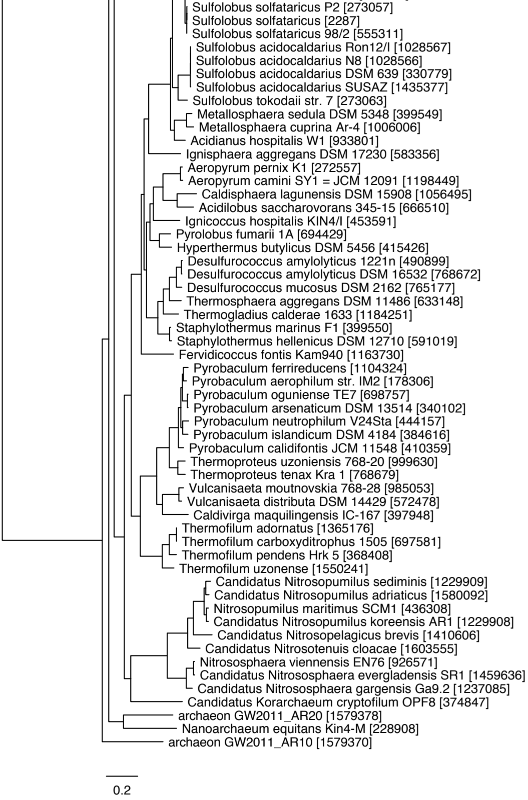
Sulfolobus solfataricus P2 [273057]
Sulfolobus solfataricus [2287]
Sulfolobus solfataricus 98/2 [555311]
Sulfolobus acidocaldarius Ron12/I [1028567]
Sulfolobus acidocaldarius N8 [1028566]
Sulfolobus acidocaldarius DSM 639 [330779]
Sulfolobus acidocaldarius SUSAZ [1435377]
Sulfolobus tokodaii str. 7 [273063]
Metallosphaera sedula DSM 5348 [399549]
Metallosphaera cuprina Ar-4 [1006006]
Acidianus hospitalis W1 [933801]
Ignisphaera aggregans DSM 17230 [583356]
Aeropyrum pernix K1 [272557]
Aeropyrum camini SY1 = JCM 12091 [1198449]
Caldisphaera lagunensis DSM 15908 [1056495]
Acidilobus saccharovorans 345-15 [666510]
Ignicoccus hospitalis KIN4/I [453591]
Pyrolobus fumarii 1A [694429]
Hyperthermus butylicus DSM 5456 [415426]
Desulfurococcus amylolyticus 1221n [490899]
Desulfurococcus amylolyticus DSM 16532 [768672]
Desulfurococcus mucosus DSM 2162 [765177]
Thermosphaera aggregans DSM 11486 [633148]
Thermogladius calderae 1633 [1184251]
Staphylothermus marinus F1 [399550]
Staphylothermus hellenicus DSM 12710 [591019]
Fervidicoccus fontis Kam940 [1163730]
Pyrobaculum ferrireducens [1104324]
Pyrobaculum aerophilum str. IM2 [178306]
Pyrobaculum oguniense TE7 [698757]
Pyrobaculum arsenaticum DSM 13514 [340102]
Pyrobaculum neutrophilum V24Sta [444157]
Pyrobaculum islandicum DSM 4184 [384616]
Pyrobaculum calidifontis JCM 11548 [410359]
Thermoproteus uzoniensis 768-20 [999630]
Thermoproteus tenax Kra 1 [768679]
Vulcanisaeta moutnovskia 768-28 [985053]
Vulcanisaeta distributa DSM 14429 [572478]
Caldivirga maquilingensis IC-167 [397948]
Thermofilum adornatus [1365176]
Thermofilum carboxyditrophus 1505 [697581]
Thermofilum pendens Hrk 5 [368408]
Thermofilum uzonense [1550241]
Candidatus Nitrosopumilus sediminis [1229909]
Candidatus Nitrosopumilus adriaticus [1580092]
Nitrosopumilus maritimus SCM1 [436308]
Candidatus Nitrosopumilus koreensis AR1 [1229908]
Candidatus Nitrosopelagicus brevis [1410606]
Candidatus Nitrosotenuis cloacae [1603555]
Nitrososphaera viennensis EN76 [926571]
Candidatus Nitrososphaera evergladensis SR1 [1459636]
Candidatus Nitrososphaera gargensis Ga9.2 [1237085]
Candidatus Korarchaeum cryptofilum OPF8 [374847]
archaeon GW2011_AR20 [1579378]
Nanoarchaeum equitans Kin4-M [228908]
archaeon GW2011_AR10 [1579370]
0.2
